# Calcium-tolerant electrode strategy for efficient hydrogen peroxide electrosynthesis in natural water

**DOI:** 10.1038/s41467-026-77022-0

**Published:** 2026-08-31

**Authors:** Ke Wang, Ying Zhu, Yuan Yao, Ke Wang

**Affiliations:** 1 State Key Laboratory of Urban-rural Water Resource and Environment, Harbin Institute of Technology, Harbin, China; 2 School of Environment, Harbin Institute of Technology, Harbin, China; 3 National Engineering Research Center for Safe Sludge Disposal and Resource Recovery, Harbin Institute of Technology, Harbin, China

**Keywords:** Electrocatalysis, Electrocatalysis, Electrocatalysis

## Abstract

Calcium scaling is traditionally regarded as a major impediment to the practical application of decentralized H_2_O_2_ electrosynthesis via two-electron oxygen reduction reaction (2e^−^ ORR). Herein, we demonstrate that the calcium scale unexpectedly enhances H_2_O_2_ electroproduction. Dissolved Ca^2+^, rather than precipitated scale, is the dominant factor suppressing H_2_O_2_ electrosynthesis. Interfacial enrichment of Ca^2+^ facilitates water dissociation and proton accessibility, shifting the pathway toward 4e^−^ ORR. To address this challenge, a quaternary ammonium cationic surfactant-modified cathode is developed, demonstrating a 2.3-fold enhancement in H_2_O_2_ yield. Theoretical calculations suggest that surfactant modification weakens Ca^2+^ adsorption and reduces its interfacial accumulation through enhanced electrostatic repulsion. Simultaneously, hydrophobic side-chain grafting decreases proton accessibility by disrupting the interfacial hydrogen-bond network, thereby improving 2e^−^ ORR selectivity. Furthermore, a 3D polyurethane sponge-fenced cathode architecture establishes an alkaline microenvironment via pore confinement, mitigating the Ca^2+^-enhanced proton accessibility and increasing H_2_O_2_ electroproduction by 293.3%. These strategies of interfacial field manipulation and 3D alkaline confinement provide a promising paradigm for designing Ca^2+^-tolerant cathode for efficient H_2_O_2_ electrosynthesis.

## Abstract (web summary)

Calcium scaling is widely considered a major barrier to decentralized H_2_O_2_ electrosynthesis. Here, the authors identify dissolved Ca^2+^ as the key factor limiting H_2_O_2_ production and develop calcium tolerant cathodes through interfacial microenvironment engineering for efficient H_2_O_2_ production.

## Introduction

As a green oxidizing agent, hydrogen peroxide (H_2_O_2_) is extensively employed in environmental applications, particularly for water disinfection^1,2^, pollutant degradation^3,4^, and algae elimination^5^. However, the conventional anthraquinone process for H_2_O_2_ production is associated with a substantial carbon footprint^6^. The electrochemical two-electron oxygen reduction reaction (2e^−^ ORR) presents a potentially sustainable and green alternative strategy for H_2_O_2_ synthesis. Nevertheless, current catalyst design approaches predominantly focus on idealized pure water systems, failing to address the complexity of real water matrices, which severely limits their practical applicability.

Electrode scaling is widely recognized as a major factor limiting the practical application of electrochemical technologies in natural water systems^7,8^. Inorganic cations are ubiquitous in natural water, with divalent cations such as Ca^2+^ frequently causing cathode contamination and consequent performance deterioration^9^. Conventional understanding primarily attributes performance degradation exclusively to calcium scaling, which can cover active sites and increase system impedance^10–12^. However, emerging research indicates that alkali metal cations at the cathode-electrolyte interface can influence electrochemical processes through various mechanisms, including altering intermediate adsorption strength and modifying interfacial hydrogen-bond networks^13–16^. In contrast, the specific impact of dissolved Ca^2+^ on the cathodic 2e^−^ ORR remains largely unexplored. Therefore, elucidating the respective effects of different calcium species (dissolved Ca^2+^ versus precipitated calcium scale) on the 2e^−^ ORR is crucial for designing highly Ca^2+^-tolerant cathodes.

Despite considerable progress in developing catalysts and innovative reactor configurations for the 2e^−^ ORR in recent years, most efforts target pure water systems, with limited attention paid to Ca^2+^-tolerant electrocatalytic systems. Interface modulation strategies have recently garnered significant attention in electrochemical field. Modification approaches employing ionic liquids and cationic polymers enable precise control over electrode surface electrostatic potential and wettability^17,18^. These modifications subsequently induce ion redistribution and alter hydrogen-bond networks at the electrode interface, thereby influencing electrochemical reaction pathways^19,20^. Nevertheless, the influence and underlying mechanism of interfacial field modulation on the electrocatalytic behavior in Ca^2+^-containing systems represent a critical knowledge gap. Furthermore, three-dimensional electrode architectures can significantly impact the migration and transformation of calcium species by modifying the mass transport of OH^−^ near the cathode in Ca^2+^-containing electrolytes^21^. This is anticipated to exert an influence on the cathodic 2e^−^ ORR in the presence of Ca^2+^, which remains entirely unexplored.

This work provides perspectives into the distinct roles of dissolved Ca^2+^ versus calcium scale in 2e^−^ ORR and establishes a paradigm for designing Ca^2+^-tolerant cathodes for H_2_O_2_ electrosynthesis. Combining experimental and theoretical approaches, we aim to: (1) comprehensively elucidate the mechanistic impacts of both dissolved and precipitated calcium species on the 2e^−^ ORR; (2) achieve tunable electrostatic potential and interface wettability through electrostatic self‑assembly of quaternary ammonium cationic surfactants, thereby enhancing the 2e^−^ ORR selectivity in Ca^2+^‑containing systems and clarifying the intrinsic mechanism underlying the high Ca^2+^-tolerance; and (3) further propose a 3D polyurethane sponge (PUS) fenced cathode configuration to facilitate an alkaline microenvironment for efficient H_2_O_2_ electrosynthesis.

## Results

### Mechanistic insights into the Ca^2+^-induced variations in 2e^−^ ORR performance

Oxygen-doped carbon black (named C0) was synthesized via nitric acid oxidation method and was employed as a model catalyst to investigate the impact of Ca^2+^ on the electrocatalytic performance for the 2e^−^ ORR, considering its high selectivity and cost-effectiveness^22^. The introduction of 200 mg L^−1^ Ca^2+^ suppressed H_2_O_2_ electrosynthesis to varying degrees under all current densities tested (Supplementary Fig. 1). The extent of cathode scaling exhibited a current dependence, increasing significantly with higher currents, consistently supported by the inductively coupled plasma optical emission spectrometry (ICP-OES) results, scanning electron microscopy (SEM) images, and 3D surface profilometry (Supplementary Figs. 2–5 and Supplementary Table 1). However, the increase in calcium scaling did not lead to a monotonic decrease in the Faradaic efficiencies (FEs) for H_2_O_2_ electroproduction. Conversely, under high current conditions, the enhanced alkalinity in the cathode region significantly promoted scaling formation, which substantially increased the FEs to levels comparable to those observed in the absence of Ca^2+^ (Supplementary Fig. 1). This finding contradicts the conventional view that scaling necessarily leads to performance degradation and suggests that calcium scale may not be the primary factor inhibiting the 2e^−^ ORR performance. Instead, dissolved Ca^2+^ likely plays a significant role in modulating the electrocatalytic behavior. To elucidate the intrinsic mechanism of Ca^2+^-induced performance variation, the potential effects of different calcium species on 2e^−^ ORR were initially investigated.

#### Evidence for the suppression of 2e^−^ ORR by dissolved Ca^2+^

**Fig. 1:**
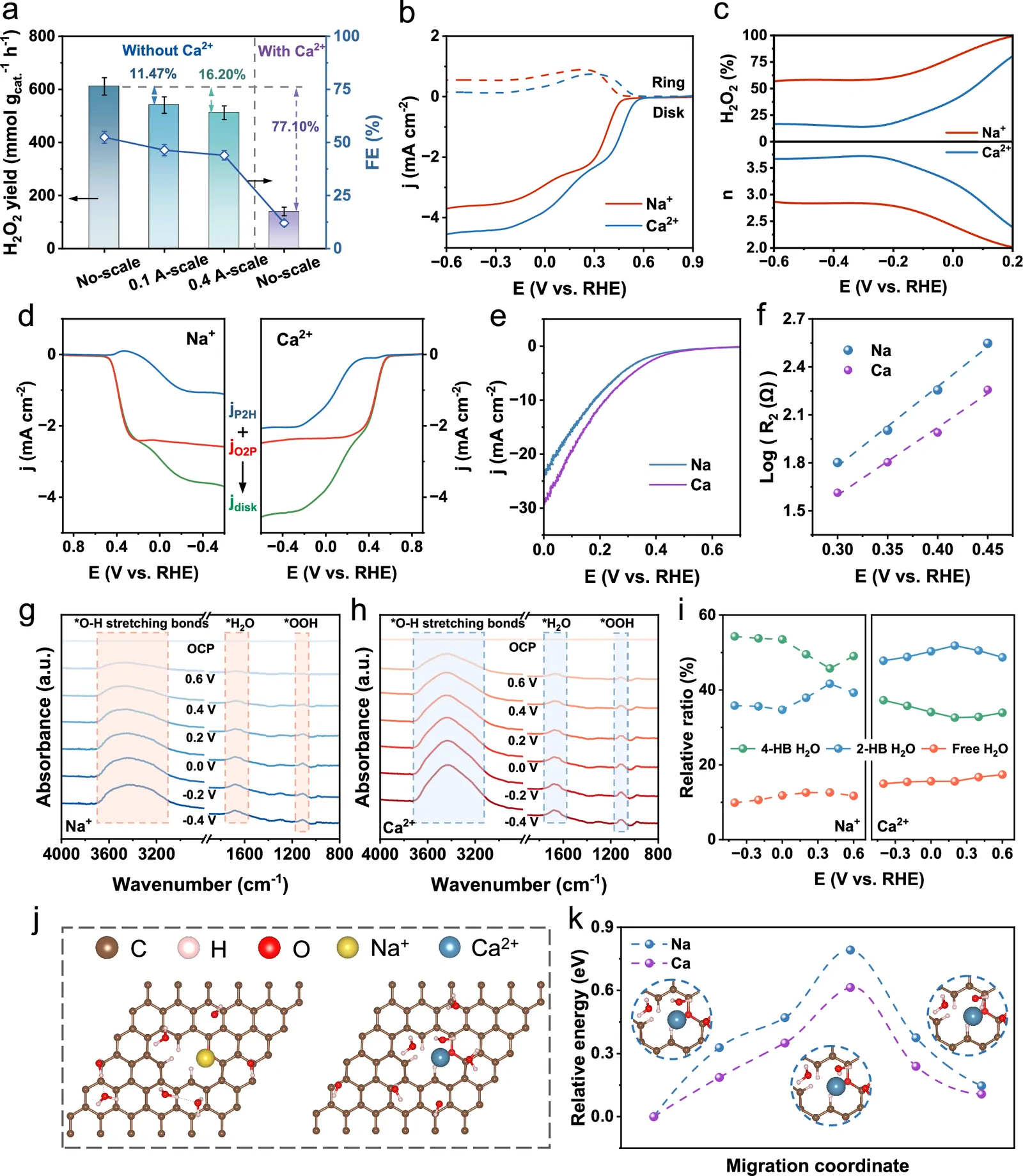
Effects of dissolved Ca^2+^ on 2e^−^ ORR for oxygen-doped carbon black (named C0). **a** H_2_O_2_ electrosynthesis performance of C0 cathodes with varying degrees of scaling in 0.05 M Na_2_SO_4_ and no-scale cathode in 0.05 M Na_2_SO_4_ and 200 mg L^−1^ Ca^2+^. Reaction conditions: a current of 0.1 A, a rotation speed of 600 rpm, an initial pH of 7.0 ± 0.1, a temperature of 25 °C, and electrolysis of 3 h in an undivided reactor. **b** LSV curves of C0 from RRDE tested at 1600 rpm in O_2_-saturated 0.1 M NaCl or 0.1 M CaCl_2_. Reaction conditions: an initial pH of 7.0 ± 0.1, an O_2_ flow rate of 100 mL min^−1^, a scan rate of 10 mV s^−1^, and a temperature of 25 °C in a single-cell reactor. **c** The electron transfer number and H_2_O_2_ selectivity of C0 in different systems. **d** Deconvolution analysis of disk currents in Na^+^ and Ca^2+^ systems. **e** LSV curves and **f** EIS-derived Tafel plots obtained from the hydrogen adsorption resistance R_2_ of C0 in N_2_-saturated 0.1 M NaCl or CaCl_2_ electrolytes containing 10 mM H_2_O_2_. Reaction conditions: an initial pH of 7.0 ± 0.1, a N_2_ flow rate of 100 mL min^−1^, a scan rate of 5 mV s^−1^ and a temperature of 25 °C in a H-type cell. In-situ ATR-SEIRAS spectra for C0 in O_2_-saturated **g** 0.1 M NaCl and **h** 0.1 M CaCl_2_. **i** The relative ratio of 4-HB-H_2_O, 2-HB-H_2_O and free H_2_O for C0 in Na^+^ and Ca^2+^ systems. **j** Theoretical models of the oxygen-doped graphene surface with Na^+^ and Ca^2+^. **k** Transition states and energy profiles of water dissociation process. The insets show molecular models of different stages for water dissociation. Data in **a** is presented as mean ± s.d. (*n* = 3). All the potentials have not been iR-corrected. Source data for Fig. 1 are provided as a Source data file.

It is important to distinguish the physical effects of scaling from the chemical catalytic effect of dissolved Ca^2+^ on the observed performance degradation. To decouple these contributions, the H_2_O_2_ electrosynthesis performance of scaled cathodes was directly evaluated in Ca^2+^-free systems, thereby preserving the physical consequences of scaling while eliminating dissolved Ca^2+^. The 0.1 A-scaled C0 cathode achieved 46.40% FE in Ca^2+^-free electrolyte, whereas the fresh C0 cathode reached only 12.00% FE in the presence of 200 mg L^−1^ Ca^2+^ (Fig. 1a and Supplementary Fig. 6). To further quantify these contributions, we roughly partitioned the overall performance loss into dissolved Ca^2+^-induced and scale-induced components (Supplementary Fig. 7). For the C0 cathode at 0.1 A, the total performance decay attributed to calcium species was 77.10%, of which dissolved Ca^2+^ contributed 85.12% and deposited scale accounted for only 14.88%. Linear sweep voltammetry (LSV) curves of cathodes with different scaling degrees further supported these observations (Supplementary Fig. 8). Despite contaminated with calcium scale, the scaled cathodes retained most of their ORR activity. Control experiments with NaCl addition were performed to isolate the effect of Cl^−^ co-introduced during Ca^2+^ addition, confirming that chloride species contributed negligibly to the observed variations in cathodic H_2_O_2_ production (Supplementary Fig. 9). These collective results demonstrate that dissolved Ca^2+^ exerts the dominant cationic effect on suppressing the 2e^−^ ORR performance, while precipitated calcium scale plays a secondary but non-negligible role under the tested conditions.

To provide direct in-situ evidence for the Ca^2+^-induced ORR pathway shift, rotating ring-disk electrode (RRDE) measurements were performed in O_2_-saturated electrolyte (Fig. 1b). In the presence of Ca^2+^, the electron transfer number (*n*) increased and the H_2_O_2_ selectivity correspondingly decreased across the entire potential range compared to the Na^+^ system (Fig. 1c), confirming that dissolved Ca^2+^ dynamically shifts the ORR pathway toward the 4e^−^ direction. To further identify the specific kinetic step affected, the total ORR current (j_D_) was deconvoluted into two sequential reactions: oxygen-to-peroxide (O2P) and peroxide-to-hydroxide (P2H) (Fig. 1d)^23^. The kinetic current for O2P (j_O2P_) showed negligible difference between the Na^+^ and Ca^2+^ systems, indicating that dissolved Ca^2+^ does not directly inhibit the 2e^−^ reduction of O_2_ to H_2_O_2_. In contrast, the kinetic current for P2H (j_P2H_) was substantially enhanced in the Ca^2+^-containing electrolyte, demonstrating that Ca^2+^ accelerates the parasitic electroreduction of in-situ generated H_2_O_2_ to H_2_O. These results provide in-situ kinetic evidence that dissolved Ca^2+^ suppresses net H_2_O_2_ electrosynthesis primarily by promoting the further reduction of H_2_O_2_ rather than by inhibiting the O_2_-to-H_2_O_2_ step itself.

Considering the inevitable decomposition of H_2_O_2_ at both the anode and cathode in a single-chamber reactor, the influence of Ca^2+^ on H_2_O_2_ decomposition was systematically investigated. It is worth noting that neither Ca^2+^ nor CaCO_3_ exhibited any significant effect on the chemical decomposition of H_2_O_2_ in the absence of an electric field (Supplementary Fig. 10). Moreover, while H_2_O_2_ is unavoidably consumed at the anode, the presence of Ca^2+^ did not measurably alter its decomposition rate (Supplementary Fig. 11). This can be attributed to the inability of Ca^2+^ to accumulate effectively at the anode-electrolyte interface under an applied electric field. To further elucidate the mechanism by which dissolved Ca^2+^ suppress the 2e^−^ ORR, electrochemical testing was employed to investigate the influence of dissolved Ca^2+^ on the cathodic H_2_O_2_ reduction reaction. Both LSV and electrochemical impedance spectroscopy (EIS) measurements were conducted in an H-type electrolytic cell separated by a cation exchange membrane (CEM). Compared to the Na^+^ system, the Ca^2+^ system for C0 cathode exhibited a more positive onset potential and a higher reduction current (Fig. 1e), demonstrating the promoting effect of Ca^2+^ on the cathodic H_2_O_2_ reduction. The EIS-derived Tafel plots revealed that the hydrogen adsorption resistance in the Ca^2+^ system was lower than that in the Na^+^ system at all tested potentials (Fig. 1f, Supplementary Fig. 12, Supplementary Tables 2, 3). This indicates that Ca^2+^ accelerates the reduction of H_2_O_2_ at the cathode by enhancing proton transfer kinetics, thereby favoring a shift in the ORR pathway from 2e^−^ to 4e^−^ route. LSV and EIS tests were further conducted in NaCl solutions of different concentrations to rule out the influence of ionic strength. The comparable reduction currents and hydrogen adsorption resistances observed in the 0.1 M and 0.2 M Na^+^ systems suggest that the impact of ionic strength on the cathodic H_2_O_2_ reduction is negligible (Supplementary Fig. 13).

The reaction intermediates and interfacial water structures were directly probed using in situ electrochemical attenuated total reflection surface-enhanced infrared absorption spectroscopy (ATR-SEIRAS). Notably, the characteristic *OOH band (~1100 cm^−1^) in the Ca^2+^ system was even stronger than that in the Na^+^ system (Fig. 1g, h), suggesting that dissolved Ca^2+^ does not impede the O2P step by inhibiting *OOH formation^24^. The reduced *OOH adsorption energy collectively indicates a stronger stabilizing effect of Ca^2+^ on *OOH intermediate (Supplementary Data 1 and Supplementary Fig. 14). In contrast, the Ca^2+^ system demonstrated enhanced signals associated with *H_2_O (~1670 cm^−1^) and O-H stretching vibrations (~3000–3700 cm^−1^) (Fig. 1g, h), indicating that dissolved Ca^2+^ can enhance interfacial H_2_O adsorption and activation. The O-H stretching modes can be further deconvoluted into tetrahedrally coordinated (4-HB-H_2_O), doubly coordinated (2-HB-H_2_O), and free water^25^. Among these species, 2-HB-H_2_O is more favorable for water dissociation toward proton generation^26,27^. Compared with Na^+^, Ca^2+^ promoted the conversion of 4-HB-H_2_O into 2-HB-H_2_O and free water (Fig. 1i and Supplementary Fig. 15). Consequently, Ca^2+^ is more favorable for interfacial water adsorption and dissociation, thereby enhancing proton availability and accelerating the further reduction of H_2_O_2_ to H_2_O. These in situ spectroscopic results provide direct evidence that dissolved Ca^2+^ primarily affects the 2e^−^ ORR selectivity by regulating interfacial water activation and proton availability, rather than by suppressing *OOH formation in the O_2_-to-H_2_O_2_ pathway.

DFT calculations were further employed to elucidate the critical role of Ca^2+^ in promoting water dissociation (Supplementary Data 1). A monolayer graphene structure incorporating an ether group and an adjacent carbon vacancy defect was constructed as a representative model for DFT calculations (Fig. 1j and Supplementary Figs. 16, 17), considering the experimental surface chemistry of C0 and the active site configuration associated with high 2e^−^ ORR selectivity in the literature^28^. The calculation results indicate that Ca^2+^ lowers the activation energy barrier for water dissociation (H_2_O→H^+^ + OH^−^), thereby enhancing the dissociation process (Fig. 1k). To further elucidate the origin of Ca^2+^-accelerated interfacial water dissociation, the role of interfacial electric field was examined. Charge transfer between the slab and electrolyte was quantified using Bader charge analysis, and the interfacial electric field strength was estimated based on a parallel-plate capacitor model. Under applied electric field, the H-OH bond of interfacial H_2_O is polarized through *H-OH^δ−^-M^+^ interactions, as established in previous fundamental studies^16^. The Ca^2+^ system exhibits an enhanced electric field strength, accompanied by elongation of the H-OH bond, indicating that Ca^2+^ facilitates the water dissociation step by promoting interfacial H_2_O polarization (Supplementary Fig. 18). Due to the simplified models and idealized conditions adopted in DFT calculations, the obtained results should be interpreted as qualitative theoretical guidance rather than quantitative predictions of the real catalytic system. Taken together, we propound that the accumulation of dissolved Ca^2+^ at the cathode promote water dissociation and proton availability at the cathode-electrolyte interface, consequently facilitating the further reduction of in-situ generated H_2_O_2_ to H_2_O and diminishing the 2e^−^ ORR selectivity.

#### Dual effect of precipitated calcium scale on 2e^−^ ORR

The influence of precipitated calcium scale on 2e^−^ ORR was further evaluated. Previous studies have indicated that scaling typically exerts adverse effects on cathode catalytic performance through covering active sites and enhancing surface wettability^9–12^. As shown in Fig. 1a, the formation of calcium scale indeed impaired the cathodic H_2_O_2_ electrosynthesis performance, although not critically. To investigate the underlying mechanism of the performance decline in the 2e^−^ ORR, the wettability evolution was evaluated for cathodes with varying scaling degrees. The results revealed that calcium scale formation significantly improved the hydrophilicity of the cathodes, with the surface of the 0.4 A-scaled cathodes becoming completely wetted (Supplementary Fig. 19). This enhanced hydrophilicity can aggravate water flooding (Supplementary Fig. 20), thereby inhibiting oxygen transport and ultimately leading to decreased H_2_O_2_ electroproduction^29^. Electrochemical characterizations were further conducted to investigate the influence of calcium scaling on cathodic active sites, represented by the electrochemical active surface area (ECSA)^30^. However, all scaling cathodes demonstrated higher ECSA than that of fresh cathodes (Supplementary Fig. 21). This is attributed to the scaling-induced enhancement in cathode wettability, which expands the contact area between the cathode and the electrolyte.

Notably, experimental results in Supplementary Fig. 1 demonstrated that under Ca^2+^ conditions, increased calcium scaling did not monotonically suppress H_2_O_2_ electroproduction, but paradoxically enhanced the 2e^−^ ORR selectivity. To elucidate the dual roles of precipitated calcium scale, we decoupled the H_2_O_2_ production and decomposition processes, systematically investigating its effect on cathodic H_2_O_2_ reduction reaction. It was proved that calcium scaling effectively suppressed H_2_O_2_ decomposition at the cathode beyond a certain threshold (Supplementary Fig. 22). LSV curves for H_2_O_2_ reduction further corroborated these findings (Supplementary Fig. 23). EIS was employed to further probe the influence of scaling on proton transfer during H_2_O_2_ reduction. All scaled cathodes exhibited higher hydrogen adsorption resistance than pristine cathodes, indicating slower proton transfer kinetics (Supplementary Fig. 24 and Supplementary Table 4). These findings demonstrate that precipitated calcium scale can effectively reduce proton availability at the cathode interface, thereby inhibiting further H_2_O_2_ reduction to H_2_O and enhancing 2e^−^ ORR selectivity. This conclusion aligns with the H_2_O_2_ decomposition experiments, confirming the protective role of precipitated calcium scale in H_2_O_2_ electroproduction.

It is worth noting that the accumulation of calcium scale over longer time can further exacerbate electrode flooding, potentially leading to negative effects that far outweigh any protective role. Nevertheless, we emphasize that even at early stages, the detrimental effect of dissolved Ca^2+^ on 2e^−^ ORR selectivity is already severe enough to cause system failure. This suggests that managing dissolved Ca^2+^ should take precedence over controlling calcium scale deposition in the design of electrochemical systems.

### Characterization of as-prepared surfactant modified catalysts

**Fig. 2:**
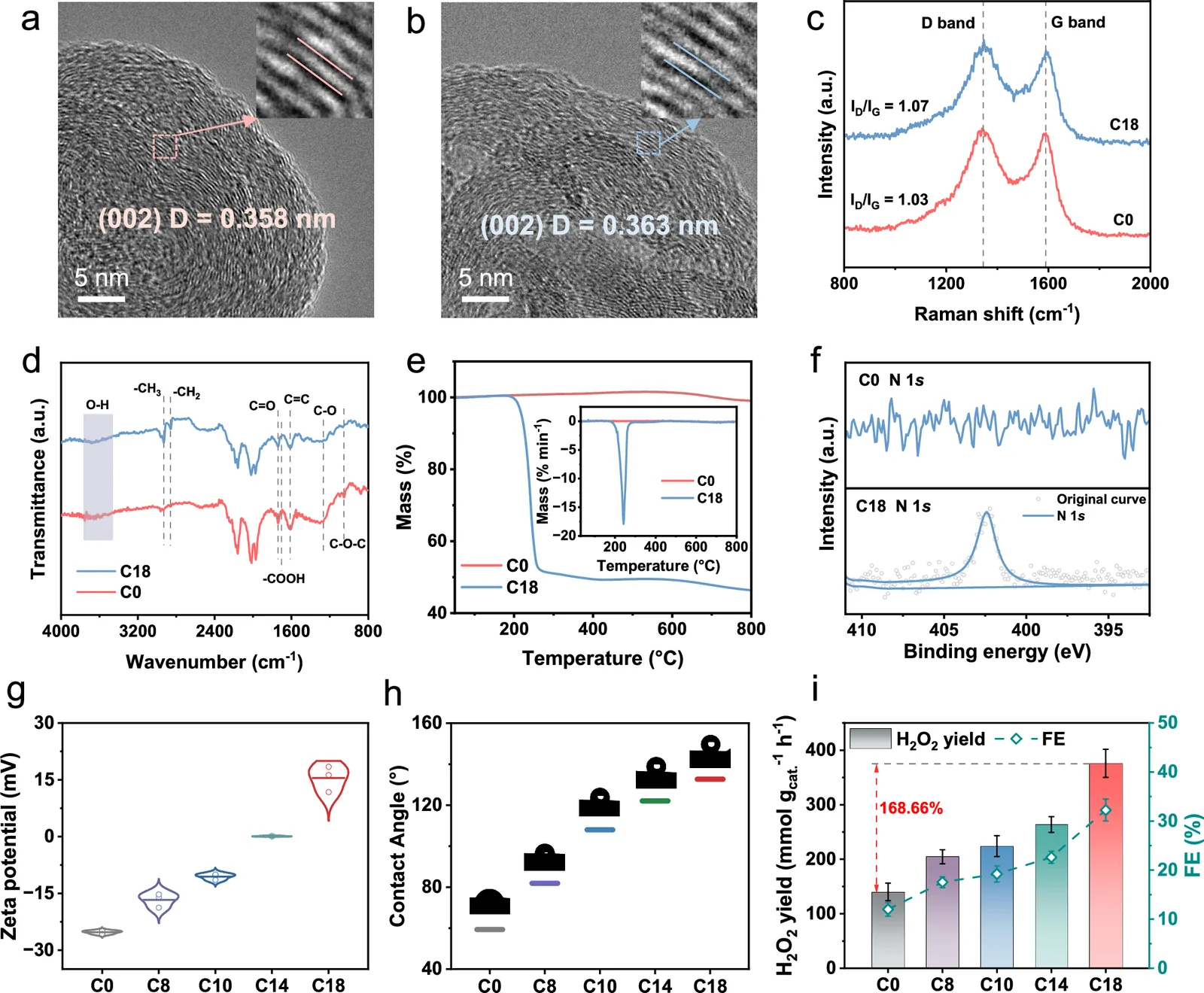
Characterization of as-prepared surfactant modified catalysts. HRTEM images of **a** C0 and **b** C18. The insets show the lattice fringes. **c** Raman spectra, **d** FTIR spectra, **e** TG curves, and **f** N 1 *s* XPS spectra of C0 and C18. The inset in **e** shows the corresponding DTG curves of C0 and C18, representing the mass-loss rate as a function of temperature. **g** Zeta potential and **h** contact angle measurements of different surfactant-modified catalysts. **i** H_2_O_2_ electrosynthesis yields and Faraday efficiencies of different surfactant-modified cathodes. Reaction conditions: [Na_2_SO_4_] = 0.05 M, [Ca^2+^]_0_ = 200 mg L^−1^, a current of 0.1 A, a rotation speed of 600 rpm, electrolysis time of 3 h, an initial pH of 7.0 ± 0.1 and a temperature of 25 °C in an undivided reactor. Data in **i** is presented as mean ± s.d. (*n* = 3). Source data for Fig. 2 are provided as a Source data file.

Based on the mechanism of 2e^−^ ORR performance degradation dominated by dissolved Ca^2+^, an interfacial field modulation strategy was proposed to regulate the interaction between the cathode surface and Ca^2+^, thereby enhancing the 2e^−^ ORR selectivity in Ca^2+^-containing systems. Four types of quaternary ammonium surfactants (OTAB, DTAB, TTAB and STAB) were employed to modify C0 with tunable surface wettability and electrostatic potential through electrostatic self-assembly (named C8, C10, C14 and C18). The SEM and transmission electron microscopy (TEM) images exhibited similar spherical layered morphologies of both C0 and C18 (Supplementary Fig. 25). However, the average particle size of C18 (44.4 nm) was slightly larger than that of C0 (42.0 nm), which can be attributed to the electrostatic self-assembly of STAB on the surface of C0. The high-resolution transmission electron microscopy (HRTEM) images indicated that the lattice spacings of both C0 and C18 were approximately 0.36 nm, corresponding to the (002) crystal plane of carbon materials (Fig. 2a, b)^31^. The energy-dispersive X-ray spectroscopy (EDX) results in Supplementary Fig. 26 demonstrated that C, O, and N elements were uniformly distributed on the surface of C18, confirming the successful incorporation of STAB. The X-ray diffraction (XRD) patterns of all samples exhibited two typical broad peaks at ≈ 25° and ≈ 43° (Supplementary Fig. 27), corresponding to the respective (002) and (101) plane graphite^32,33^, which was consistent with the HRTEM images. Raman spectra in Fig. 2c showed that the I_D_/I_G_ ratio of C18 was slightly higher than that of C0, suggesting a greater degree of structural defects in C18^34,35^.

Fourier transform infrared spectroscopy (FTIR) revealed the presence of prominent oxygen functional groups on the surfaces of both C0 and C18 (Fig. 2d)^36,37^, attributable to the nitric acid oxidation. Compared with C0, C18 exhibited significantly enhanced peak intensities at 2861 and 2935 cm^−1^ (Fig. 2d), originating from the alkyl chains of STAB^38^. Thermogravimetric analysis demonstrated that C0 exhibited negligible mass loss over the tested temperature range, whereas C18 showed a mass loss of approximately 50% between 200 and 300 °C (Fig. 2e), corresponding to the melting point of STAB^39^, thereby confirming the successful grafting of STAB. The surface chemical states of C0 and C18 were further analyzed by X-ray photoelectron spectroscopy (XPS). Both C 1*s* and O 1*s* peaks were observed in the spectra of C0 and C18. However, the N 1*s* peak was exclusively detected in C18, with a nitrogen content of 2.77% (Supplementary Fig. 28). The high-resolution N 1*s* spectrum of C18 displayed a characteristic peak at 402.41 eV, ascribed to the quaternary ammonium nitrogen atoms^40,41^, while no distinct characteristic peaks were observed in C0 (Fig. 2f). These results provided further evidence for the successful assembly of STAB on the C0 surface.

Furthermore, Zeta potential analysis in Fig. 2g demonstrated that the electrostatic self-assembly of quaternary ammonium surfactants with varying chain lengths shifted the zeta potential from −25.26 mV (C0) to 15.48 mV (C18), indicating successful modulation of the surface electrostatic potential of carbon materials. The distinct electrostatic potential of the samples may result in contrary interactions with Ca^2+^, consequently influencing their 2e^−^ ORR selectivity in Ca^2+^-containing electrolytes. Additionally, as the length of the hydrophobic side chain increases, the contact angle rose from 59.34° (C0) to 132.67° (C18), reflecting a significant enhancement in surface hydrophobicity (Fig. 2h), despite a concurrent reduction in the electrochemical active surface area (ECSA) (Supplementary Fig. 29). These analyses confirmed the feasibility of tailoring both surface electrostatic potential and wettability of carbon materials using quaternary ammonium surfactants with different chain lengths, demonstrating its potential in enhancing H_2_O_2_ electrosynthesis by modifying the microenvironment at the cathode interface.

### 2e^−^ ORR performance and scale formation of surfactant-modified cathodes in Ca^2+^-containing electrolytes

**Fig. 3:**
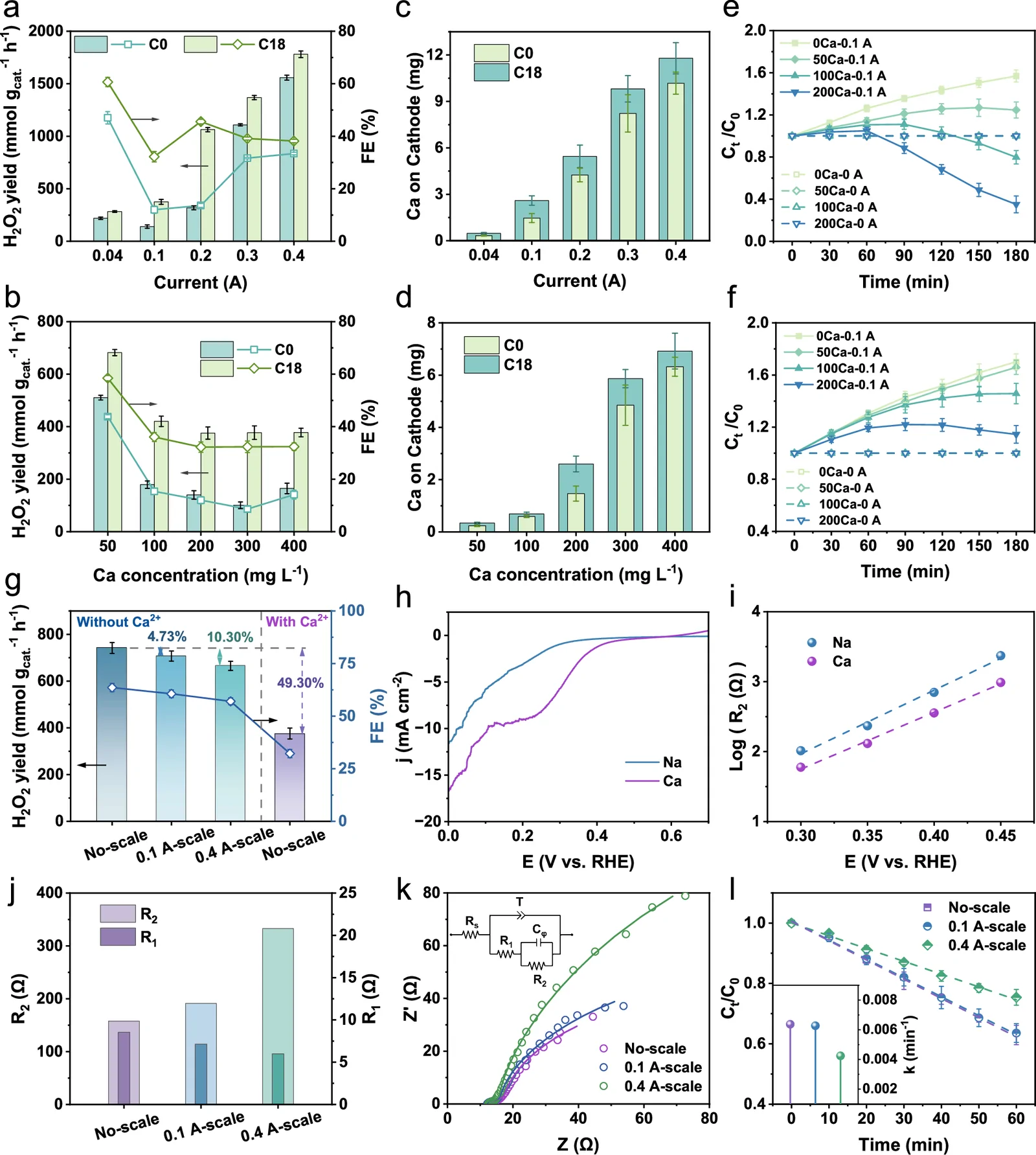
Enhanced H_2_O_2_ electrosynthesis performance of C18. Effects of **a** current densities and **b** initial Ca^2+^ concentrations on H_2_O_2_ electrosynthesis performance for C0 and C18 in the presence of Ca^2+^. The mass of calcium scale on cathode under different **c** current densities and **d** initial Ca^2+^ concentrations. H_2_O_2_ electrosynthesis performance of **e** C0 and **f** C18 under varying Ca^2+^ concentrations at an initial 900 mg L^−1^ H_2_O_2_. **g** H_2_O_2_ electrosynthesis performance of C18 cathodes with varying degrees of scaling in 0.05 M Na_2_SO_4_ and no-scale cathode in 0.05 M Na_2_SO_4_ and 200 mg L^−1^ Ca^2+^. **h** LSV curves and **i** EIS-derived Tafel plots obtained from the hydrogen adsorption resistance R_2_ of C18 in N_2_-saturated 0.1 M NaCl or 0.1 M CaCl_2_ electrolytes containing 10 mM H_2_O_2_. Reaction conditions: an initial pH of 7.0 ± 0.1, a N_2_ flow rate of 100 mL min^−1^, a scan rate of 5 mV s^−1^, and a temperature of 25 °C in a H-type cell. **j** Impedance components and **k** EIS Nyquist plots of H_2_O_2_ reduction for C18 cathodes with varying degrees of scaling in N_2_-saturated (a N_2_ flow rate of 100 mL min^−1^) 0.1 M Na_2_SO_4_ solution containing 10 mM H_2_O_2_ in a H-type cell. The inset shows the equivalent circuit. **l** H_2_O_2_ decomposition curves and decomposition rate constant k of C18 cathodes with varying degrees of scaling at an initial H_2_O_2_ concentration of 1200 mg L^−1^, a current of 0.1 A, a rotation speed of 600 rpm, an initial pH of 7.0 ± 0.1 and a temperature of 25 °C in a dual-chamber reactor. Unless otherwise specified, the reaction conditions for (**a**–**g**) are as follows: [Na_2_SO_4_] = 0.05 M, [Ca^2+^]_0_ = 200 mg L^−1^, a current of 0.1 A, a rotation speed of 600 rpm, an initial pH of 7.0 ± 0.1, a temperature of 25 °C, and electrolysis of 3 h in an undivided reactor. Data in (**a**–**g**, **l**) are presented as mean ± s.d. (*n* = 3). All the potentials have not been iR-corrected. Source data for Fig. 3 are provided as a Source data file.

To elucidate the influence of quaternary ammonium surfactant grafting with different chain lengths on the selectivity of 2e^−^ ORR in Ca^2+^-containing systems, the H_2_O_2_ electroproduction performance of five cathodes (C0, C8, C10, C14, C18) was evaluated. In the absence of Ca^2+^, C18 exhibited higher H_2_O_2_ electrosynthesis performance (Supplementary Fig. 30) despite its significantly reduced ECSA (0.94 vs 3.01 mF cm^−2^ for C0). This can be attributed to the enhanced hydrophobicity of C18, which facilitates oxygen mass transport by mitigating water flooding^42^, and simultaneously promotes the desorption and diffusion of in-situ generated H_2_O_2_ into the bulk solution, thereby suppressing its parasitic reduction to H_2_O^43^, as will be discussed in detail below. Upon introduction of Ca^2+^ into the system, the FEs of all cathodes decreased to varying degrees. However, C18 exhibited significantly higher tolerance to Ca^2+^ compared to C0. In the presence of 200 mg L^−1^ Ca^2+^, the FE of C18 (32.25%) was 168.66% higher than that of C0 (12.00%) (Fig. 2i and Supplementary Fig. 31). To further investigate the effects of surface electrostatic potential and hydrophobicity on cathode tolerance to Ca^2+^, the H_2_O_2_ electrosynthesis performance and scaling behaviors of C0 and C18 cathodes were evaluated under different currents and Ca^2+^ concentrations (Fig. 3a, b and Supplementary Figs. 32, 33). C18 demonstrated enhanced Ca^2+^ tolerance compared to C0 under all tested conditions, although their performance was comparable in the absence of Ca^2+^ (Supplementary Fig. 34). In the presence of 200 mg L^−1^ Ca^2+^ at 0.2 A, the FE of C18 (45.60%) was 3.34 times that of C0 (13.65%). However, under identical conditions, the scaling extent on C18 was more severe than C0, supported by the ICP-OES results (Fig. 3c, d) and post-reaction cathode observations (Supplementary Figs. 35, 36). This phenomenon challenges the prevailing paradigm that attributes performance decay to calcium scale deposition^10,11^. Notably, the presence of high H_2_O_2_ concentration further confirmed the enhanced Ca^2+^ tolerance of C18, with its advantage being more pronounced at elevated H_2_O_2_ concentrations (Fig. 3e, f and Supplementary Fig. 37).

A comprehensive characterization of C0 and C18 cathodes before and after electrolysis was further conducted. Compared to the pre-electrolysis state, the diffraction peaks corresponding to vaterite and calcite appeared in the XRD patterns of both cathodes after electrolysis, suggesting that the scale primarily consists of calcium carbonate (CaCO_3_) (Supplementary Fig. 38)^44,45^. The FTIR results further confirmed the formation of CaCO_3_ on the cathode surfaces (Supplementary Fig. 39)^46,47^. The SEM coupled with EDX and 3D surface profilometry were employed to obtain detailed information on the surface morphology and elemental distribution of the cathodes. Both cathodes exhibited significant scaling after electrolysis, with the calcium scale morphology comprising a mixture of amorphous structures, vaterite and calcite (Supplementary Figs. 3, 40). No significant differences in scale morphology were observed between C0 and C18, consistent with the XRD and FTIR results. Notably, the EDS quantification indicated a higher degree of scaling on C18 compared to C0, aligning with the ICP-OES data and 3D surface profilometry results (Supplementary Figs. 5, 41). Furthermore, the cross-sectional SEM-EDS analysis revealed that calcium scale was predominantly deposited on the surface of both C0 and C18 cathodes, with no fundamental difference in the spatial location of scale formation between the two electrodes (Supplementary Figs. 4, 42). These characterization results provide evidence of the high similarity in calcium scale morphology and location between C0 and C18 cathodes. Combined with the anomalous difference in scaling quantity, this further suggests that the enhanced Ca^2+^ tolerance of C18 does not stem from a reduction in scaling amount or alterations in scale morphology and location. These findings prompt further investigations into the underlying mechanism of the high Ca^2+^ tolerance exhibited by C18.

It is noteworthy that the mechanistic role of dissolved Ca^2+^ versus precipitated calcium scale in modulating the 2e^−^ ORR selectivity also applies to the C18 cathode. This observation further supports the main contribution of dissolved Ca^2+^ and the secondary contribution of deposited calcium scale to the suppression of H_2_O_2_ electroproduction (Fig. 3g and Supplementary Figs. 43–45). Nevertheless, C18 exhibited a more negative onset potential and a lower current density compared to C0 in both Na^+^ and Ca^2+^ systems for the cathodic H_2_O_2_ reduction reaction (Fig. 3h). This behavior is attributed to its slower proton transfer kinetics (Fig. 3i and Supplementary Fig. 46), which effectively inhibits the reduction of H_2_O_2_ at the cathode. Moreover, C18 cathodes with varying degrees of scaling consistently demonstrated smaller hydrogen adsorption resistances R_2_ than C0 counterparts during the H_2_O_2_ reduction process (Fig. 3j, k). This results in a further decrease of H_2_O_2_ reduction at the cathode (Fig. 3l, Supplementary Figs. 47, 48), thereby enhancing the selectivity toward the 2e^−^ ORR. Notably, scaling also improved the wettability of the C18 cathode (Supplementary Fig. 49). While it increased the ECSA (Supplementary Fig. 50), the concomitant exacerbation of flooding exerted a negative impact on the electrocatalytic synthesis of H_2_O_2_ (Supplementary Fig. 51).

### Mechanistic study for enhanced Ca^2+^-tolerance of surfactant-modified cathodes

**Fig. 4:**
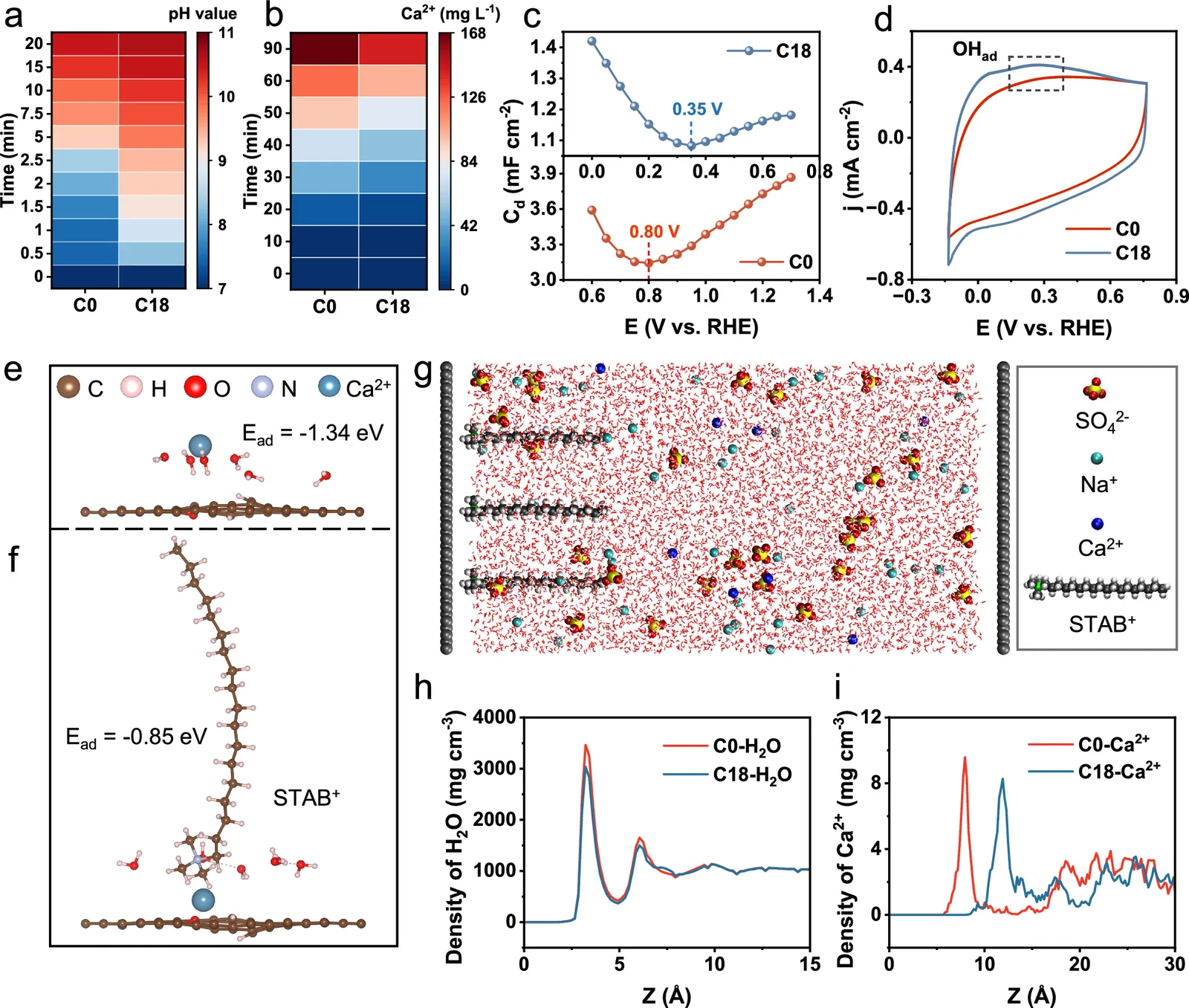
Experimental and theoretical verification of the regulated mass transfer of OH^−^ and Ca^2+^. **a** pH and **b** Ca^2+^ concentration changes versus time during diffusion tests using C0/GF and C18/GF as membranes. **c** Comparison of PZC of C0 and C18. **d** CV curves in N_2_-saturated 0.1 M NaOH. Reaction conditions: an initial pH of 13.0 ± 0.1, a N_2_ flow rate of 100 mL min^−1^, a scan rate of 50 mV s^−1^, and a temperature of 25 °C in a single-chamber cell. The adsorption energies (*E*_ad_) for Ca^2+^ on the surfaces of **e** C0 and **f** C18 cathodes obtained from DFT calculations. **g** Snapshot of the C18 cathode-electrolyte interface in MD simulation. Colors: C in dark gray, H in light gray, O in red, S in yellow, N in green, Na in cyan, and Ca in blue. Radial density distributions of **h** H_2_O and **i** Ca^2+^ for C0 and C18 systems in MD simulation. All the potentials have not been iR-corrected. Source data for Fig. 4 are provided as a Source data file.

Compared to the C0 cathode, the C18 cathode demonstrated significantly enhanced Ca^2+^ tolerance, particularly under high H_2_O_2_ concentrations. However, in the absence of an electric field, no significant difference was observed in the chemical decomposition of H_2_O_2_ between C0 and C18 catalysts (Supplementary Fig. 52). To elucidate the mechanism of the improved Ca^2+^ tolerance achieved through STAB interfacial modification, a series of strategically designed experiments were conducted. Diffusion experiments for OH^−^ and Ca^2+^ were performed using an H-type cell^48^. One compartment was filled with 0.1 M NaOH or 0.1 M CaCl_2_ solution, while the other contained an equal volume of deionized water, separated by a glass fiber (GF) membrane loaded with either C0 or C18. Driven by the concentration gradient, OH^−^ or Ca^2+^ diffused freely into the deionized water compartment. The concentrations of OH^−^ and Ca^2+^ in the compartment were monitored over time, with OH^−^ measured via pH meter and Ca^2+^ quantified by ICP-OES. As shown in Fig. 4a, when using the C0/GF membrane, the pH in the deionized water compartment increased from 7.0 to 10.49 over 20 min, whereas with C18/GF, it rose to 10.66. This difference is attributed to their opposing surface electrostatic potentials. The more positive surface potential of C18 enables electrostatic attraction of OH^−^ during diffusion, thereby forming a more alkaline environment. As anticipated, Ca^2+^ diffusion exhibited the opposite trend to OH^−^. Due to electrostatic repulsion from the positive surface potential and strong hydrophobicity of C18, the Ca^2+^ concentration in the deionized water compartment with C18/GF reached only 142.76 mg L^−1^ after 90 min, compared to 167.88 mg L^−1^ with C0/GF (Fig. 4b). These diffusion measurements provide qualitative evidence for the intrinsic Ca^2+^-surface interactions under open-circuit conditions. To more directly probe Ca^2+^-electrode interactions under polarized conditions, an electrode transfer method was employed to quantify electric-field-driven Ca^2+^ adsorption^16,26^. The electro-adsorbed Ca^2+^ amount on C18 (3.28 mg L^−1^) was significantly lower than that on C0 (14.99 mg L^−1^) (Supplementary Fig. 53), further confirming the substantially reduced affinity of C18 toward Ca^2+^. This effective exclusion of Ca^2+^ significantly mitigates the inhibitory effect of dissolved Ca^2+^ on the 2e^−^ ORR, representing a key factor in the enhanced Ca^2+^-resistance of C18.

To investigate the differences in interfacial properties between C0 and C18, the potential of zero charge (PZC) was determined by the differential capacitance method^49^. Compared with C0 (0.80 V vs RHE), C18 exhibited a pronounced negative shift in PZC (0.35 V vs RHE), attributable to the modification with positively charged quaternary ammonium groups (-N^+^(CH_3_)_3_) on its surface (Fig. 4c)^50^. Under cathodic polarization (typically E < E_PZC_ for both cathodes), the excess negative surface charge is governed by the deviation from PZC (E-E_PZC_)^51^. Therefore, the more negative PZC of C18 can reduce the negative deviation from PZC under identical cathodic polarization, thereby exhibiting less negative charge and weakening the purely electrostatic driving force for Ca^2+^ accumulation. Beyond net surface charge, the STAB modification can substantially alter the electrical double-layer (EDL) structure. The hydrophobic alkyl chains and interfacial organic layer can reduce Ca^2+^ accessibility to the inner Helmholtz plane and suppress specific Ca^2+^ adsorption through steric effects. The influence of surface electrostatic potential on OH^−^ distribution was further investigated by cyclic voltammetry (CV) tests^52^, revealing an enhanced OH_ad_ intensity on the surface of C18 (Fig. 4d). The more positive surface potential of C18 significantly enriched OH^−^ concentration at the interface. The alkaline microenvironment not only kinetically enhances H_2_O_2_ selectivity but also mitigates the cationic effects of dissolved Ca^2+^ while promoting calcium precipitation, collectively suppressing H_2_O_2_ reduction.

To elucidate the mechanism underlying the high Ca^2+^ tolerance of the C18 cathode, DFT calculations were employed to investigate the thermodynamic properties of the C0 and C18 (Supplementary Data 1). The pristine graphene model and the one modified with STAB were used to represent C0 and C18, respectively (Fig. 4e, f and Supplementary Fig. 54). The adsorption energies (E_ad_) of Ca^2+^ on C0 and C18 were −1.34 eV and −0.85 eV, respectively, suggesting a weaker interaction between Ca^2+^ and C18. Moreover, molecular dynamics (MD) simulations were performed across different cathode systems to further unveil the cathode-electrolyte interfacial microenvironment (Fig. 4g and Supplementary Figs. 55, 56). Statistical analysis of radial distributions for interfacial species was conducted after system stabilization (simulation duration of 20 ns). Compared to C0, the C18 exhibited a decrease number of interfacial water molecules and hydrogen bonds (Fig. 4h and Supplementary Fig. 57), indicating that the hydrophobic chains of STAB can disrupt the interfacial water structure and suppress proton availability at the interface. Consequently, the further reduction of H_2_O_2_ to H_2_O is suppressed, favoring the 2e^−^ ORR pathway that requires fewer protons, consistent with the experimental results. The influence of STAB grafting on the interfacial distribution of Ca^2+^ was further analyzed, as shown in Fig. 4i. In the C18 system, the peak Ca^2+^ signal appeared at approximately 11.91 Å, significantly farther from the cathode compared to C0 (~7.95 Å). Additionally, the peak Ca^2+^ density in the C18 system (8.27 mg cm^−3^) was lower than that in C0 (9.58 mg cm^−3^). These findings align with the Ca^2+^ diffusion experiments, suggesting that the more positive surface electrostatic potential of C18 induces strong electrostatic repulsion against Ca^2+^, effectively expelling it from the cathode interface. This further mitigates the negative cationic effects of Ca^2+^ on cathodic H_2_O_2_ electroproduction, thereby enhancing the selectivity toward the 2e^−^ ORR. It should be noted that both DFT calculations and MD simulations rely on simplified structural models and idealized simulation conditions, which cannot fully capture the complexity and dynamic evolution of the real catalyst under operating conditions. Therefore, the simulation results are intended to provide theoretical insights that complement the experimental observations.

Furthermore, to decouple the electrostatic and hydrophobic contributions of the C18 modification, a non-ionic long-chain surfactant-modified cathode (named N-C18) was prepared using 1-octadecanethiol (ODT). XRD, Raman, and XPS spectra confirmed the successful modification of N-C18 (Supplementary Fig. 58). N-C18 exhibited a contact angle of 134.02° (comparable to C18 at 132.67°) while retaining a zeta potential of −23.05 mV (comparable to C0 at −25.26 mV) (Supplementary Fig. 58), thereby isolating the hydrophobic effect from electrostatic repulsion. Compared with C0, N-C18 exhibited a 29.95% improvement in performance in Ca^2+^-containing electrolyte, whereas C18 achieved a 168.66% enhancement (Supplementary Fig. 59). These results indicate that the hydrophobicity-driven mass transport plays a secondary role in enhancing Ca^2+^-tolerance (~17.8% contribution), whereas the electrostatic effect constitutes the dominant mechanism (~82.2% contribution), which mitigates the adverse effects of dissolved Ca^2+^ on H_2_O_2_ electroproduction.

### Enhanced Ca^2+^-tolerance by a fenced cathode structure

**Fig. 5:**
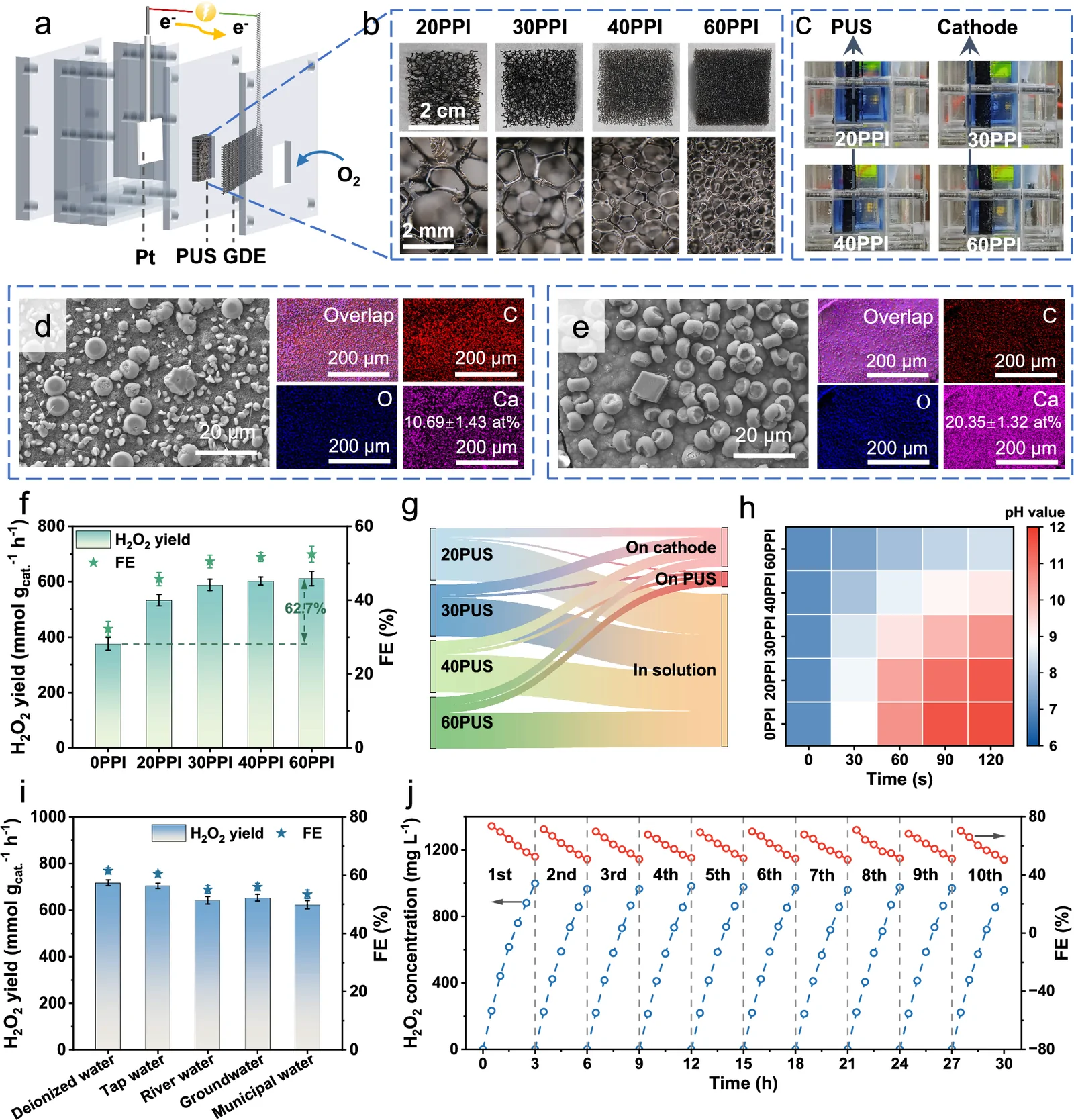
H_2_O_2_ electrosynthesis performance enhanced by fenced cathode structure employing polyurethane sponges (PUS). **a** Schematic illustration of the PUS-fenced cathode system. **b** Optical images of PUS with varying pore sizes. **c** Visualization of the spatial distribution of the alkaline region employing thymolphthalein as the pH indicator in PUS-fenced cathode systems at 120 s. SEM images and EDS mappings of the C18 cathode **d** without and **e** with PUS (60 PPI) after experiment at 0.1 A in the presence of 200 mg L^−1^ Ca^2+^. **f** H_2_O_2_ yield and FE of C18 in PUS-fenced cathode systems with varying pore sizes. **g** Mass balance of Ca in different PUS-fenced systems for C18. **h** The pH of the bulk solution at 1 cm from the cathode in PUS-fenced systems. **i** H_2_O_2_ yield and FE of 60PPI-PUS-fenced C18 cathode in real water matrices. **j** Stability test performed with 60PPI-PUS-fenced cathode of C18 in ten cycles, each of 3 h duration. Unless otherwise specified, the reaction conditions are as follows: [Na_2_SO_4_] = 0.05 M, [Ca^2+^]_0_ = 200 mg L^−1^, a current of 0.1 A, a rotation speed of 600 rpm, an initial pH of 7.0 ± 0.1, a temperature of 25 °C and electrolysis of 3 h in an undivided reactor. Data in **f**, **i** are presented as mean ± s.d. (*n* = 3). Source data for Fig. 5 are provided as a Source data file.

Inspired by the mechanism of enhanced Ca^2+^ tolerance through an alkaline cathode microenvironment, we designed a three-dimensional (3D) foam-fenced cathode architecture to further improve H_2_O_2_ electrosynthesis in Ca^2+^-containing systems. As illustrated in Fig. 5a–c, incorporating a polyurethane sponge (PUS) on the cathode side increases mass transfer resistance for in-situ generated OH^−^, establishing a localized highly alkaline microenvironment and facilitating cathode scaling (Fig. 5d, e). The PUS-fenced cathode dramatically improved H_2_O_2_ electroproduction in Ca^2+^-containing electrolyte. With 200 mg L^−1^ Ca^2+^ at 0.1 A, the C0-PUS cathode achieved 550.26 mmol g_cat._^−1^ h^−1^ H_2_O_2_ yield, representing a 293.25% enhancement over the C0 cathode (139.96 mmol g_cat._^−1^ h^−1^) (Supplementary Fig. 60). Under identical conditions, the C18-PUS cathode exhibited a significantly higher H_2_O_2_ yield of 611.88 mmol g_cat._^−1^ h^−1^, which is 1.63 times that of the C18 cathode (376.04 mmol g_cat._^−1^ h^−1^) (Fig. 5f). The C18-PUS system consistently outperformed the bare C18 cathode across varying Ca^2+^ concentrations and current densities (Supplementary Fig. 61), demonstrating the robust adaptability of the fenced cathode configuration to fluctuations in water chemistry and operational conditions. Notably, the FEs of C0-PUS and C18-PUS cathodes were comparable to that of the bare counterpart in Ca^2+^-free electrolyte (Supplementary Fig. 62). The striking divergence between the Ca^2+^-free and Ca^2+^-containing results directly demonstrates that the PUS-induced enhancement is predominantly Ca^2+^-specific rather than any general alkaline effect. Additional H_2_O_2_ electrosynthesis in Ca^2+^-free systems (Supplementary Fig. 63) and decomposition experiments (Supplementary Fig. 64) with scaled PUS cathodes further corroborated the dual effects of dissolved calcium inhibition and precipitated calcium protection.

The effect of PUS pore size on H_2_O_2_ electroproduction was systematically investigated (Supplementary Fig. 65). For both cathodes, H_2_O_2_ concentration increased with decreasing PUS pore size, with C0 from 455.55 mg L^−1^ (20 PPI) to 898.02 mg L^−1^ (60 PPI) and C18 from 870.72 mg L^−1^ (20 PPI) to 998.58 mg L^−1^ (60 PPI) (Supplementary Fig. 66), attributed to OH^−^ enrichment in confined spaces improving 2e^−^ ORR selectivity. Further analysis of calcium transformation revealed increasing total precipitated calcium with decreasing pore size in both C0-PUS and C18-PUS systems (Fig. 5g and Supplementary Figs. 67, 68), confirming that pore confinement effects of PUS enhance the interfacial alkalinity. Additionally, calcium scaling within the PUS increased as the PUS pore size decreased, particularly at 60 PPI, resulting from combined alkaline intensification and increased nucleation sites^21^. Notably, although facilitating cathode scaling, the introduction of PUS did not alter the scale morphology on the cathode surface, as evidenced by SEM images, XRD, and FTIR patterns (Supplementary Figs. 69–72)^45–47^. Additionally, the enhanced scaling induced by PUS also enhanced the hydrophilicity of the cathodes (Supplementary Fig. 73).

The pH of the bulk solution at 1 cm from the cathode was measured to illustrate the regulatory effect of PUS on alkalinity near the cathode region. The results indicated that a smaller pore size leads to a more pronounced alkaline environment. The bulk pH followed the order: No-PUS > 20 PPI > 30 PPI > 40 PPI > 60 PPI (Fig. 5h). Given the theoretically consistent OH^−^ generation across cathodes, this trend directly confirms that the pore confinement effect of PUS restricts OH^−^ diffusion, thereby enhancing the alkaline environment. Visualization experiments employing thymolphthalein as the pH indicator further corroborated this finding (Supplementary Fig. 74)^53^. Collectively, the 3D foam-fenced cathode system utilizes the pore confinement effect of the PUS to substantially elevate the OH^−^ concentration, creating a highly alkaline environment within the confined near-cathode region. The elevated alkalinity promotes the 2e^−^ ORR pathway by reducing proton availability while simultaneously inducing calcium precipitation, thereby decreasing interfacial dissolved Ca^2+^ concentration and mitigating its adverse effects on the 2e^−^ ORR selectivity.

In summary, we have developed a PUS-fenced cathode architecture that significantly enhances Ca^2+^ tolerance. The C18-PUS system achieved high H_2_O_2_ production rates in tap water, river water, groundwater and municipal wastewater, approaching the performance in deionized water and demonstrating its broad applicability (Fig. 5i). Cycling tests confirmed the stability of PUS-fenced cathodes in Ca^2+^-containing environments, with both C0-PUS and C18-PUS retaining 96% of their initial performance after ten cycles (Fig. 5j and Supplementary Fig. 75). Notably, the operational stability of the electrostatically assembled STAB layer was verified by post-reaction XPS analysis. After 10 cycles (30 h cumulative operation) of H_2_O_2_ electrosynthesis in Ca^2+^-containing electrolyte, the characteristic N 1*s* peak at ~403.0 eV corresponding to the quaternary ammonium nitrogen (-N^+^(CH_3_)_3_) of STAB remained clearly detectable on the C18 cathode (Supplementary Fig. 76), with the N content retaining ~70.3% of its initial value (from 2.73 at% to 1.92 at%). This demonstrates that majority of STAB molecules remain anchored on the cathode surface despite prolonged exposure to the alkaline interfacial microenvironment and the applied electric field. Taken together, the synergistic combination of electrostatic potential modulation and PUS confinement substantially elevates the OH^−^ concentration in the near-cathode region. This highly alkaline environment mitigates the adverse cationic effects of Ca^2+^ and effectively suppresses the transition from the 2e^−^ ORR to the 4e^−^ ORR pathway, thereby enhancing H_2_O_2_ electrosynthesis performance in Ca^2+^-containing systems.

## Discussion

Although electrode scaling is conventionally regarded as the primary cause of performance degradation in electrocatalytic systems, this work proves that the transformation of dissolved Ca^2+^ into solid scale paradoxically enhances the performance of H_2_O_2_ electroproduction. Combining experimental tests and theoretical calculations, we conclusively demonstrate that dissolved Ca^2+^, rather than precipitated scale, constitutes the primary source of performance degradation by accelerating interfacial water dissociation and shifting the reaction pathway toward the 4e^−^ ORR. This provides a perspective to understand the roles of dissolved and precipitated calcium species in electrochemical systems.

Furthermore, two Ca^2+^-tolerance strategies, including cationic surfactant modification and a 3D PUS-fenced cathode architecture, were proposed, boosting the H_2_O_2_ electrosynthesis performance by 234.0% and 293.3%, respectively, in Ca^2+^-containing systems. The quaternary ammonium cationic surfactants regulation creates a cathode interface with positive potential and strong hydrophobicity, electrostatically repelling Ca^2+^ and disrupting the interfacial hydrogen-bond network. This dual action effectively reduces proton accessibility, thereby improving 2e^−^ ORR selectivity. Moreover, the pore confinement effect of the 3D-PUS restricts OH^−^ diffusion and creates a localized alkaline environment within the confined near-cathode region that further inhibits Ca^2+^ activity, thereby enhancing the 2e^−^ ORR selectivity in Ca^2+^-containing environments. The hybrid strategy delivered H_2_O_2_ electrosynthesis performance in four real water matrices comparable to that in deionized water, and retained 96% of its initial performance over 10 intermittent operation cycles (30 h cumulative), demonstrating promising reusability toward dissolved Ca^2+^. In conclusion, this work pioneers two strategies, including surfactant-induced interfacial field control and 3D fenced-cathode confinement, that enables simultaneous Ca^2+^ repulsion, proton-availability suppression, and alkaline microenvironment localization, establishing a paradigm for designing Ca^2+^-tolerant cathodes, with potential promise for extension to natural water matrices.

## Methods

### Chemicals and materials

Carbon paper (SGL Sigracet 28BC) and Nafion (D520, 5 wt%) were obtained from SCI Materials Hub. Commercial carbon black (CB, Cabot Vulcan XC-72R) was purchased from Suzhou Sinero Technology Co., Ltd. Potassium titanium oxalate (C_4_H_2_K_2_O_10_Ti, > 99%), 1-Octadecanethiol (ODT, 99%), Octyltrimethylammonium bromide (OTAB, 98%), decyltrimethylammonium bromide (DTAB, 99%), tetradecyltrimethylammonium bromide (TTAB, 99%), and stearyltrimethylammonium bromide (STAB, 98%) were purchased from Shanghai Macklin Biotechnology Co., Ltd. Isopropyl alcohol (99.7%), anhydrous ethanol (99.7%), thymolphthalein (99%), sulfuric acid (H_2_SO_4_, 98 wt%), sodium hydroxide (NaOH, >99%), hydrogen peroxide (H_2_O_2_, 30 wt%), anhydrous sodium sulfate (Na_2_SO_4_, >99%), sodium chloride (NaCl, >99%) and calcium chloride dihydrate (CaCl_2_·2H_2_O, >99%) were supplied from Chinese Sinopharm Chemical Reagent Co., Ltd. Deionized water (≥18.2 MΩ cm^−1^) was used throughout all experiments.

### Synthesis of surfactants modified oxygen-doped carbon black

Synthesis of oxygen-doped carbon black (OCB). The OCB catalyst (named C0) was synthesized using a conventional nitric acid oxidation method. Specifically, 200 mg of commercial CB (Cabot Vulcan XC-72R) was added into 200 mL of nitric acid (12 M). The above mixture was then refluxed at 85 °C for 12 h. After natural cooling to room temperature, the product was collected by vacuum filtration and thoroughly washed with deionized water and ethanol until neutral pH was achieved. Finally, the sample was vacuum-dried at 60 °C for 24 h to obtain the OCB catalyst.

Synthesis of OTAB-OCB, DTAB-OCB, TTAB-OCB and STAB-OCB (named C8, C10, C14 and C18). To prepare the surfactant modified OCB catalyst, 60 mg of OCB and 240 μmol of cationic surfactant (e.g., OTAB, DTAB, TTAB, or STAB) were added into 200 mL of deionized water, followed by sonication for 1 h to obtain a homogeneous suspension. The final samples were collected through vacuum filtration followed by washing with plenty deionized water, and were subsequently vacuum-dried for 24 h at 60 °C.

Synthesis of ODT-OCB (named N-C18, non-ionic long-chain surfactant-modified C0). To prepare the non-ionic long-chain surfactant-modified OCB catalyst, 60 mg of OCB and 240 μmol of ODT were added into 20 mL of anhydrous ethanol, followed by sonication for 1 h and stirring for 24 h to obtain a homogeneous suspension. The final samples were collected through vacuum filtration followed by washing with plenty anhydrous ethanol, and were subsequently vacuum-dried for 24 h at 60 °C.

### Fabrication of the working electrodes

This study employed gas diffusion electrodes (GDEs) as working electrodes, considering their efficient oxygen mass transfer capability and energy efficiency advantages. For GDEs fabrication, 8 mg of catalyst sample was dispersed in a mixed solution containing 950 μL of isopropyl and 50 μL of Nafion solution (5 wt%). The above mixture was ultrasonicated for at least 30 min to obtain a homogeneous catalyst ink. Subsequently, 200 μL of the ink was uniformly coated onto the carbon paper (SGL Sigracet 28BC, 2 × 2 cm^2^ in effective area, with a catalyst loading of 400 μg cm^−2^) and dried under ambient conditions (25 °C) to fabricate the GDEs.

### H_2_O_2_ electrosynthesis in GDEs

H_2_O_2_ electrosynthesis experiments were conducted in a custom-made undivided electrochemical reactor, as illustrated in Supplementary Fig. 77. To avoid confounding effects from interactions among the complex components present in real water matrices, a 100 mL Na_2_SO_4_ (0.05 M) solution without or with Ca^2+^ served as the supporting electrolyte, ensuring that any observed impacts could be attributed solely to Ca^2+^. The electrolytes used for H_2_O_2_ electrosynthesis were stored at room temperature (25 °C) after preparation and used within one week. Unless otherwise specified, the pH of all as-prepared electrolytes was adjusted to 7.0 ± 0.1 using H_2_SO_4_ or NaOH. The solution was continuously stirred at 600 rpm to ensure sufficient mass transfer. A Pt sheet (1 × 1 cm^2^) was used as the anode, and a catalyst-loaded GDE served as the cathode, with its gas diffusion layer facing the air phase. The distance between the two electrodes was maintained at 1.5 cm. Throughout the experiment, no external aeration was applied, as oxygen was solely supplied via the GDE. A direct current power source (HSPY-300-03) provided constant current for H_2_O_2_ electroproduction. The reaction was conducted over 3 h, with 1 mL of solution sampled every 30 min for subsequent H_2_O_2_ concentration measurement. After the reaction, an additional 5 mL of solution was collected and filtered through a 0.22 μm membrane for Ca^2+^ analysis. The fundamental modification from 2D planar to 3D fenced cathode architecture involved only the incorporation of a PUS (20ppi, 30ppi, 40ppi, 60ppi; 20 × 20 × 5 mm) positioned in direct contact with the cathode, preserving identical experimental conditions otherwise. The 3D PUS fenced cathode configuration was illustrated in Fig. 5a and Supplementary Fig. 78.

### H_2_O_2_ decomposition in GDEs

To investigate the influence of different cathodes on the electroreduction of H_2_O_2_, decomposition experiments were conducted in a dual-chamber electrochemical cell separated by a cation exchange membrane (CEM, 6 × 6 cm, with a thickness of 400 μm) (Supplementary Fig. 79). Prior to use, the CEM was immersed in Na_2_SO_4_ (0.05 M) solution for 24 h. Given the susceptibility of H_2_O_2_ to decomposition, the electrolytes employed in H_2_O_2_ decomposition experiments were freshly prepared before use. The anodic chamber was filled with 50 mL of Na_2_SO_4_ (0.05 M) solution as the supporting electrolyte, while the cathodic chamber contained 100 mL of a mixed solution consisting of Na_2_SO_4_ (0.05 M) and H_2_O_2_ (1200 mg L^−1^). Throughout the reaction, the electrolyte was continuously stirred at 600 rpm to ensure homogeneity. A Pt sheet (1 × 1 cm^2^) was employed as the anode, and the GDEs with varying degrees of scaling were used as cathodes. The air-facing side of the GDE was sealed during the whole experiment, with the cathodic chamber continuously purged with N_2_ (100 mL min^−1^) to keep an O_2_-free environment. The decomposition experiment was conducted over 1 h, with 0.5 mL samples taken from the cathodic chamber at 10-min intervals for subsequent determination of the residual H_2_O_2_ concentration.

### Quantification of H_2_O_2_

The concentration of H_2_O_2_ was determined spectrophotometrically at *λ* = 400 nm using the potassium titanium oxalate method. Specifically, 1 mL of sample was added to a mixture containing 1 mL of potassium titanium oxalate solution (0.05 M) and 1 mL of H_2_SO_4_ (3 M). The resulting solution was diluted to 50 mL with deionized water, vigorously mixed, and allowed to stand for 10 min before measuring its absorbance. The H_2_O_2_ concentration of samples was calculated based on a calibration curve shown in Supplementary Fig. 80a. The Faradaic efficiency (FE) and H_2_O_2_ yield were determined using the following equations:

1 $${{{{\rm{H}}}}}_{2}{{{{\rm{O}}}}}_{2}{{{\rm{FE}}}}\left(\%\right)=\frac{2\times 96485\times C\times V}{{\int }_{0}^{t}{Idt}}\times 100\%$$

2 $${{{{\rm{H}}}}}_{2}{{{{\rm{O}}}}}_{2}{{{\rm{Yield}}}}=\frac{C\times V}{{mt}}$$

Where *C* represents the H_2_O_2_ concentration (mol L^−1^), *V* represents the electrolyte volume (*L*), ${\int }_{0}^{t}{Idt}$ represents the total passed charge (*C*), *m* represents the catalyst loading mass (*g*), and *t* represents the reaction time (*h*).

To exclude the potential interference from Ca^2+^ in the electrolyte on H_2_O_2_ quantification, standard H_2_O_2_ solutions containing different Ca^2+^ concentrations (0–400 mg L^−1^) were analyzed by UV-vis spectroscopy (Supplementary Fig. 80b). The results indicated no significant difference in absorbance peaks at *λ* = 400 nm, confirming that Ca^2+^ exert negligible interference on the spectrophotometric determination of H_2_O_2_.

### Electrochemical measurements

All electrochemical tests were conducted at ambient temperature (25 °C) using a standard three-electrode system with a multi-channel electrochemical workstation (VMP3 Bio-Logic, France). Unless otherwise specified, the prepared GDE (2 × 2 cm^2^) served as the working electrode, a Pt sheet (1 × 1 cm^2^) as the counter electrode, and an Ag/AgCl (KCl, saturated) electrode as the reference electrode. Calibration of the Ag/AgCl reference electrode was performed against a freshly purchased Ag/AgCl reference electrode. The two electrodes were placed in the same electrolyte, and the open-circuit potential difference between them was measured. All potentials reported in this study were converted to the potentials versus reversible hydrogen electrode (RHE) using the following Nernst equation:

3 $${E}_{{RHE}}={E}_{{Ag}/{AgCl}}+0.0591\times {{{\rm{pH}}}}+0.197$$

The double-layer capacitance (*C*_dl_) of the prepared electrodes was determined by cyclic voltammetry (CV) tests to evaluate the electrochemical active surface area (ECSA). CV measurements were performed in a single-chamber cell filled with N_2_-saturated Na_2_SO_4_ (0.05 M) solution, with a potential range of 0.8–1.0 V vs. RHE and at varying scan rates of 10, 20, 40, 60, 80, and 100 mV s^−1^. The capacitive current density (Δj) was calculated according to the following equation:

4 $$\triangle j=\frac{{j}_{{anode}}-{j}_{{cathode}}}{2}$$

Where *j*_anode_ and *j*_cathode_ denote the anodic and cathodic current densities at 0.90 V vs. RHE, respectively, extracted from each CV curve. The calculated Δ*j* values were plotted as a function of the scan rate and subjected to linear regression. The slope of the resulting linear fit was taken as the *C*_dl_ of the electrode. Because *C*_dl_ is generally proportional to the ECSA under identical measurement conditions, the relative differences in ECSA among the investigated cathodes were evaluated by comparing their *C*_dl_ values. For the ORR, linear sweep voltammetry (LSV) was conducted in an O_2_-saturated Na_2_SO_4_ (0.05 M) electrolyte at a scan rate of 5 mV s^−1^ over a potential range of 0.6 to −0.4 V vs RHE.

All electrochemical tests related to the cathodic reduction of H_2_O_2_ were carried out in an H-type cell separated by a CEM. To investigate the influence of Ca^2+^ on H_2_O_2_ reduction, a fresh GDE was used as the working electrode, with Na_2_SO_4_ (0.05 M) solution as the anolyte and N_2_-saturated 0.1 M NaCl, 0.2 M NaCl, or 0.1 M CaCl_2_ solution containing 10 mM H_2_O_2_ as the catholyte. To examine the effect of precipitated calcium scale on H_2_O_2_ reduction, GDEs with varying degrees of scaling were employed as working electrodes, with 0.05 M Na_2_SO_4_ as the anolyte and N_2_-saturated Na_2_SO_4_ (0.05 M) containing 10 mM H_2_O_2_ as the catholyte. LSV curves were collected from 0.7 to 0 V vs. RHE at a scan rate of 5 mV s^−1^. Electrochemical impedance spectroscopy (EIS) was performed at the potential ranging from 0.3 to 0.45 V vs. RHE, with a frequency range of 0.1 Hz to 100 kHz and an amplitude of 5 mV.

The potential of zero charge (PZC) for C0 and C18 was determined using the differential capacitance method in a single-chamber cell filled with N_2_-saturated Na_2_SO_4_ (0.1 M) solution. EIS measurements were performed at a frequency of 1 Hz with a sinusoidal voltage perturbation amplitude of 5 mV. The potential range was set to 0.1–0.7 V vs RHE within potential steps of 50 mV. The PZC was identified at the potential corresponding to the minimum value of the differential capacitance (*C*_d_), which was calculated using the following formula:

5 $${C}_{d}=\left|\frac{1}{\omega Z{\prime\prime} }\right|$$

Where ω represents the angular frequency (Hz) and $Z{\prime\prime}$ represents the imaginary part of the impedance spectra (Ω).

The adsorption of OH^−^ (OH_ad_) was determined using CV methods. The CV curves were collected in a single-compartment cell containing 0.1 M NaOH solution (pH = 13.0 ± 0.1) saturated with N_2_. A Pt sheet and an Hg/HgO electrode were employed as the counter electrode and reference electrode, respectively. The measurements were conducted over a potential range of −1.0 to −0.1 V vs Hg/HgO with a scan rate of 50 mV s^−1^. The potentials were converted to the RHE reference scale using the following formula:

6 $${E}_{{RHE}}={E}_{{Hg}/{HgO}}+0.0591\times {{{\rm{pH}}}}+0.0983$$

### RRDE measurement and current density deconvolution for ORR

The selectivity of 2e^−^ ORR was evaluated using a rotating ring-disk electrode (RRDE) setup (AFMSRCE, Pine, Physicochemical Co. Ltd.), consisting of a glassy carbon disk electrode (diameter: 5.61 mm, area: 0.2475 cm^2^) and a platinum ring electrode (inner diameter: 6.25 mm, outer diameter: 7.92 mm, area: 0.1866 cm^2^). 10 μL of the catalyst ink was drop-cast onto the disk electrode and allowed to dry naturally, serving as the working electrode. An Ag/AgCl electrode and a platinum wire were used as the reference and counter electrodes, respectively. Prior to electrochemical measurements, the catalyst was electrochemically activated by performing cyclic voltammetry (CV) scans at 50 mV s^−1^ until a stable voltammogram was obtained. Linear sweep voltammetry (LSV) was conducted in O_2_‑saturated 0.1 M NaCl or 0.1 M CaCl_2_ solutions at a scan rate of 10 mV s^−1^, with the RRDE rotating at 1600 rpm. The platinum ring electrode was held at 1.2 V vs RHE to detect the generated H_2_O_2_. The H_2_O_2_ selectivity and the electron transfer number (*n*) were calculated using the following equations:

7 $${{{{\rm{H}}}}}_{2}{{{{\rm{O}}}}}_{2}{{{\rm{selectivity}}}}\left(\%\right)=\frac{{I}_{R}/N}{{I}_{D}+{I}_{R}/N}\times 200$$

8 $$n=\frac{{I}_{D}}{{I}_{D}+{I}_{R}/N}\times 4$$

Where *I*_D_ and *I*_R_ represent the disk current and ring current, respectively, and *N* is the collection efficiency of the ring electrode (*N* = 0.37).

The ring-disk current densities can be further deconvoluted into two sequential reaction current densities: those corresponding to the reduction of oxygen-to-peroxide (O2P), and those corresponding to the subsequent reduction of peroxide-to-hydroxide (P2H)^23^. The method describes a stepwise oxygen reduction reaction (ORR) pathway, which is consistent with the current reaction occurring on the carbon electrode. The current densities for the O2P and P2H steps were then calculated using the following formulas:

9 $${j}_{O2P}={j}_{D}\times \left(\frac{\sigma N+1}{2\sigma N}\right)$$

10 $${j}_{P2H}={j}_{D}\times \left(\frac{\sigma N-1}{2\sigma N}\right)$$

Where j_D_ is the disk current density, σ is the disk-to-ring current density ratio.

### In-situ ATR-SEIRAS measurements

In-situ electrochemical ATR-SEIRAS measurements were performed using a Fourier transform infrared spectrometer (Nicolet iS50, Thermo Fisher Scientific) equipped with a liquid-nitrogen-cooled mercury cadmium telluride (MCT) detector. A Si ATR prism chemically coated with a thin Au film was used simultaneously as the optical waveguide and the conductive working-electrode substrate, onto which the catalyst ink was uniformly deposited. A standard three-electrode configuration was employed for all tests, comprising the catalyst-modified Au/Si ATR electrode as the working electrode, an Ag/AgCl electrode as the reference electrode, and a Pt wire as the counter electrode. All electrochemical measurements were conducted in a dual-compartment H-cell reactor. The cathodic compartment was filled with O_2_-saturated 0.1 M NaCl or 0.1 M CaCl_2_ as the working electrolyte, while the anodic compartment contained 0.1 M Na_2_SO_4_. The ATR-SEIRAS spectra were collected over a potential range from 0.6 to −0.4 V vs RHE. The spectrum collected at the open-circuit potential was used as the background.

### Measurement of electric-field-induced Ca^2+^ adsorption

The Ca^2+^ adsorbed on the electrode surface under an applied electric field was quantified using an electrode transfer method^16,26^. Specifically, the electrode was operated at −0.3 V vs Ag/AgCl in 0.1 M CaCl_2_ solution for 120 s. Subsequently, the electrode was rapidly transferred from the electrolyte into 30 mL of ultrapure water while maintaining constant potential. The applied potential was then disconnected to release the adsorbed Ca^2+^ into the pure water, and the Ca^2+^ concentration was quantified by ICP-OES.

### Characterizations

The microstructures of the surfactant-modified OCB samples were observed by transmission electron microscope (TEM, FEI Tecnai G2 F30, USA) and high-resolution TEM (HRTEM). The morphologies of as-prepared cathodes with varying degrees of scaling were obtained by scanning electron microscope (SEM, ZEISS Sigma500, Germany) equipped with an energy dispersive spectrum (EDS, Oxford X-max, UK) system. A 3D optical profilometer (S neox, Sensofar, Spain) was used to detect the surface topology of the cathodes. Raman spectrometer (HORIBA HR Evolution, France) was employed to analyze the defection structure of synthesized catalysts. The functional groups of as-prepared samples were identified by Fourier transformation infrared spectroscopy (FTIR, PerkinElmer Frontier, USA). X-ray photoelectron spectroscopy (XPS, Thermo Scientific K-Alpha+, USA) was applied to investigate the surface chemical valance states of the synthesized catalysts. X-ray diffraction (XRD, Bruker D8 Advance, Germany) was employed to obtain the crystal information of the cathodes. The thermal stability of obtained samples was detected by using a thermogravimetric analysis instrument (TGA, Netzsch STA 449 F5 Jupiter, Germany). Zeta potentials measurements were conducted with Anton Paar Litesizer500. The wettability of all fabricated cathodes was detected by the contact angle (CA) instrument (Kino SL150, USA). Ultraviolet-visible spectra (UV-vis) were collected with Shimadzu UV-2550. Inductively coupled plasma optical emission spectrometry (ICP-OES, PerkinElmer Avio 550 Max, USA) was employed to determine the concentration of Ca^2+^ in the electrolyte before and after the electrochemical reaction.

### Density functional theory (DFT) calculations

All first-principles DFT calculations in this work were carried out using the Vienna Ab initio Simulation Package (VASP)^54,55^. The electron-ion interactions were described by the projector-augmented wave (PAW) method^56,57^, while the valence electronic states were expanded in a plane-wave basis set with a cutoff energy of 500 eV. The exchange-correlation effects were treated within the generalized gradient approximation (GGA) via the Perdew-Burke-Ernzerhof (PBE) functional^58^. Geometry optimization was considered converged when the forces on all atoms fell below 0.02 eV Å^−1^, and the energy difference between successive self-consistent field iterations was less than 10^−5^ eV. A vacuum layer of 20 Å was introduced along the z-direction to screen the effect of neighboring layers. Monkhorst-Pack’s sampling scheme was adopted for Brillouin-zone sampling with a k-point grid of 2π × 0.04 Å^−1^ ^59^. The DFT-D3 with Beck-Johnson (BJ) damping method was employed to account for the long-range van der Waals interaction^60^. The energy barriers of water dissociation process were calculated through the climbing image nudged elastic band (CI-NEB) method^61^, in which all of force components perpendicular to the tangent of the reaction path were relaxed to be less than 0.03 eV Å^−1^. A water bi-layer was included to describe explicitly the polarization involved in water dissociation. The implicit solvation effects were implemented using the VASPsol package.

The electric field strength at the interface between the graphene surface and the electrolyte was evaluated using a parallel-plate capacitor model^16^. The electric field strength (*E*) was calculated according to the following equation:

11 $$E=\frac{{Q}_{1}+{Q}_{2}}{2\varepsilon S}$$

Where *Q*_1_ and *Q*_2_ represent the net charges of the graphene slab and the electrolyte, respectively, as determined by Bader charge analysis; *S* represents the surface area of the graphene slab; and *ε* represents the absolute permittivity of water.

The adsorption energies of *OOH or Ca^2+^ on the surface of C0 or C18 were calculated by the following formula:

12 $${E}_{{ad}}={E}_{{total}}-{E}_{{base}}-{E}_{{adsorbate}}$$

Where *E*_total_ is the total energy of the absorbed system, *E*_base_ is the basement energy and *E*_adsorbate_ is the energy of adsorbate.

### Molecular dynamics (MD) simulation

To investigate the distribution of H_2_O and Ca^2+^ at the electrode-electrolyte interface, MD simulations were performed using GROMACS (Version 2020.06). Periodic graphene electrodes (52.60 Å × 52.08 Å in xoy plane), with or without STAB modification, were positioned on both sides of the simulation box, with a spacing of 100 Å along the z-direction. The electrolyte, composed of 20 Na_2_SO_4_, 8 CaSO_4_, and 8938 H_2_O molecules, was placed between the two graphene electrodes. A vacuum layer of 50 Å was introduced along the z-direction to minimize electrodes interactions. All MD simulations in this work employed the General Amber Force Field (GAFF), with atomic charges assigned using the Restrained Electrostatic Potential (RESP) method. An external electric field of 0.1 V nm^−1^ was physically applied perpendicular to the electrode surface (along the z-direction). Prior to the formal simulation, an energy minimization procedure was conducted. All MD simulations were carried out under the NVT ensemble for 20 ns. The Berendsen thermostat method for temperature coupling (*T* = 298 K) was applied with a relaxation constant of 0.5 ps. A time step of 2 fs was used, and system trajectories were collected at 1 ps intervals. Long-range electrostatic interactions were treated using the Particle Mesh Ewald (PME) method, while van der Waals interactions were computed with a cutoff scheme, both using a cutoff distance of 1.2 nm. Radial distribution functions for H_2_O and Ca^2+^ were analyzed using data from the final 5 ns of the simulations. Snapshots of the electrode-electrolyte interface were also presented.

## Supplementary information

Supplementary Information

Description of Additional Supplementary Files

Supplementary Data 1

Transparent Peer Review file

## Source data

Source Data

## Data Availability

All data supporting the findings of this study are available within the article and the Supplementary Information file. Source data are provided with this paper.
