# Supplementary material for: Calcium-tolerant electrode strategy for efficient hydrogen peroxide electrosynthesis in natural water
Source: Nat Commun. 2026 Aug 31;17:10358. doi: 10.1038/s41467-026-77022-0 (PMC13623961; doi:10.1038/s41467-026-77022-0)
Supplement: Supplementary file 1 [file 41467_2026_77022_MOESM1_ESM.pdf]

Supplementary Information

Calcium-tolerant electrode strategy for efficient hydrogen peroxide electrosynthesis in natural water

Ke Wang[1,2], Ying Zhu[1,2], Yuan Yao[1], Ke Wang[1,2,3]*

[1]State Key Laboratory of Urban-rural Water Resource and Environment, Harbin Institute of Technology, Harbin 150090, China

[2]School of Environment, Harbin Institute of Technology, Harbin 150090, China

[3]National Engineering Research Center for Safe Sludge Disposal and Resource Recovery, Harbin Institute of Technology, Harbin 150090, China

[*]Corresponding author:

Ke Wang, State Key Laboratory of Urban-rural Water Resource and Environment, Harbin Institute of Technology, Harbin 150090, China.

E-mail address: hitwk@sina.com

ORCID: https://orcid.org/0009-0005-4955-2193

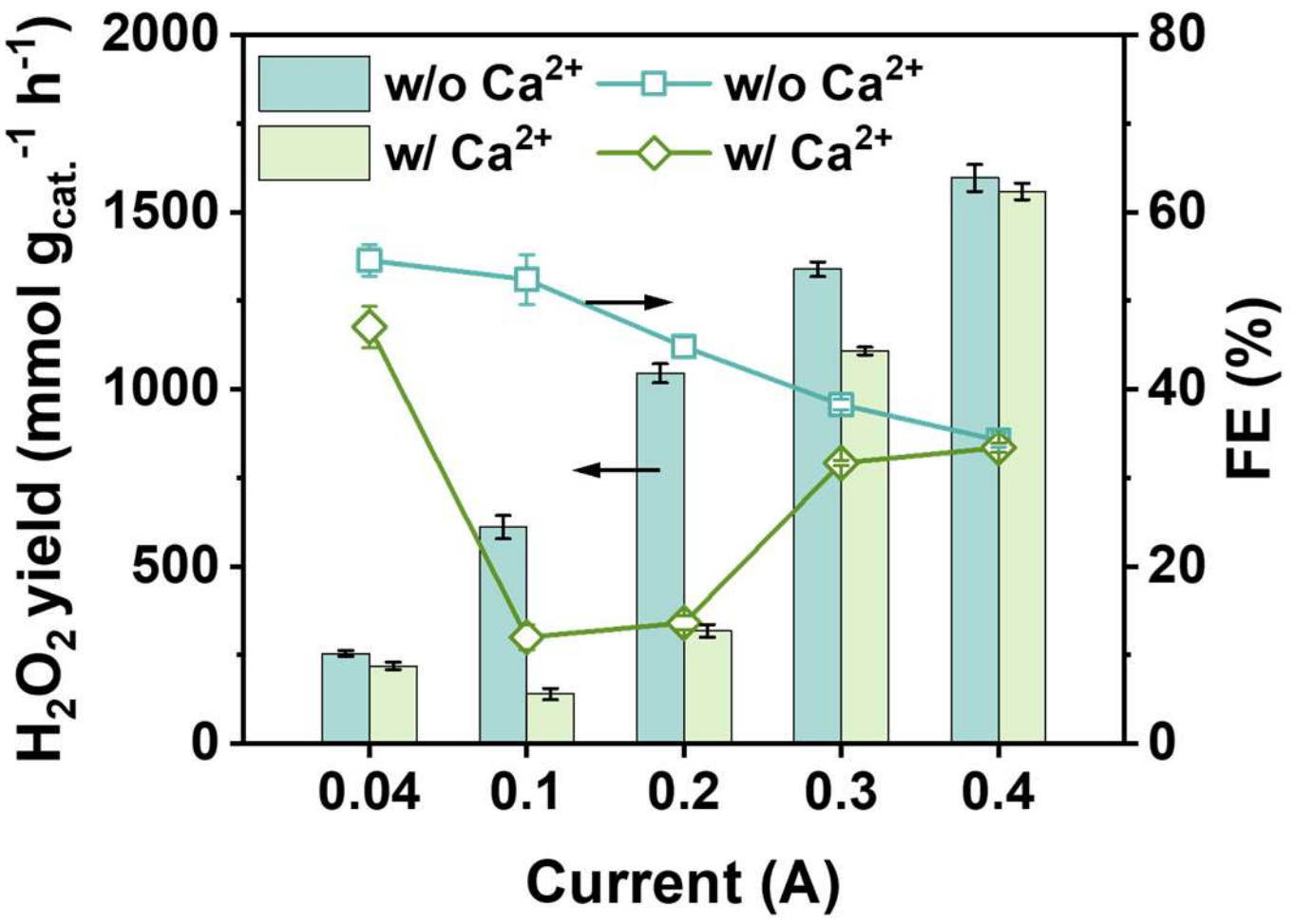

Supplementary Fig. 1. Effects of current densities on H_2O_2 electrosynthesis performance for C0 in the absence (w/o) and presence (w/) of 200 mg L^{-1} Ca^{2+}. Reaction conditions: [Na_2SO_4] = 0.05 M, a rotation speed of 600 rpm, an initial pH of 7.0 ± 0.1 and electrolysis of 3 h. The data is presented as mean ± s.d. (n = 3). Source data for Supplementary Fig. 1 are provided as a Source Data file.

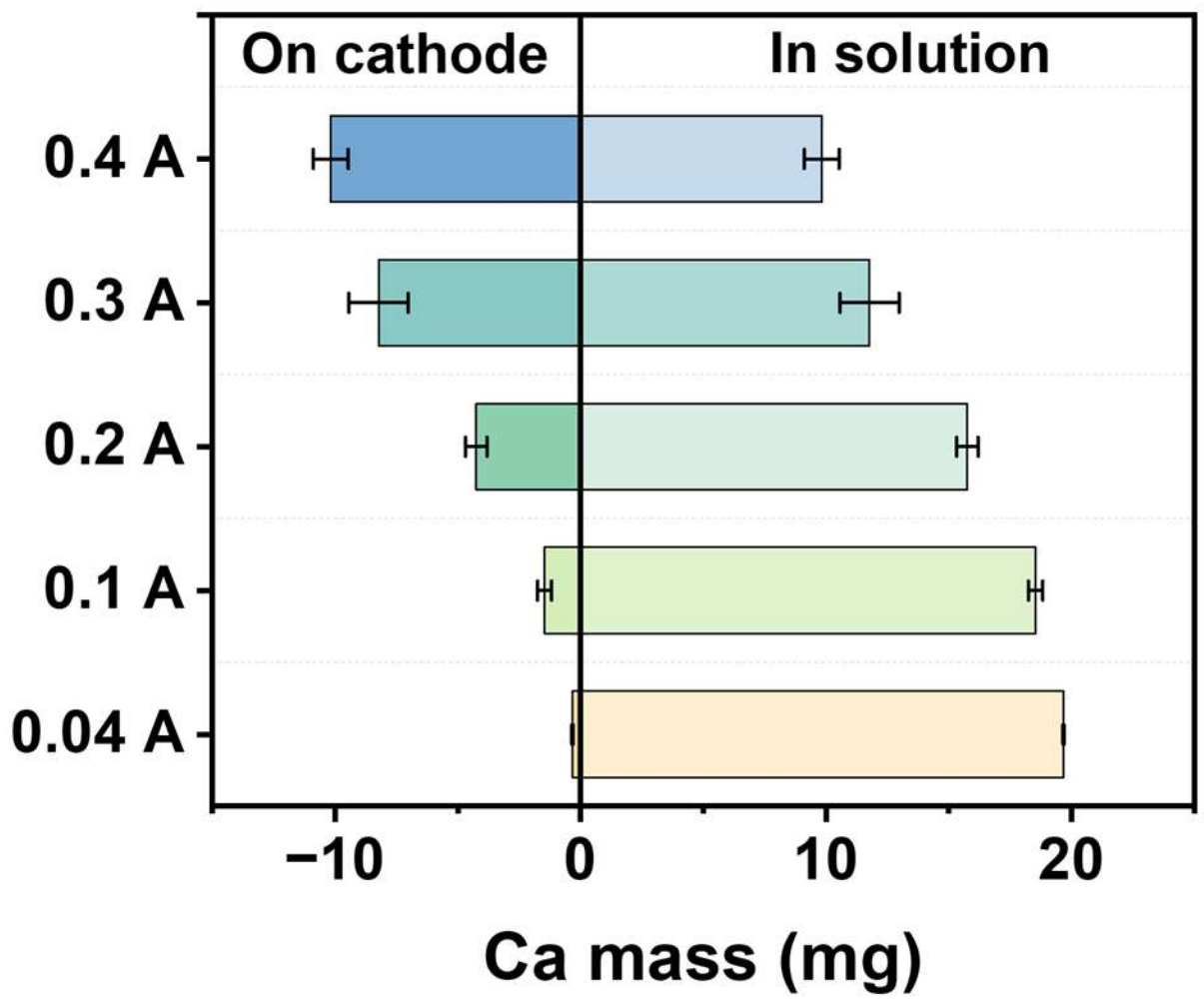

Supplementary Fig. 2. Distributions of two calcium species under different current densities for C0 cathode. Reaction conditions: [Na_2SO_4] = 0.05 M, [Ca^{2+}]$_0$ = 200 mg L^{-1}, a rotation speed of 600 rpm, an initial pH of 7.0 ± 0.1 and electrolysis of 3 h. The data is presented as mean ± s.d. (n = 3). Source data for Supplementary Fig. 2 are provided as a Source Data file.

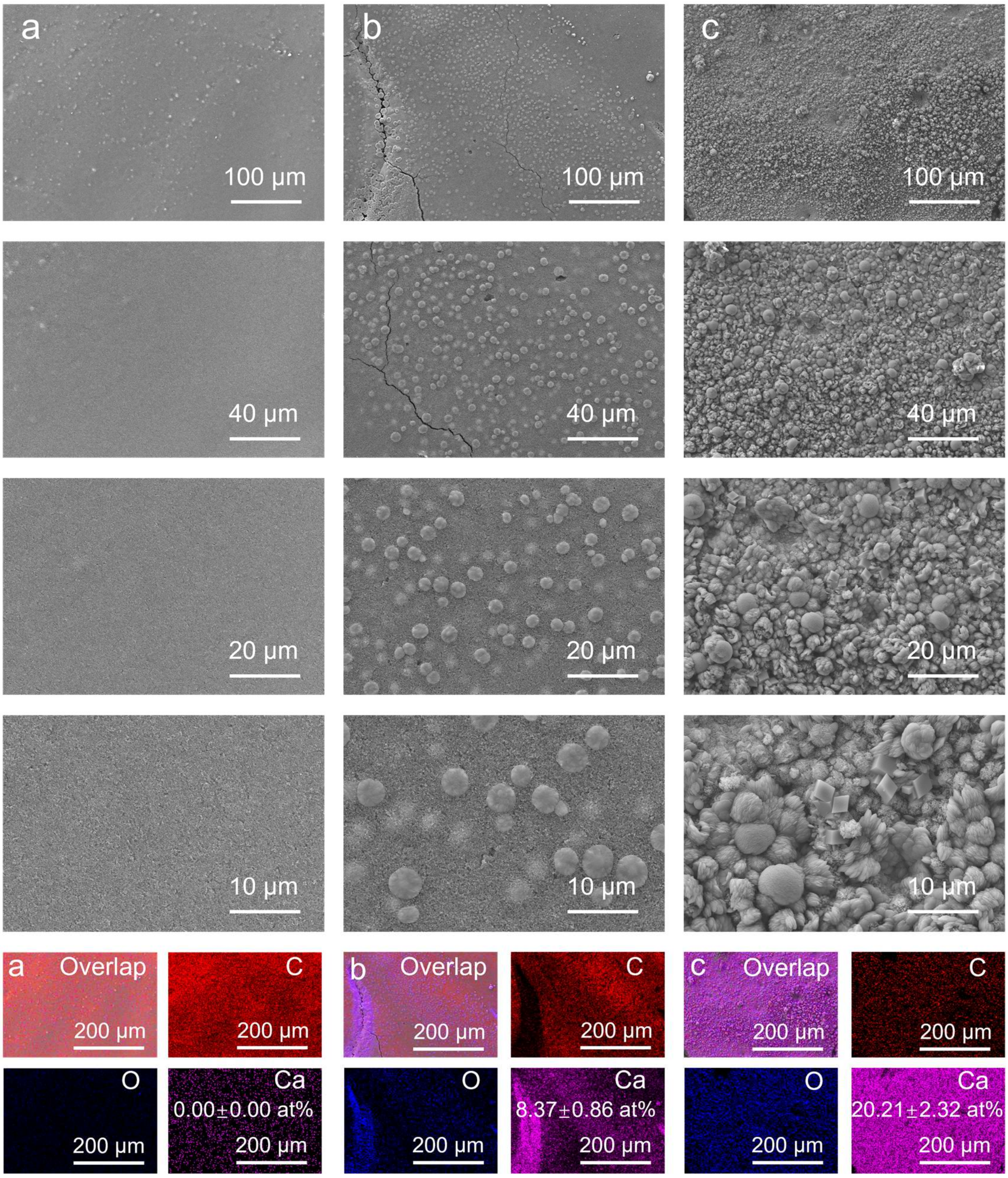

Supplementary Fig. 3. SEM images and EDS maps of the C0 cathode (a) before experiment, (b) after experiment at 0.1 A and (c) after experiment at 0.4 A. Reaction conditions: $[Na_2SO_4]$ = 0.05 M, $[Ca^{2+}]_0$ = 200 mg L^{-1}, a rotation speed of 600 rpm, an initial pH of 7.0 ± 0.1 and electrolysis of 3 h.

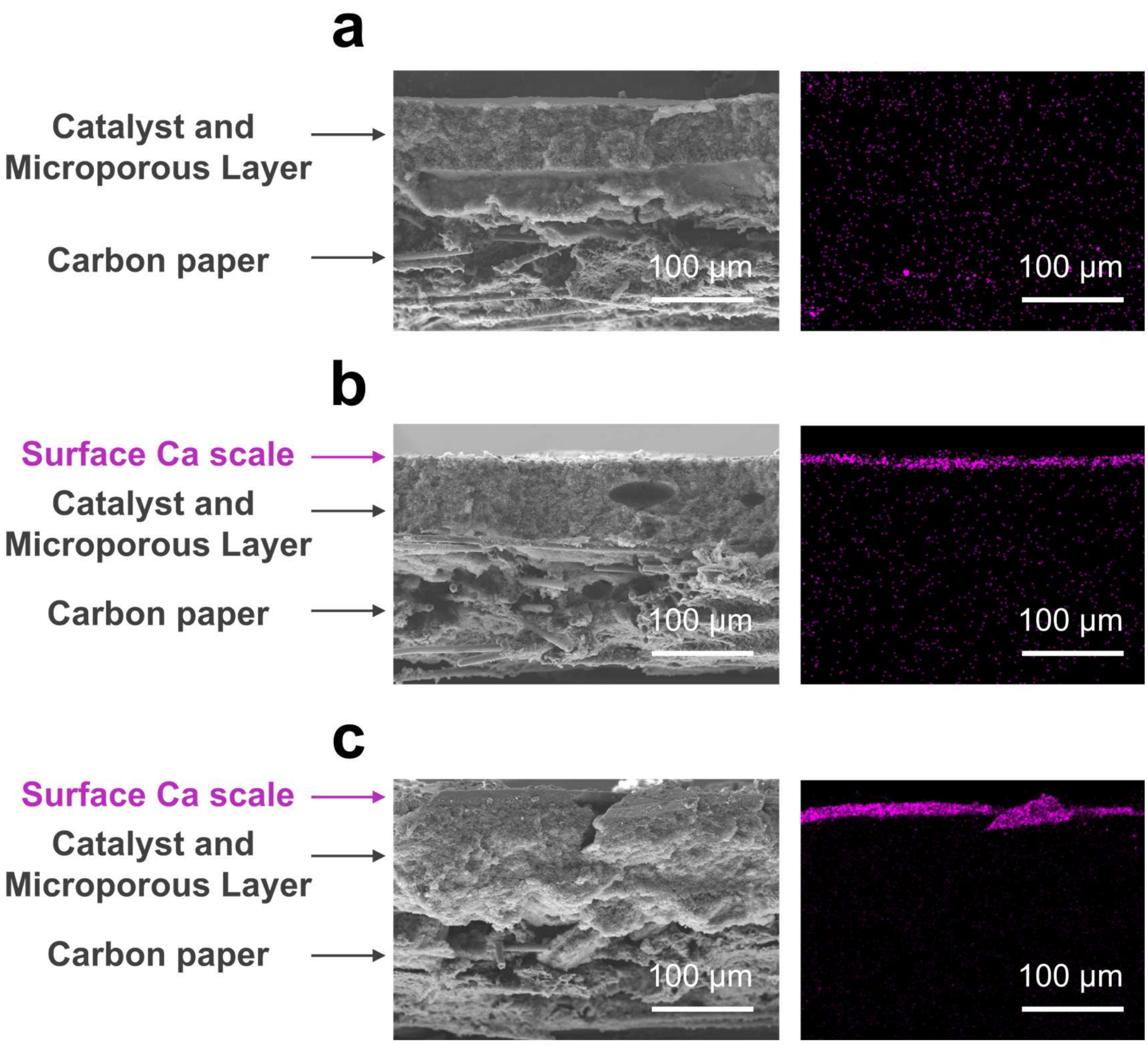

Supplementary Fig. 4. SEM images of cross-sectional view and EDS maps of Ca element for the C0 cathode (a) before experiment, (b) after experiment at 0.1 A and (c) after experiment at 0.4 A. Reaction conditions: [Na_2SO_4] = 0.05 M, [Ca^{2+}]$_0$ = 200 mg L^{-1}, a rotation speed of 600 rpm, an initial pH of 7.0 ± 0.1 and electrolysis of 3 h.

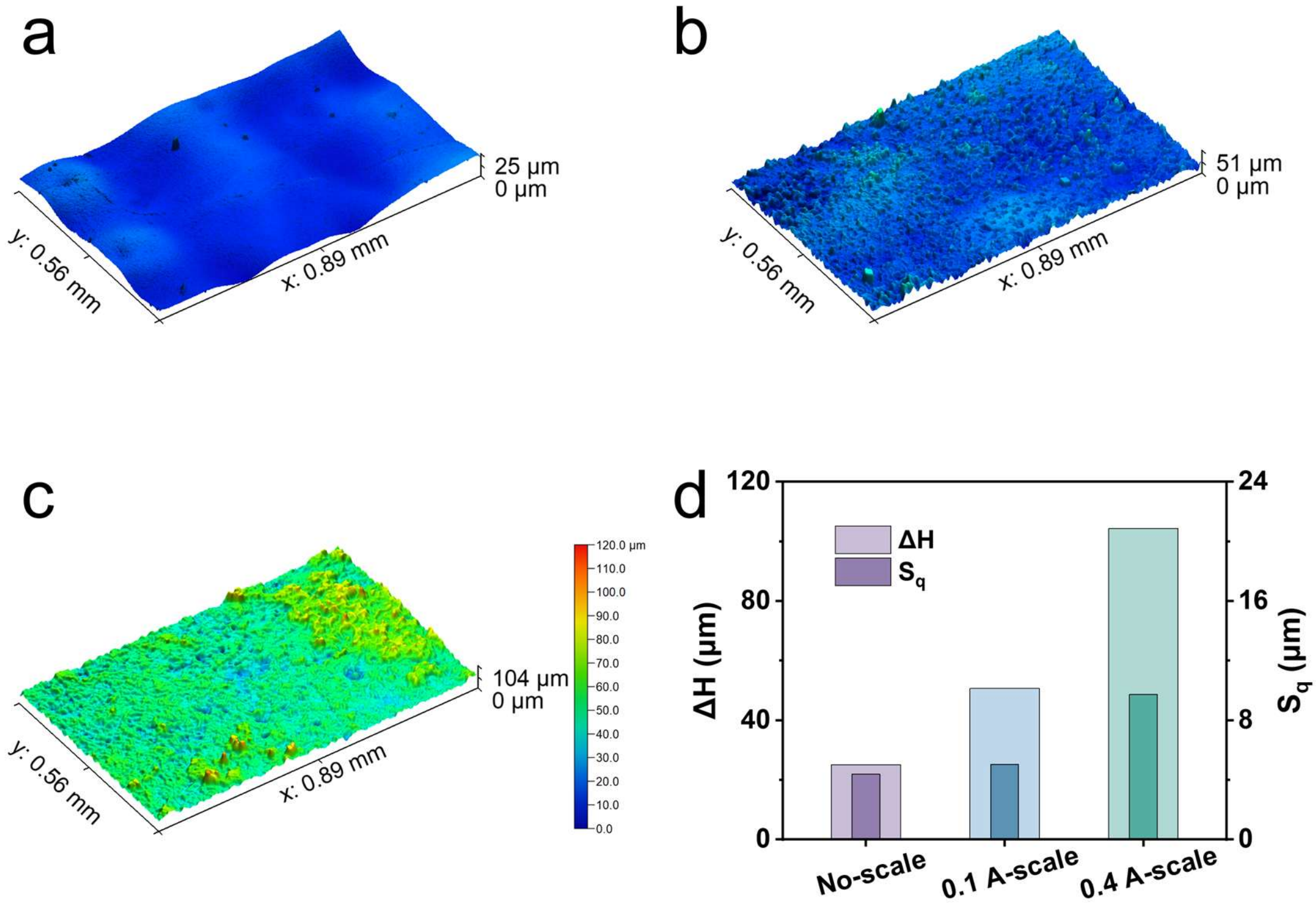

Supplementary Fig. 5. 3D surface profilometry of C0 (a) before experiments and after experiments at (b) 0.1 A and (c) 0.4 A in the presence of 200 mg L^{-1} Ca^{2+}. Variations in the height range (ΔH) and RMS roughness (S_q) of C0 cathodes with varying degrees of scaling. Reaction conditions: [Na_2SO_4] = 0.05 M, [Ca^{2+}]$_0$ = 200 mg L^{-1}, a rotation speed of 600 rpm, an initial pH of 7.0 ± 0.1 and electrolysis of 3 h. Source data for Supplementary Fig. 5d are provided as a Source Data file.

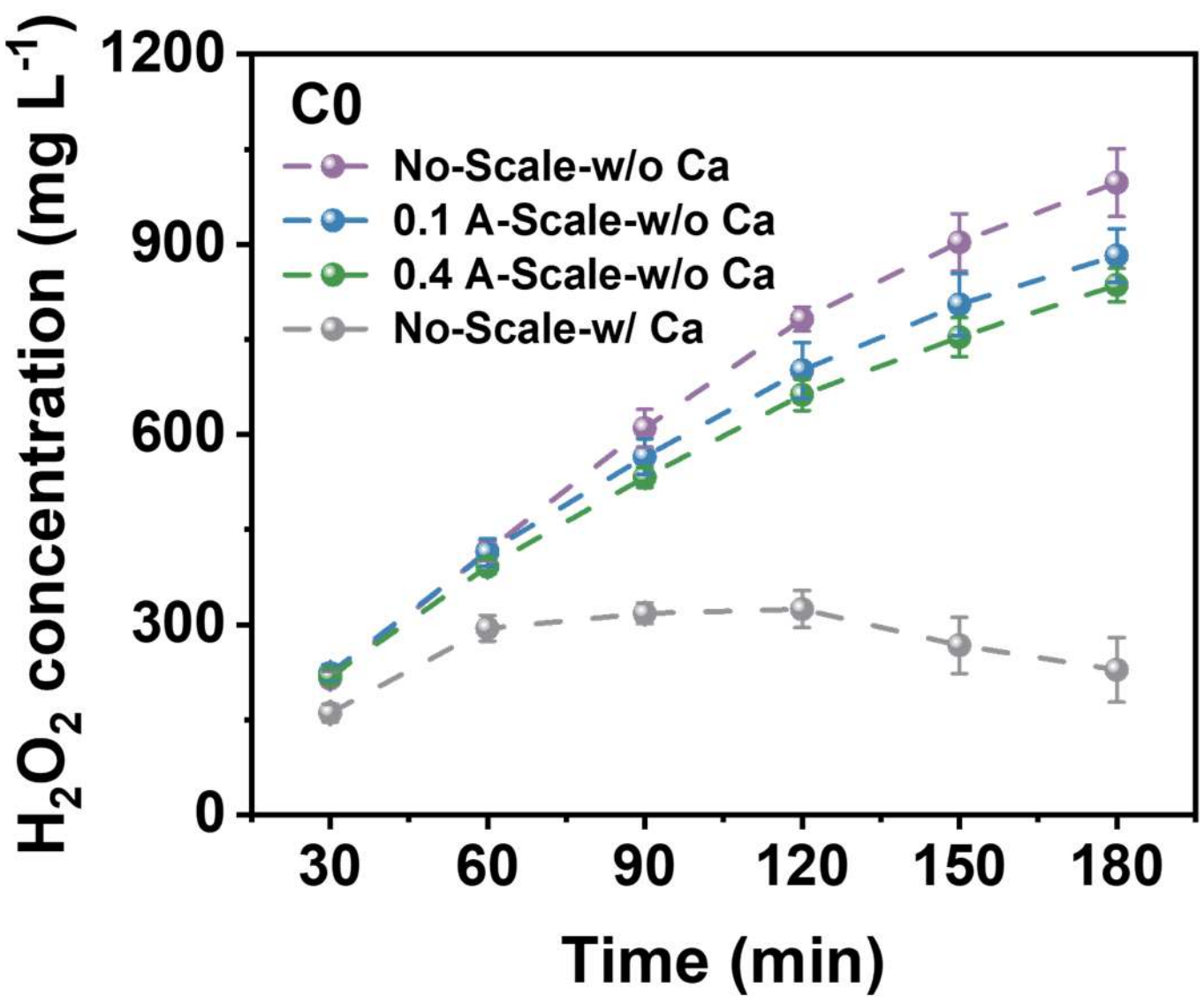

Supplementary Fig. 6. Time-dependent curves depicting H_2O_2 concentration of the C0 cathodes with varying degrees of scaling in 0.05 M Na_2SO_4 and no-scale cathode in 0.05 M Na_2SO_4 and 200 mg L^{-1} Ca^{2+}. Reaction conditions: a current of 0.1 A, a rotation speed of 600 rpm, electrolysis time of 3 h, and an initial pH of 7.0 ± 0.1. The data is presented as mean ± s.d. (n = 3). Source data for Supplementary Fig. 6 are provided as a Source Data file.

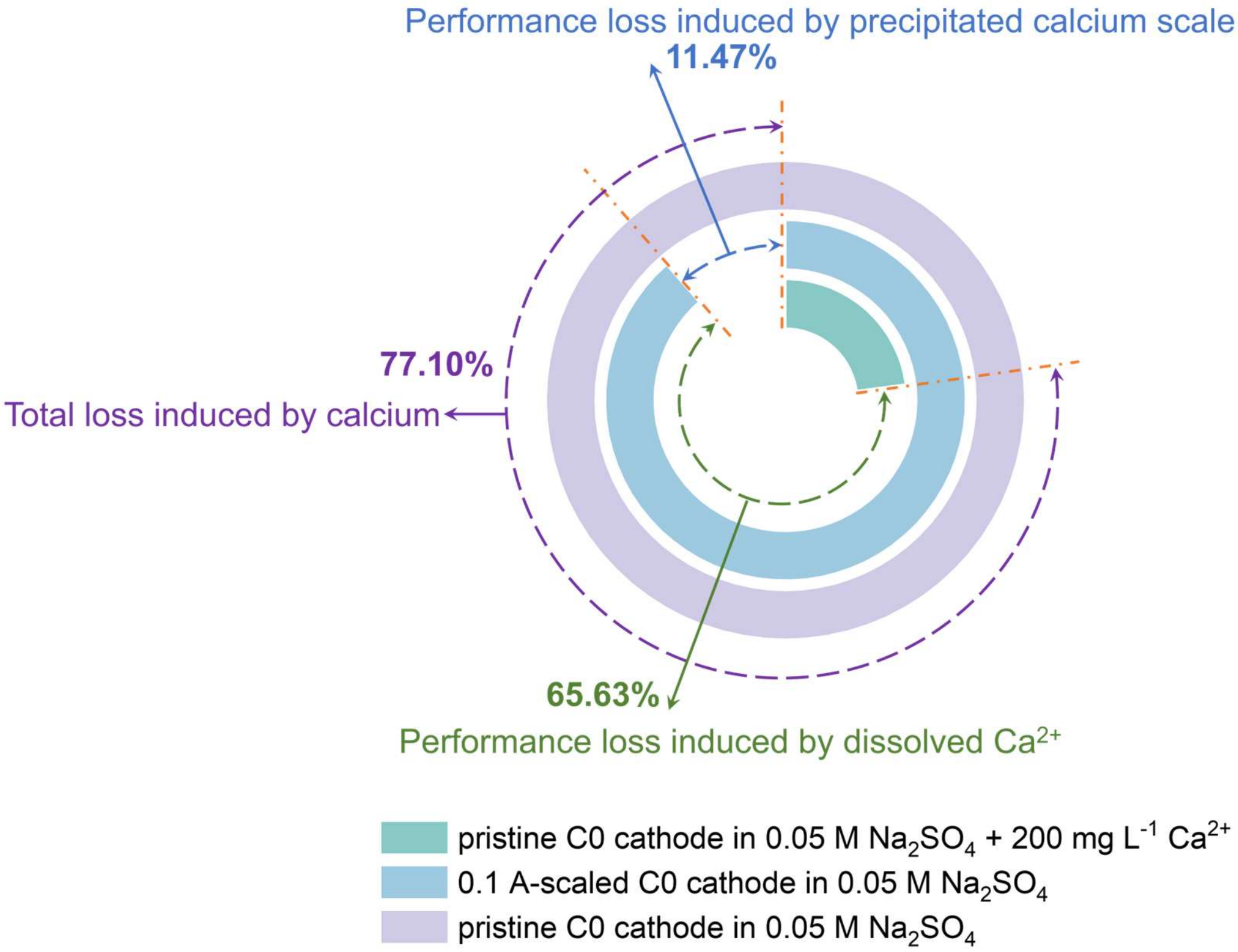

Supplementary Fig. 7. Decoupling analysis of the inhibitory effects of dissolved Ca^{2+} and precipitated calcium scale on H_2O_2 electrosynthesis performance for C0. Source data for Supplementary Fig. 7 are provided as a Source Data file.

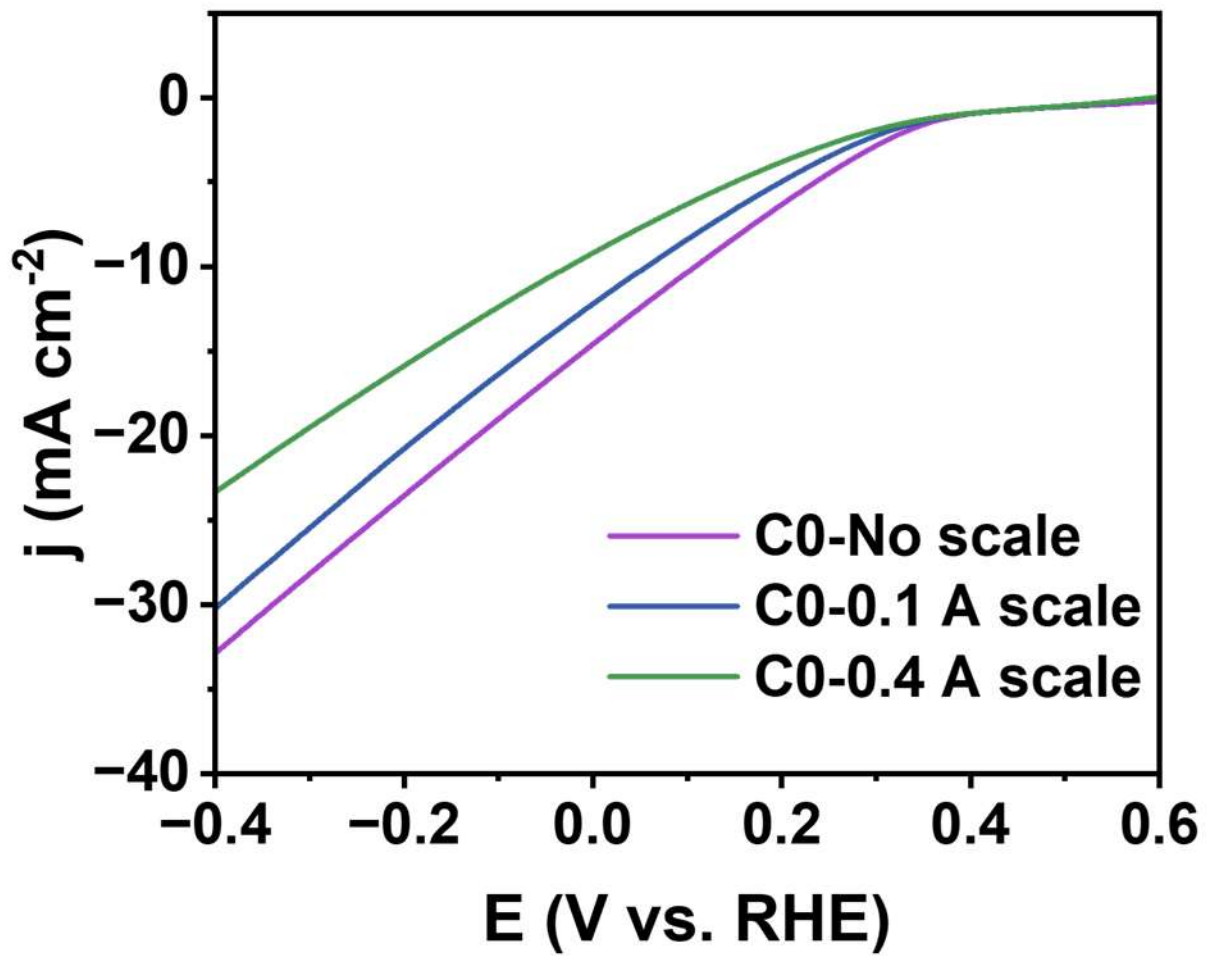

Supplementary Fig. 8. LSV curves of the C0 cathodes with varying degrees of scaling in O_2-saturated 0.1 M Na_2SO_4 electrolyte. All the potentials have not been iR-corrected. Source data for Supplementary Fig. 8 are provided as a Source Data file.

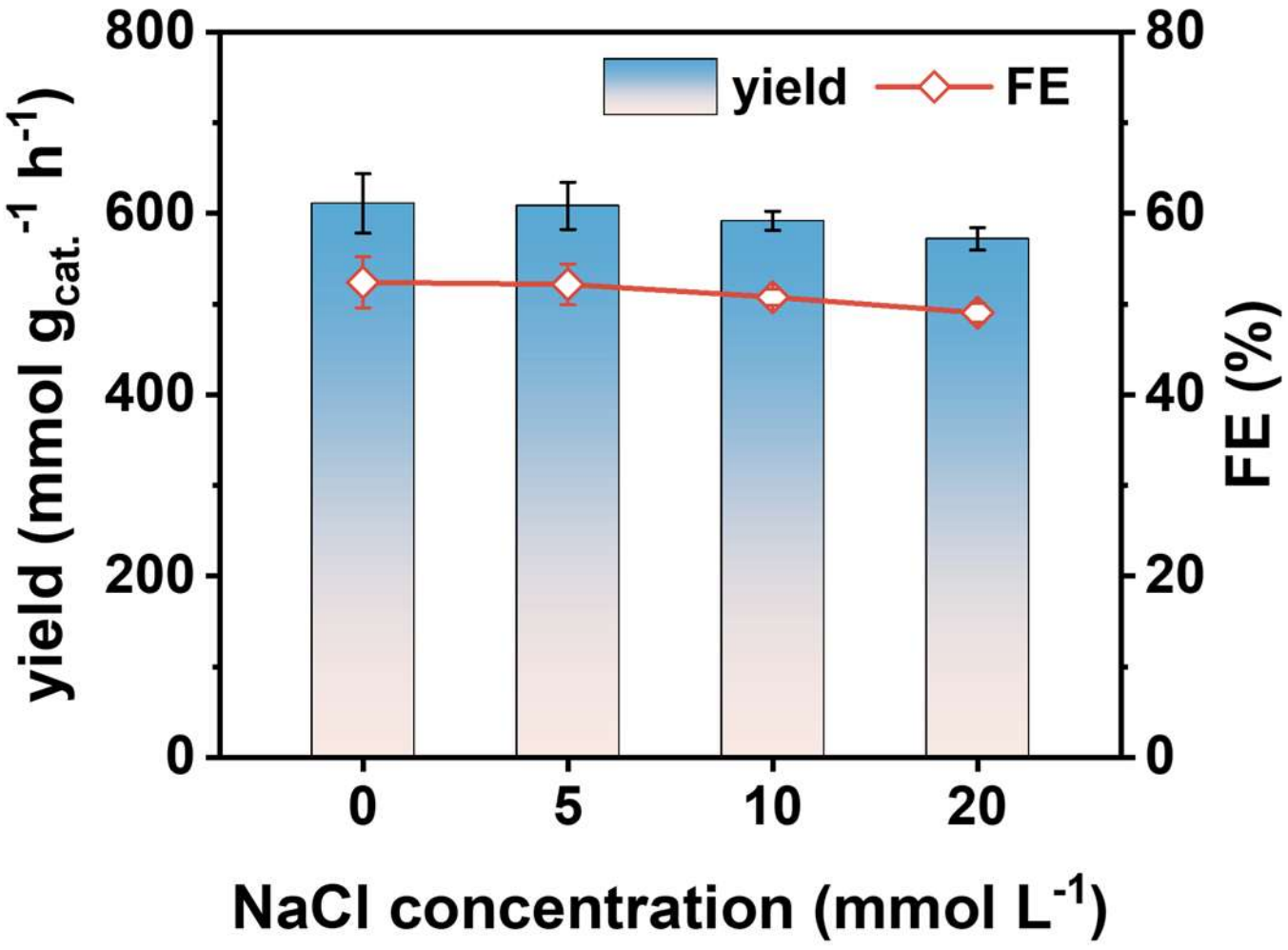

Supplementary Fig. 9. The effects of varying addition of NaCl on the cathodic H_2O_2 production performance. Reaction conditions: [Na_2SO_4] = 0.05 M, a current of 0.1 A, a rotation speed of 600 rpm, electrolysis time of 3 h, and an initial pH of 7.0 ± 0.1. The data is presented as mean ± s.d. (n = 3). Source data for Supplementary Fig. 9 are provided as a Source Data file.

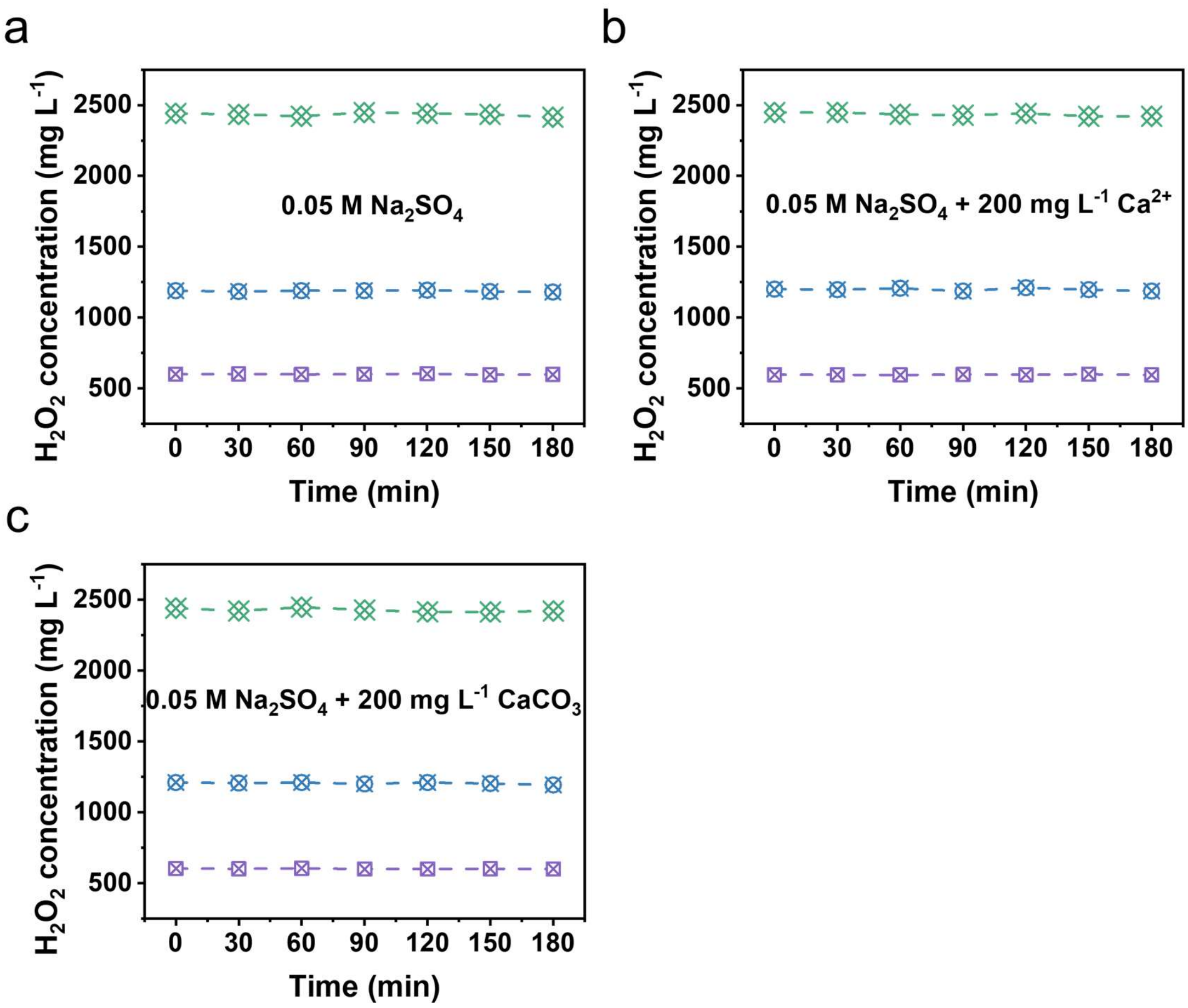

Supplementary Fig. 10. The self-decomposition curves of H_2O_2 in (a) 0.05 M Na_2SO_4, (b) 0.05 M Na_2SO_4 + 200 mg L^{-1} Ca^{2+} and (c) 0.05 M Na_2SO_4 + 200 mg L^{-1} $CaCO_3$ solutions under varying initial H_2O_2 concentrations. Reaction conditions: a current of 0 A, a rotation speed of 600 rpm, reaction time of 3 h, and an initial pH of 7.0 ± 0.1. Source data for Supplementary Fig. 10 are provided as a Source Data file.

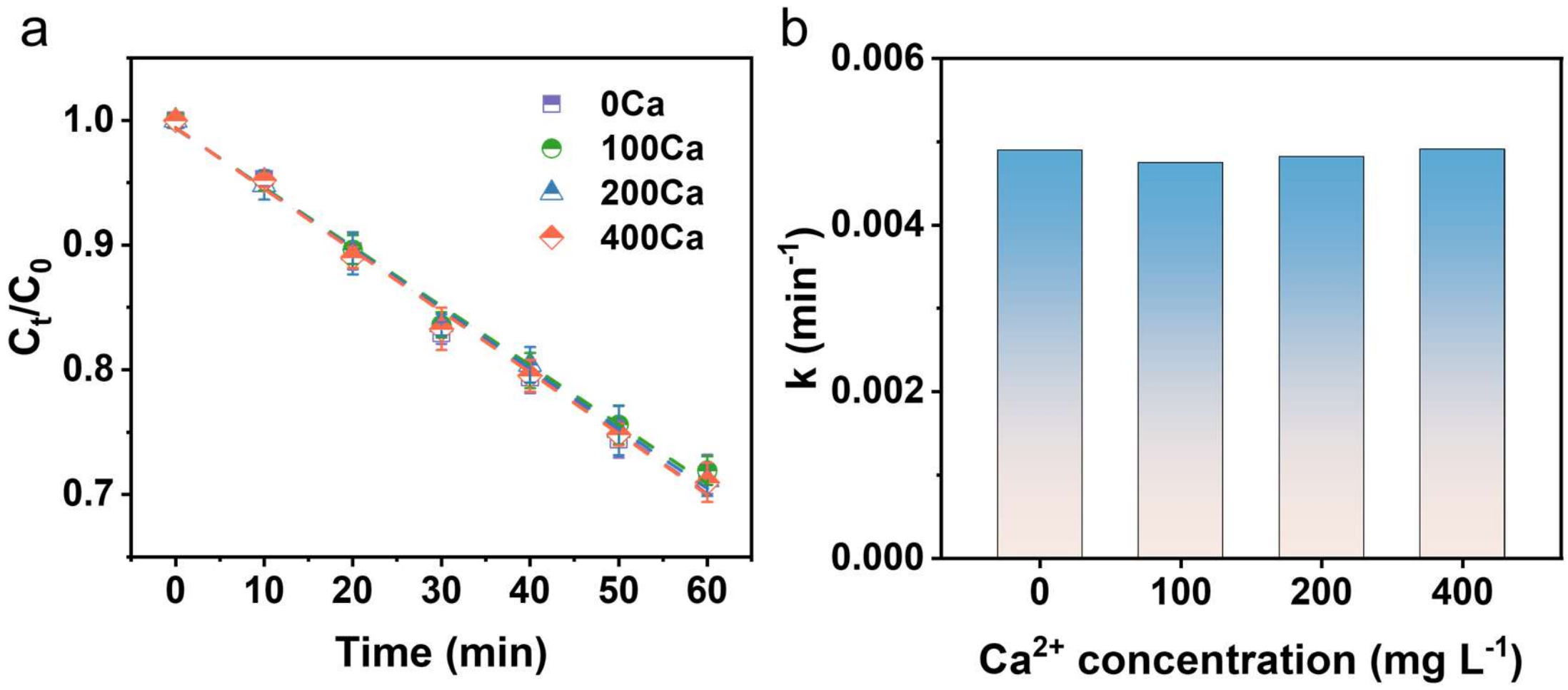

Supplementary Fig. 11. (a) H_2O_2 decomposition curves and (b) decomposition rate constant k of the Pt anode at an initial H_2O_2 concentration of 1200 mg L^{-1} with varying concentrations of Ca^{2+}. Reaction conditions: a current of 0.1 A, a rotation speed of 600 rpm, an initial pH of 7.0 ± 0.1, and electrolysis of 1 h. The data is presented as mean ± s.d. (n = 3). Source data for Supplementary Fig. 11 are provided as a Source Data file.

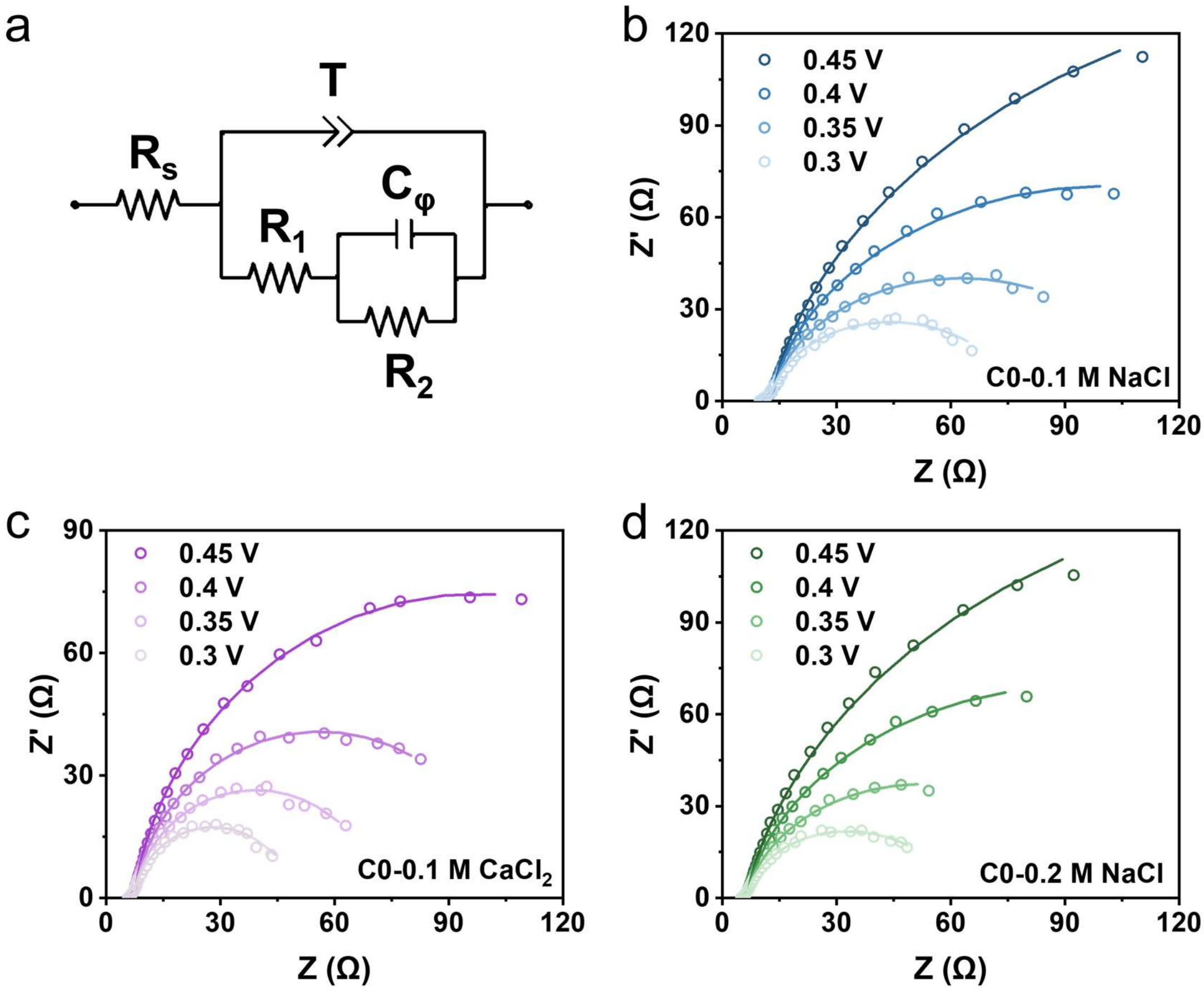

Supplementary Fig. 12. (a) The equivalent circuit. R_s is the solution resistance. T represents the double layer capacitance. R_1 is the charge transfer resistance, associated with the main electrochemical reaction. R_2 and C_φ represent the hydrogen adsorption resistance and pseudo-capacitance, respectively. The EIS Nyquist plots of the C0 cathode measured at different potentials in N_2-saturated (b) 0.1 M NaCl, (c) 0.1 M $CaCl_2$ and (d) 0.2 M NaCl electrolytes at an initial H_2O_2 concentration of 10 mM. Solid lines are the fitting results according to the equivalent circuit. All the potentials have not been iR-corrected. Source data for Supplementary Fig. 12 are provided as a Source Data file.

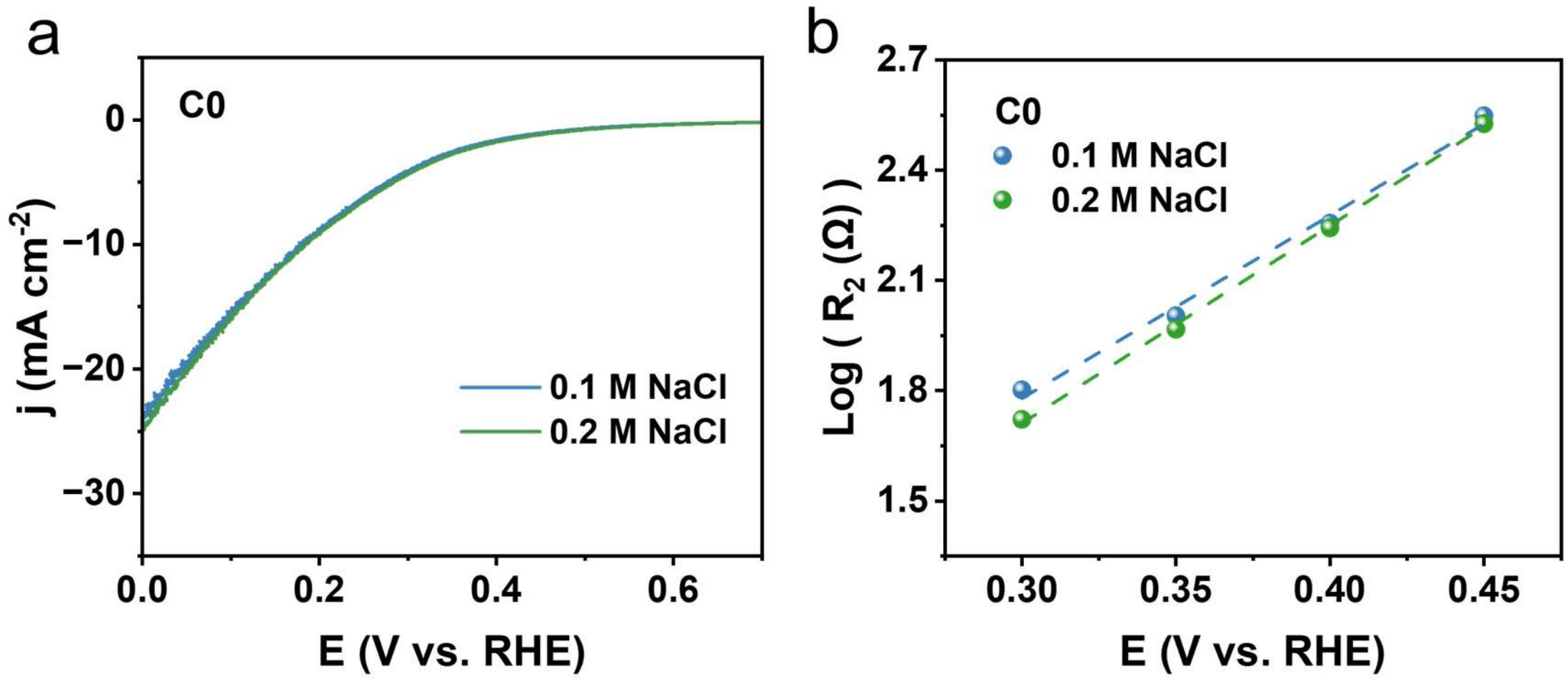

Supplementary Fig. 13. (a) LSV curves and (b) EIS-derived Tafel plots obtained from the hydrogen adsorption resistance R_2 of the C0 cathode in N_2-saturated 0.1 M NaCl or 0.2 M NaCl electrolytes containing 10 mM H_2O_2. All the potentials have not been iR-corrected. Source data for Supplementary Fig. 13 are provided as a Source Data file.

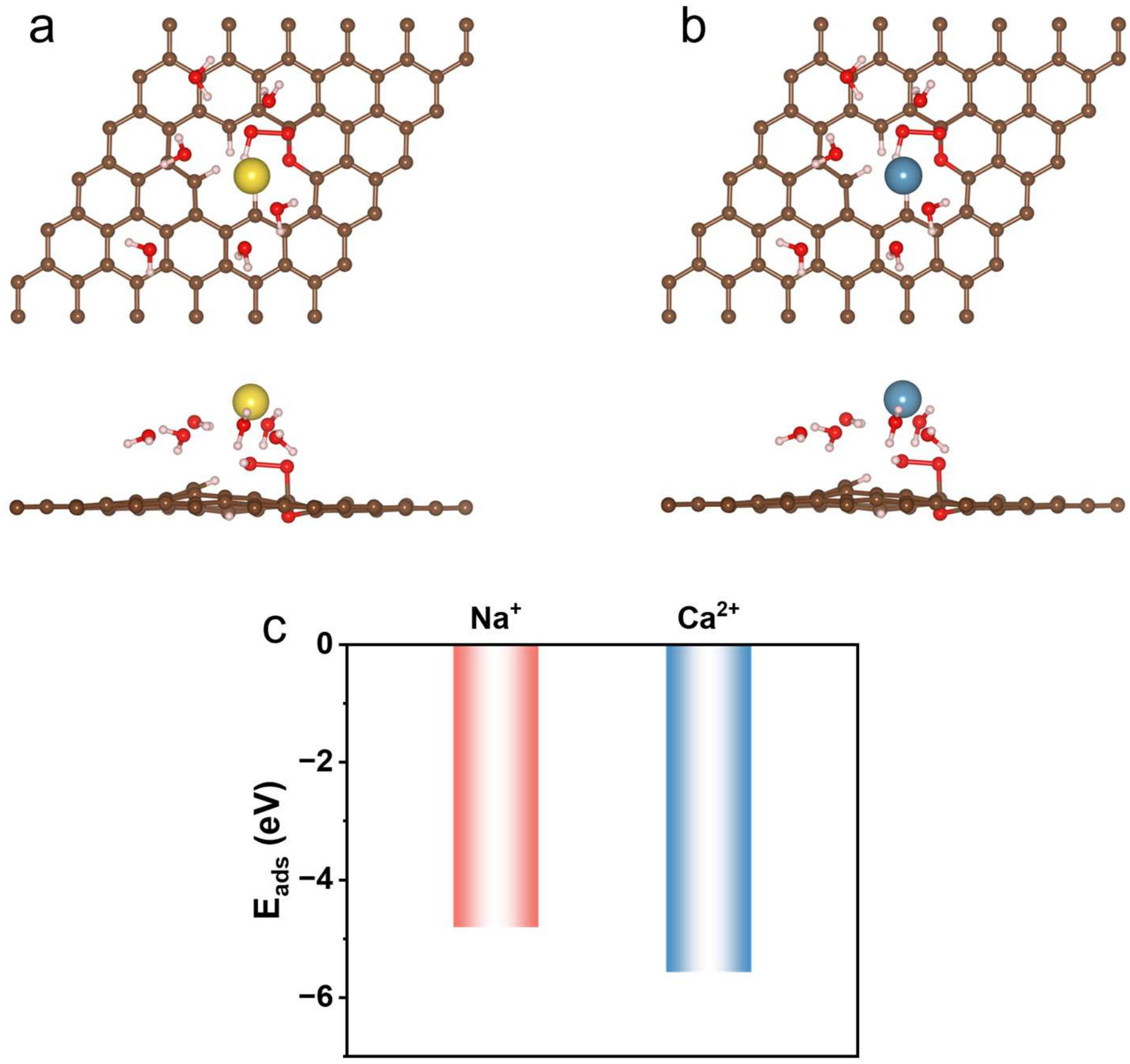

Supplementary Fig. 14. Atomic configurations of *OOH on the surface with (a) Na^+ and (b) Ca^{2+}. (c) The adsorption energies (E_{ads}) for *OOH on the graphene surfaces in Na^+ and Ca^{2+} systems. The brown, red, pink, yellow and blue spheres represent C, O, H, Na and Ca atoms, respectively. Source data for Supplementary Fig. 14 are provided as a Source Data file.

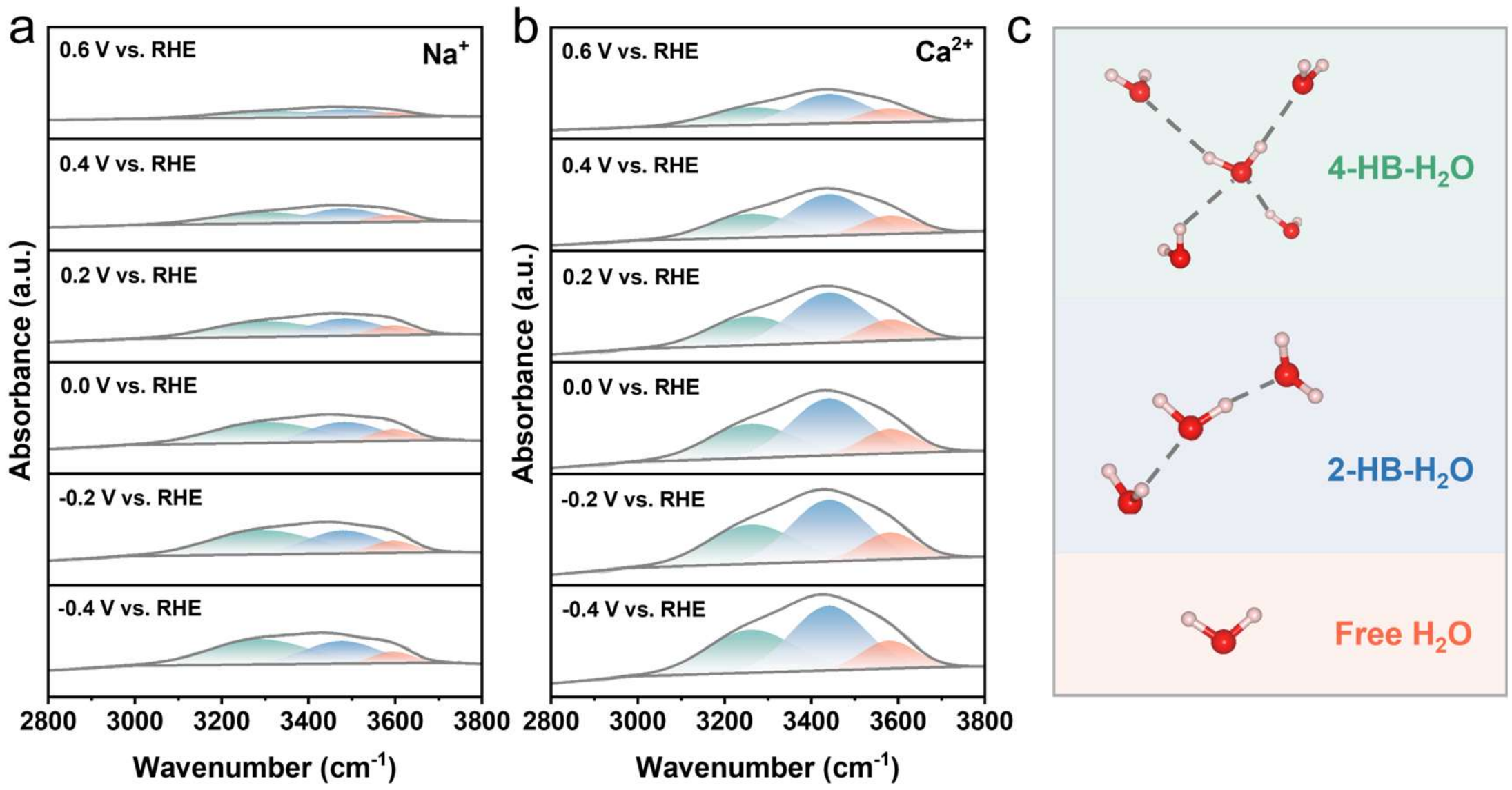

Supplementary Fig. 15. Deconvolution of O-H stretching band in ATR-SEIRAS spectra into 4-HB-H_2O, 2-HB-H_2O and free H_2O for C0 in (a) Na^+ and (b) Ca^{2+} systems. (c) Schematic illustration of the three interfacial water species. All the potentials have not been iR-corrected. Source data for Supplementary Fig. 15 are provided as a Source Data file.

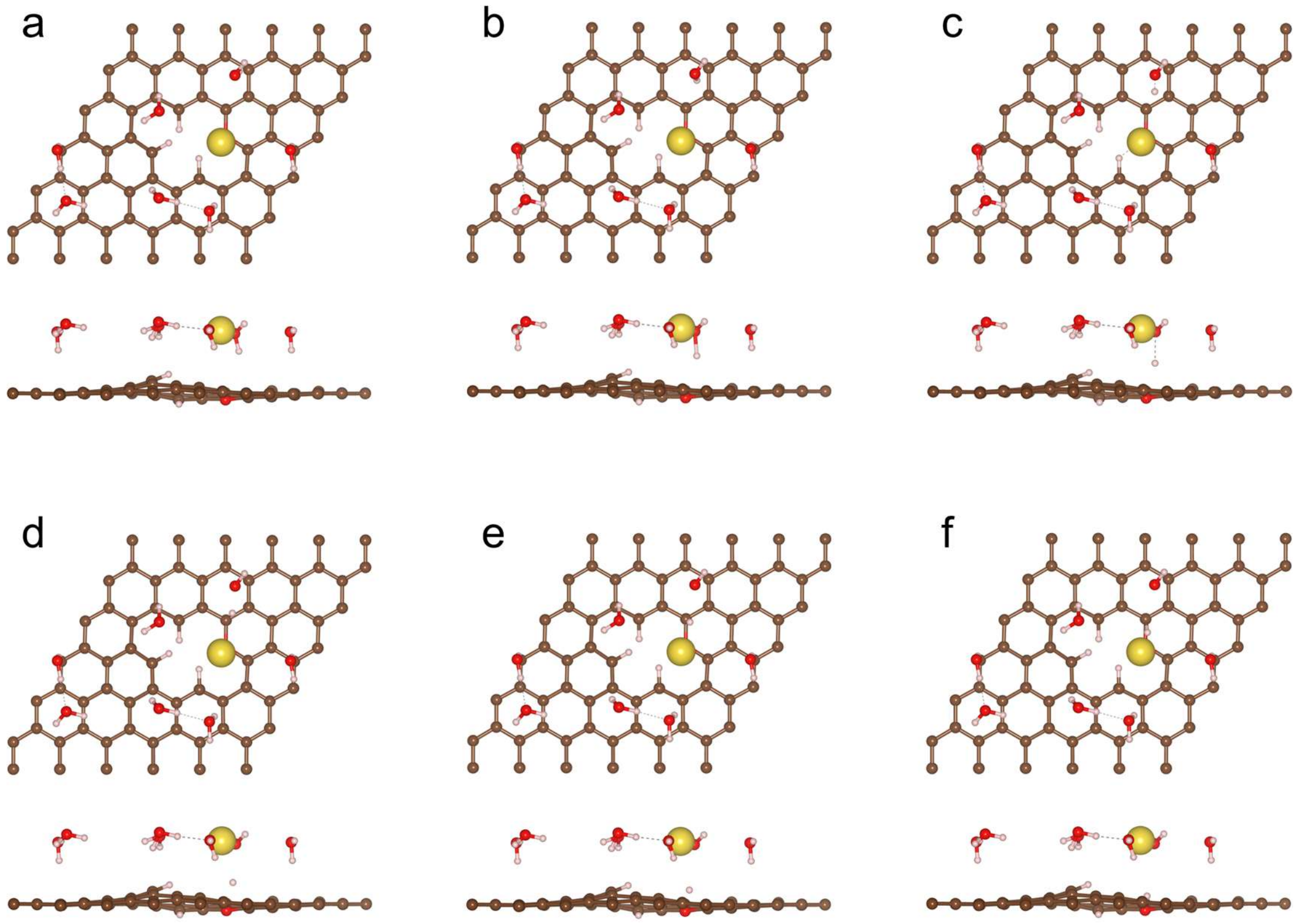

Supplementary Fig. 16. Atomic configurations of water dissociation step on the graphene surface with Na^+: (a) initial state, (b-e) transition state and (f) final state. The brown, red, pink, and yellow spheres represent C, O, H and Na atoms, respectively.

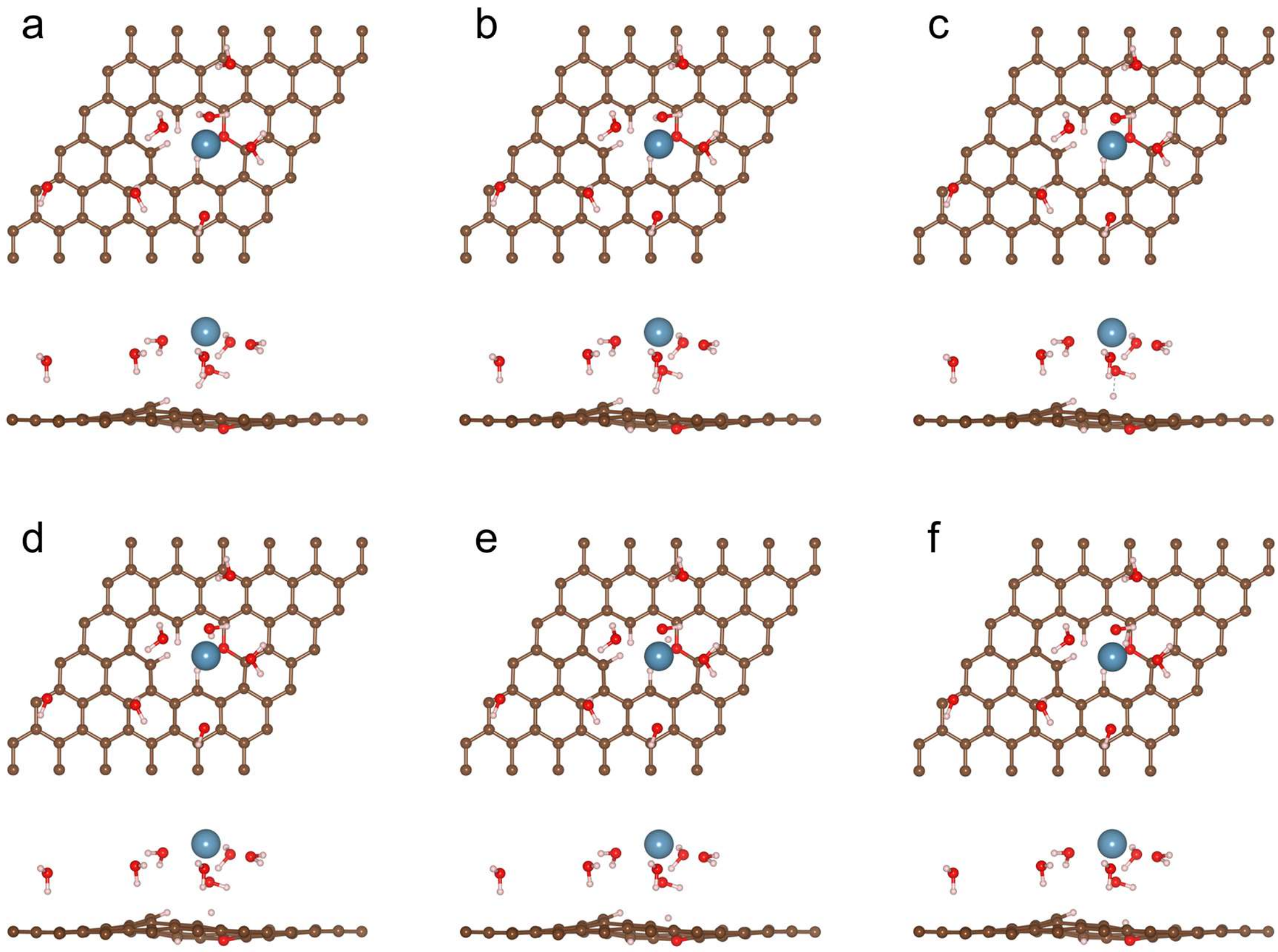

Supplementary Fig. 17. Atomic configurations of water dissociation step on the graphene surface with Ca^{+}: (a) initial state, (b-e) transition state and (f) final state. The brown, red, pink, and blue spheres represent C, O, H and Ca atoms, respectively.

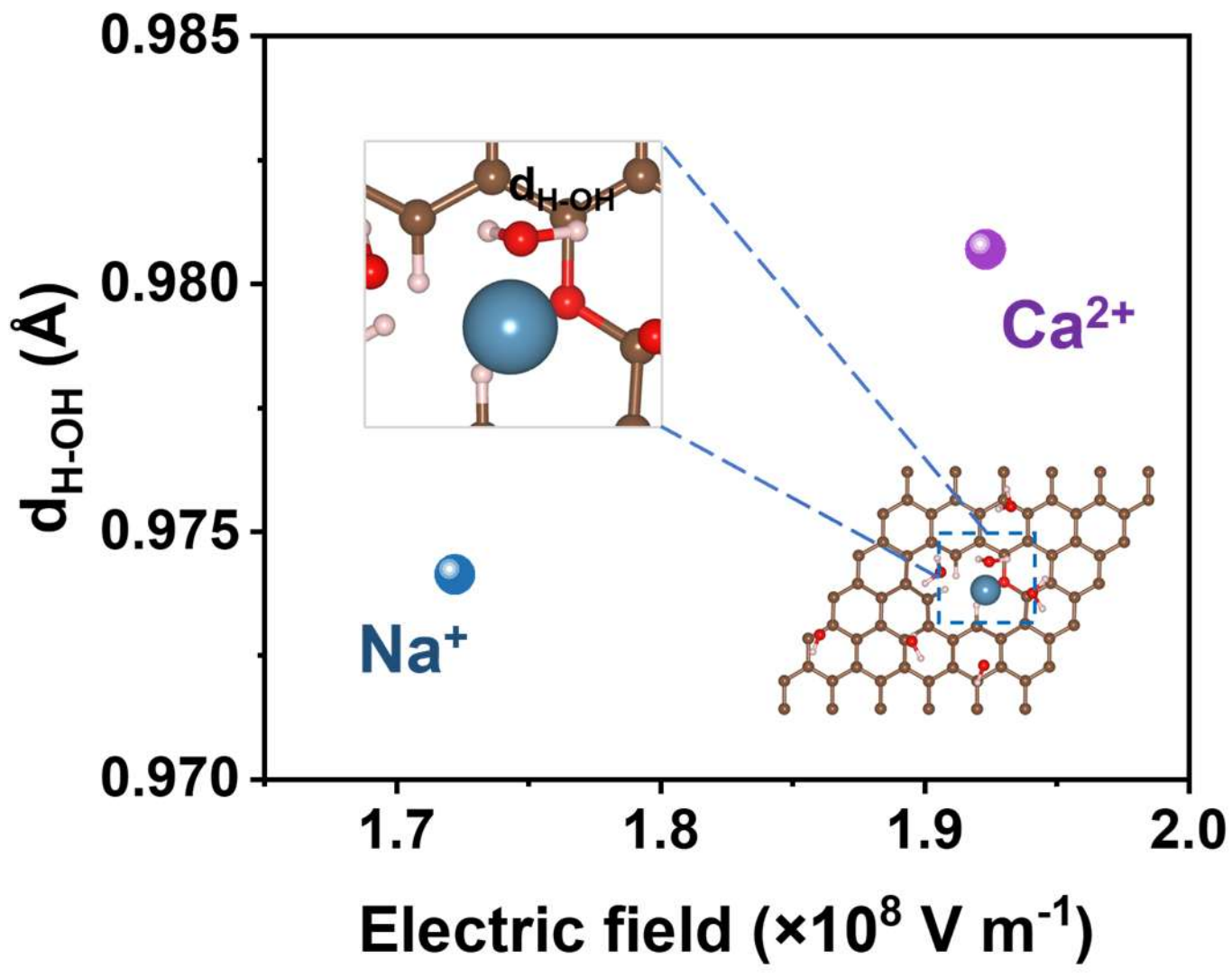

Supplementary Fig. 18. The measured bond length of H-OH bond in Na^+ and Ca^{2+} systems. The insets show molecular models of water dissociation step on the graphene surface with Ca^+. The brown, red, pink, and blue spheres represent C, O, H and Ca atoms, respectively. Source data for Supplementary Fig. 18 are provided as a Source Data file.

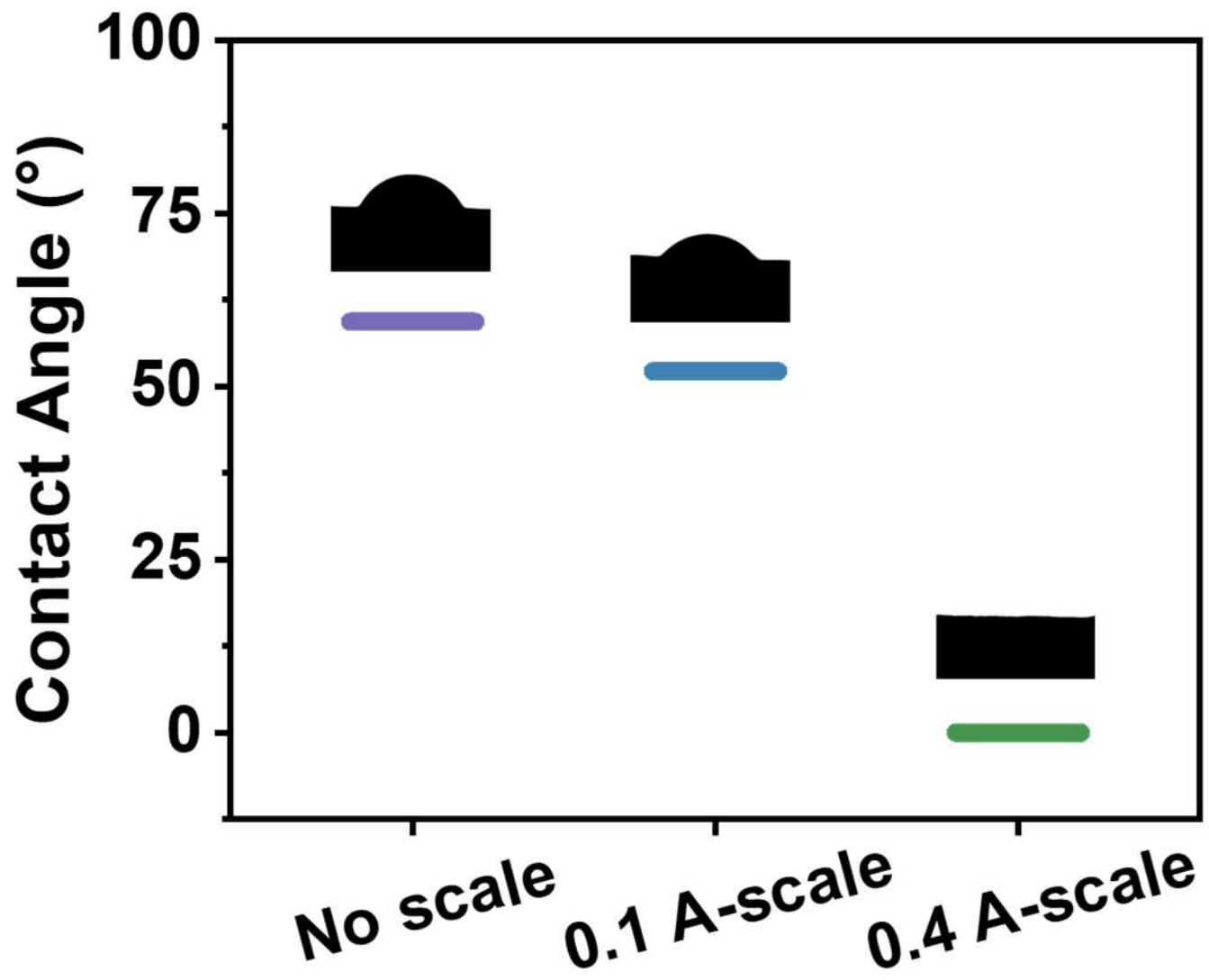

Supplementary Fig. 19. Contact angle measurements of the C0 cathodes with varying degrees of scaling. Reaction conditions: [Na_2SO_4] = 0.05 M, [Ca^{2+}]$_0$ = 200 mg L^{-1}, a rotation speed of 600 rpm, an initial pH of 7.0 ± 0.1 and electrolysis of 3 h. Source data for Supplementary Fig. 19 are provided as a Source Data file.

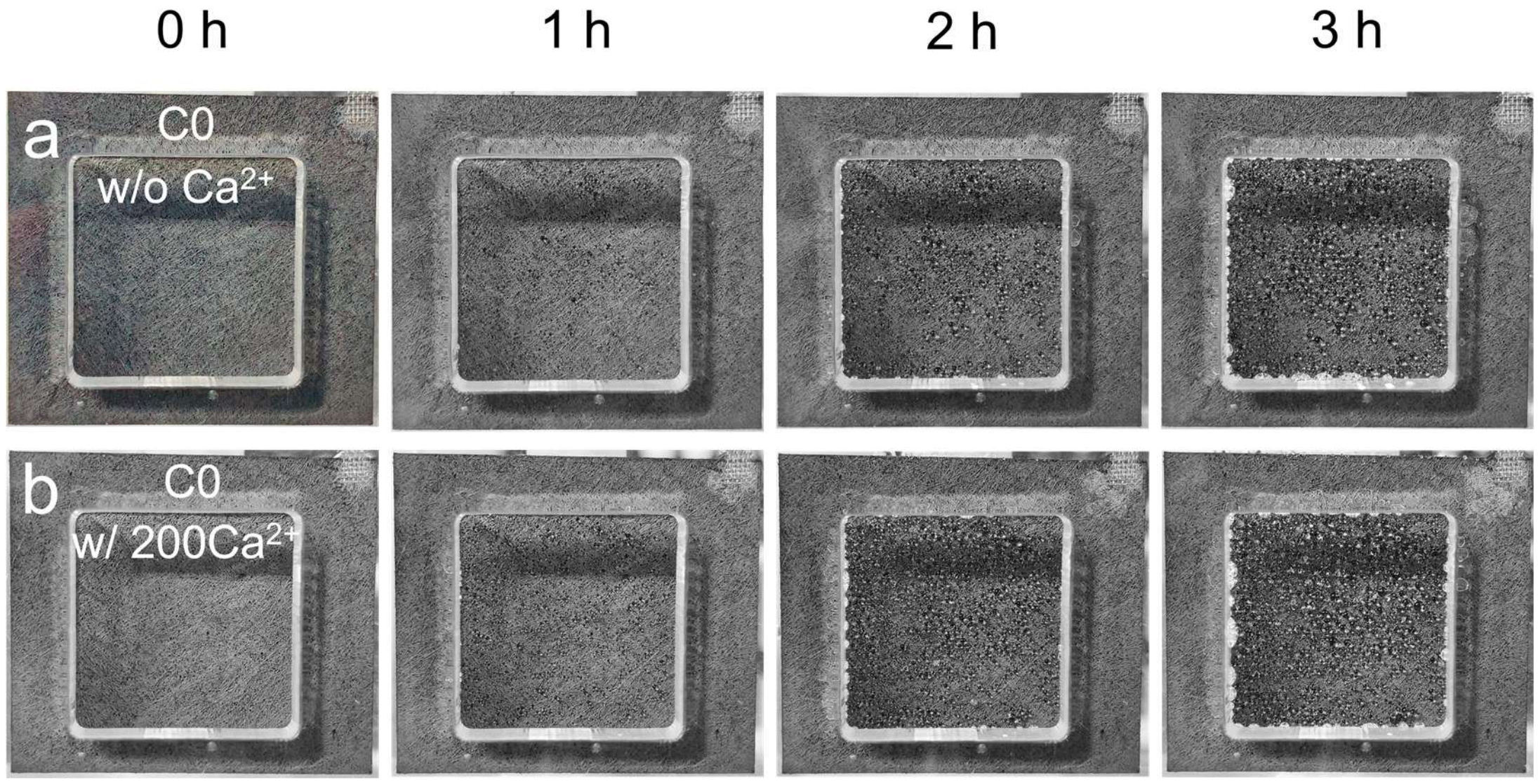

Supplementary Fig. 20. Optical images of the C0-GDL water flooding over time in the (a) absence and (b) presence of 200 mg L^{-1} Ca^{2+} at 0.1 A.

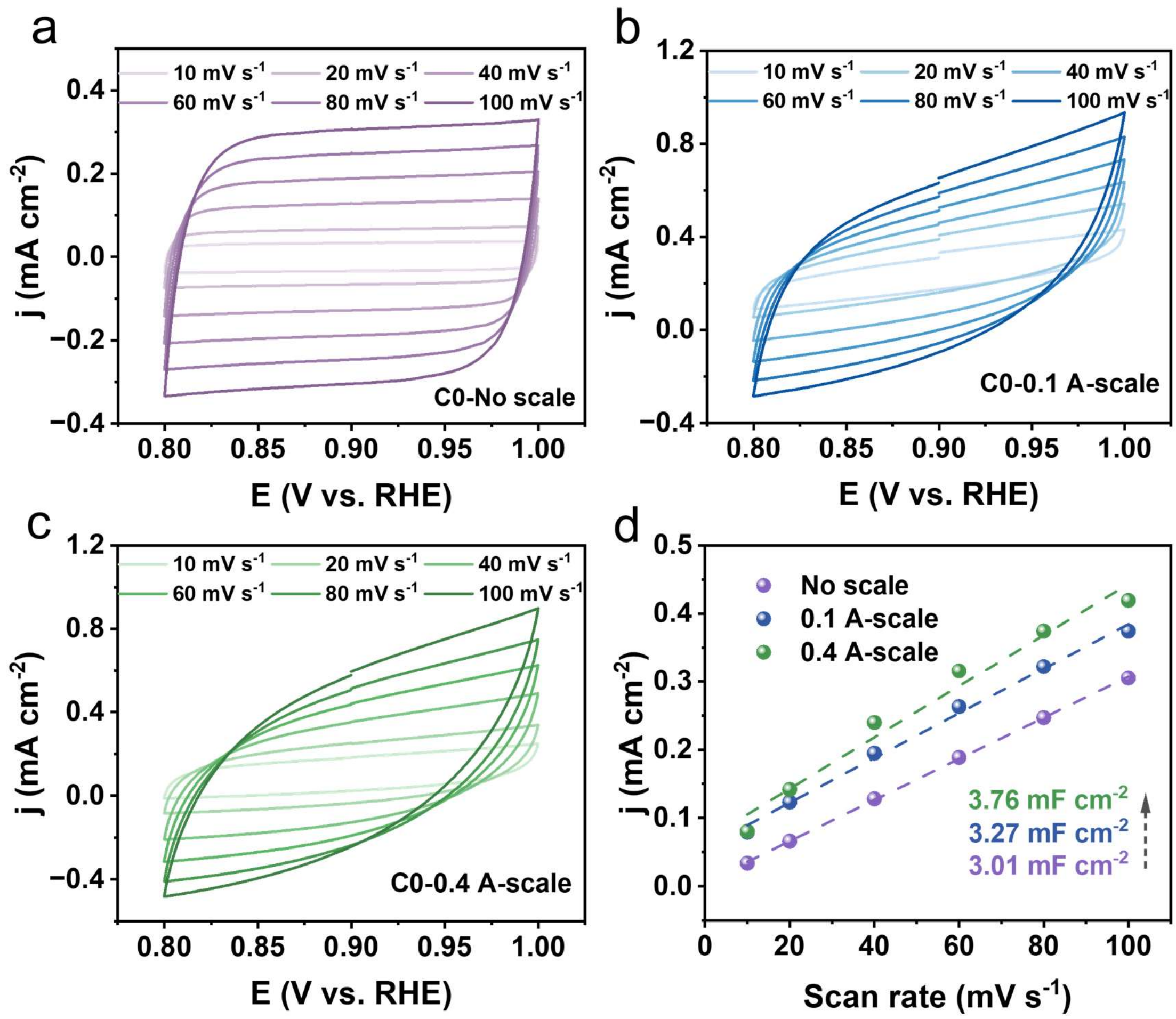

Supplementary Fig. 21. CV curves of the (a) No-scale, (b) 0.1 A-scale and (c) 0.4 A-scale C0 cathodes in N_2-saturated 0.1 M Na_2SO_4 electrolyte for ECSA measurements. (d) Calculated ECSA of C0 cathodes with varying degrees of scaling. All the potentials have not been iR-corrected. Source data for Supplementary Fig. 21 are provided as a Source Data file.

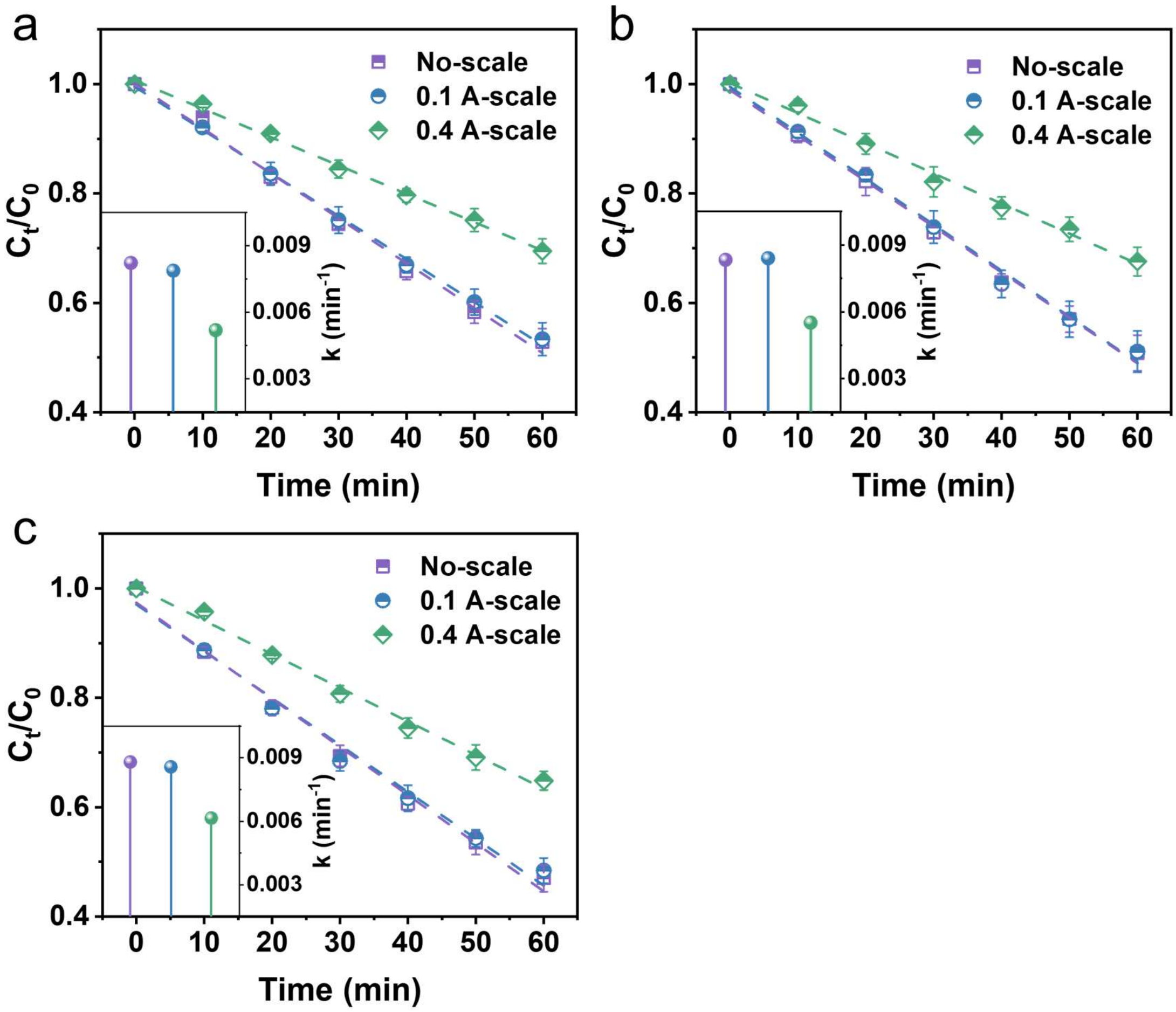

Supplementary Fig. 22. H_2O_2 decomposition curves and H_2O_2 decomposition rate constant k of C0 cathodes with varying degrees of scaling at (a) 0.1 A, (b) 0.2 A and (c) 0.4 A with an initial H_2O_2 concentration of 1200 mg L^{-1}. Reaction conditions: [Na_2SO_4] = 0.05 M, a rotation speed of 600 rpm, an initial pH of 7.0 ± 0.1, and electrolysis of 1 h. The data is presented as mean ± s.d. (n = 3). Source data for Supplementary Fig. 22 are provided as a Source Data file.

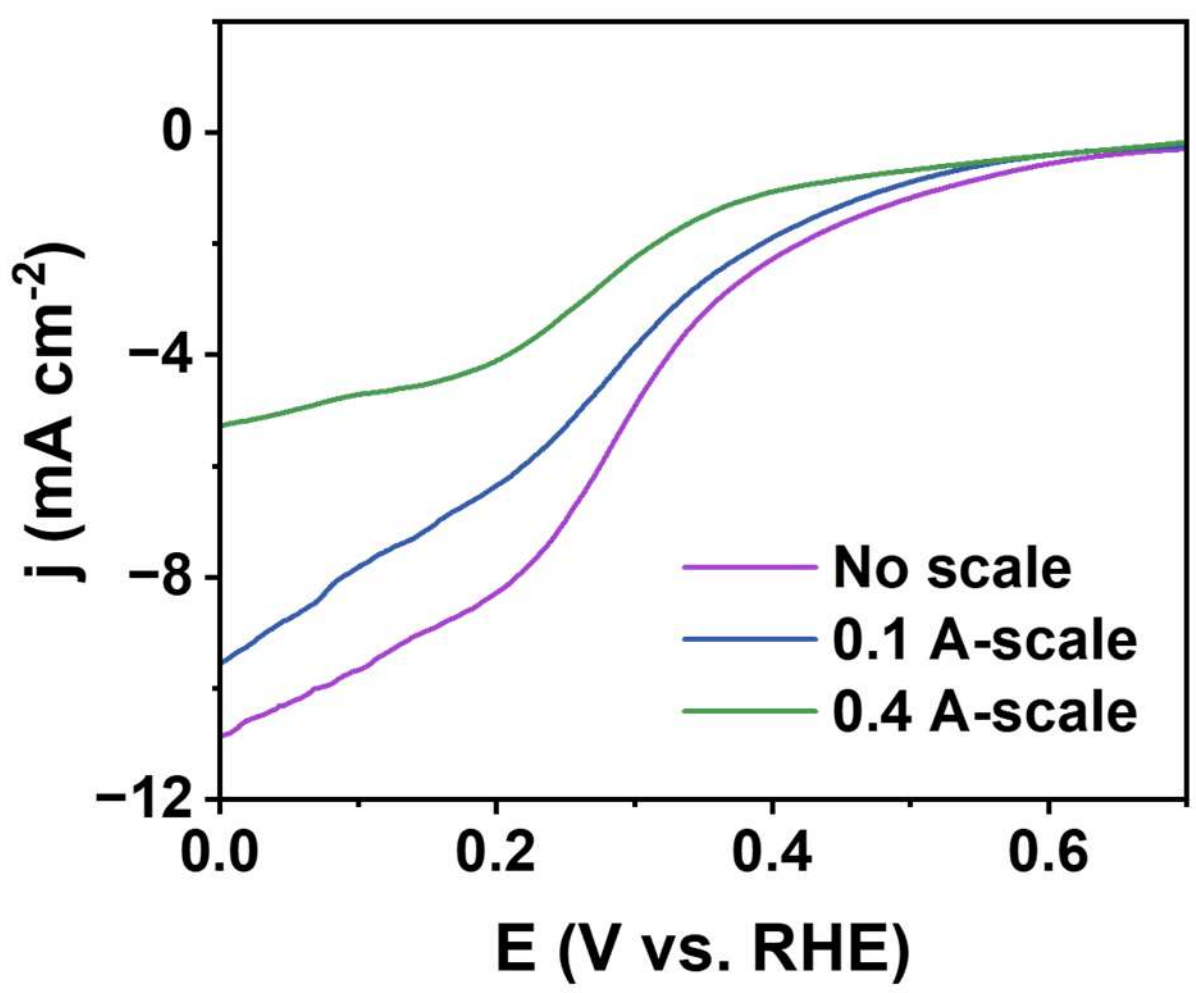

Supplementary Fig. 23. LSV curves of H_2O_2 reduction for C0 cathodes with varying degrees of scaling in N_2-saturated 0.1 M Na_2SO_4 solution containing 10 mM H_2O_2. All the potentials have not been iR-corrected. Source data for Supplementary Fig. 23 are provided as a Source Data file.

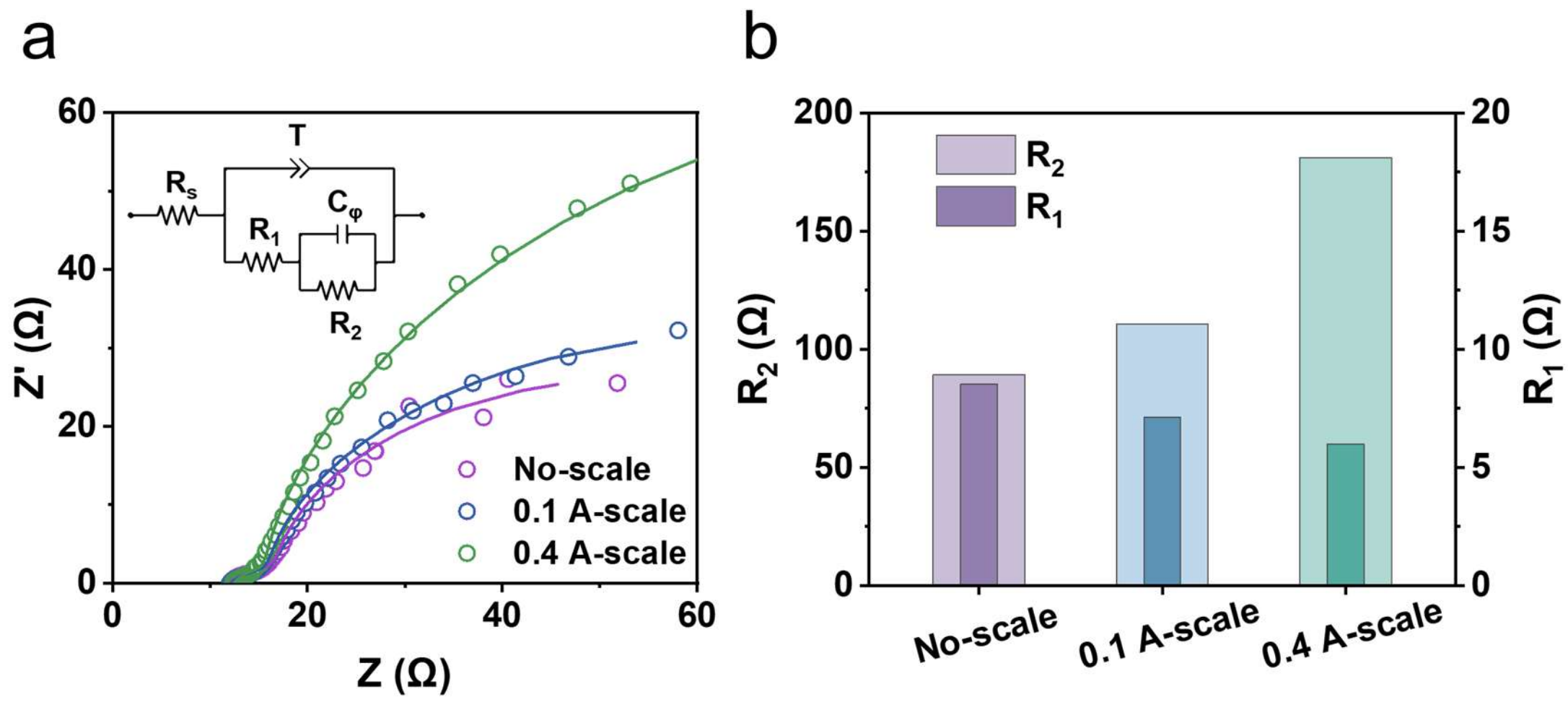

Supplementary Fig. 24. (a) EIS Nyquist plots and (b) impedance components of H_2O_2 reduction for C0 cathodes with varying degrees of scaling in N_2-saturated 0.1 M Na_2SO_4 solution containing 10 mM H_2O_2. The inset in (a) shows the equivalent circuit. R_s is the solution resistance. T represents the double layer capacitance. R_1 is the charge transfer resistance, associated with the main electrochemical reaction. R_2 and C_φ represent the hydrogen adsorption resistance and pseudo-capacitance, respectively. All the potentials have not been iR-corrected. Source data for Supplementary Fig. 24 are provided as a Source Data file.

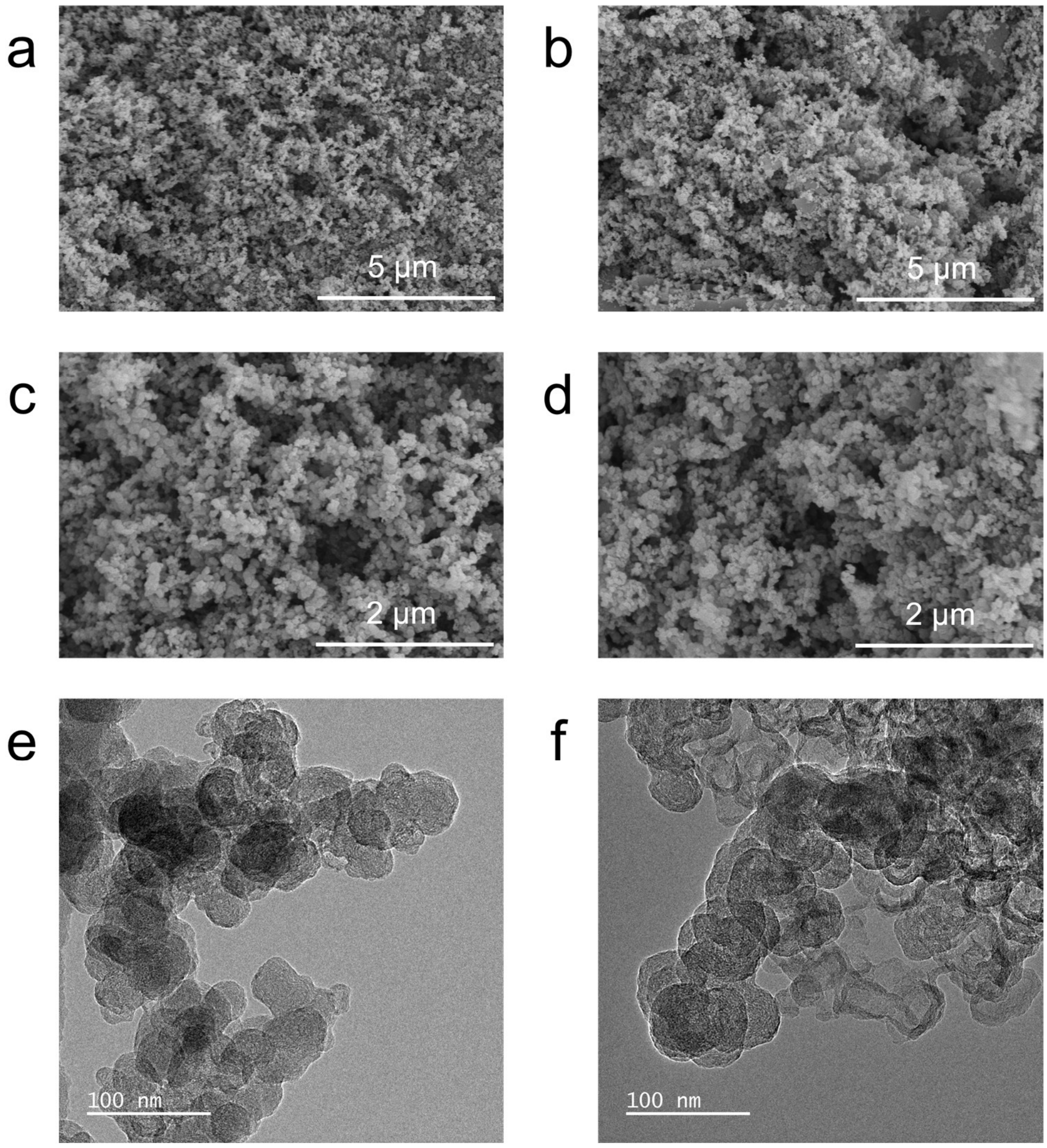

Supplementary Fig. 25. SEM images of (a, c) C0 and (b, d) C18. TEM images of (e) C0 and (f) C18.

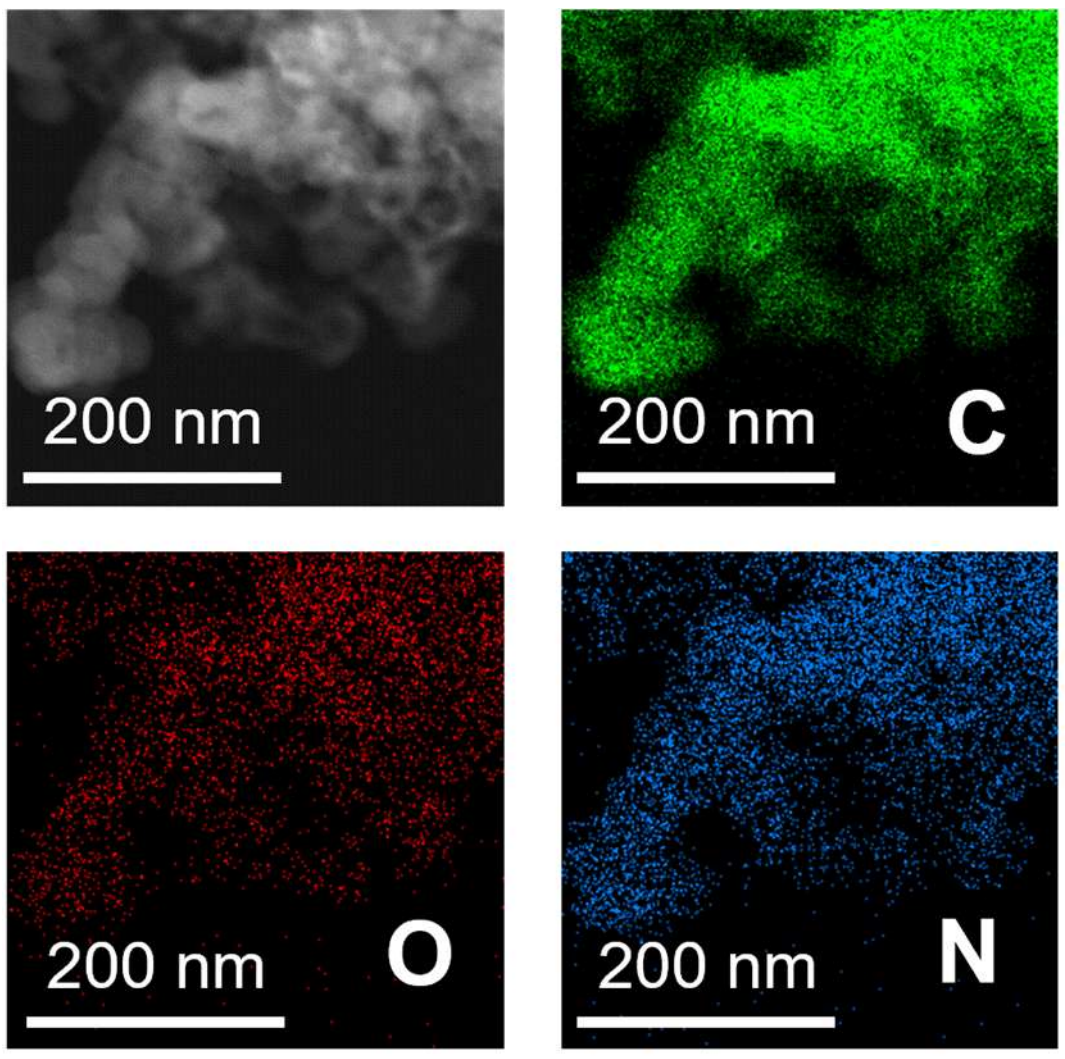

Supplementary Fig. 26. EDS mapping images of C18.

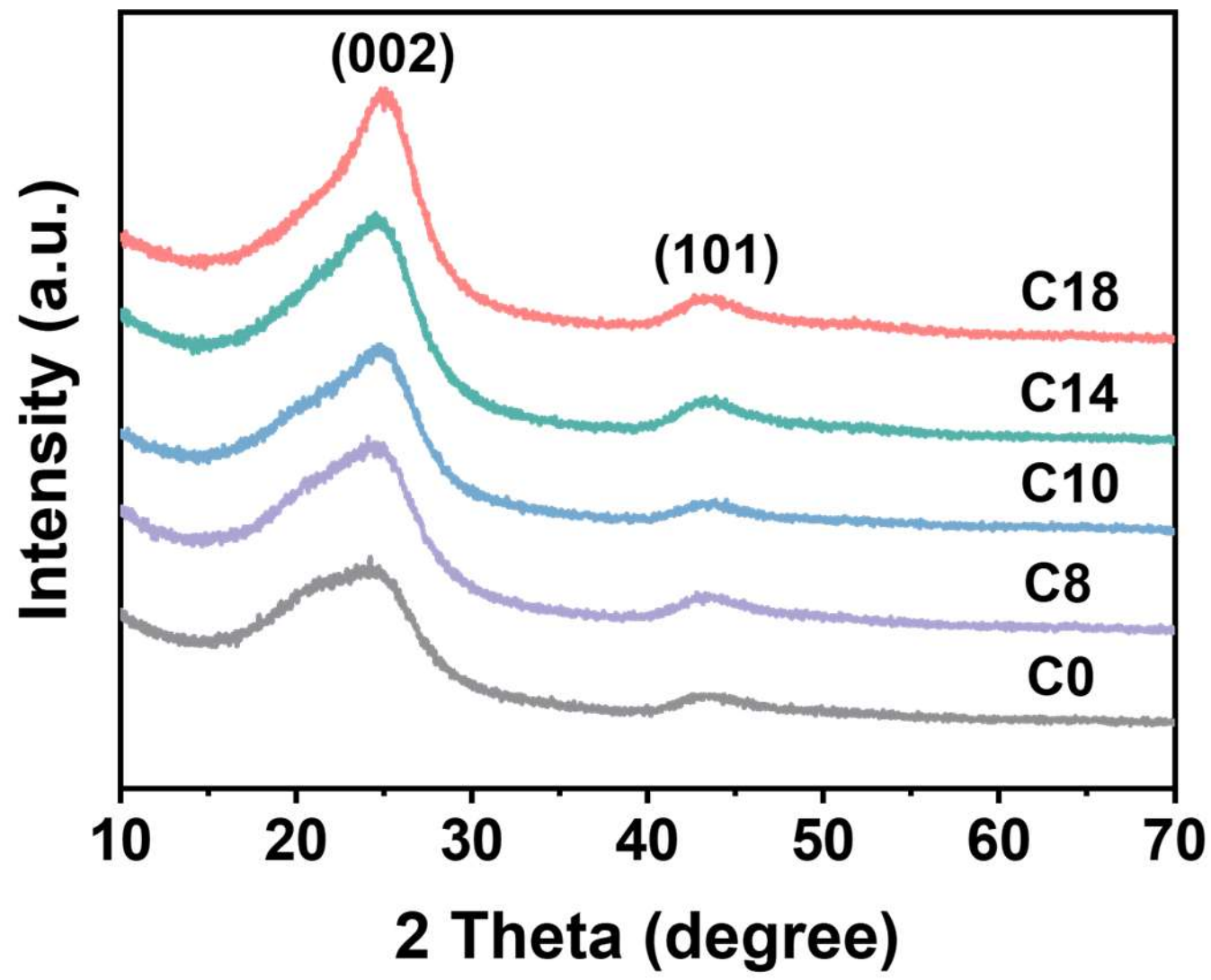

Supplementary Fig. 27. XRD patterns of different Cx catalysts. Source data for Supplementary Fig. 27 are provided as a Source Data file.

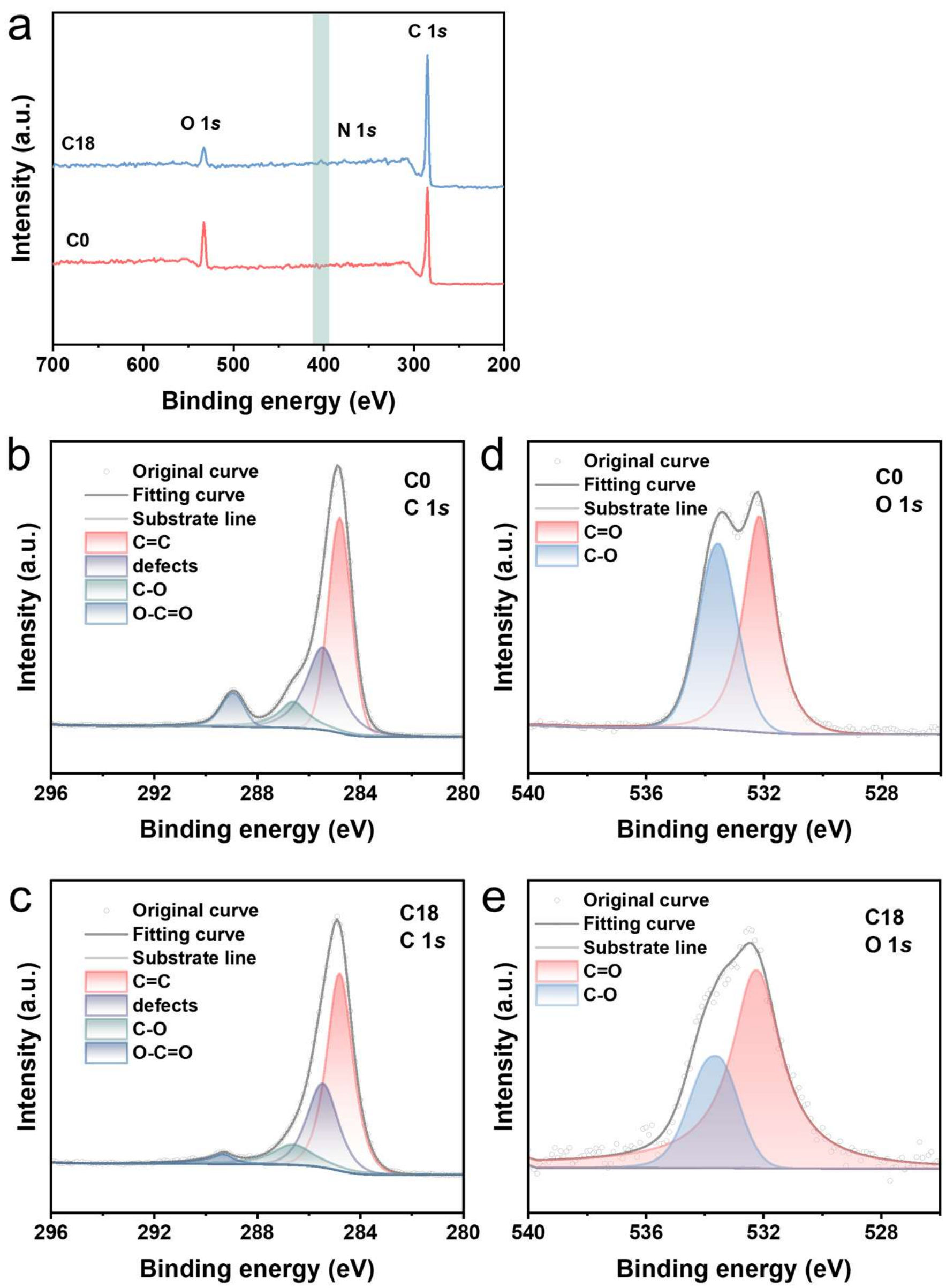

Supplementary Fig. 28. (a) XPS spectra of C0 and C18. XPS high-resolution C 1*s* spectra of (b) C0 and (c) C18. XPS high-resolution O 1*s* spectra of (d) C0 and (e) C18. Source data for Supplementary Fig. 28 are provided as a Source Data file.

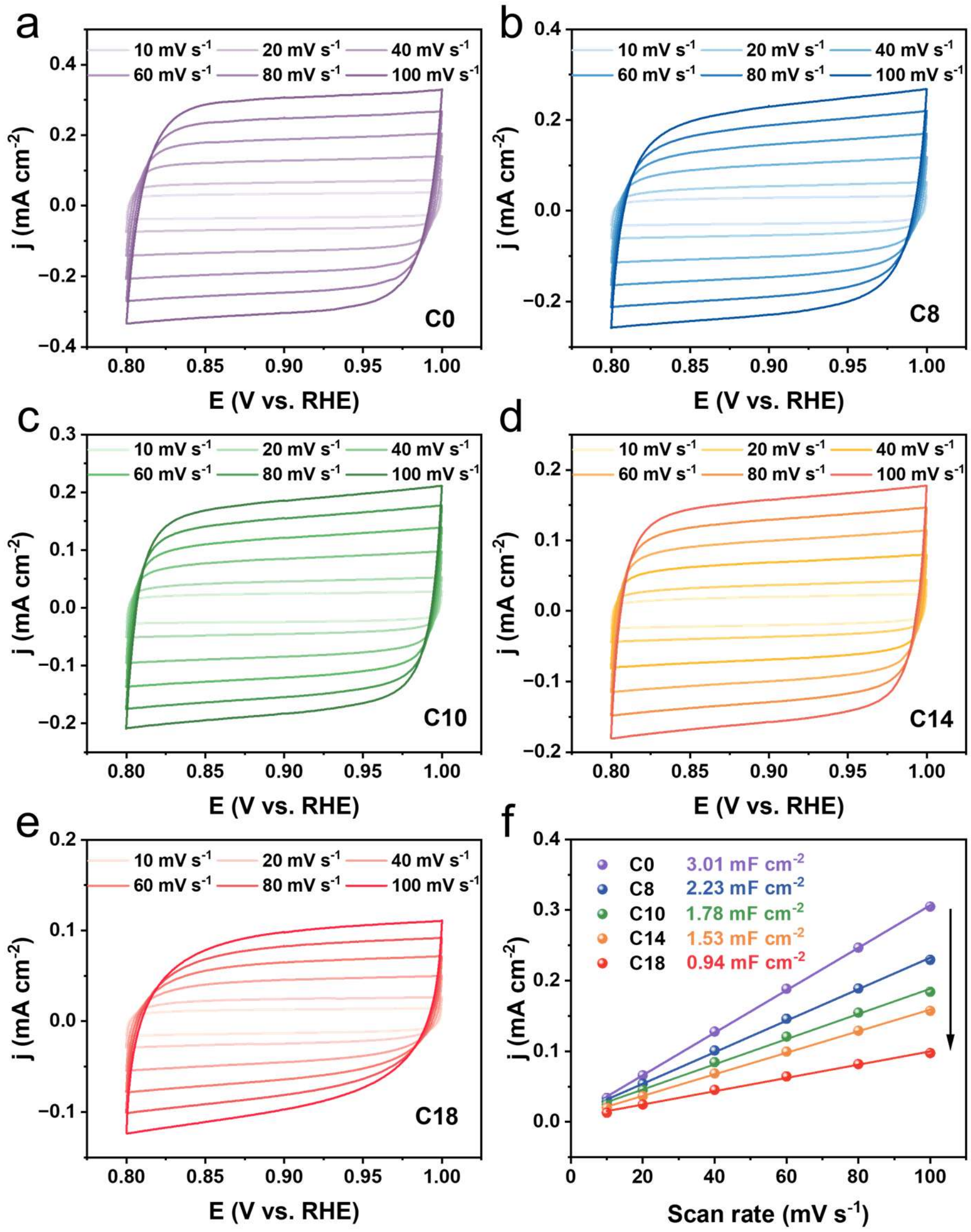

Supplementary Fig. 29. CV curves of the (a) C0, (b) C8, (c) C10, (d) C14 and (e) C18 cathodes in N_2-saturated 0.1 M Na_2SO_4 electrolyte for ECSA measurements. (f) Calculated ECSA of different Cx cathodes based on CV curves at different sweep rates. All the potentials have not been iR-corrected. Source data for Supplementary Fig. 29 are provided as a Source Data file.

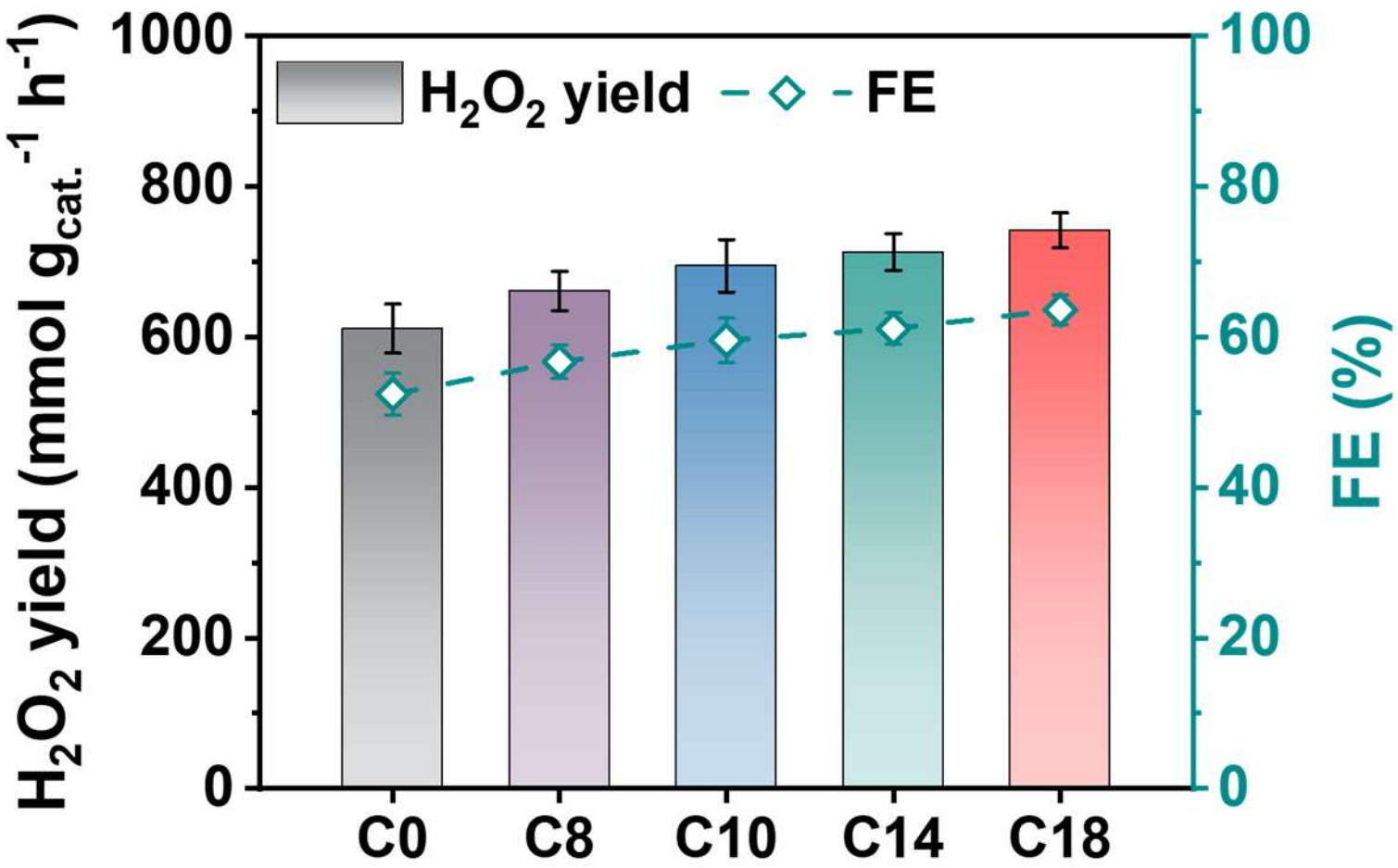

Supplementary Fig. 30. H_2O_2 electrosynthesis yields and Faraday efficiencies of different Cx cathodes in 0.05 M Na_2SO_4 solution. Reaction conditions: [Na_2SO_4] = 0.05 M, a current of 0.1 A, a rotation speed of 600 rpm, electrolysis time of 3 h, and an initial pH of 7.0 ± 0.1. The data is presented as mean ± s.d. (n = 3). Source data for Supplementary Fig. 30 are provided as a Source Data file.

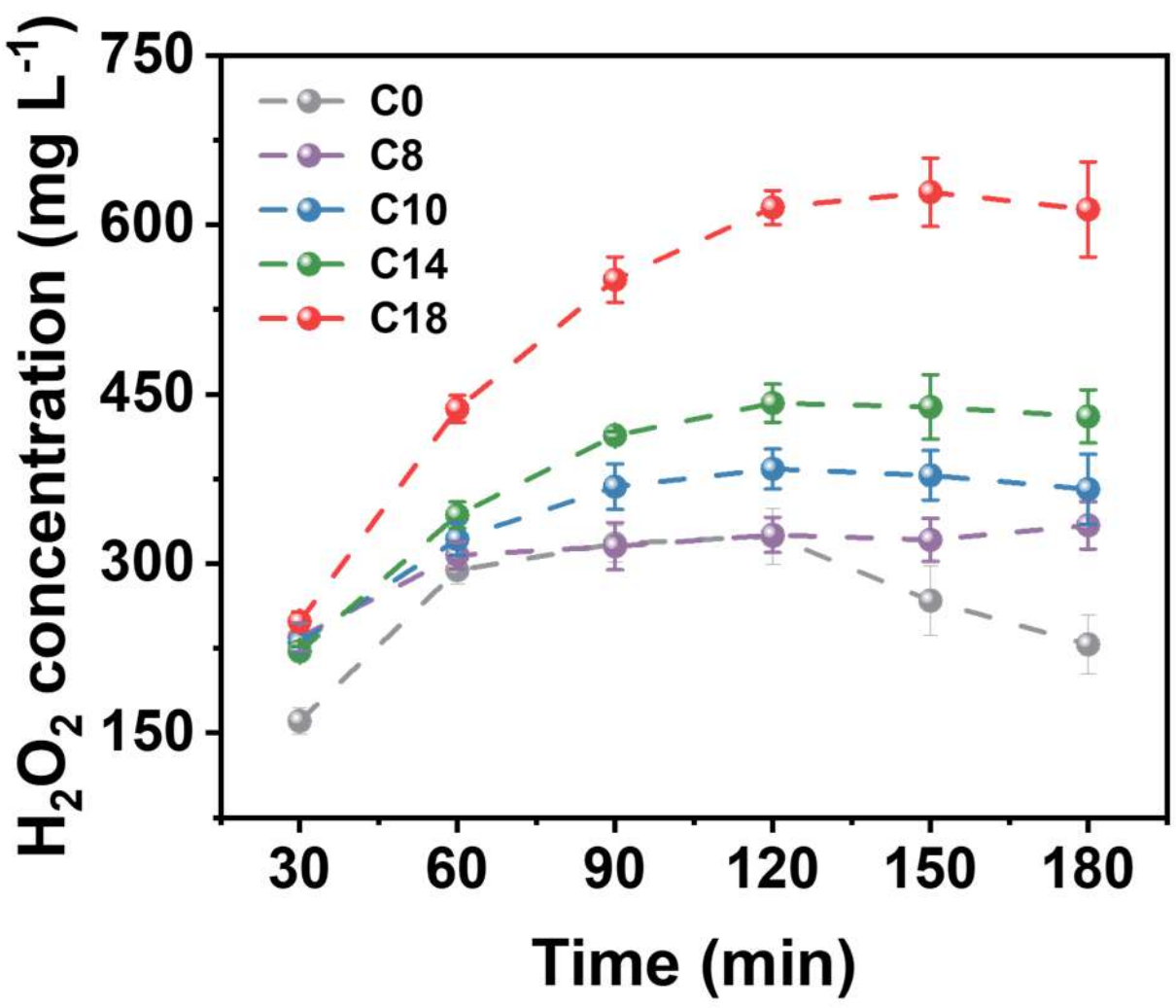

Supplementary Fig. 31. Time-dependent curves depicting H_2O_2 concentration. Reaction conditions: $[Na_2SO_4]$ = 0.05 M, $[Ca^{2+}]_0$ = 200 mg L^{-1}, a current of 0.1 A, a rotation speed of 600 rpm, electrolysis time of 3 h, and an initial pH of 7.0 ± 0.1. The data is presented as mean ± s.d. (n = 3). Source data for Supplementary Fig. 31 are provided as a Source Data file.

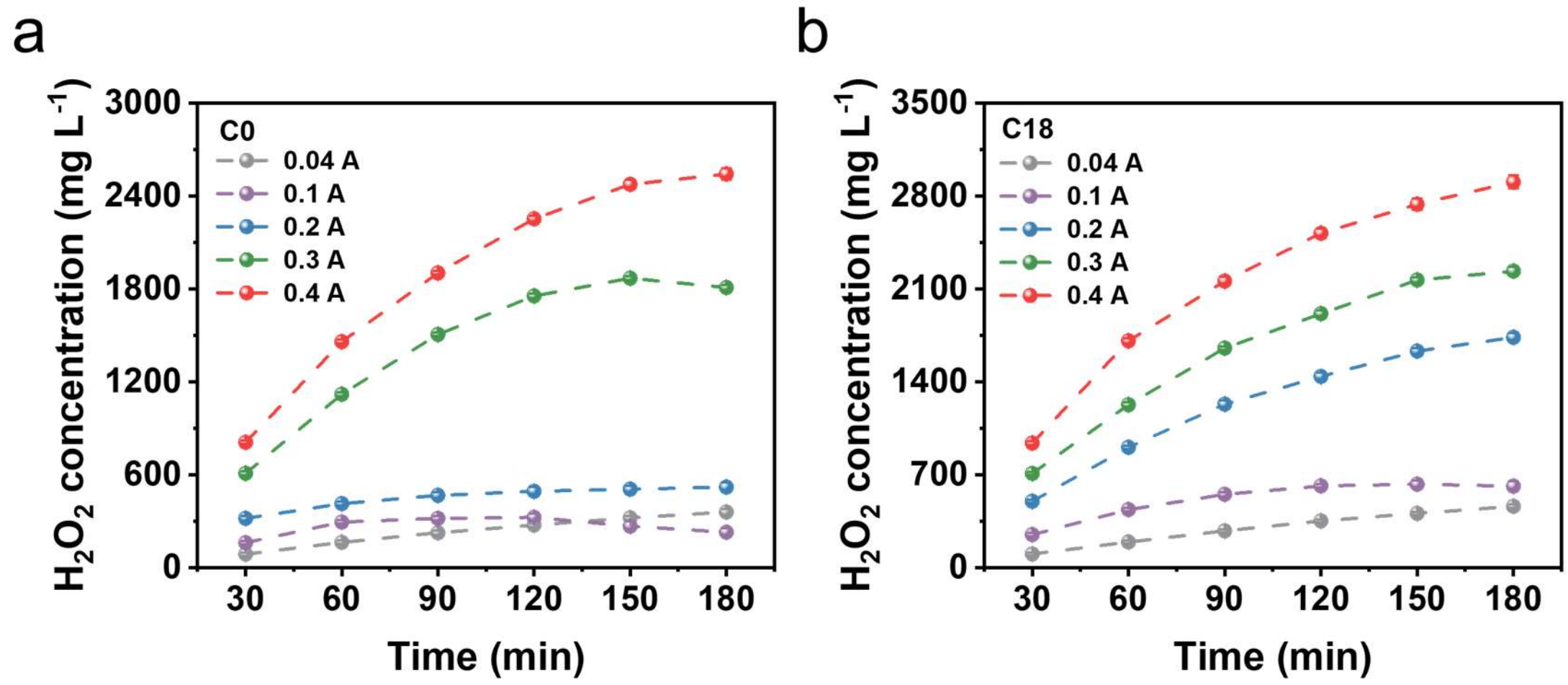

Supplementary Fig. 32. Time-dependent curves depicting H_2O_2 concentration of the (a) C0 and (b) C18 cathode at different currents. Reaction conditions: $[Na_2SO_4]$ = 0.05 M, $[Ca^{2+}]_0$ = 200 mg L^{-1}, a rotation speed of 600 rpm, electrolysis time of 3 h, and an initial pH of 7.0 ± 0.1. The data is presented as mean ± s.d. (n = 3). Source data for Supplementary Fig. 32 are provided as a Source Data file.

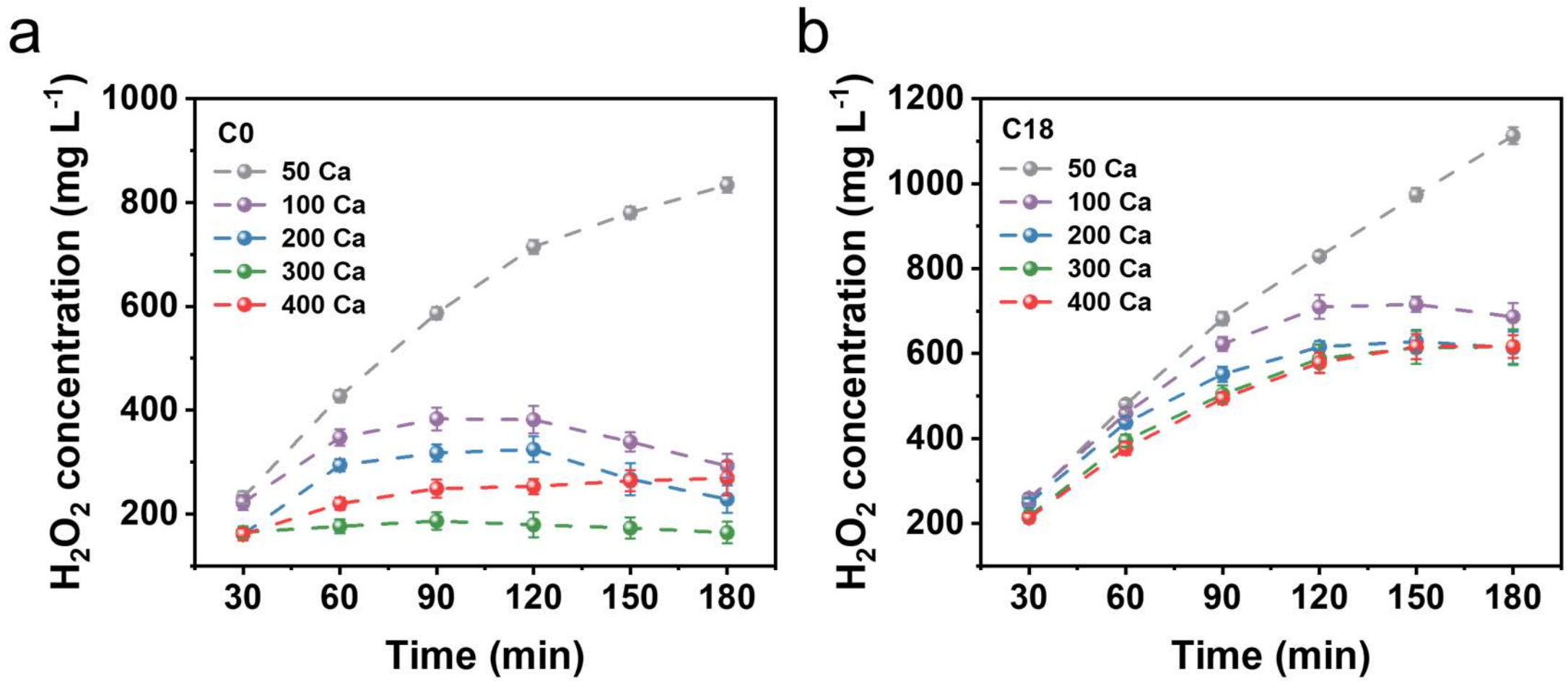

Supplementary Fig. 33. Time-dependent curves depicting H_2O_2 concentration of the (a) C0 and (b) C18 cathode under different initial concentrations of Ca^{2+}. Reaction conditions: $[Na_2SO_4]$ = 0.05 M, a current of 0.1 A, a rotation speed of 600 rpm, electrolysis time of 3 h, and an initial pH of 7.0 ± 0.1. The data is presented as mean ± s.d. (n = 3). Source data for Supplementary Fig. 33 are provided as a Source Data file.

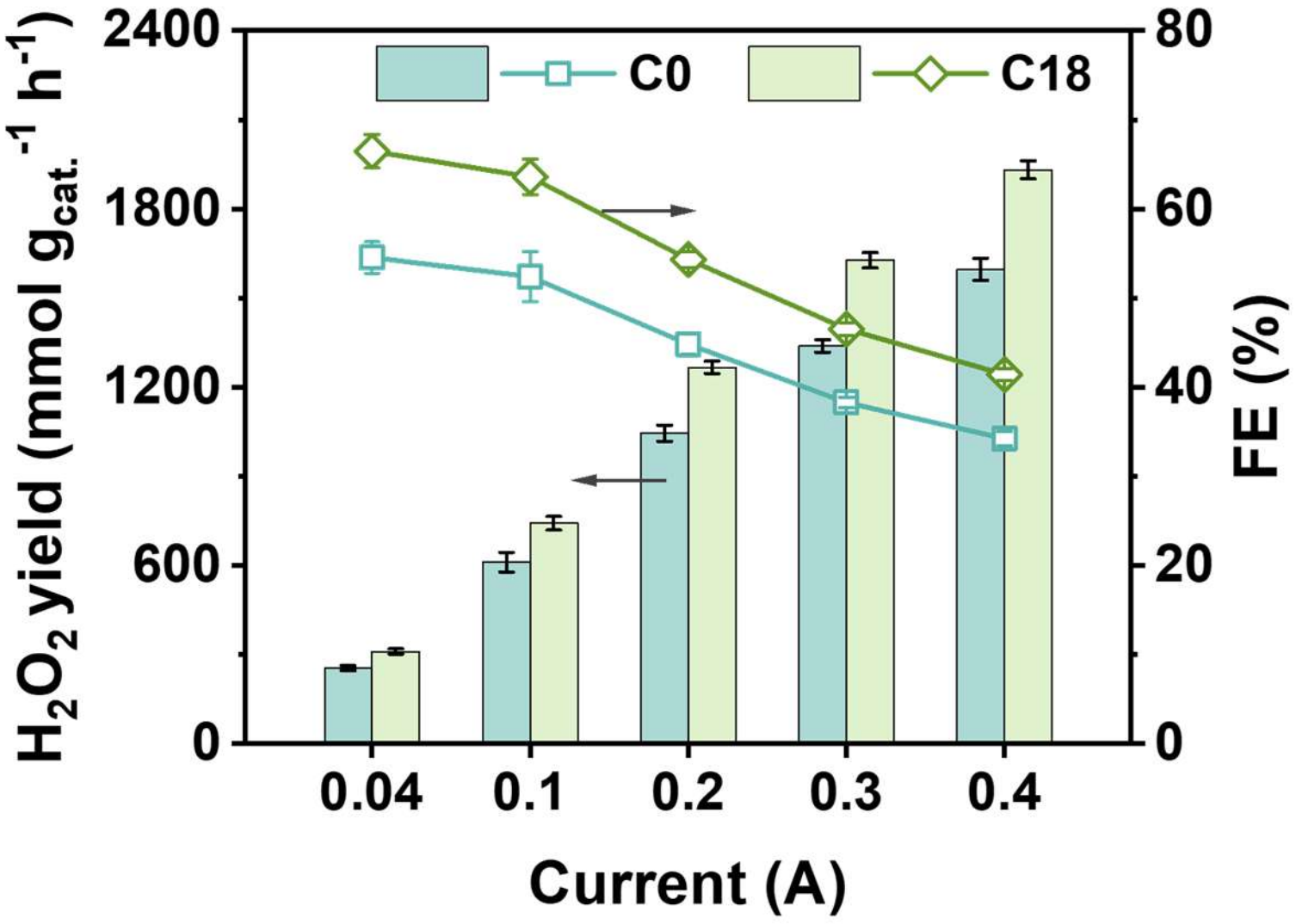

Supplementary Fig. 34. H_2O_2 electrosynthesis yields and Faraday efficiencies of the C0 and C18 cathode at different currents in 0.05 M Na_2SO_4 solution. Reaction conditions: [Na_2SO_4] = 0.05 M, a rotation speed of 600 rpm, electrolysis time of 3 h, and an initial pH of 7.0 ± 0.1. The data is presented as mean ± s.d. (n = 3). Source data for Supplementary Fig. 34 are provided as a Source Data file.

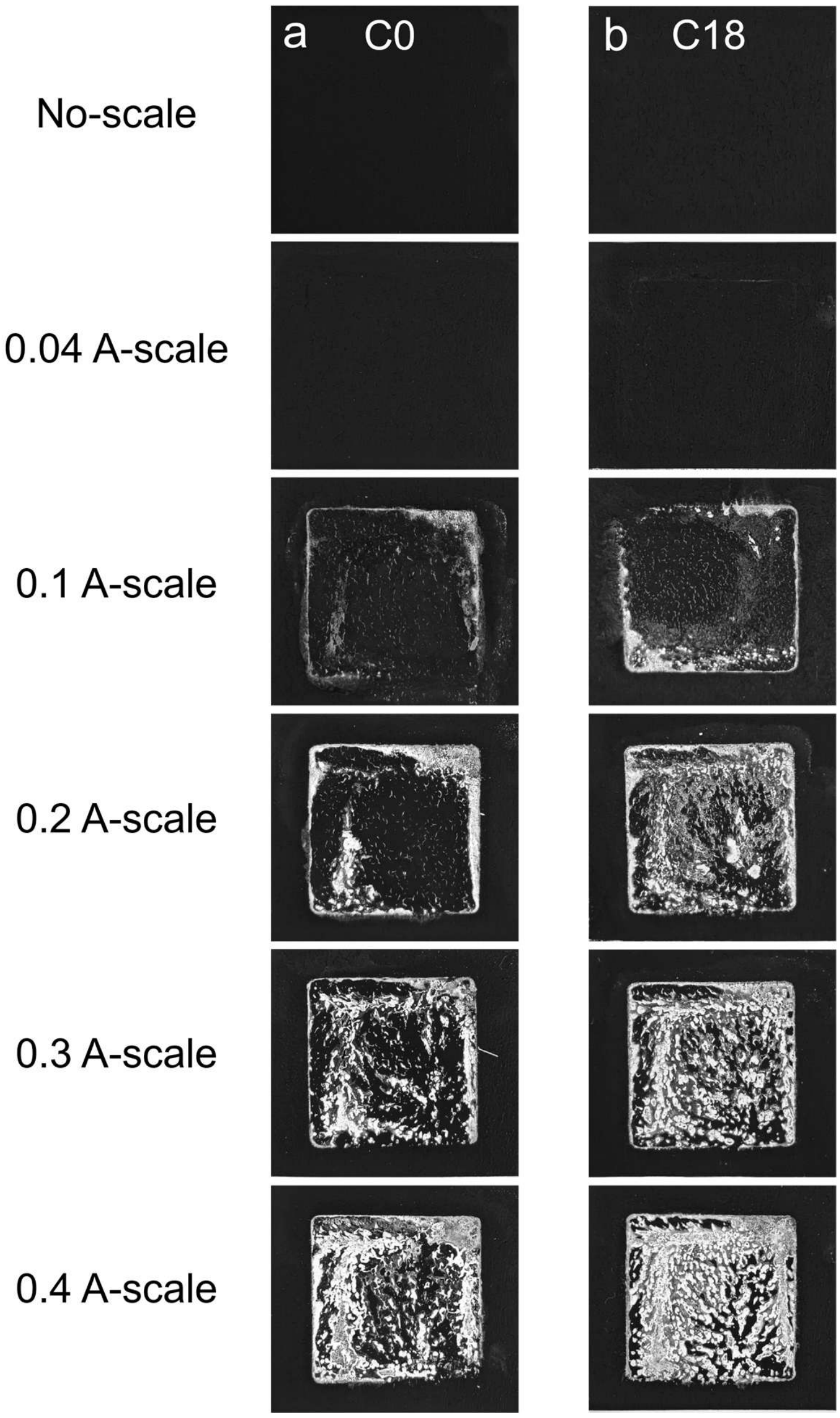

Supplementary Fig. 35. Optical images of (a) C0 and (b) C18 cathodes before and after experiments in the presence of 200 mg L^{-1} Ca^{2+} at different currents.

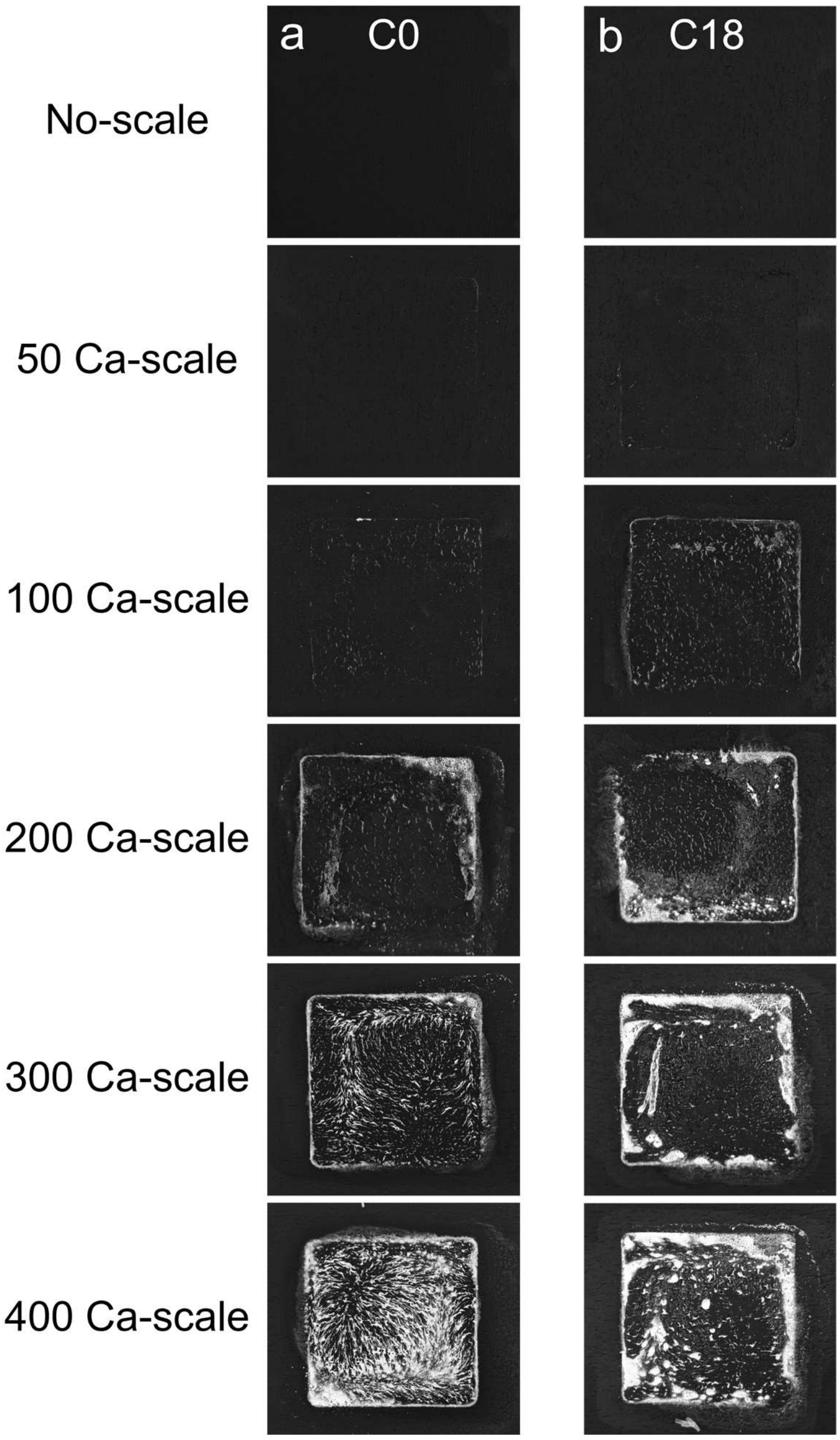

Supplementary Fig. 36. Optical images of (a) C0 and (b) C18 cathodes before and after experiments at 0.1 A under different initial concentrations of Ca^{2+}.

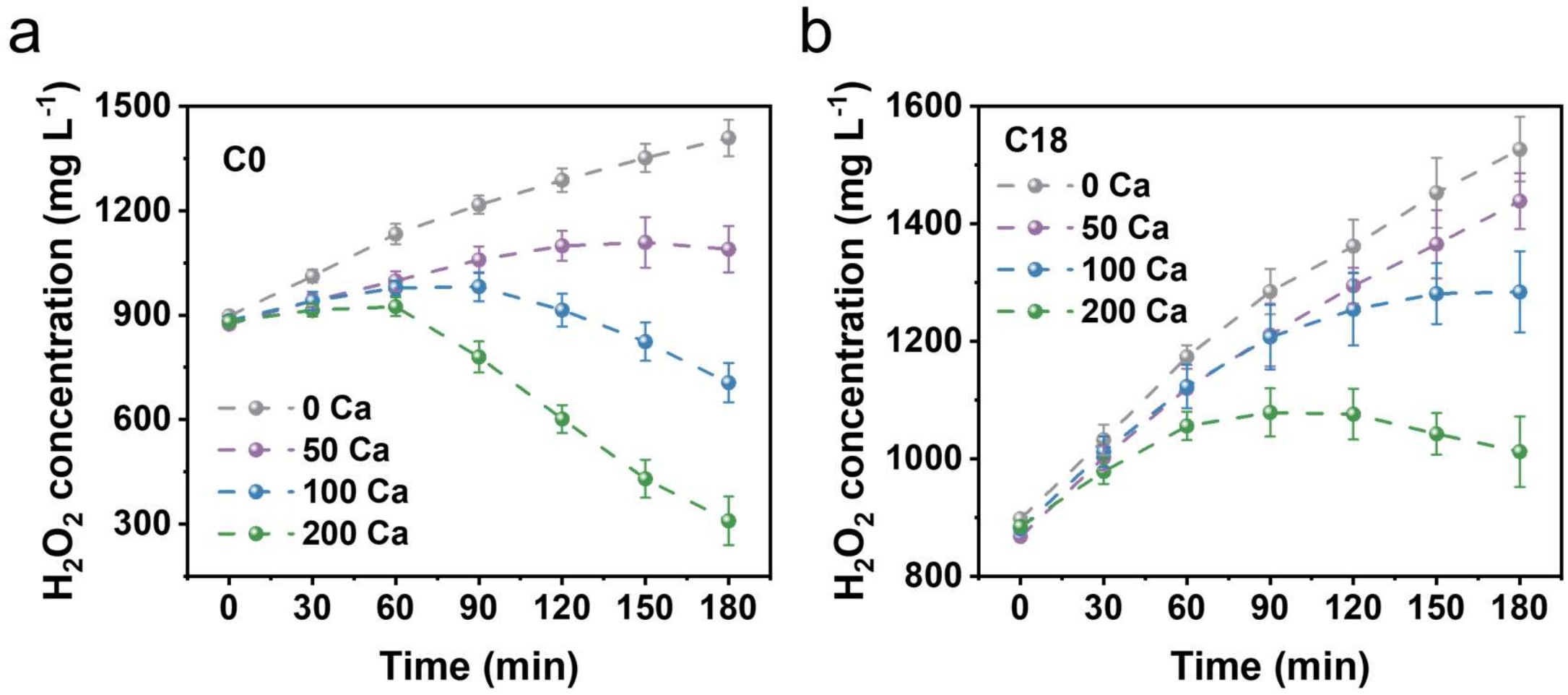

Supplementary Fig. 37. Time-dependent curves depicting H_2O_2 concentration of the (a) C0 and (b) C18 cathodes under varying Ca^{2+} concentrations at an initial H_2O_2 concentration of 900 mg L^{-1}. Reaction conditions: [Na_2SO_4] = 0.05 M, a current of 0.1 A, a rotation speed of 600 rpm, electrolysis time of 3 h, and an initial pH of 7.0 ± 0.1. The data is presented as mean ± s.d. (n = 3). Source data for Supplementary Fig. 37 are provided as a Source Data file.

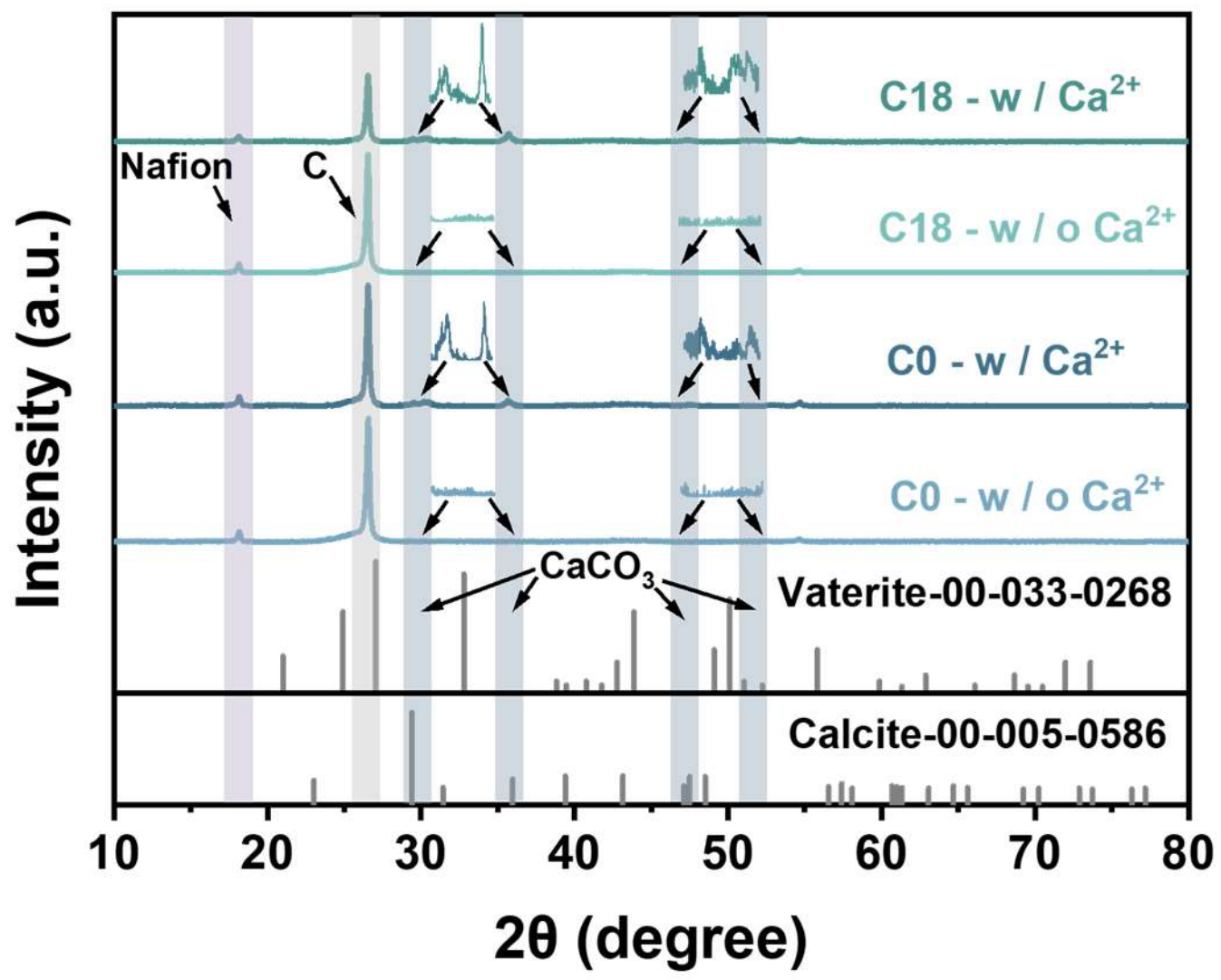

Supplementary Fig. 38. XRD patterns of the C0 and C18 cathodes after experiments at 0.1 A in the presence or absence of 200 mg L^{-1} Ca^{2+}. Source data for Supplementary Fig. 38 are provided as a Source Data file.

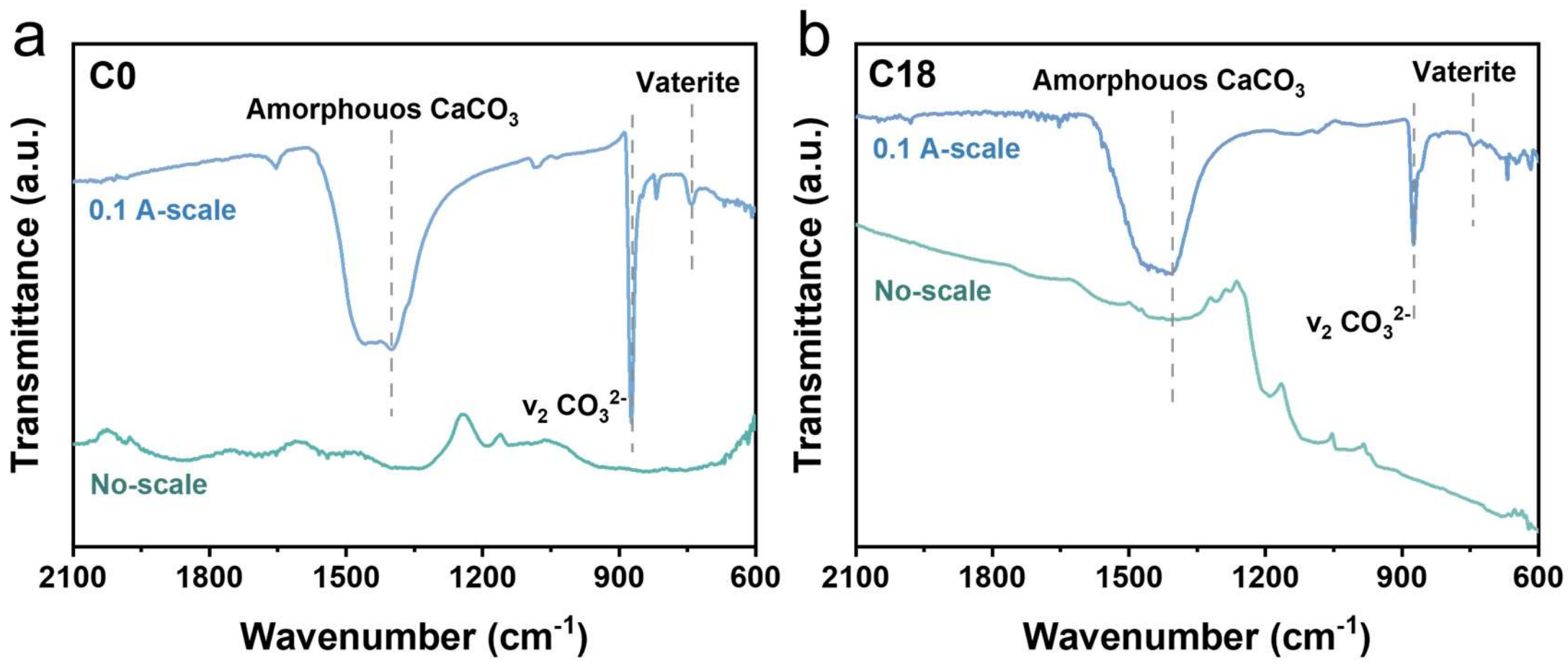

Supplementary Fig. 39. FTIR spectra of the (a) C0 and (b) C18 cathodes after experiments at 0.1 A in the presence or absence of 200 mg L^{-1} Ca^{2+}. Source data for Supplementary Fig. 39 are provided as a Source Data file.

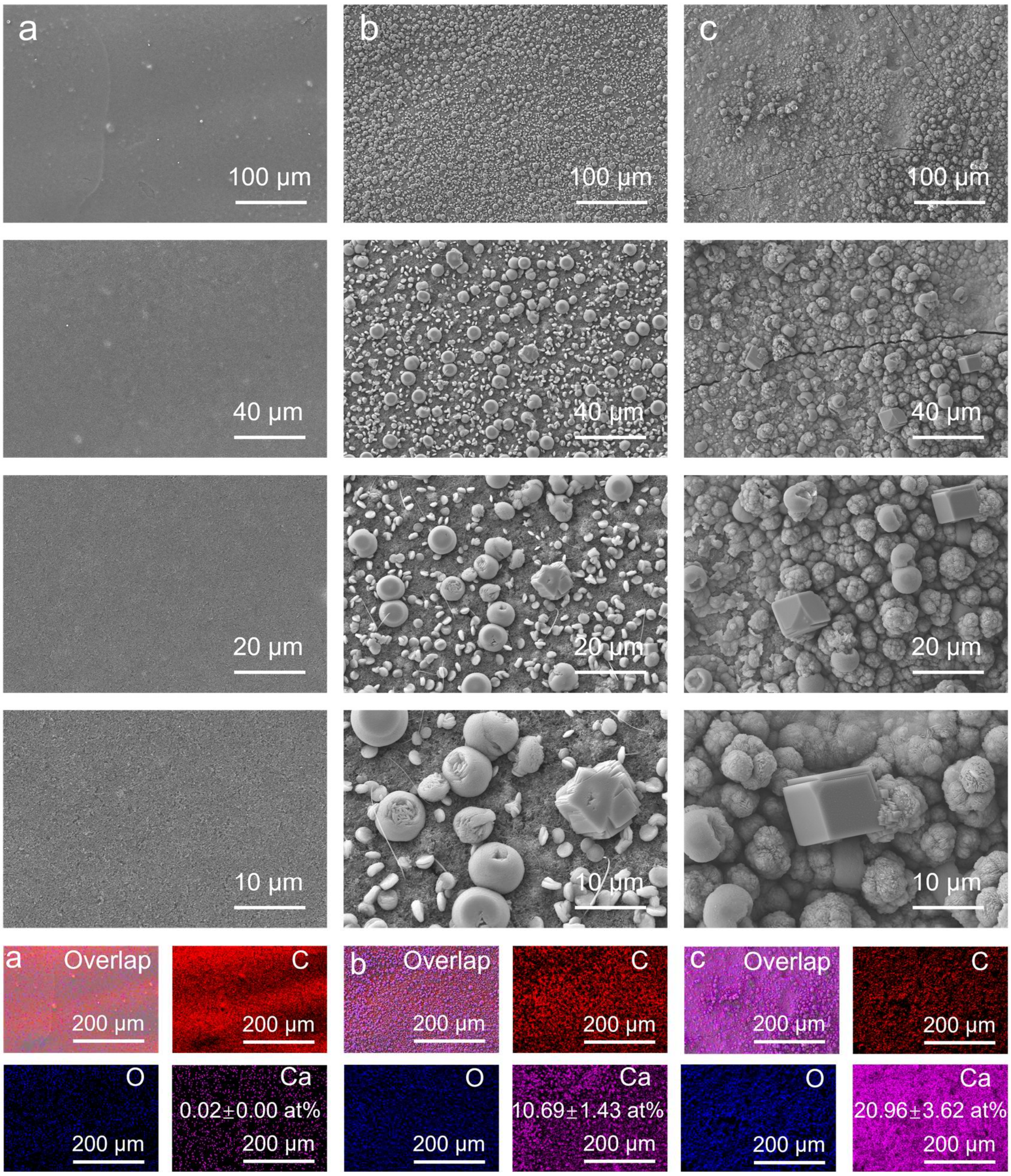

Supplementary Fig. 40. SEM images and EDS maps of the C18 cathode (a) before experiment, (b) after experiment at 0.1 A and (c) after experiment at 0.4 A. Reaction conditions: $[Na_2SO_4]$ = 0.05 M, $[Ca^{2+}]_0$ = 200 mg L^{-1}, a rotation speed of 600 rpm, an initial pH of 7.0 ± 0.1 and electrolysis of 3 h.

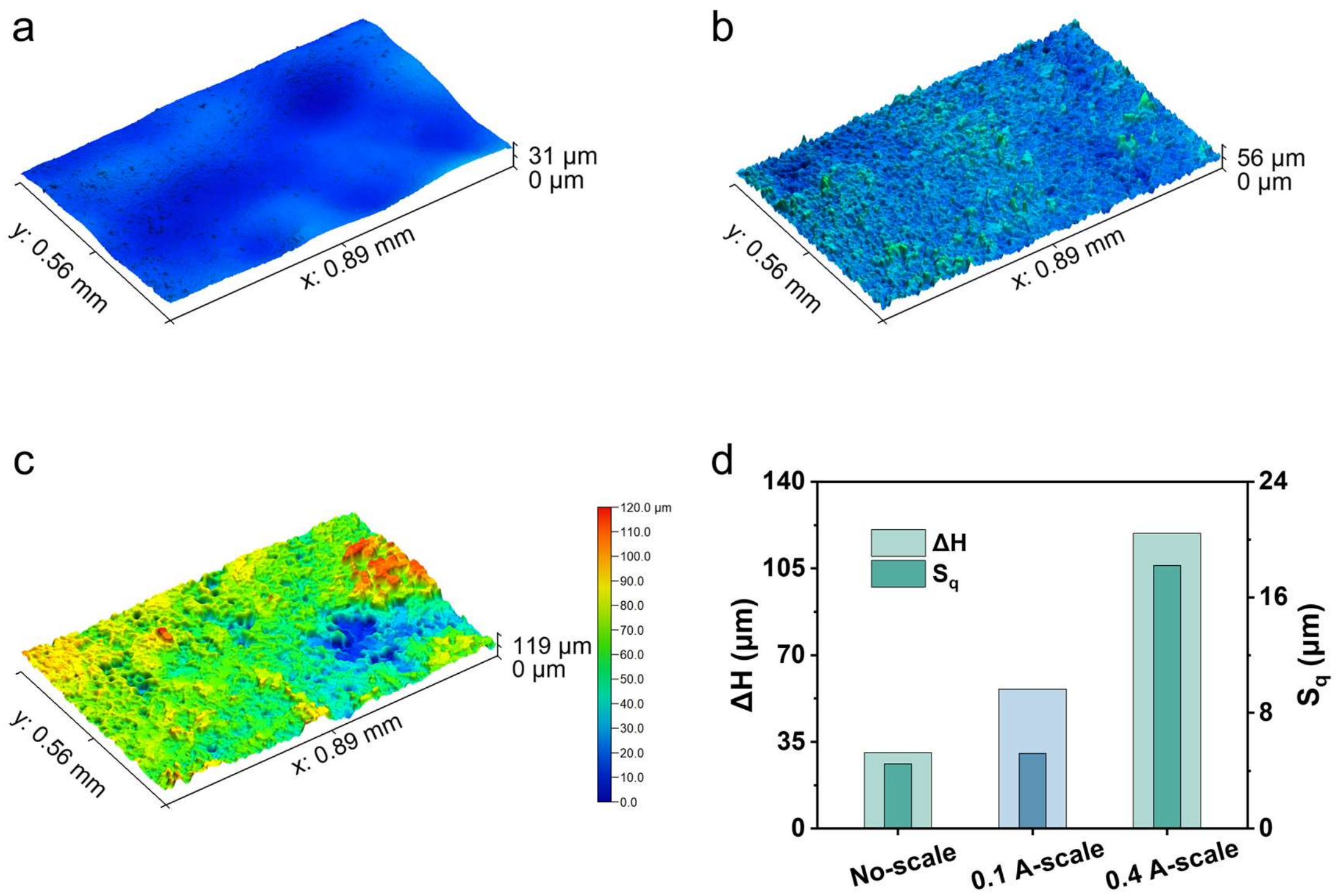

Supplementary Fig. 41. 3D optical profilometry images of the C18 cathode (a) before experiment, (b) after experiment at 0.1 A and (c) after experiment at 0.4 A. (d) Variations in the height range (ΔH) and RMS roughness (S_q) of the C18 cathodes with varying degrees of scaling. Reaction conditions: $[Na_2SO_4]$ = 0.05 M, $[Ca^{2+}]_0$ = 200 mg L^{-1}, a rotation speed of 600 rpm, an initial pH of 7.0 ± 0.1 and electrolysis of 3 h. Source data for Supplementary Fig. 41d are provided as a Source Data file.

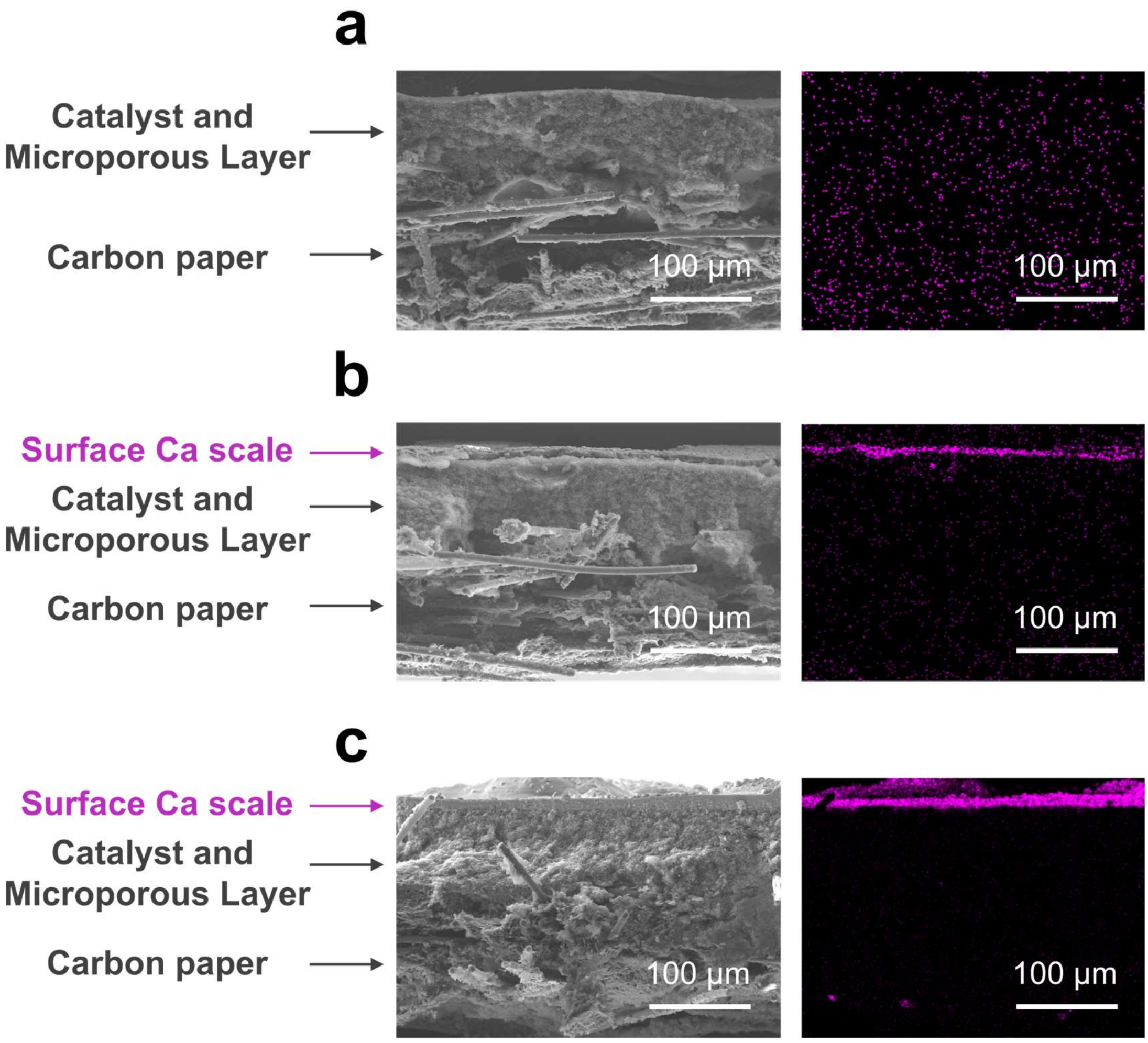

Supplementary Fig. 42. SEM images of cross-sectional view and EDS maps of Ca element for the C18 cathode (a) before experiment, (b) after experiment at 0.1 A and (c) after experiment at 0.4 A. Reaction conditions: [Na_2SO_4] = 0.05 M, [Ca^{2+}]$_0$ = 200 mg L^{-1}, a rotation speed of 600 rpm, an initial pH of 7.0 ± 0.1 and electrolysis of 3 h.

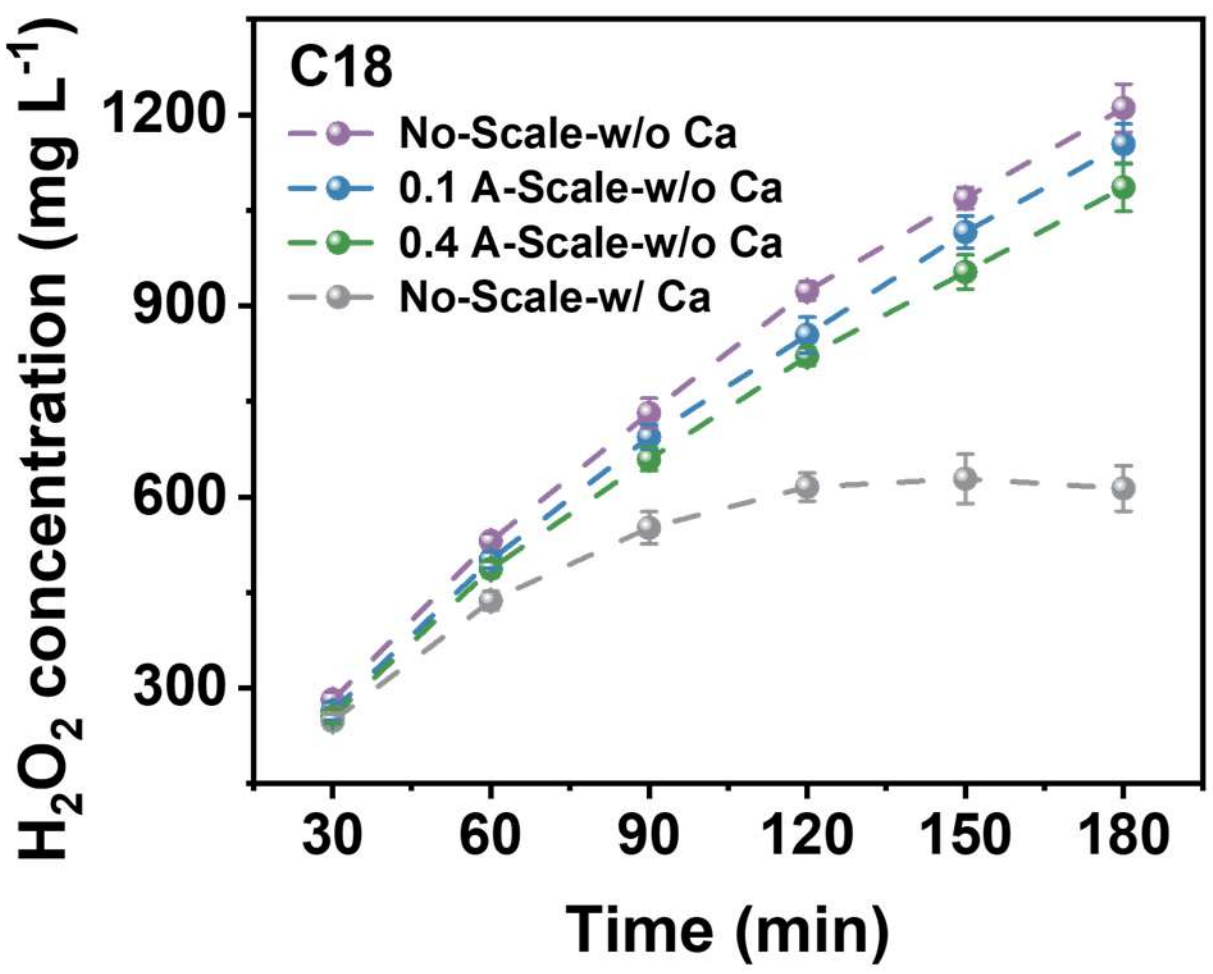

Supplementary Fig. 43. Time-dependent curves depicting H_2O_2 concentration of the C18 cathodes with varying degrees of scaling in 0.05 M Na_2SO_4 and no-scale cathode in 0.05 M Na_2SO_4 and 200 mg L^{-1} Ca^{2+}. Reaction conditions: a current of 0.1 A, a rotation speed of 600 rpm, electrolysis time of 3 h, and an initial pH of 7.0 ± 0.1. The data is presented as mean ± s.d. (n = 3). Source data for Supplementary Fig. 43 are provided as a Source Data file.

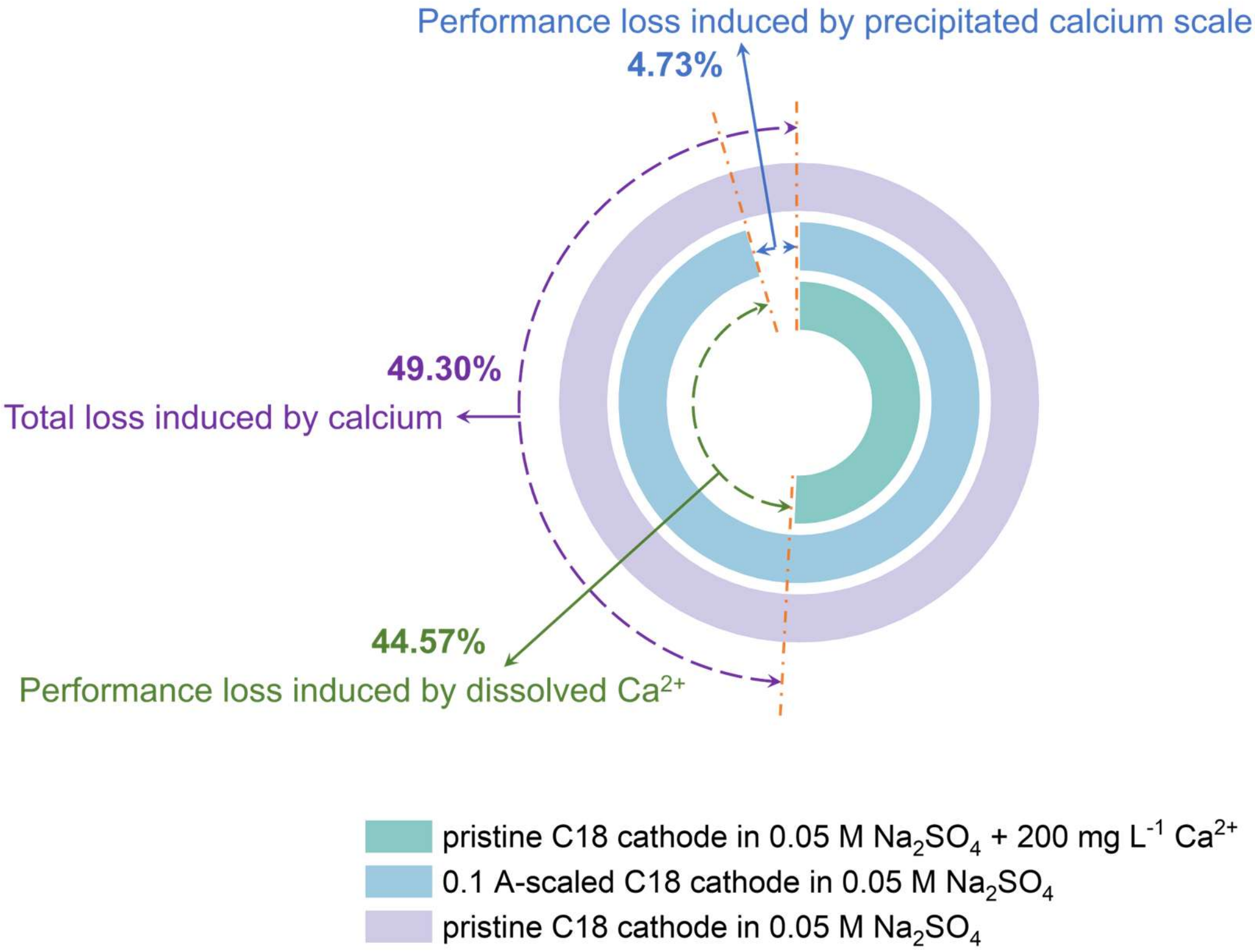

Supplementary Fig. 44. Decoupling analysis of the inhibitory effects of dissolved Ca^{2+} and precipitated calcium scale on H_2O_2 electrosynthesis performance for C18. Source data for Supplementary Fig. 44 are provided as a Source Data file.

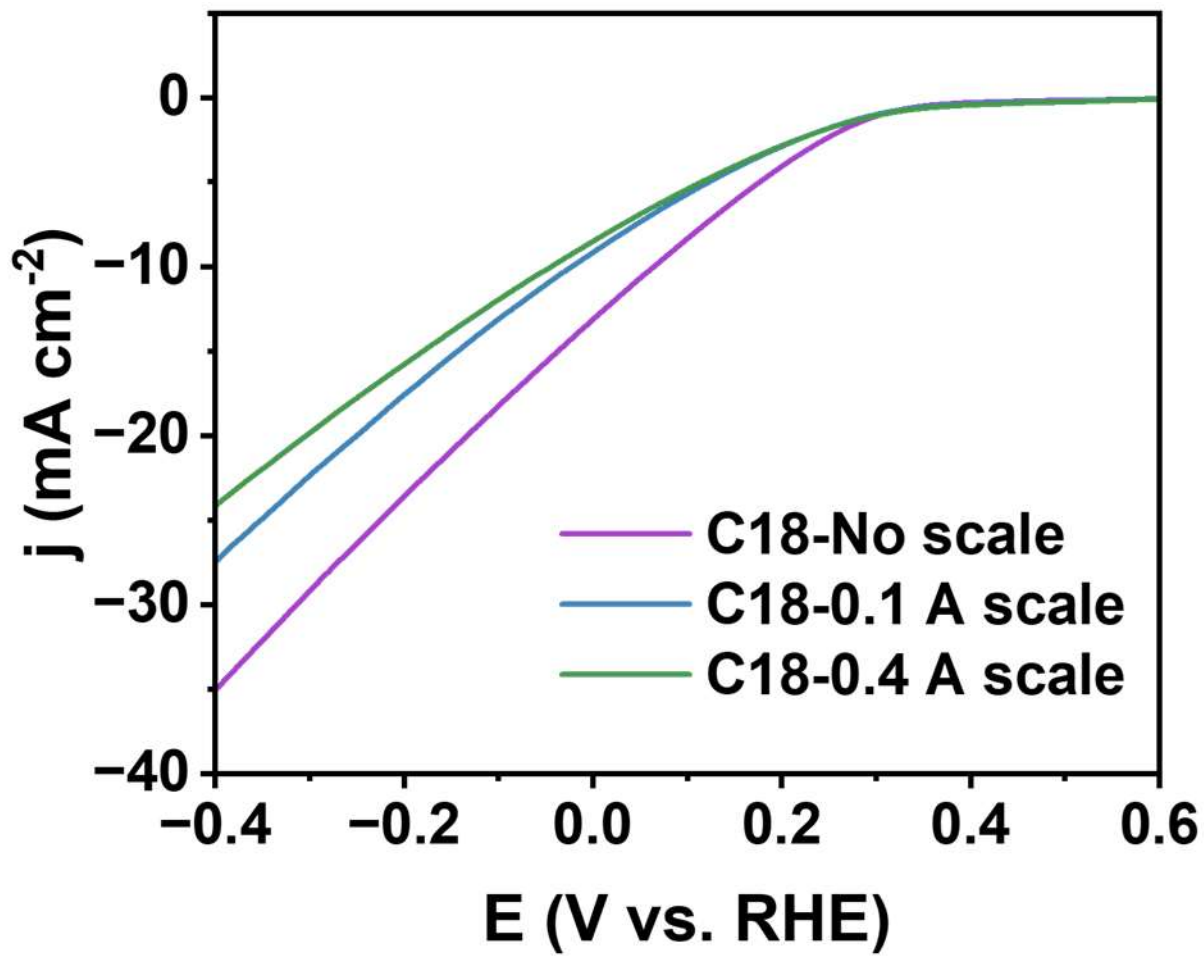

Supplementary Fig. 45. LSV curves of the C18 cathodes with varying degrees of scaling in O_2-saturated 0.1 M Na_2SO_4 electrolyte. All the potentials have not been iR-corrected. Source data for Supplementary Fig. 45 are provided as a Source Data file.

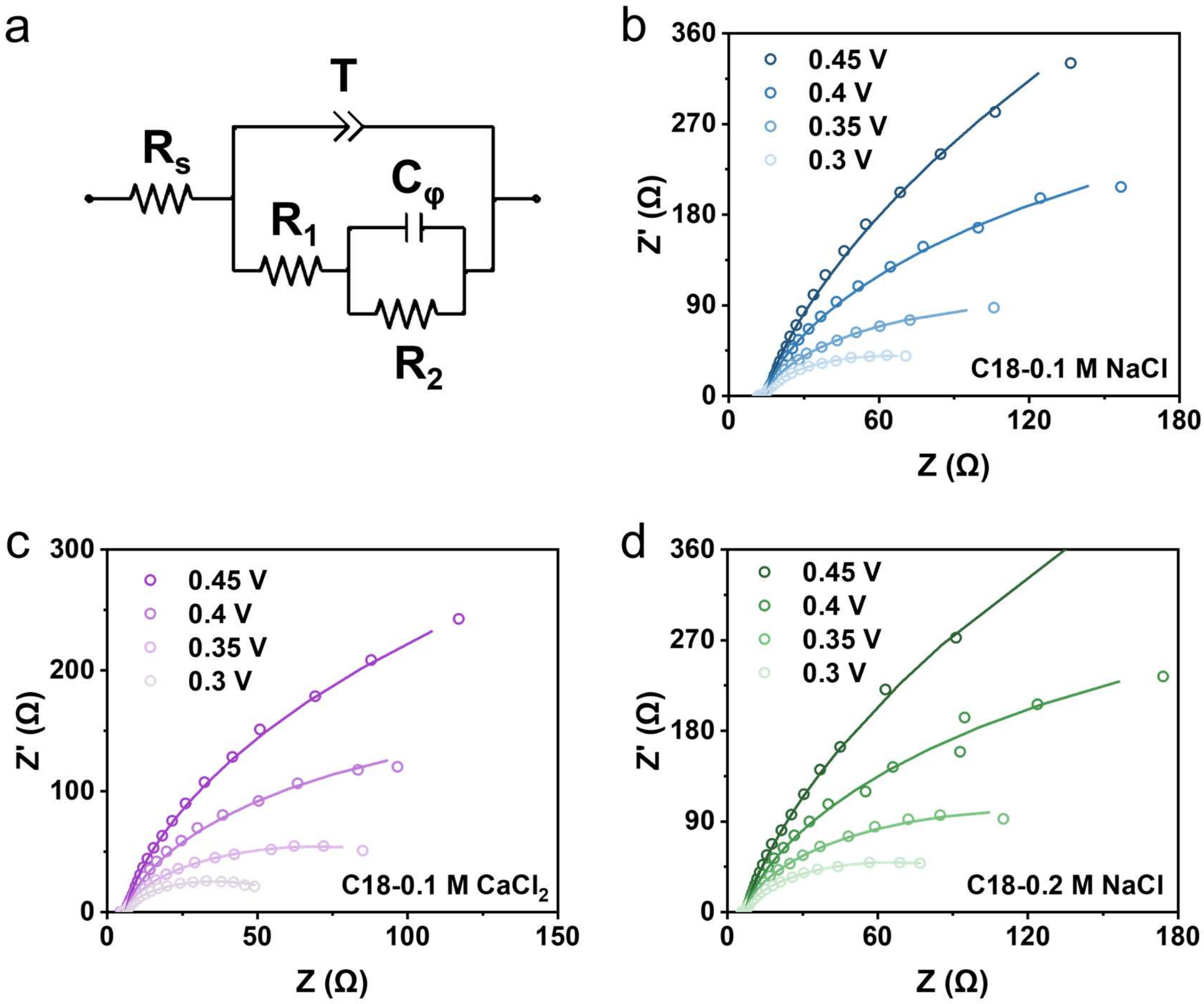

Supplementary Fig. 46. (a) The equivalent circuit. R_s is the solution resistance. T represents the double layer capacitance. R_1 is the charge transfer resistance, associated with the main electrochemical reaction. R_2 and C_φ represent the hydrogen adsorption resistance and pseudo-capacitance, respectively. The EIS Nyquist plots of the C18 cathode measured at different potentials in N_2-saturated (b) 0.1 M NaCl, (c) 0.1 M $CaCl_2$ and (d) 0.2 M NaCl electrolytes at an initial H_2O_2 concentration of 10 mM. Solid lines are the fitting results according to the equivalent circuit. All the potentials have not been iR-corrected. Source data for Supplementary Fig. 46 are provided as a Source Data file.

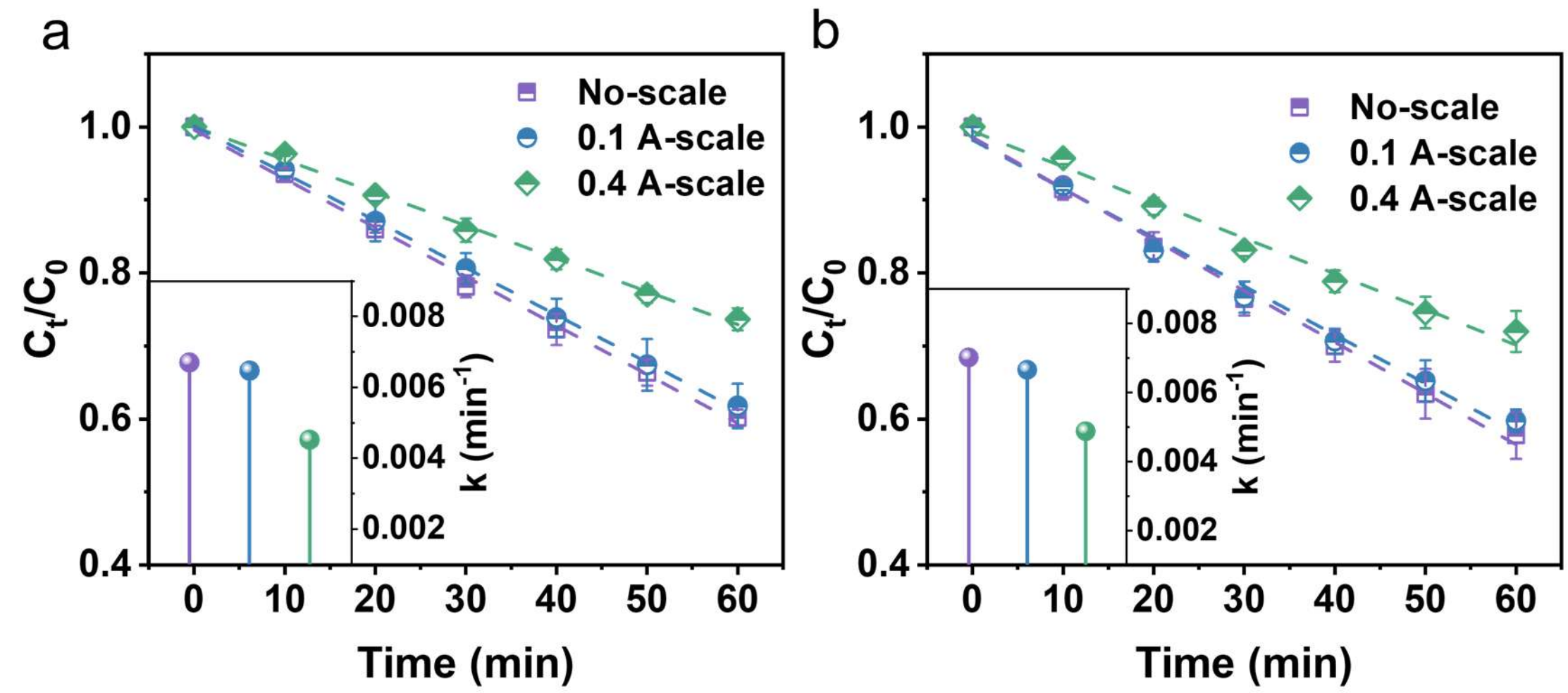

Supplementary Fig. 47. H_2O_2 decomposition curves and H_2O_2 decomposition rate constant k of C18 cathodes with varying degrees of scaling at (a) 0.2 A and (a) 0.4 A with an initial H_2O_2 concentration of 1200 mg L^{-1}. Reaction conditions: [Na_2SO_4] = 0.05 M, a rotation speed of 600 rpm, an initial pH of 7.0 ± 0.1, and electrolysis of 1 h. The data is presented as mean ± s.d. (n = 3). Source data for Supplementary Fig. 47 are provided as a Source Data file.

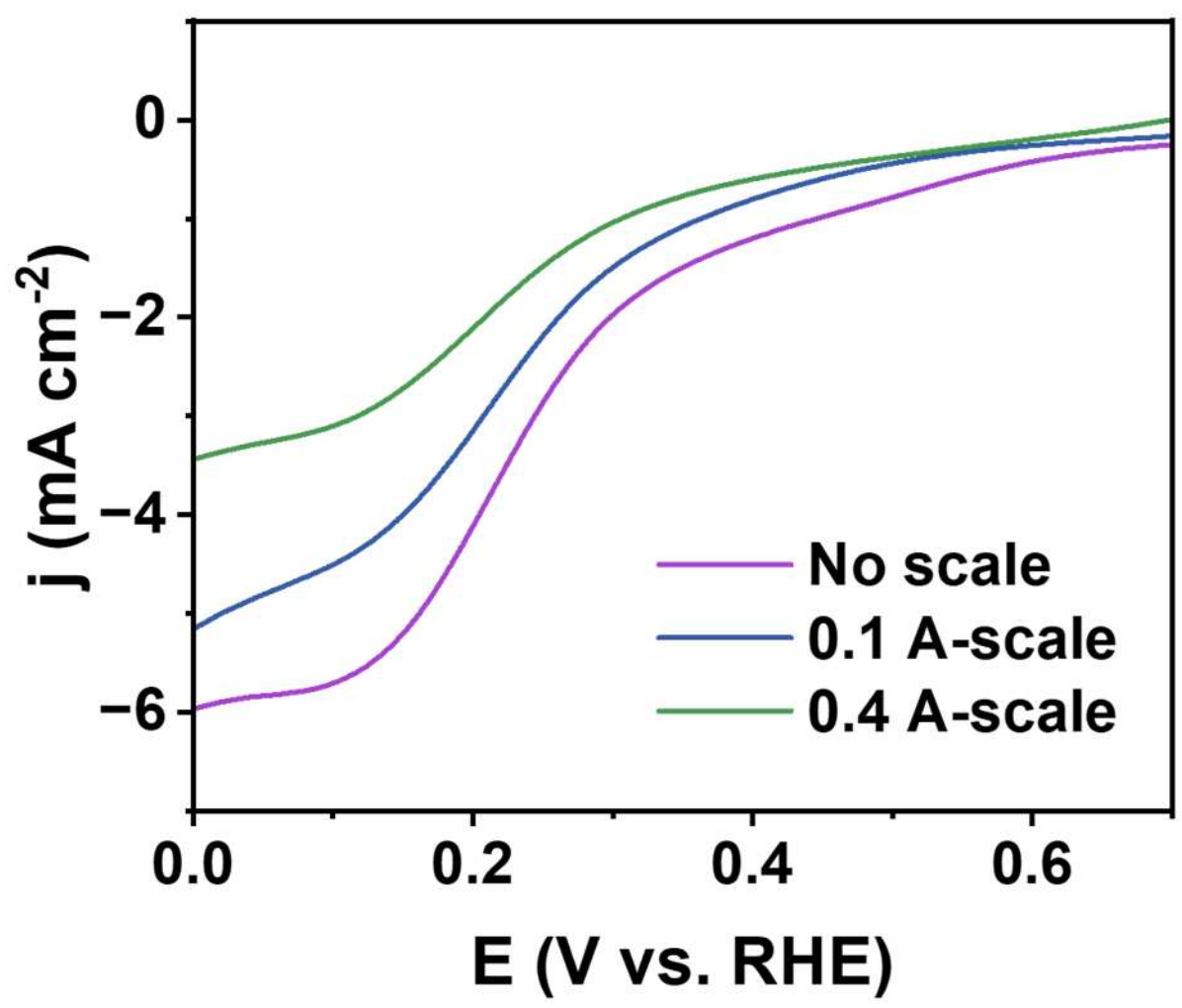

Supplementary Fig. 48. LSV curves of H_2O_2 reduction for C18 cathodes with varying degrees of scaling in N_2-saturated 0.1 M Na_2SO_4 solution containing 10 mM H_2O_2. All the potentials have not been iR-corrected. Source data for Supplementary Fig. 48 are provided as a Source Data file.

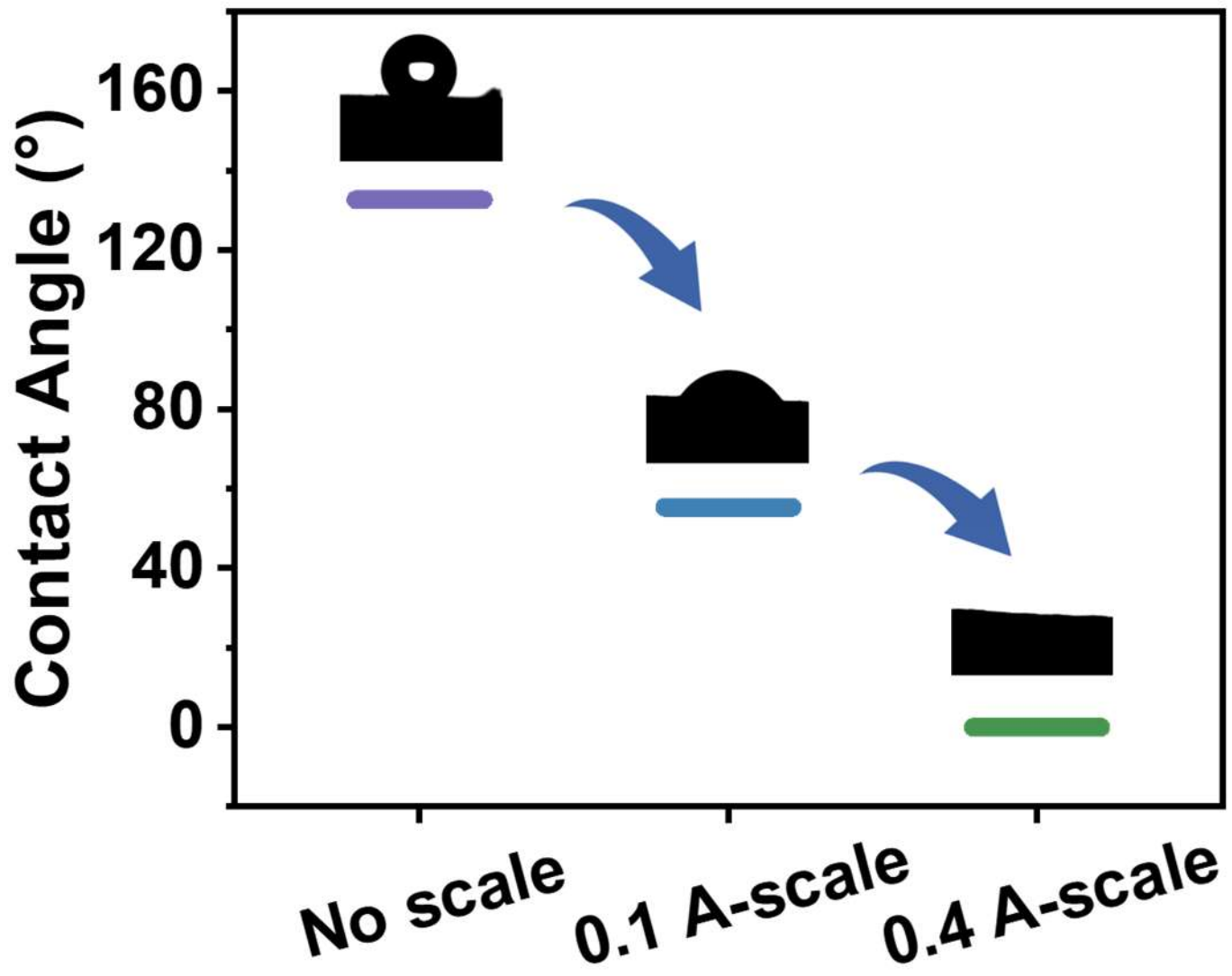

Supplementary Fig. 49. Contact angle measurements of the C18 cathodes with varying degrees of scaling. Reaction conditions: $[Na_2SO_4]$ = 0.05 M, $[Ca^{2+}]_0$ = 200 mg L^{-1}, a rotation speed of 600 rpm, an initial pH of 7.0 ± 0.1 and electrolysis of 3 h. Source data for Supplementary Fig. 49 are provided as a Source Data file.

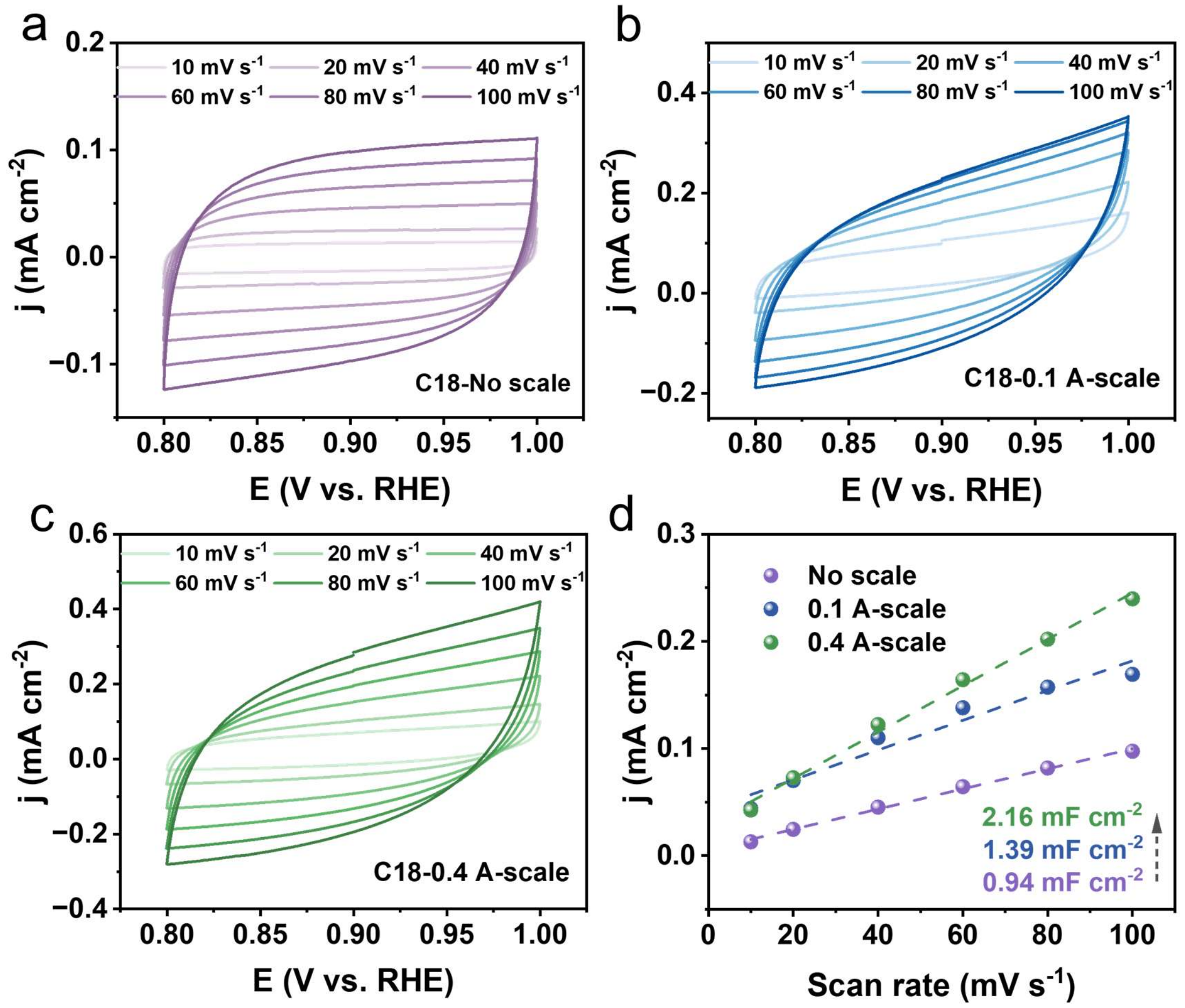

Supplementary Fig. 50. CV curves of the (a) No-scale, (b) 0.1 A-scale and (c) 0.4 A-scale C18 cathodes in N_2-saturated 0.1 M Na_2SO_4 electrolyte for ECSA measurements. (d) Calculated ECSA of C18 cathodes with varying degrees of scaling. All the potentials have not been iR-corrected. Source data for Supplementary Fig. 50 are provided as a Source Data file.

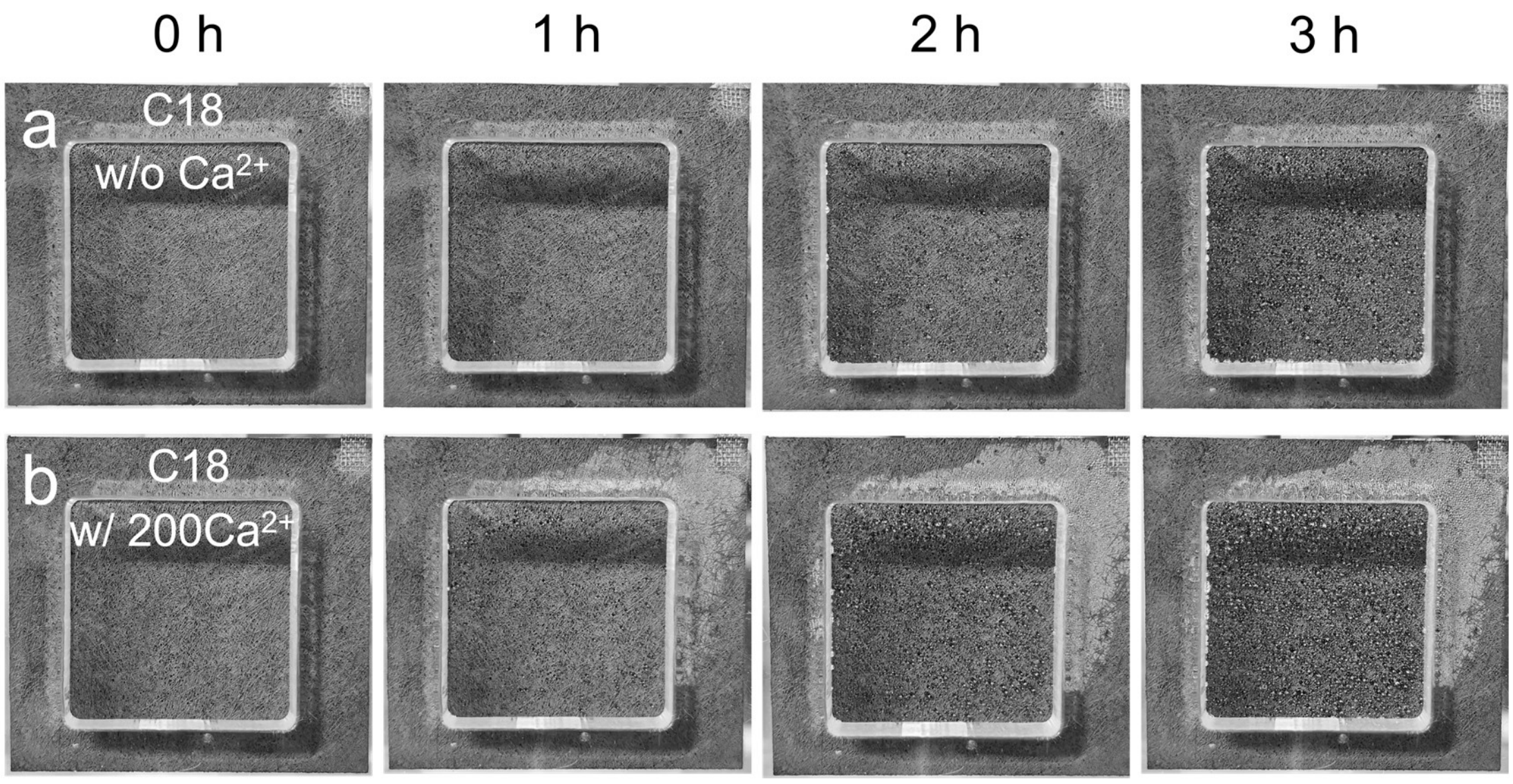

Supplementary Fig. 51. Optical images of the C18-GDL water flooding over time in the (a) absence and (b) presence of 200 mg L^{-1} Ca^{2+} at 0.1 A.

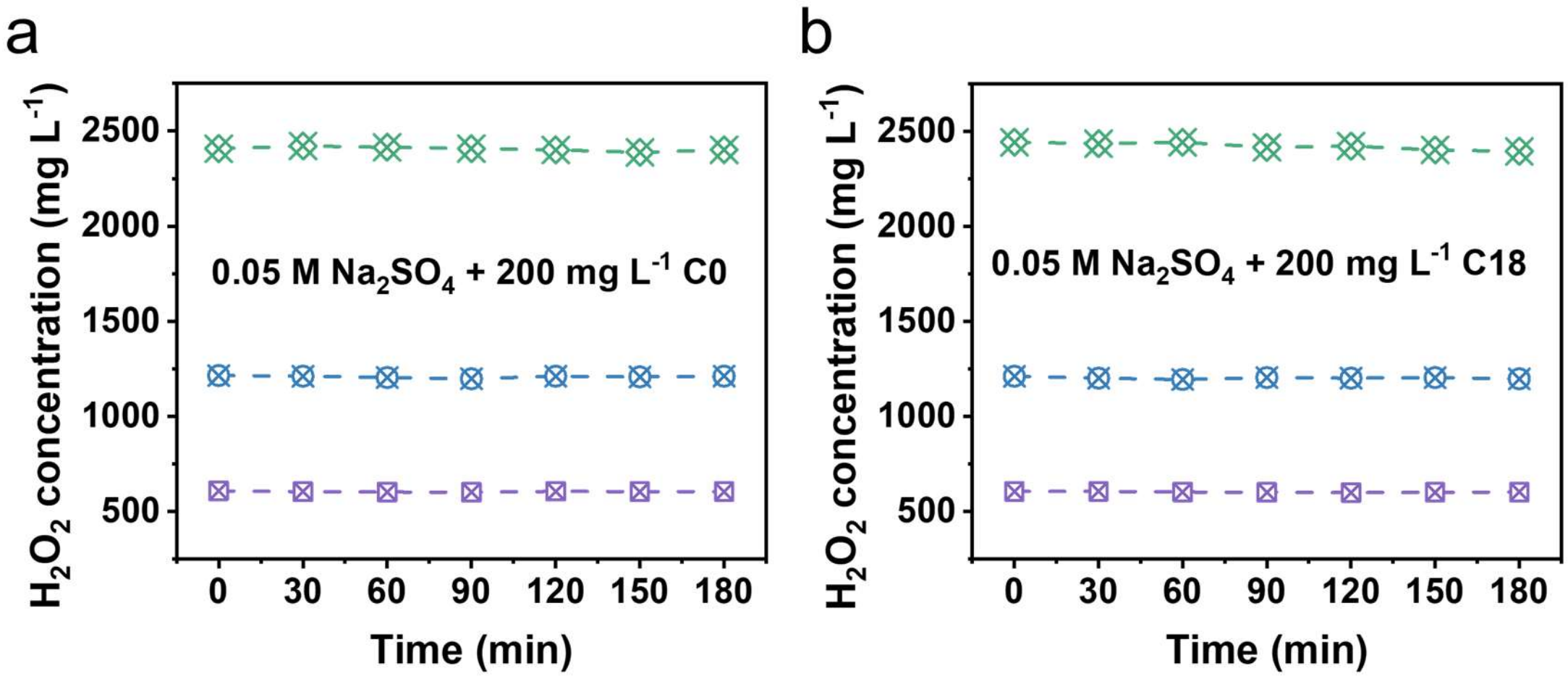

Supplementary Fig. 52. The self-decomposition curves of H_2O_2 in 0.05 M Na_2SO_4 solutions with 200 mg L^{-1} (a) C0 and (b) C18 catalysts under varying initial H_2O_2 concentrations. Reaction conditions: a current of 0 A, a rotation speed of 600 rpm, reaction time of 3 h, and an initial pH of 7.0 ± 0.1. Source data for Supplementary Fig. 52 are provided as a Source Data file.

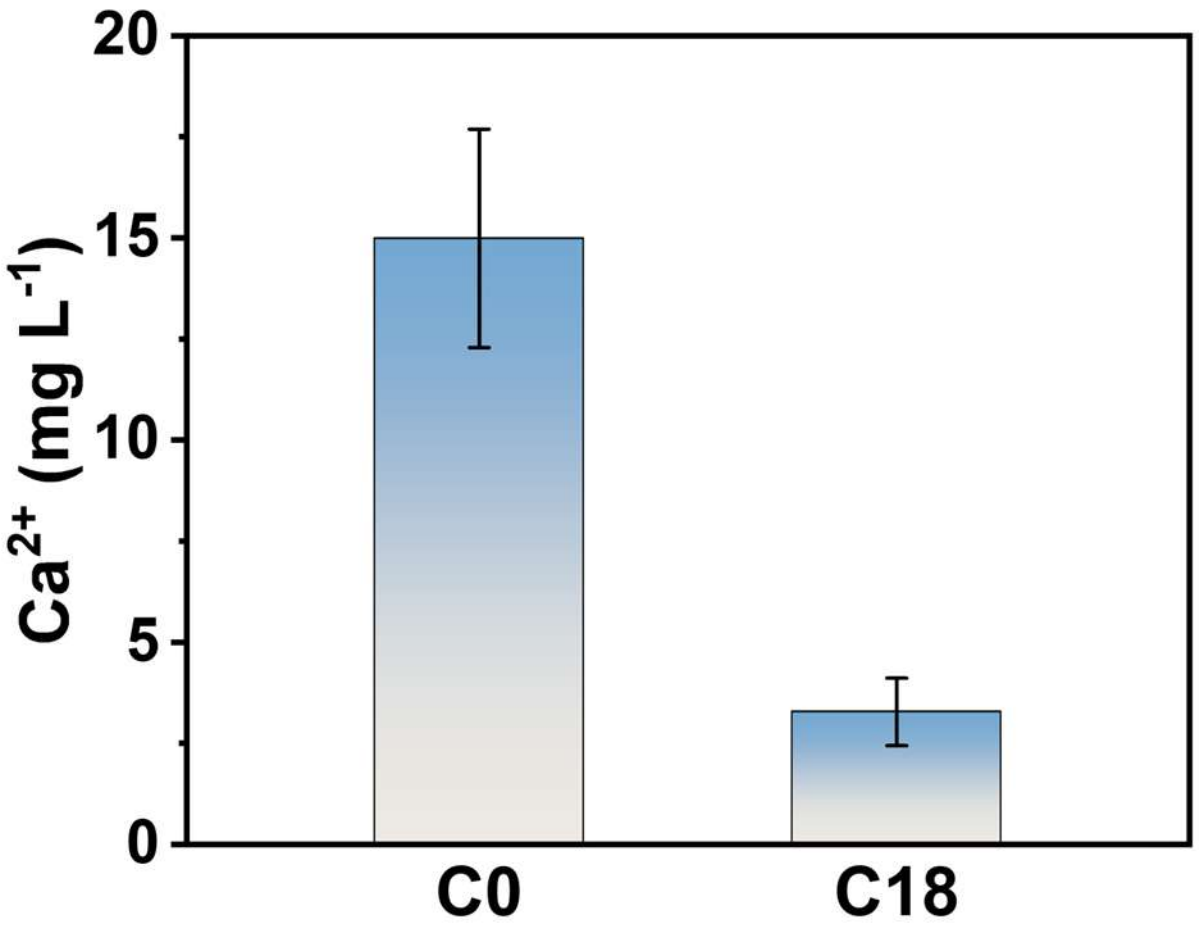

Supplementary Fig. 53 The electric-field-driven Ca^{2+} adsorption concentration detected by ICP-OES for the C0 and C18 cathode. The data is presented as mean ± s.d. (n = 3). Source data for Supplementary Fig. 53 are provided as a Source Data file.

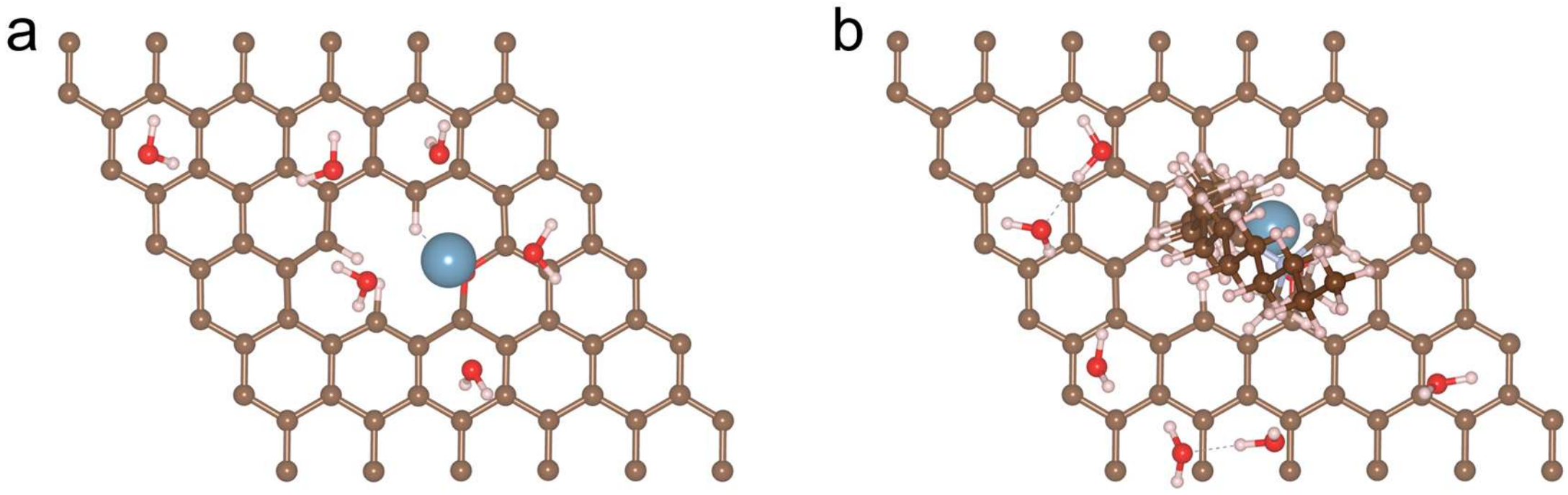

Supplementary Fig. 54. The adsorption planar models for Ca^{2+} on the surfaces of (a) C0 and (b) C18 cathodes. The brown, red, pink, and blue spheres represent C, O, H and Ca atoms, respectively.

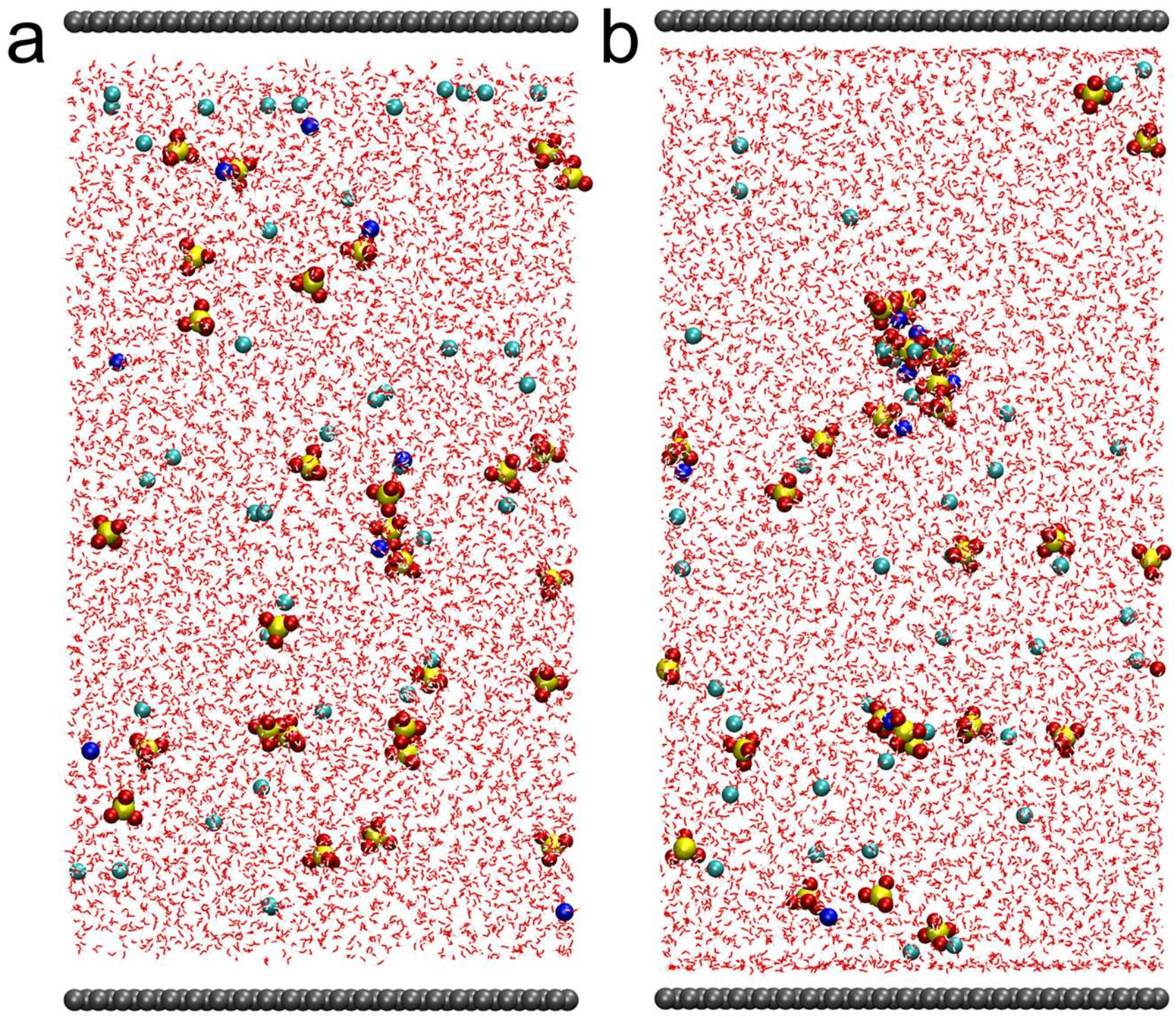

Supplementary Fig. 55. Snapshot of the C0 system at (a) 0 ns and (b) 20 ns in MD simulation. Colors: C in dark gray, H in light gray, O in red, S in yellow, Na in cyan and Ca in blue.

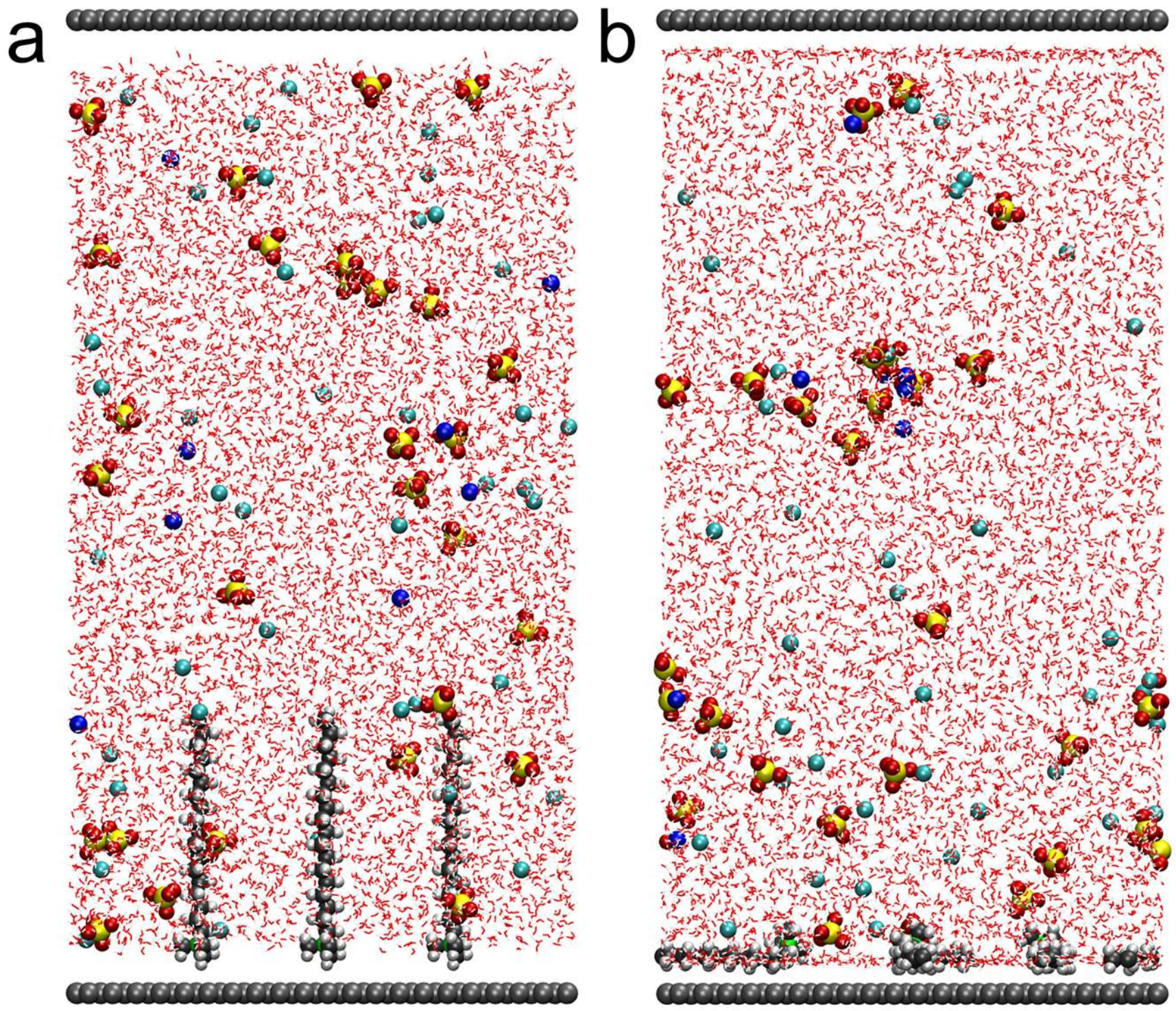

Supplementary Fig. 56. Snapshot of the C0 system at (a) 0 ns and (b) 20 ns in MD simulation. Colors: C in dark gray, H in light gray, O in red, S in yellow, N in green, Na in cyan and Ca in blue.

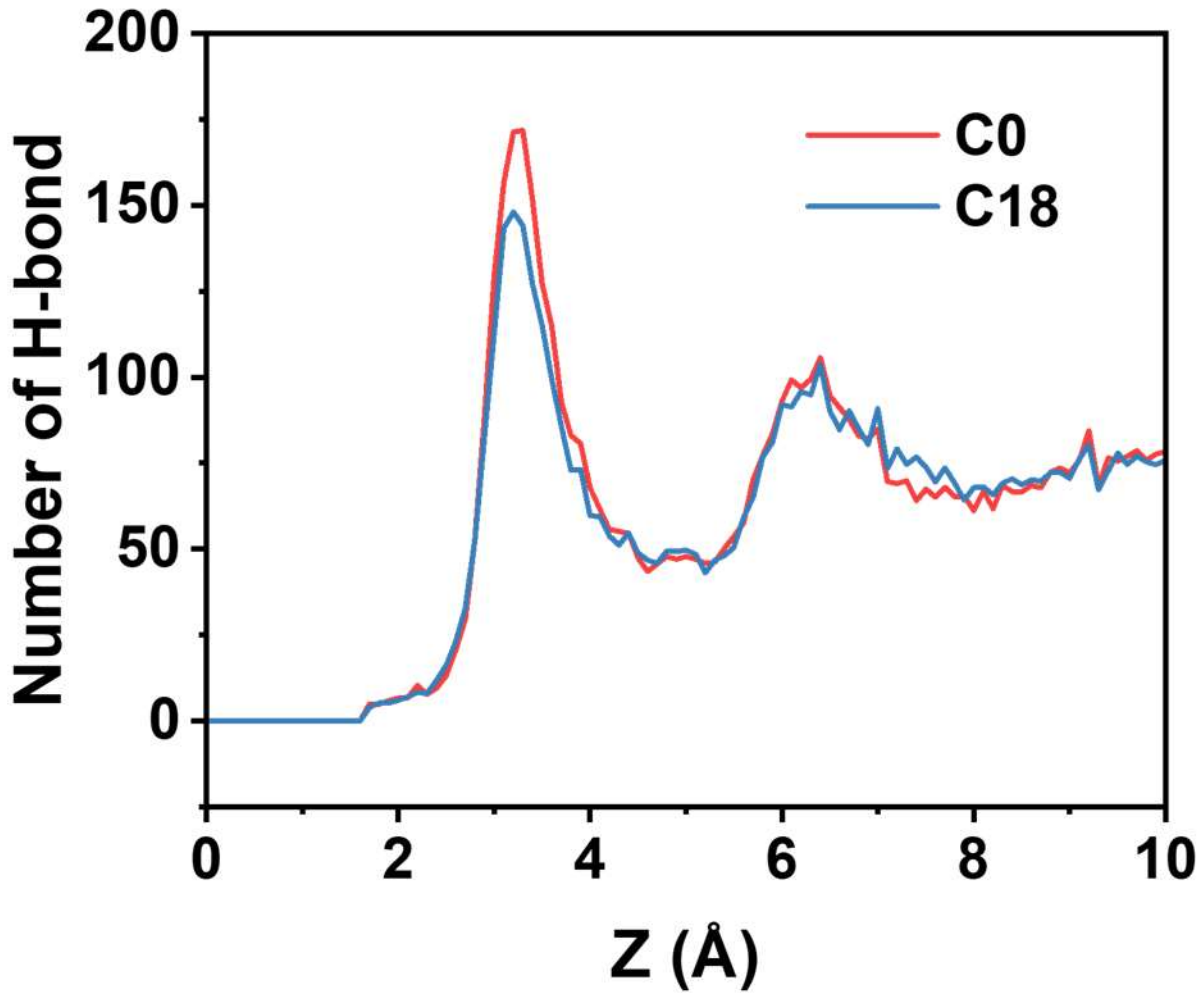

Supplementary Fig. 57. Radial density distributions of H-bond for C0 and C18 systems in MD simulation. Source data for Supplementary Fig. 57 are provided as a Source Data file.

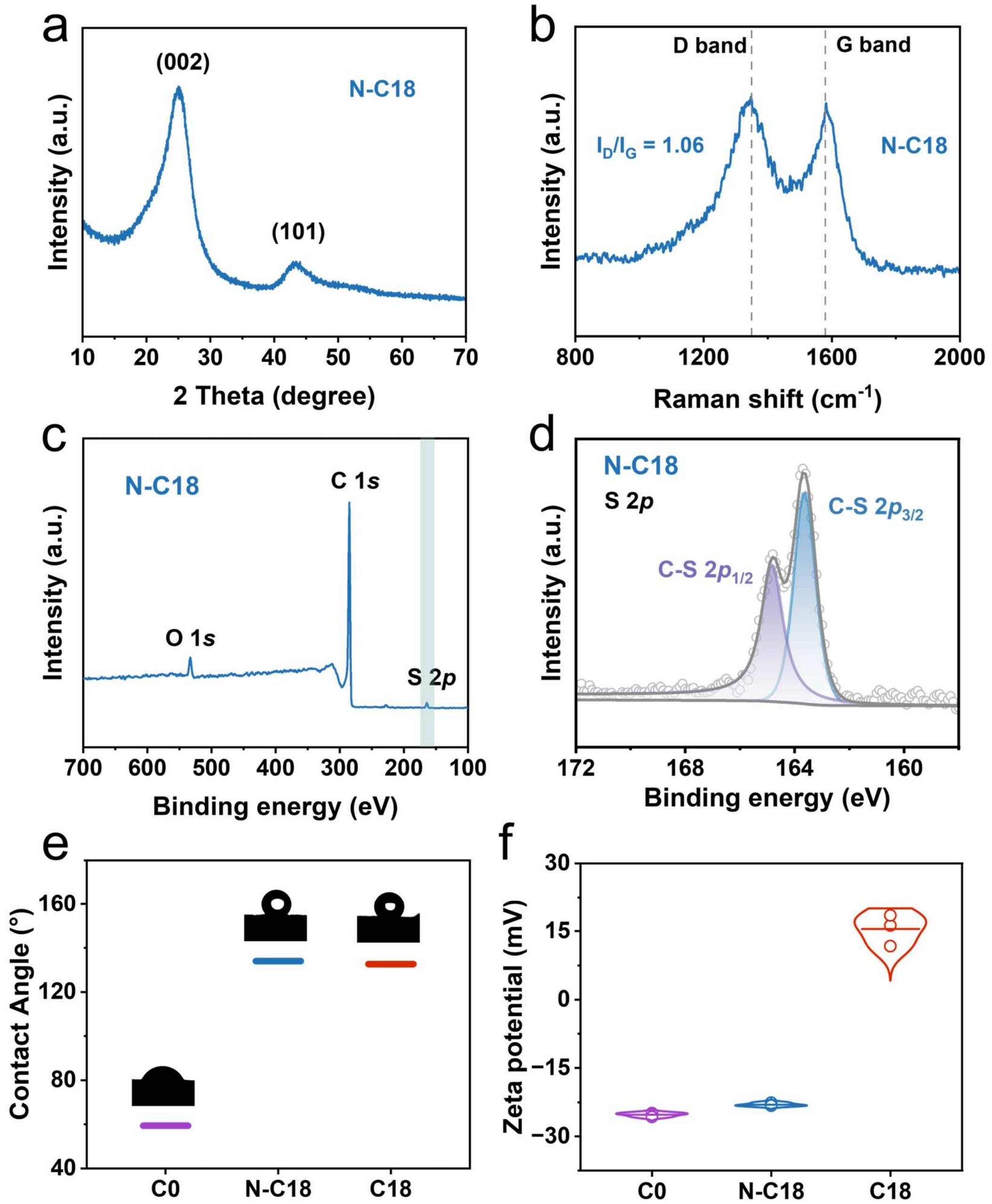

Supplementary Fig. 58. (a) XRD pattern and (b) Raman spectra of the N-C18 catalyst. (c) XPS spectra and (d) high-resolution S 2*p* spectra of N-C18. (e) Contact angle and (f) Zeta potential measurements of C0, N-C18, and C18. Source data for Supplementary Fig. 58 are provided as a Source Data file.

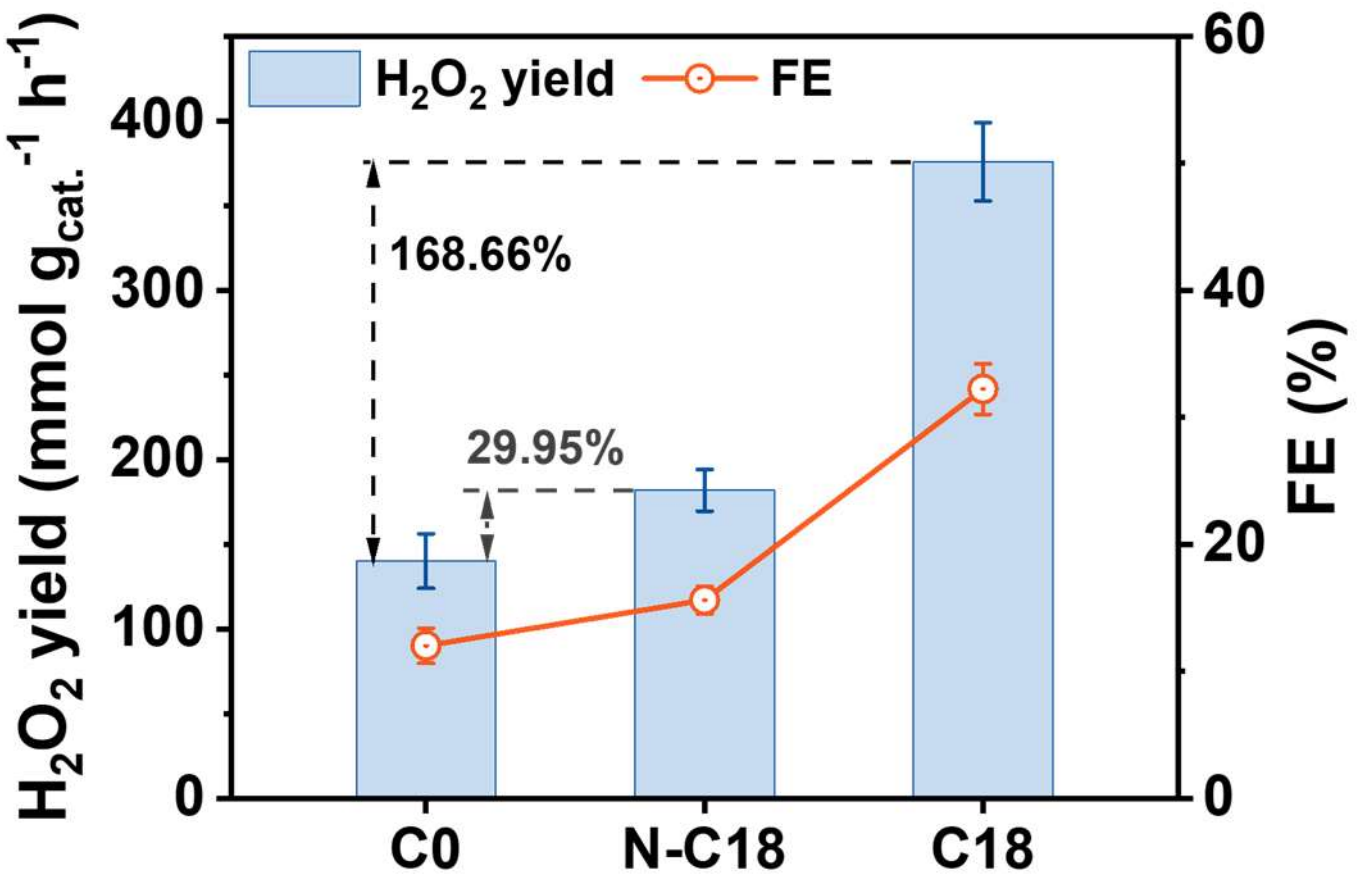

Supplementary Fig. 59. H_2O_2 electrosynthesis performance of the C0, N-C18, and C18 cathodes in the presence of Ca^{2+}. Reaction conditions: $[Na_2SO_4]$ = 0.05 M, $[Ca^{2+}]_0$ = 200 mg L^{-1}, a current of 0.1 A, a rotation speed of 600 rpm, an initial pH of 7.0 ± 0.1 and electrolysis of 3 h. The data is presented as mean ± s.d. (n = 3). Source data for Supplementary Fig. 59 are provided as a Source Data file.

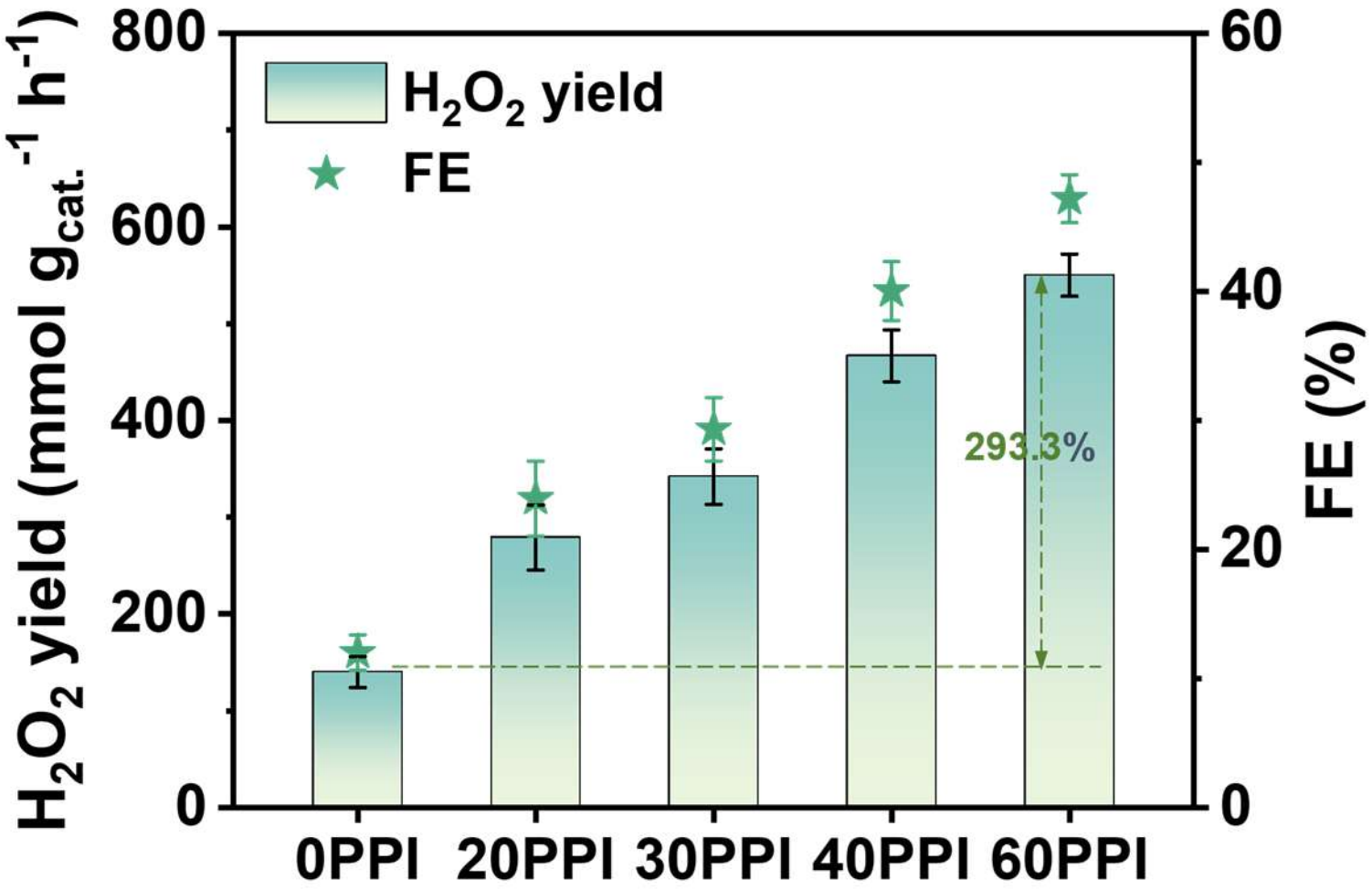

Supplementary Fig. 60. H_2O_2 yield and FE of C0 in PUS-fenced cathode systems with varying pore sizes. Reaction conditions: [Na_2SO_4] = 0.05 M, [Ca^{2+}]$_0$ = 200 mg L^{-1}, a current of 0.1 A, a rotation speed of 600 rpm, an initial pH of 7.0 ± 0.1 and electrolysis of 3 h. The data is presented as mean ± s.d. (n = 3). Source data for Supplementary Fig. 60 are provided as a Source Data file.

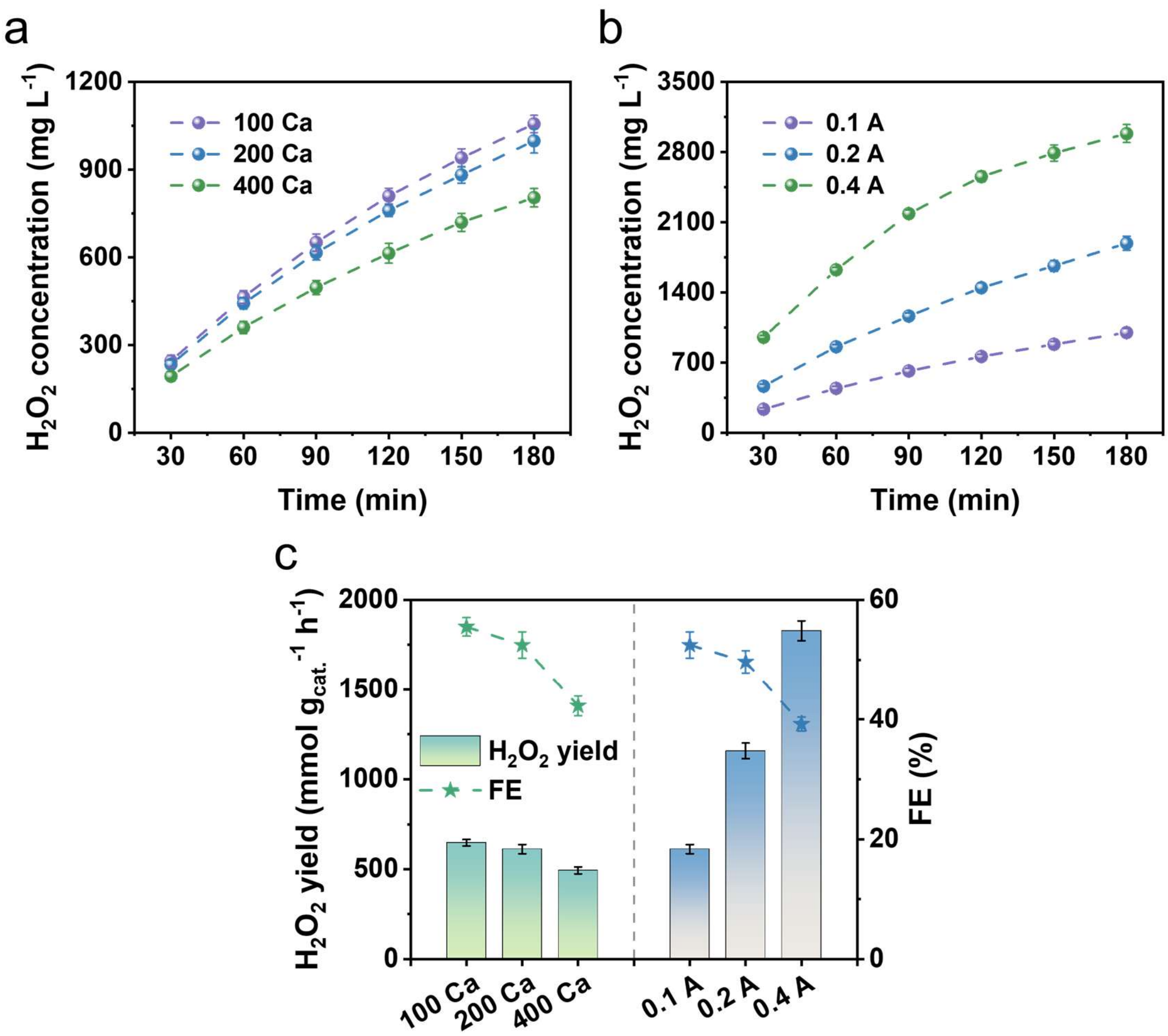

Supplementary Fig. 61. Time-dependent curves depicting H_2O_2 concentration of the 60PPI-PUS-fenced C18 cathode under different (a) Ca^{2+} concentrations and (b) currents. (c) H_2O_2 yield and FE of the 60PPI-PUS-fenced C18 cathode under different Ca^{2+} concentrations and currents. The data is presented as mean ± s.d. (n = 3). Source data for Supplementary Fig. 61 are provided as a Source Data file.

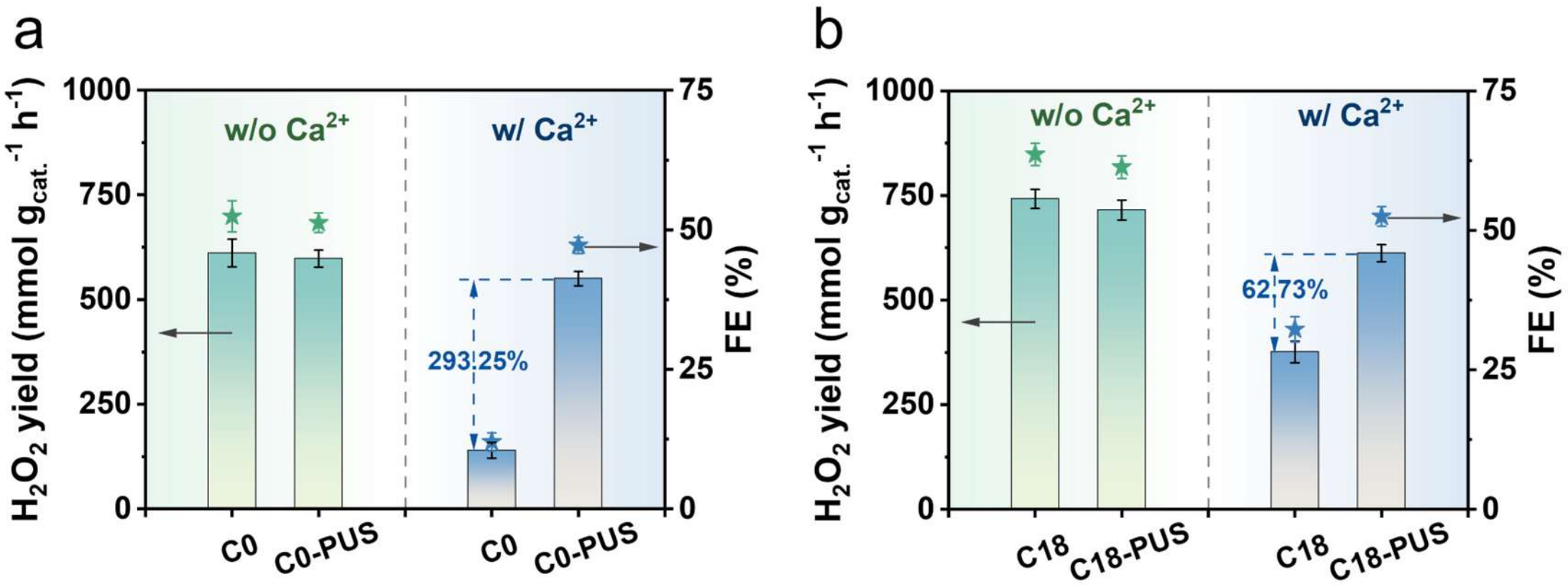

Supplementary Fig. 62. (a) H_2O_2 electrosynthesis performance of the C0 and C0-PUS cathodes in the absence (w/o) or presence (w/) of 200 mg L^{-1} Ca^{2+}. (a) H_2O_2 electrosynthesis performance of the C18 and C18-PUS cathodes in the absence (w/o) or presence (w/) of 200 mg L^{-1} Ca^{2+}. Reaction conditions: [Na_2SO_4] = 0.05 M, a current of 0.1 A, a rotation speed of 600 rpm, an initial pH of 7.0 ± 0.1 and electrolysis of 3 h. The data is presented as mean ± s.d. (n = 3). Source data for Supplementary Fig. 62 are provided as a Source Data file.

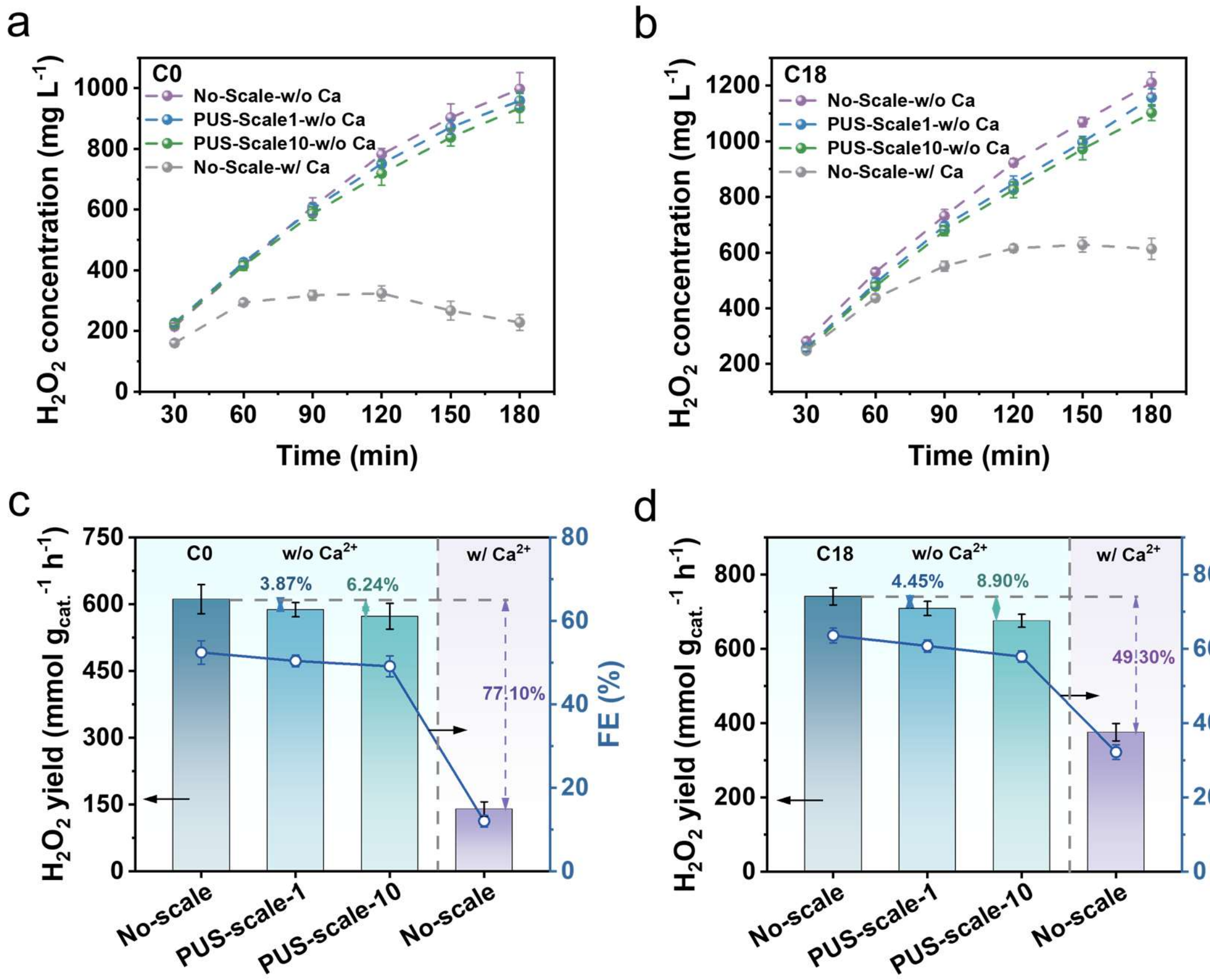

Supplementary Fig. 63. Time-dependent curves depicting H_2O_2 concentration of the (a) C0 and (b) C18 cathodes with 1 cycle and 10 cycles PUS-fenced scaling in 0.05 M Na_2SO_4 and no-scale cathode in 0.05 M Na_2SO_4 and 200 mg L^{-1} Ca^{2+}. H_2O_2 yield and FE of the (c) C0 and (d) C18 cathodes with 1 cycle and 10 cycles PUS-fenced scaling in 0.05 M Na_2SO_4 and no-scale cathode in 0.05 M Na_2SO_4 and 200 mg L^{-1} Ca^{2+}. Reaction conditions: a current of 0.1 A, a rotation speed of 600 rpm, electrolysis time of 3 h, and an initial pH of 7.0 ± 0.1. The data is presented as mean ± s.d. (n = 3). Source data for Supplementary Fig. 63 are provided as a Source Data file.

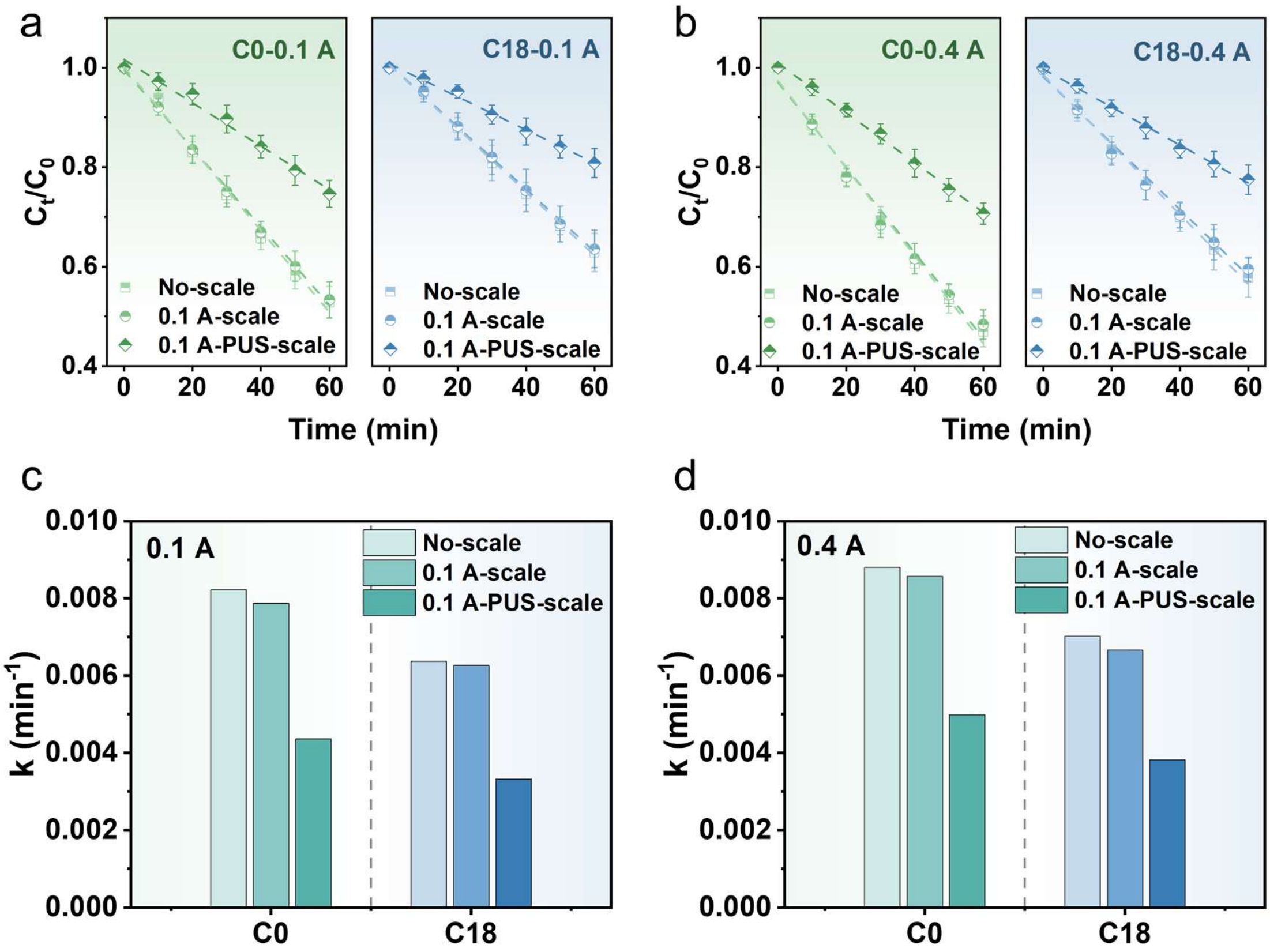

Supplementary Fig. 64. H_2O_2 decomposition curves at (a) 0.1 A and (b) 0.4 A of the C0 and C18 cathodes with varying degrees of scaling at an initial H_2O_2 concentration of 1200 mg L^{-1}. H_2O_2 decomposition rate constant k at (c) 0.1 A and (d) 0.4 A of the C0 and C18 cathodes with varying degrees of scaling at an initial H_2O_2 concentration of 1200 mg L^{-1}. Reaction conditions: [Na_2SO_4] = 0.05 M, a rotation speed of 600 rpm, an initial pH of 7.0 ± 0.1, and electrolysis of 1 h. The data is presented as mean ± s.d. (n = 3). Source data for Supplementary Fig. 64 are provided as a Source Data file.

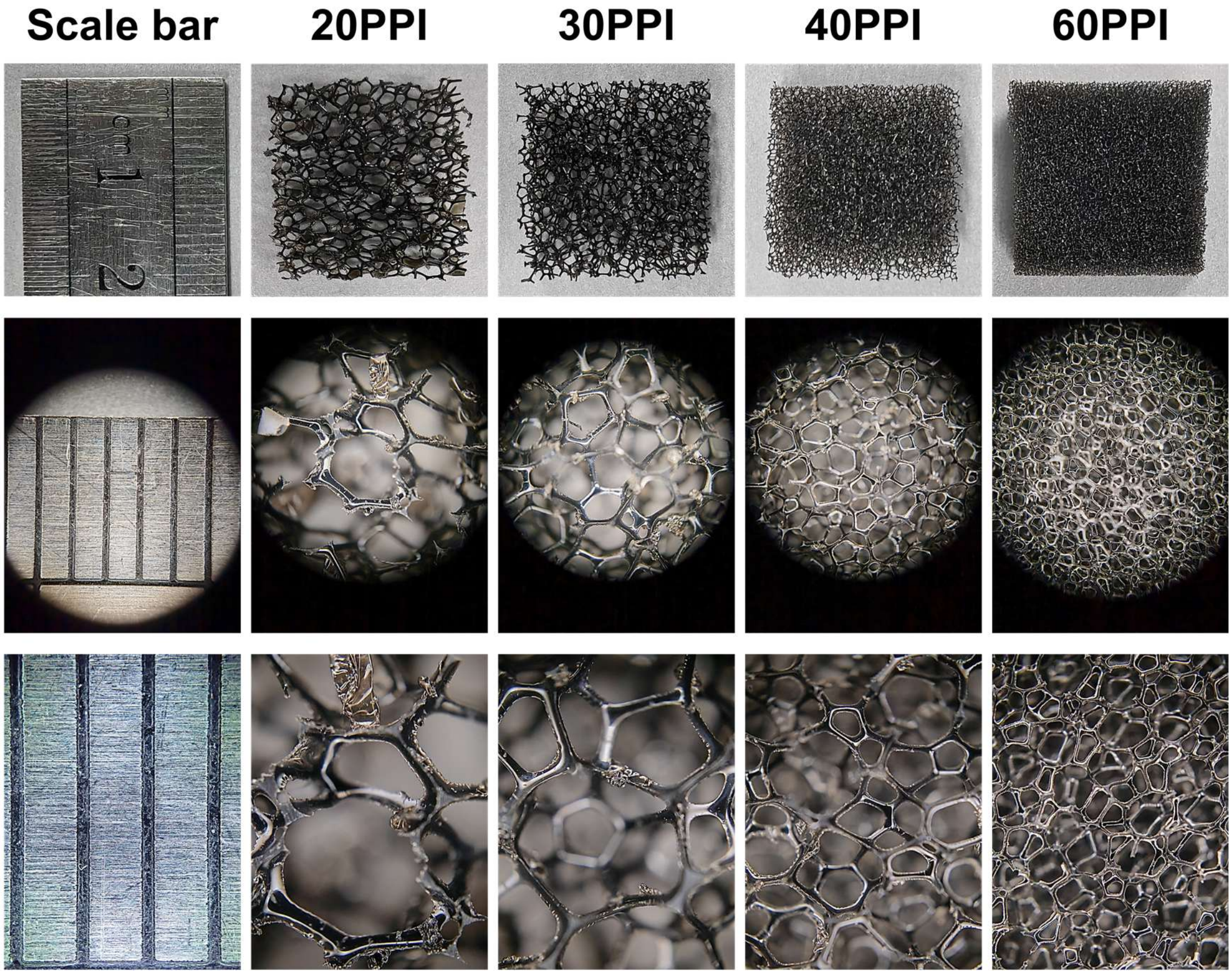

Supplementary Fig. 65. Optical images of PUS with varying pore sizes.

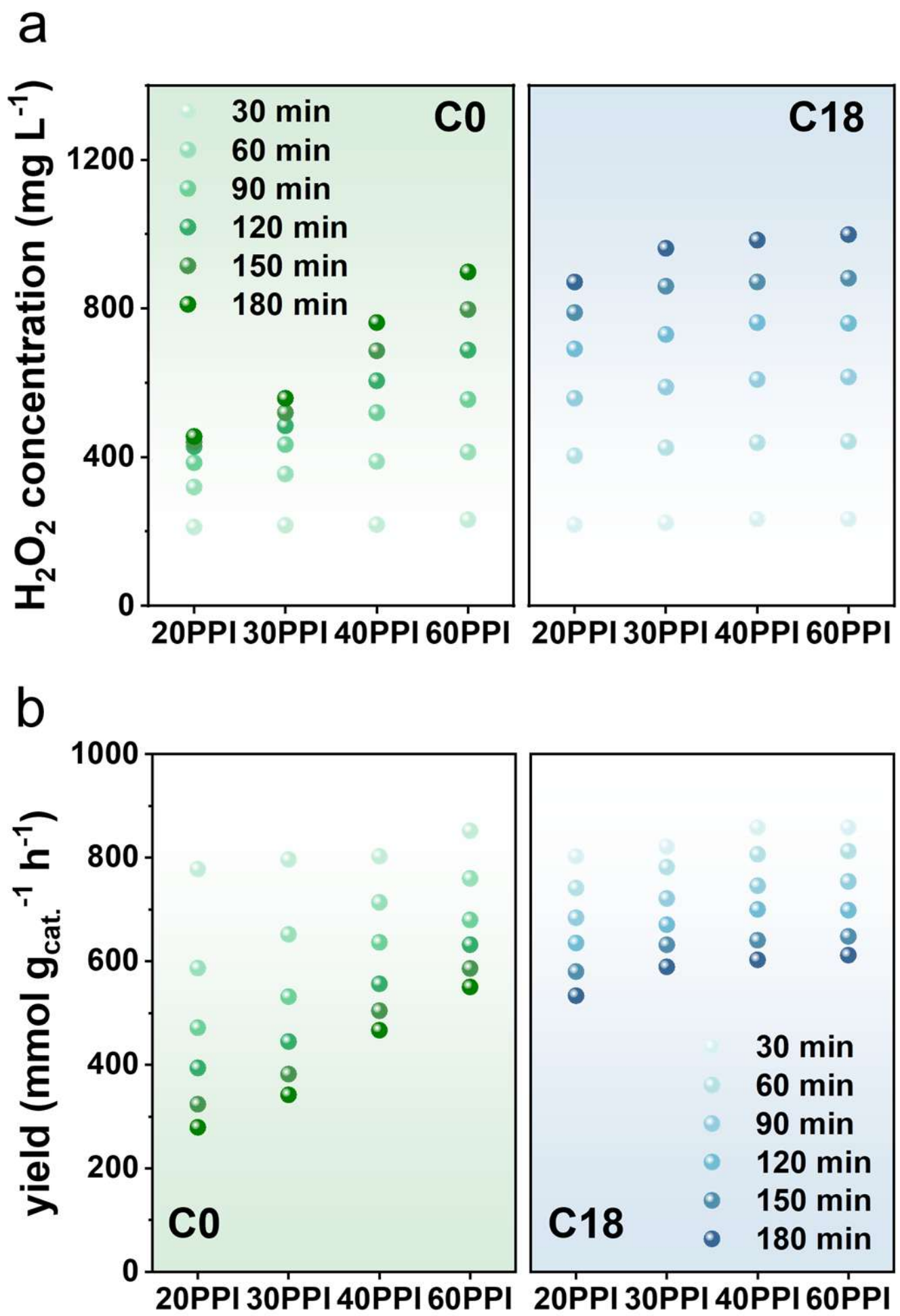

Supplementary Fig. 66. (a) H_2O_2 concentration and (b) yield versus time in PUS-fenced cathode systems with varying pore sizes for C0 and C18. Reaction conditions: [Na_2SO_4] = 0.05 M, [Ca^{2+}]$_0$ = 200 mg L^{-1}, a rotation speed of 600 rpm, an initial pH of 7.0 ± 0.1 and electrolysis of 3 h. Source data for Supplementary Fig. 66 are provided as a Source Data file.

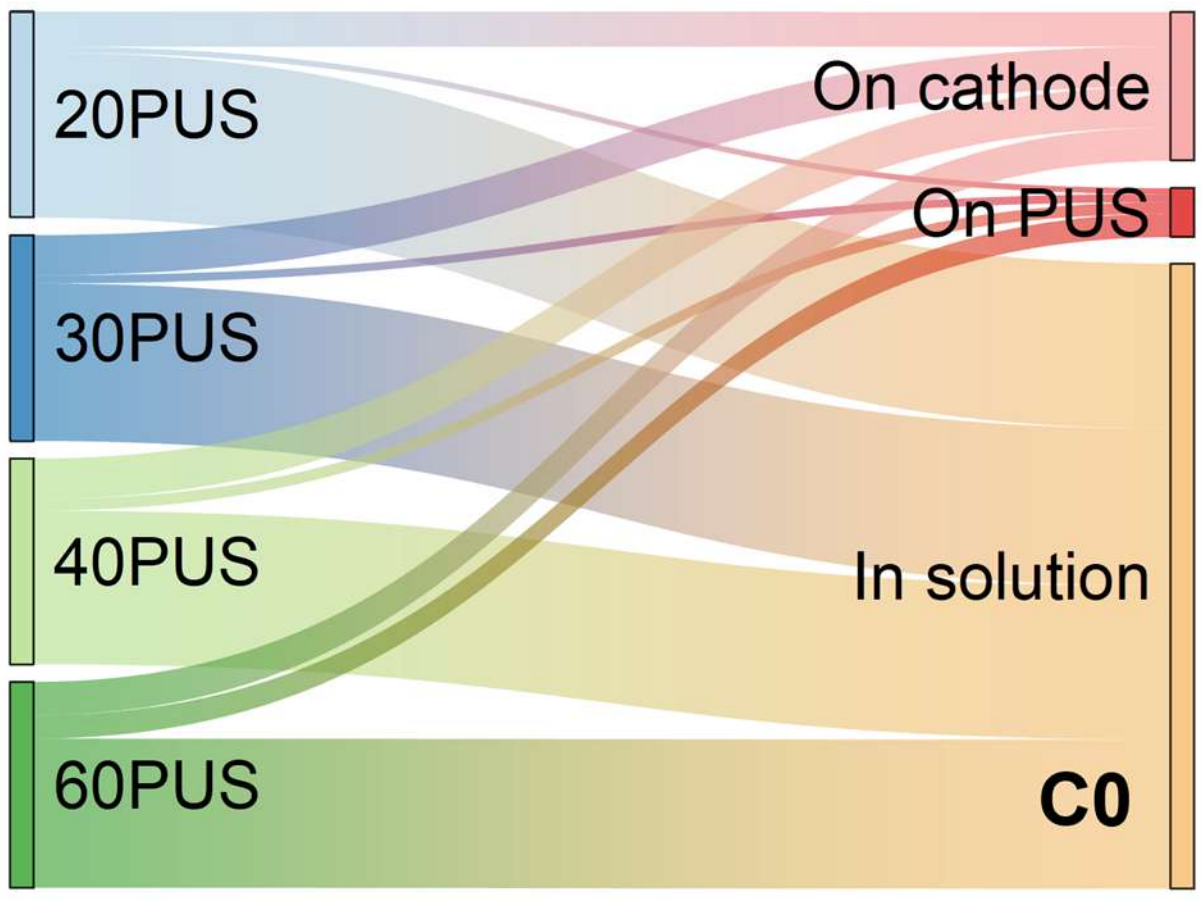

Supplementary Fig. 67. Mass balance of Ca in different PUS-fenced systems for C0. Source data for Supplementary Fig. 67 are provided as a Source Data file.

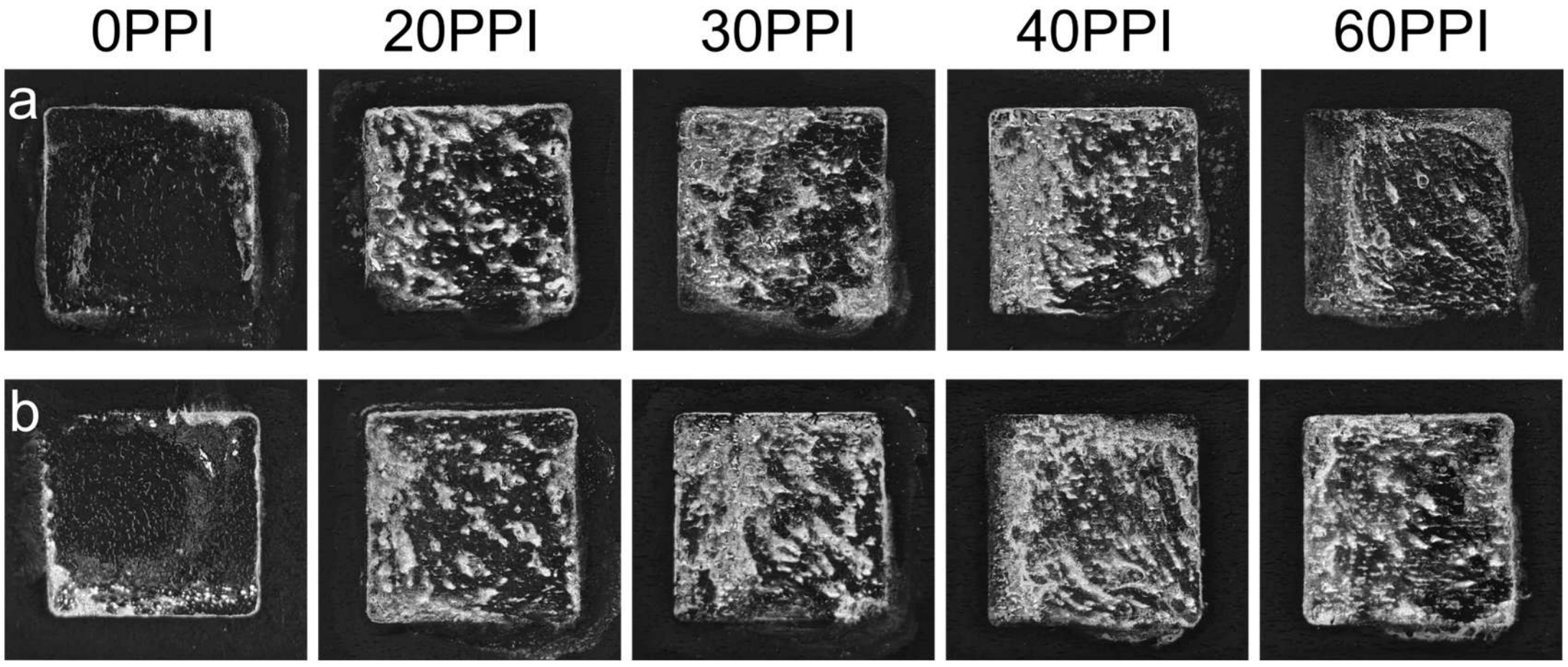

Supplementary Fig. 68. Optical images of (a) C0 and (b) C18 cathodes after experiments in different PUS-fenced systems under an initial concentration of 200 mg L^{-1} Ca^{2+} at 0.1 A.

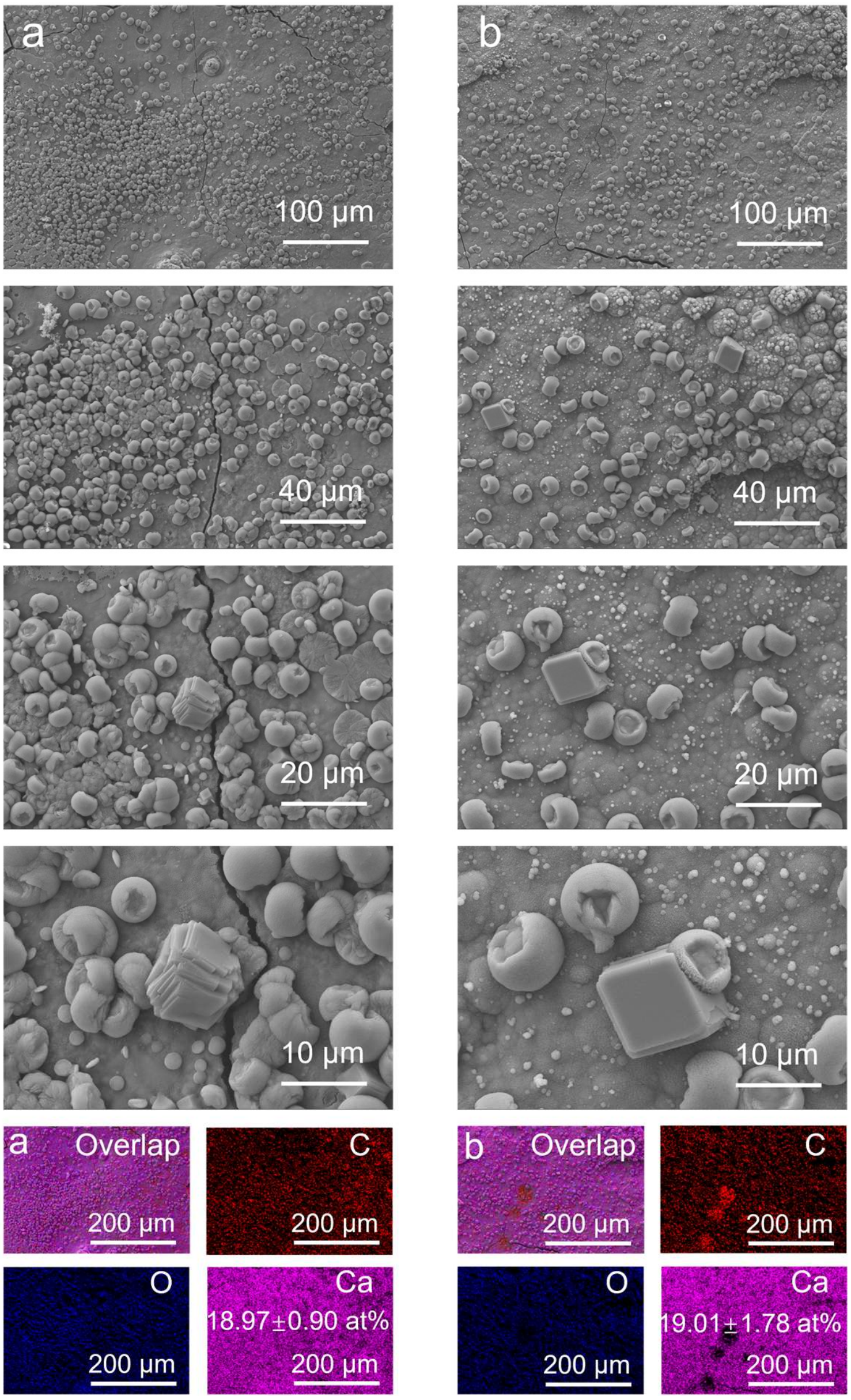

Supplementary Fig. 69. SEM images and EDS mappings of the C0 cathode after experiments in (a) 20PPI-PUS-fenced and (b) 60PPI-PUS-fenced systems at 0.1 A in the presence of 200 mg L^{-1} Ca^{2+}.

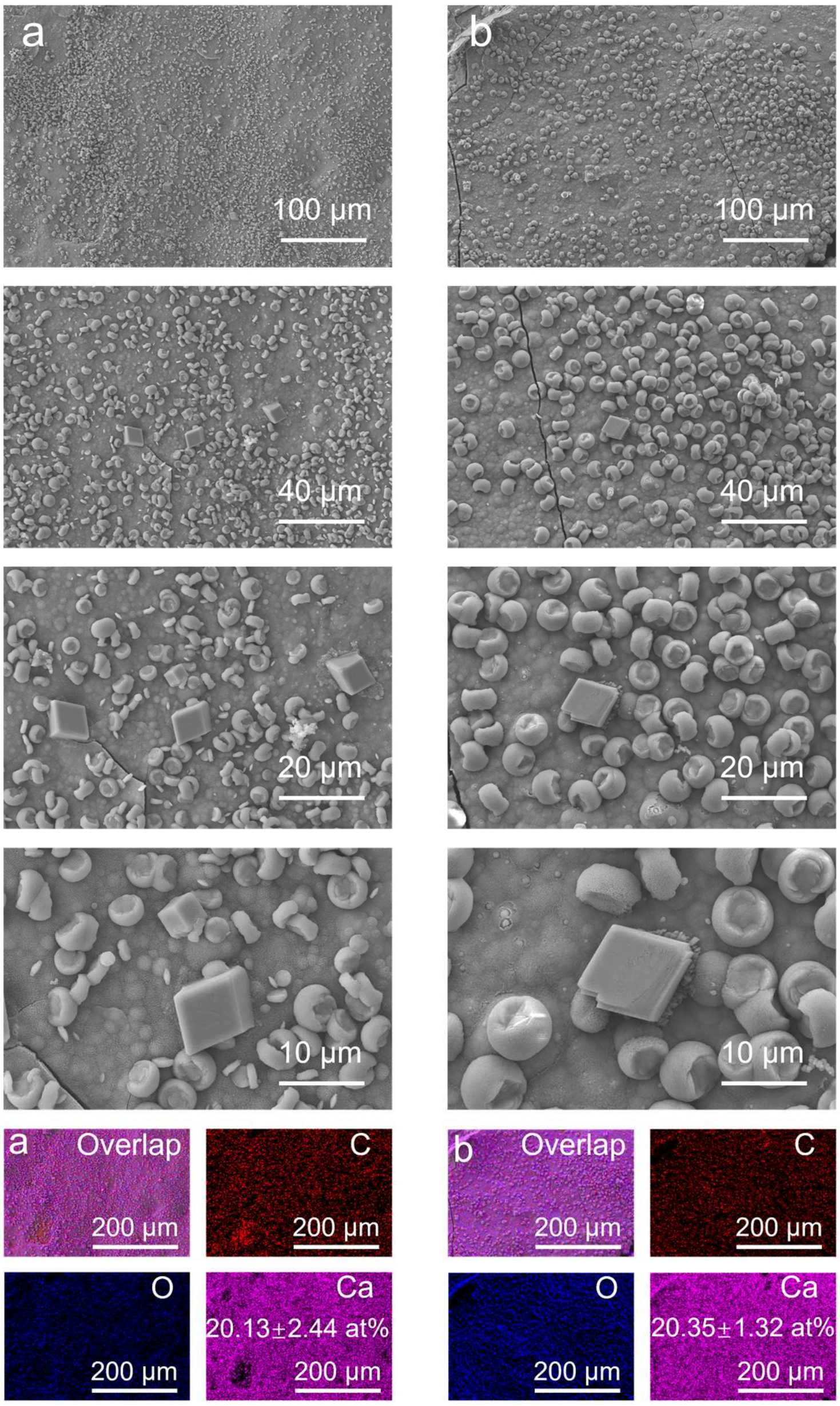

Supplementary Fig. 70. SEM images and EDS mappings of the C18 cathode after experiments in (a) 20PPI-PUS-fenced and (b) 60PPI-PUS-fenced systems at 0.1 A in the presence of 200 mg L^{-1} Ca^{2+}.

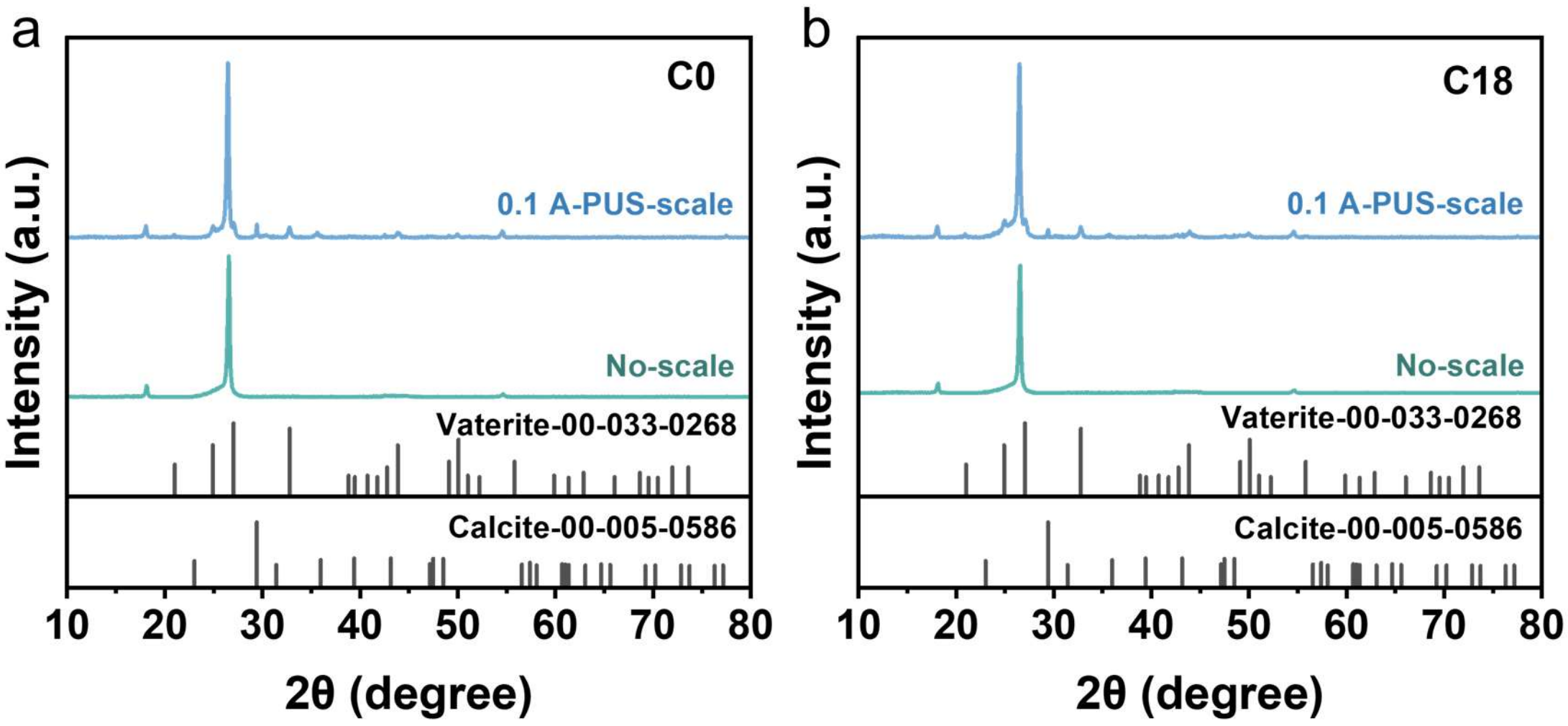

Supplementary Fig. 71. XRD patterns of the (a) C0 and (b) C18 cathodes before and after experiments at 0.1 A under an initial concentration of 200 mg L^{-1} Ca^{2+} in the presence of 60PPI-PUS. Source data for Supplementary Fig. 71 are provided as a Source Data file.

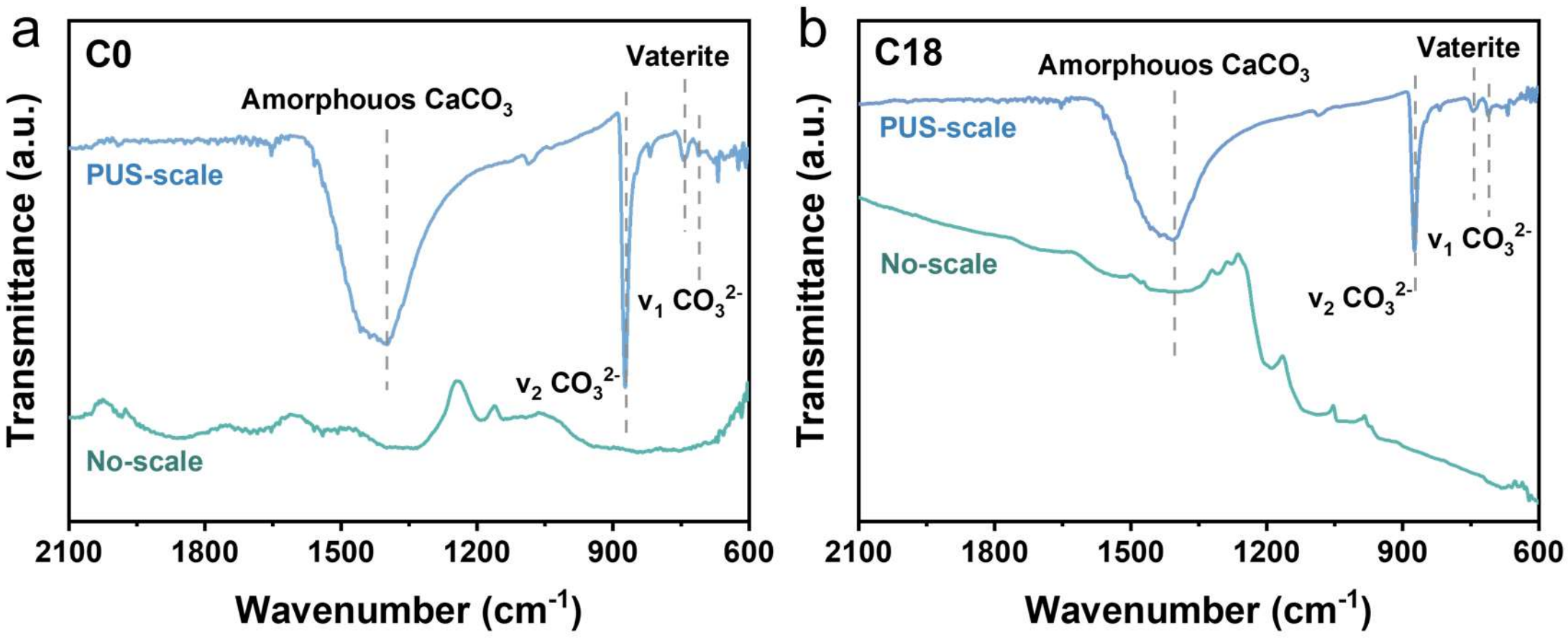

Supplementary Fig. 72. FTIR spectra of the (a) C0 and (b) C18 cathodes before and after experiments at 0.1 A under an initial concentration of 200 mg L^{-1} Ca^{2+} in 60PPI-PUS-fenced systems. Source data for Supplementary Fig. 72 are provided as a Source Data file.

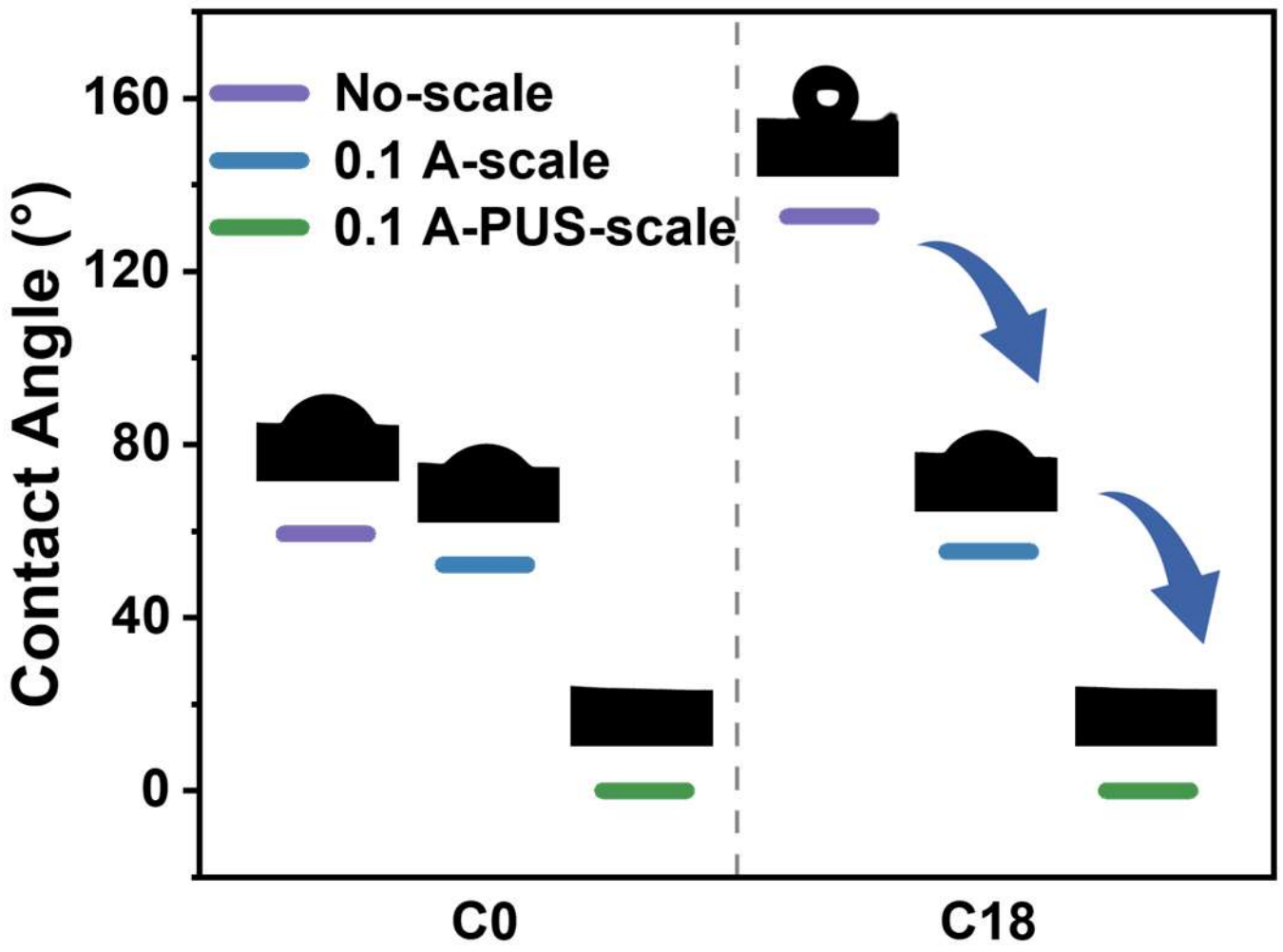

Supplementary Fig. 73. Contact angle measurements of the C0 and C18 cathodes before and after experiments at 0.1 A under an initial concentration of 200 mg L^{-1} Ca^{2+} in the presence or absence of 60PPI-PUS. Source data for Supplementary Fig. 73 are provided as a Source Data file.

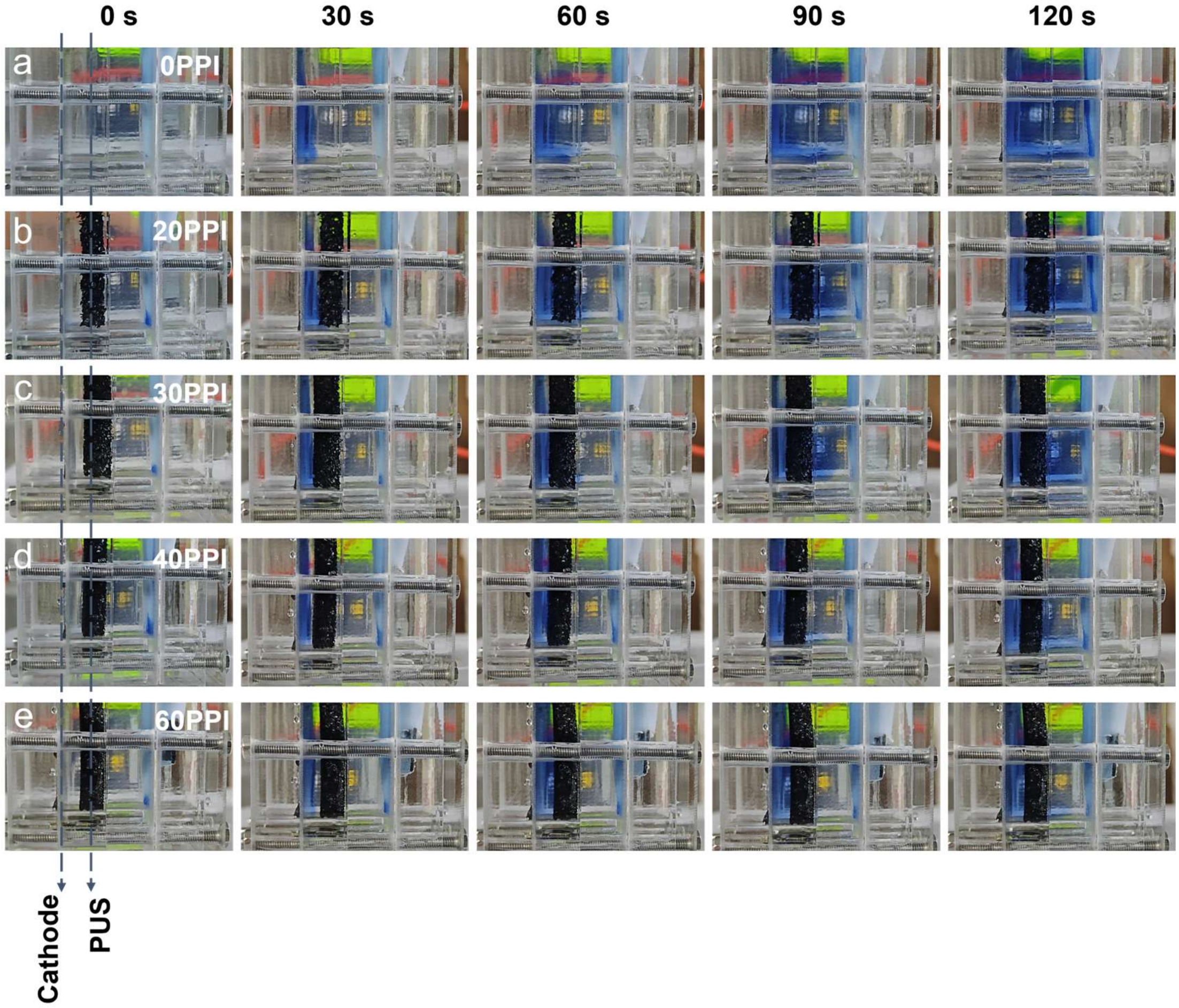

Supplementary Fig. 74. Visualization of the spatial and temporal distributions of the alkaline region employing thymolphthalein as the pH indicator in PUS-fenced cathode systems: (a) No PUS, (b) 20PPI-PUS, (c) 30PPI-PUS, (d) 40PPI-PUS and (e) 60PPI-PUS.

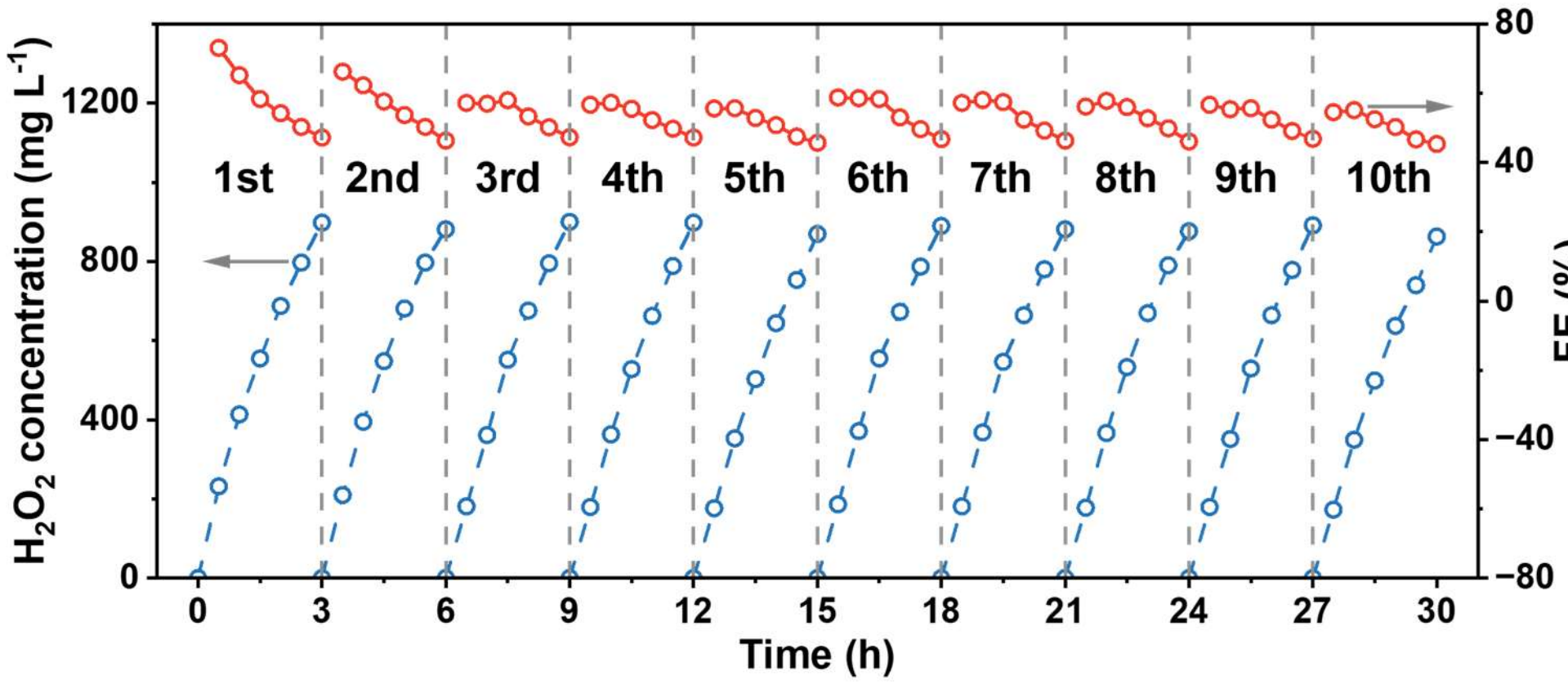

Supplementary Fig. 75. Stability test performed with 60PPI-PUS-fenced cathode of C0 in ten cycles, each of 3 h duration. Reaction conditions: [Na_2SO_4] = 0.05 M, [Ca^{2+}]$_0$ = 200 mg L^{-1}, a current of 0.1 A, a rotation speed of 600 rpm, an initial pH of 7.0 ± 0.1. Source data for Supplementary Fig. 75 are provided as a Source Data file.

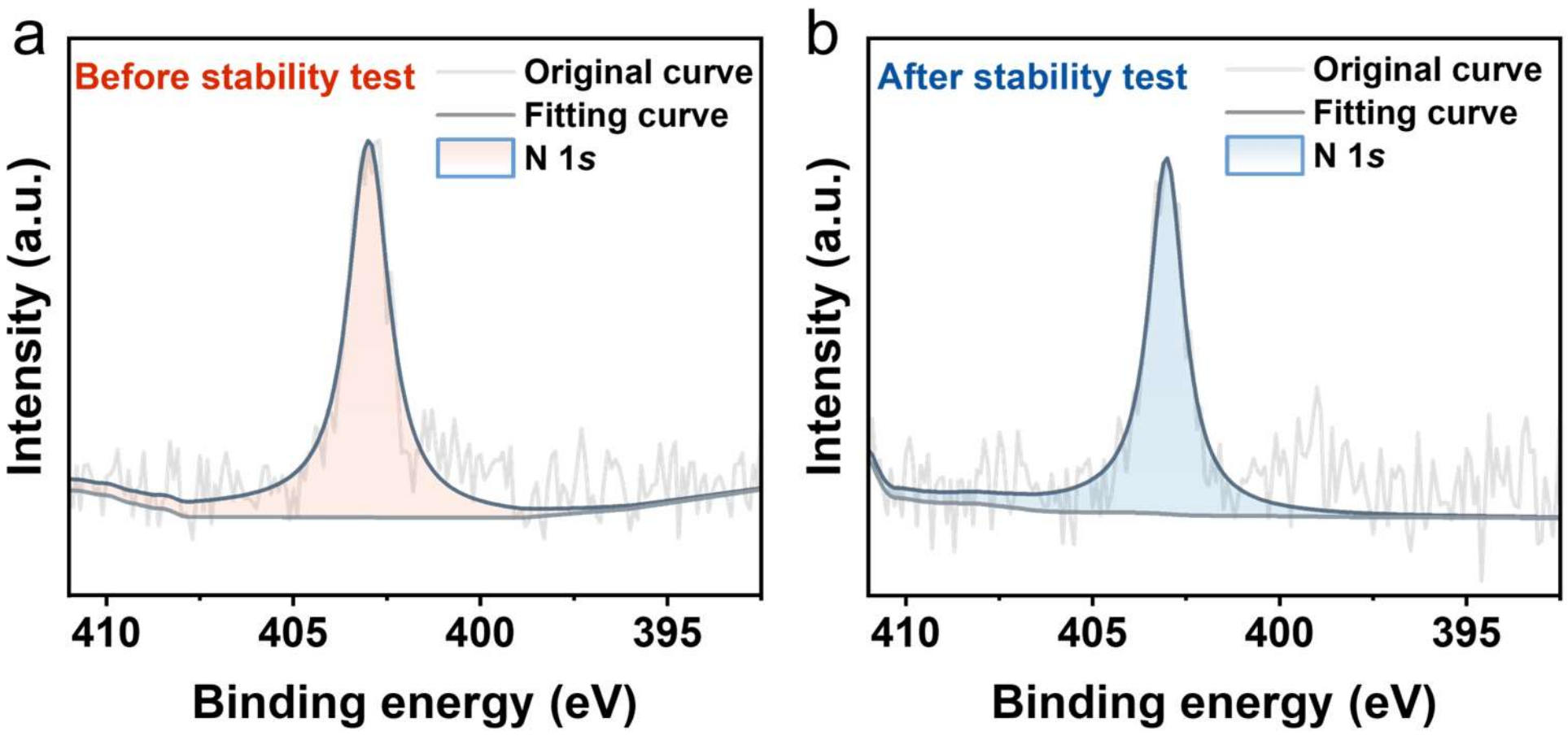

Supplementary Fig. 76. XPS high-resolution N 1*s* spectra of C18 (a) before and (b) after stability measurements. Source data for Supplementary Fig. 76 are provided as a Source Data file.

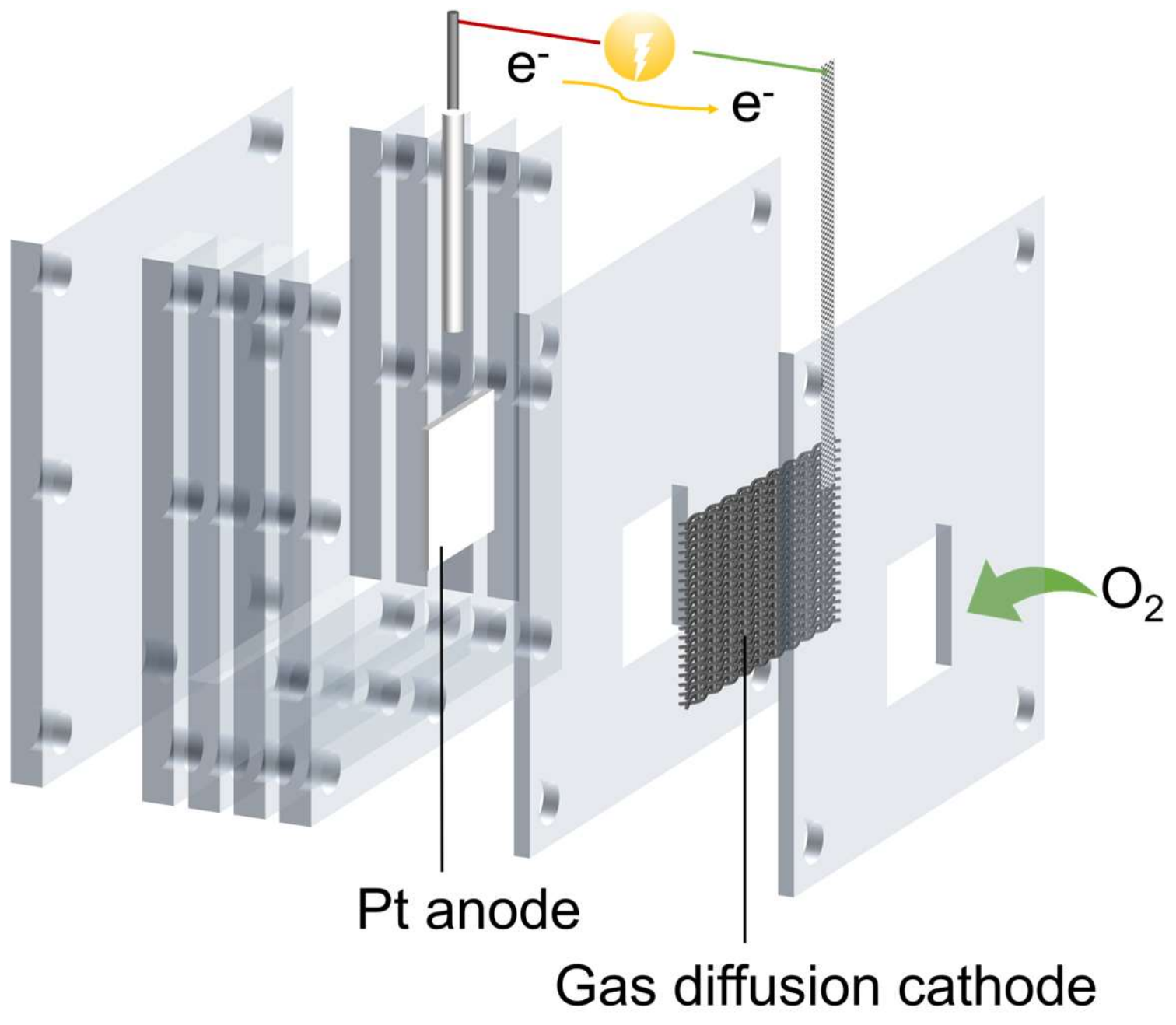

Supplementary Fig. 77. Schematic diagram of the homemade undivided electrolytic reactor for H_2O_2 production.

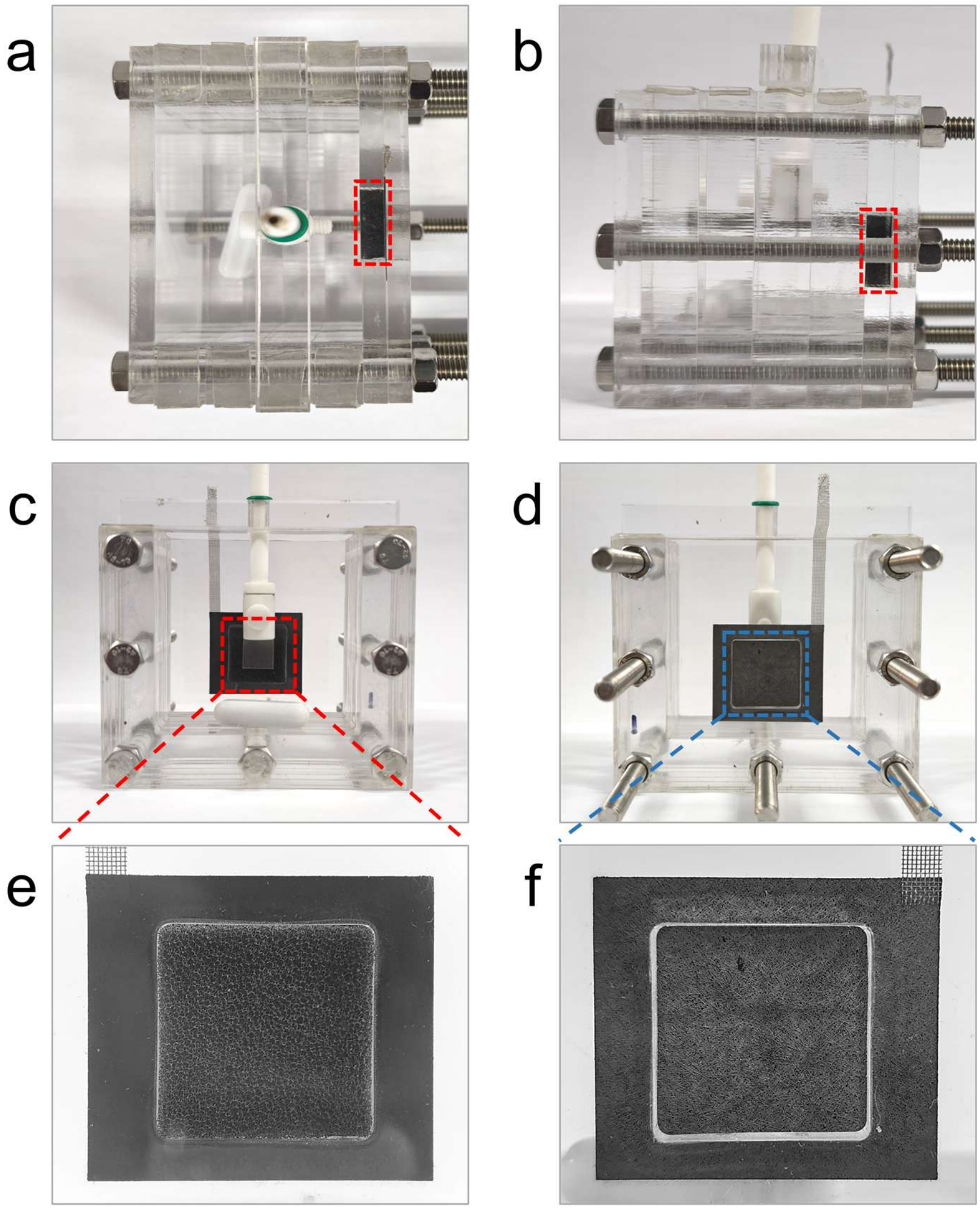

Supplementary Fig. 78. Photograph of the 3D PUS-fenced reactor for H_2O_2 electrosynthesis. (a) Top view, (b) side view, (c) front view, and (d) back view of the 3D PUS-fenced reactor. (e) Enlarged view of the PUS structure and (f) the air-diffusion cathode. The structure enclosed by the red dashed box corresponds to the PUS.

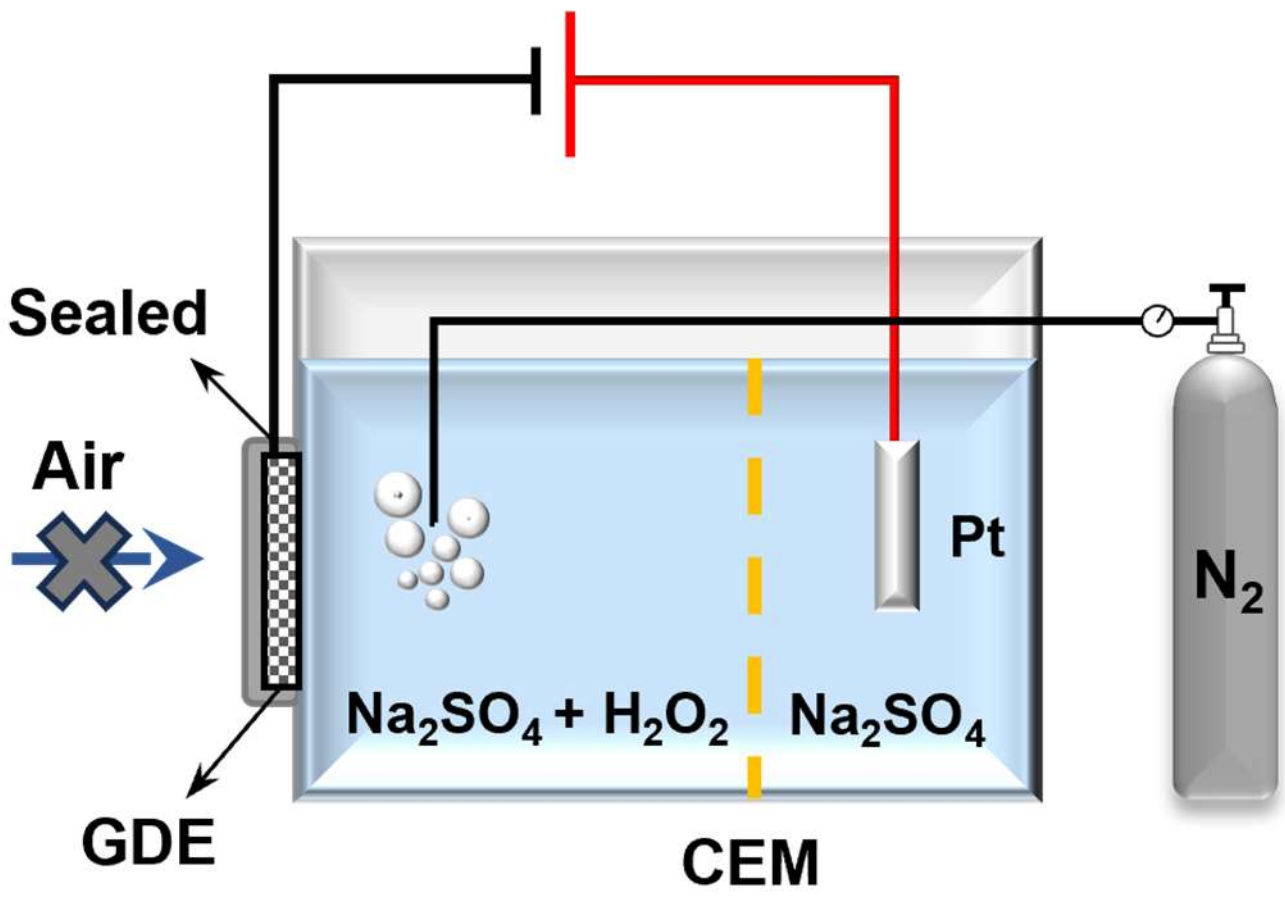

Supplementary Fig. 79. Schematic diagram of the H_2O_2 decomposition experimental configuration.

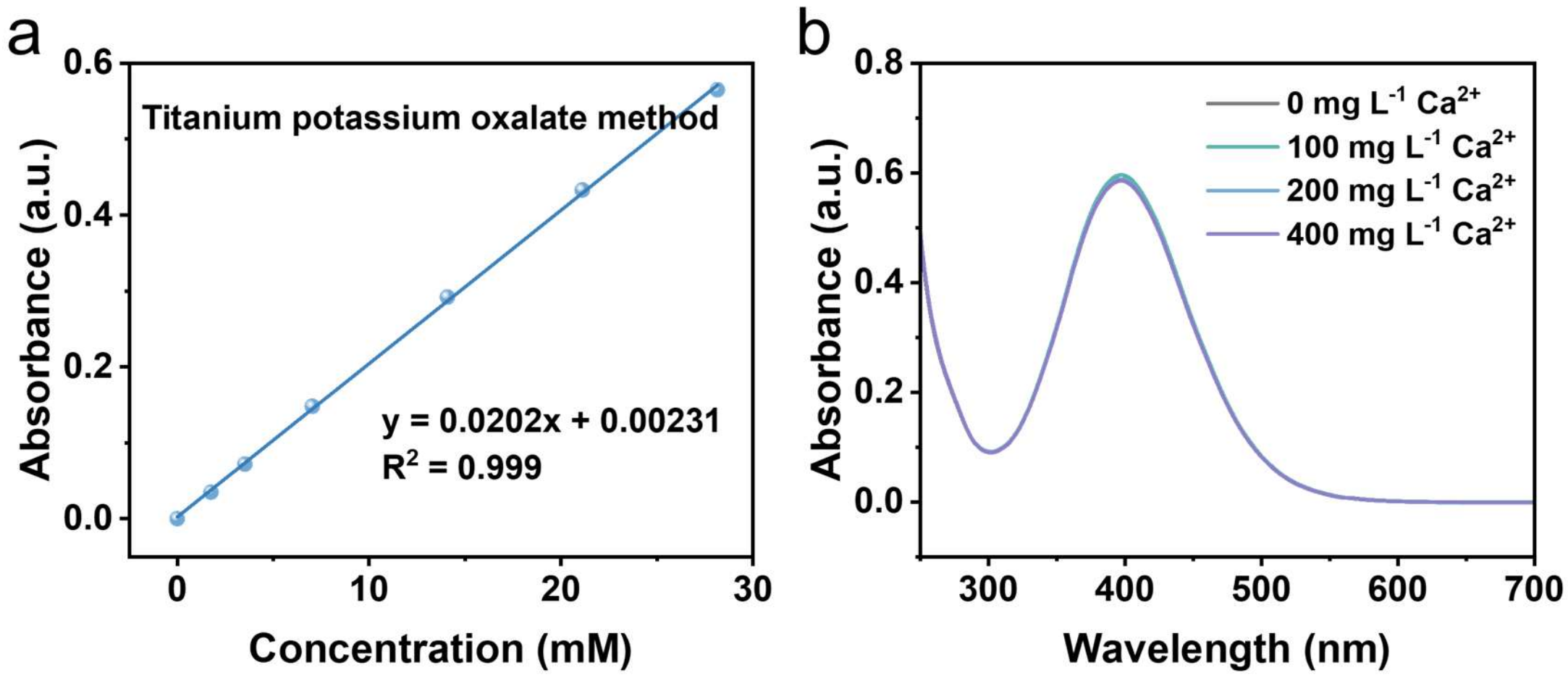

Supplementary Fig. 80. (a) H_2O_2 measurement calibration curve by titanium potassium oxalate method. (b) UV-vis spectra of standard H_2O_2 solution containing 0-400 mg L^{-1} Ca^{2+}. Source data for Supplementary Fig. 80 are provided as a Source Data file.

Supplementary Table 1. The surface Ca element distribution of scaled cathodes obtained from EDS tests.

Electrode	Ca content (at%)	Ca content (at%)	Ca content (at%)	Mean (at%)	SD (at%)
C0-0.1 A-scale	8.09	7.68	9.33	8.37	0.86
C0-0.4 A-scale	22.85	18.49	19.30	20.21	2.32
C18-0.1 A-scale	10.09	9.66	12.32	10.69	1.43
C18-0.4 A-scale	24.85	20.33	17.69	20.96	3.62
C0-20PUS-scale	19.92	18.12	18.87	18.97	0.90
C0-60PUS-scale	20.95	18.64	17.44	19.01	1.78
C18-20PUS-scale	22.95	18.75	18.70	20.13	2.44
C18-60PUS-scale	20.96	21.26	18.83	20.35	1.32

Supplementary Table 2. EIS fitting results of C0 and C18 measured in N_2-saturated 0.1 M NaCl electrolyte containing 10 mM H_2O_2 at different potentials.

Electrode	E (V vs. RHE)	R_s (Ω)	Error (%)	R_1 (Ω)	Error (%)	R_2 (Ω)	Error (%)
C0	0.45 V	10.16	0.37	7.09	3.54	353.3	3.82
C0	0.40 V	10.11	0.38	6.95	3.48	180.3	2.50
C0	0.35 V	10.07	0.36	6.72	3.42	101.0	1.84
C0	0.30 V	9.97	0.42	6.32	4.00	63.5	1.83
C18	0.45 V	10.19	0.47	5.89	4.36	2352	12.00
C18	0.40 V	10.04	0.48	5.63	4.16	702.2	5.17
C18	0.35 V	9.95	0.45	5.32	3.83	233.6	3.99
C18	0.30 V	9.87	0.40	5.16	3.46	103	2.69

Supplementary Table 3. EIS fitting results of C0 and C18 measured in N_2-saturated 0.1 M $CaCl_2$ electrolyte containing 10 mM H_2O_2 at different potentials.

Electrode	E (V vs. RHE)	R_s (Ω)	Error (%)	R_1 (Ω)	Error (%)	R_2 (Ω)	Error (%)
C0	0.45 V	4.95	0.40	4.50	4.60	180.9	1.99
C0	0.40 V	4.92	0.36	4.27	4.05	97.5	1.39
C0	0.35 V	4.87	0.36	3.95	3.99	63.7	1.28
C0	0.30 V	4.84	0.31	3.76	3.67	41.0	1.14
C18	0.45 V	4.77	0.79	2.85	3.13	973.1	6.10
C18	0.40 V	4.94	0.61	2.74	3.87	357	4.37
C18	0.35 V	4.82	0.64	2.84	3.84	130.5	2.56
C18	0.30 V	5.03	0.48	2.54	3.17	59.8	1.68

Supplementary Table 4. EIS fitting results of C0 and C18 cathodes with varying degrees of scaling measured in N_2-saturated 0.1 M Na_2SO_4 solution containing 10 mM H_2O_2.

Electrode	R_s (Ω)	Error (%)	R_1 (Ω)	Error (%)	R_2 (Ω)	Error (%)
No-scale C0	11.98	0.71	9.00	4.75	89.3	10.58
0.1 A-scale C0	12.12	0.43	7.31	3.25	110.6	5.97
0.4 A-scale C0	12.48	0.51	6.04	3.56	181.1	5.10
No-scale C18	12.03	0.61	8.53	4.46	157.7	11.85
0.1 A-scale C18	12.20	0.42	7.13	3.19	191.4	9.10
0.4 A-scale C18	12.64	0.51	5.98	3.88	332.8	8.13
