# Supplementary material for: Calcium-tolerant electrode strategy for efficient hydrogen peroxide electrosynthesis in natural water
Source: Nat Commun. 2026 Aug 31;17:10358. doi: 10.1038/s41467-026-77022-0 (PMC13623961; doi:10.1038/s41467-026-77022-0)
Supplement: Supplementary file 2 [file 41467_2026_77022_MOESM2_ESM.pdf]

Description of Additional Supplementary Files

File Name: Supplementary Data 1

Description: This file includes all the atomic coordinates of the optimized computational models for DFT calculation or MD simulation.

- C0-MD-final.gro
- C0-MD-initial.gro
- C18-MD-final.gro
- C18-MD-initial.gro
- Ead-C0-Ca.vasp
- Ead-C18-Ca.vasp
- Ead-Ca-OOH.vasp
- Ead-Na-OOH.vasp
- water dissociation-Ca-final.vasp
- water dissociation-Ca-initial.vasp
- water dissociation-Ca-middle1.vasp
- water dissociation-Ca-middle2.vasp
- water dissociation-Ca-middle3.vasp
- water dissociation-Ca-middle4.vasp
- water dissociation-Na-final.vasp
- water dissociation-Na-initial.vasp
- water dissociation-Na-middle1.vasp
- water dissociation-Na-middle2.vasp
- water dissociation-Na-middle3.vasp
- water dissociation-Na-middle4.vasp
