# Supplementary material for: Calcium-tolerant electrode strategy for efficient hydrogen peroxide electrosynthesis in natural water
Source: Nat Commun. 2026 Aug 31;17:10358. doi: 10.1038/s41467-026-77022-0 (PMC13623961; doi:10.1038/s41467-026-77022-0)
Supplement: Supplementary file 3 [file 41467_2026_77022_MOESM4_ESM.pdf]

nature portfolio

Peer Review File

Calcium-tolerant electrode strategy for efficient hydrogen peroxide electrosynthesis in natural water

Corresponding Author: Dr Ke Wang

This file contains all reviewer reports in order by version, followed by all author rebuttals in order by version.

Version 0:

Reviewer comments:

Reviewer #1

(Remarks to the Author)
The article employs experimental and theoretical analyses to reveal that dissolved Ca2+ rather than precipitated calcium scale, is the primary factor suppressing the two-electron oxygen reduction reaction (2e- ORR) for H2O2 electrosynthesis in natural water. Furthermore, the surfactant-modified cathode (C18) and the 3D polyurethane sponge (PUS)-fenced cathode architecture developed in this study demonstrate exceptional activity and Ca2+-tolerance by effectively modulating the interfacial microenvironment. This provides new insights and methods for the practical synthesis of hydrogen peroxide in natural water matrices. I have a few questions shown below:
1.The C18 system operates with high selectivity in Ca2+-containing electrolytes. While the authors proposed that Ca2+ shifts the pathway toward the 4e- ORR, characterization using techniques such as the rotating ring-disk electrode (RRDE) is required to calculate the electron transfer number and confirm this mechanism dynamically.
2. To confirm the stability of the non-covalently assembled STAB molecules after long-term stability measurements, characterization using techniques such as post-reaction high-resolution XPS or FTIR is required, especially considering the highly alkaline local microenvironment generated during operation.
3. It is suggested to provide the calculated adsorption energies of key 2e- ORR intermediates (e.g., *OOH) on the C18 surface with and without Ca2+, which would fully validate the proposed thermodynamic shift from the 2e- to the 4e- pathway.
4. In light of the contact angle and ECSA measurements, the authors should provide a reasonable explanation for the performance differences observed between the significantly reduced active area (ECSA drops from 3.01 to 0.94 mF/cm²) and the enhanced H2O2 yield of C18. A discussion on this trade-off is needed.
5. A photograph of the 3D PUS-fenced electrolyzer during actual operation should be provided, as beyond experimental data, such visual documentation holds significant referential value.

Reviewer #2

(Remarks to the Author)
This manuscript investigates the roles of calcium ions and scaling in the electrosynthesis of hydrogen peroxide within natural water systems. The authors propose the perspective that “dissolved Ca^{2+} is harmful, whereas precipitated calcium scale is actually beneficial.” This topic demonstrates a degree of novelty and possesses the potential to challenge conventional wisdom. However, after thoroughly reviewing the main text and supplementary information, I believe the core issue with the current manuscript is that the strength of the existing evidence is insufficient to support such strong conclusions. Specifically, key issues regarding the interfacial microenvironment, the pathways of further H_2O_2 reduction, the representativeness of long-term operation, and the credibility of the theoretical calculations remain unconvincingly resolved.

1. The authors’ core conclusion is that dissolved Ca^{2+} promotes interfacial water dissociation and shifts the reaction pathway toward the $4e^-$ ORR, whereas precipitated calcium scale acts as a local alkaline or proton barrier that inhibits the further reduction of H_2O_2, thereby enhancing $2e^-$ ORR selectivity. However, this proposed mechanism currently relies primarily on electrochemical impedance spectroscopy (EIS) fitting parameters, H_2O_2 decomposition experiments, contact angle measurements, and theoretical calculations. It lacks direct evidence to conclusively substantiate this core claim, such as the characterization of the local interfacial pH microenvironment, changes in proton accessibility, or in-situ/operando monitoring of reaction intermediates.

2. In lines 531-538 of the main text, the authors claim: “The hybrid strategy delivered H_2O_2 electrosynthesis performance in four real water matrices… and maintained 96% of its initial efficacy after 30 h of cyclic testing, highlighting its robust stability. In conclusion, this work… establishing a new paradigm for designing efficient and stable electrocatalytic systems in natural water.” However, almost all experimental evidence supporting the “beneficial scale” mechanism is based on extremely early-stage scaling conditions (only 3 hours of electrolysis at a relatively low current density of 0.1 A). Furthermore, the claimed 30-hour stability is merely an intermittent cyclic test (10 cycles × 3 hours each), not continuous operation. More importantly, the authors themselves observed scale-induced electrode water flooding (Figs. S15 and S44). In actual long-term continuous operation, this flooding and the continuous thickening of the scale layer will inevitably lead to the complete collapse of the three-phase boundary. Therefore, the current experimental results are completely insufficient to support the conclusion that “calcium scale remains beneficial and stable during long-term operation.” Unless a single, continuous long-term test of at least 100 hours is provided, the claim of “efficient and stable in natural water” must be considered a severe overestimation.
3. The authors explicitly point out in the text (Lines 173-179) and SI (Figs. S14, S15, S42, S44) that the formation of calcium scale transitions the electrode surface from hydrophobic to hydrophilic, triggering severe water flooding and inhibiting oxygen transport. Additionally, the ECSA and surface roughness change significantly with the degree of scaling. In Gas Diffusion Electrodes (GDEs), the kinetics and selectivity of the $2e^-$ ORR are extremely sensitive to the gas-liquid distribution at the three-phase boundary and the local oxygen concentration. Since scaling has already triggered such severe mass transport limitations and interfacial physical changes, the attenuation or fluctuation in electrode performance is entirely dominated by these physical factors. However, when discussing the core mechanism, the authors repeatedly attribute this phenomenon to dissolved Ca^{2+} promoting interfacial water dissociation and shifting the reaction pathway toward the $4e^-$ ORR. The current experimental design fails to decouple the ‘physical effects caused by deteriorated mass transport/wettability’ from the ‘chemical catalytic effects of the scale barrier.’ Without ruling out the severe disruption of the three-phase boundary as a dominant confounding factor, this mechanistic hypothesis remains unconvincing.”
4.The manuscript attempts to demonstrate that dissolved Ca^{2+} promotes the further consumption of H_2O_2, while calcium scale inhibits this parasitic reaction, by relying on H_2O_2 decomposition curves, reduction polarization, and impedance analysis. However, these experiments represent oversimplified conditions that are not equivalent to the real ORR environment where H_2O_2 is generated dynamically under continuous oxygen supply and local alkalinization.
5. The authors employ theoretical calculations to demonstrate the core hypothesis that “dissolved Ca^{2+} accelerates interfacial water dissociation.” However, the constructed theoretical model is unreasonable. First, the authors selected standard single-layer graphene as the model, completely ignoring the crucial influence of the abundant oxygen-containing functional groups and defect sites present on the surface of the actual catalyst used (oxygen-doped carbon black, C0). Second, as a core step in the electrochemical reaction, the calculation of the water dissociation energy barrier ignores the critical effects of the applied potential and the interfacial electric field. Consequently, the obtained energy barrier data lack credibility.
6. In the latter half of the manuscript, the authors introduce a C18 surfactant modification and a 3D-PUS sponge fenced strategy to demonstrate the engineering application value of their mechanism. However, the introduction of these two strategies adds more variables to the electrochemical reaction, leading to a somewhat confused core logical chain. The C18 modification (with the introduction of long alkyl chains) not only alters the Zeta potential but significantly changes the electrode’s wettability. The authors must provide control experiments to decouple the effects of improved physical mass transport from electrostatic repulsion, rather than attributing the performance enhancement solely to the repulsion of Ca^{2+}. Furthermore, regarding the 3D-PUS strategy, the physical barrier of the sponge inherently hinders OH^- diffusion and elevates local pH, an effect that typically improves $2e^-$ ORR selectivity in other systems anyway (even in the absence of calcium). Overall, the engineering optimization in the latter half fails to further solidify the fundamental chemical mechanism proposed in the first half; instead, by mixing in additional variables, it blurs the main storyline of the manuscript.
7. Although the title, abstract, and conclusions heavily frame this work around applications in “natural water,” the primary experimental conditions presented in the main text and supplementary materials indicate that the vast majority of the experiments were actually conducted in simplified model systems (e.g., 0.05 M Na_2SO_4 with added Ca^{2+}, or $NaCl/CaCl_2$). Real natural water matrices are highly complex, involving the simultaneous and interactive presence of not only Ca^{2+} but also Mg^{2+}, bicarbonate, natural organic matter, sulfate, chloride, and other constituents. By attributing nearly all performance variations solely to calcium ions without clearly delineating the boundaries of applicability, the manuscript’s claim of “universal applicability in natural water” is overstated.

Reviewer #3

(Remarks to the Author)
Summary: This manuscript targets Ca2+-related performance loss in electrochemical H2O2 production in realistic aqueous systems. Authors argue that dissolved Ca2+ cation rather than precipitated CaCO3 scale is the dominant source of selectivity loss and propose two mitigation strategies: (i) employment of quaternary ammonium surfactants to modify the oxygen-doped carbon cathode (C0) with tunable surface wettability and electrostatic potential (C8, C10, C14 and C18) and (ii) incorporation of a 3D polyurethane sponge (PUS) on the cathode surface to induce a localized alkaline environment. Authors managed to improve H2O2 selectivity and Ca2+ tolerance using modified electrodes and accomplish their mechanistic interpretation using scaling characterization, electrochemical probes, and MD/DFT calculations. I am a computational chemist, so my comments will focus more on mechanistic and computational components of the manuscript.

Major Issues:
(1) Central paradox of more scaling yet better performance is not convincingly resolved. A striking observation is that C18 can deposit more CaCO3 scaling than C0 while delivering superior Ca2+ tolerance and higher H2O2 selectivity. Authors frame this as a challenge to the prevailing opinion about the detrimental impact of scaling. However, the mechanistic

explanation remains incomplete. For example, why does additional scaling on C18 not systematically correlate with an improved protection, and how do the surface location and morphology of scales affect mass transport and active site accessibility? At minimum, the manuscript should clarify where scales form (surface vs pores vs phase boundary) and demonstrate their effects in mass transport.
(2) Multiple mechanisms are invoked without being disentangled or quantified. Authors attribute the improved selectivity to a combination of multiple factors, including (i) exclusion of Ca2+ from a more positive surface potential, (ii) disruption of hydrogen bond networks and limited proton transfers via hydrophobicity, (iii) formation of a highly alkaline microenvironment from the PUS confinement, and (iv) suppression of side reactions like H2O2 reduction and decomposition from protective roles of scales. All these effects can compete and overlap with each other, and authors should perform an ablation study to elucidate which factor is more deterministic. To “make up” some examples, dissolved Ca2+ may suppress the synthesis of H2O2 by accelerating O–O dissociation, and C18 may play an opposite role by slowing proton transfers. Without even a simplest rate and transport analysis, the conclusions read as a list of narratives rather than testable mechanisms.
(3) Electrostatic interpretation based on PZC/zeta potential is oversimplified. Authors link the exclusion of Ca2+ to a more positive surface potential of C18. However, PZC and zeta potential measured under non-operando conditions cannot be directly mapped onto the interfacial charge and potential. Authors should discuss the relationship between PZC, electrode potential, specific adsorption, and double-layer structure. Moreover, authors suggest enhanced interfacial cation density and claim electrostatic repulsion of Ca2+, which appears contradictory unless clarified.
(4) Claims about highly alkaline interfacial microenvironment rely on questionable proxies. Authors argue PUS-fenced electrode to restrict OH− diffusion and therefore create a localized alkaline microenvironment that suppresses the Ca2+ activity, but they bulk pH values taken ~1 cm from the electrode, which is not a reliable proxy. Authors should either (i) provide more appropriate interfacial pH diagnostics (e.g., microelectrode profiling at the surface, spectroscopic probes, or modeling constrained by measured currents), or (ii) substantially soften this claim and reframe it as a meso-scale transport effect.
(5) DFT and MD modeling do not fully support the mechanistic claims. Reported MD simulations (20 ns NVT, GAFF/RESP, explicit electrolyte) provide structural information but do not establish proton transfer kinetics. Therefore, the claim about slower proton transfer kinetics from reduced hydrogen bond or water density is not directly grounded. Charge and potential of the electrode are not physically implemented and the claim about the repulsion of Ca2+ is not straightforward either. For DFT calculations, while the computational workflow is standard (VASP PBE/D3, NEB), the treatment for Ca2+ adsorption and energetics is incomplete without being fully solvated by water molecules.

Minor Issues:
(1) Authors should clarify the rationale for selecting the specific active sites and models used in DFT, and whether alternative defects and functional group sites were tested.
(2) Authors interpret results from electrochemical impedance spectroscopy (EIS) (e.g., “hydrogen adsorption resistance”) as evidence of slower proton transfers. They should provide the equivalent circuit, fitting quality, parameter identifiability, and a clearer connection between these parameters, and the surface availability of protons.
(3) Authors should discuss the diffusion and H-type cell experiments used to support Ca2+ exclusion more carefully with respect to their transferability to polarized cathode surfaces.
(4) Authors mention that C18 differs from C0 not only in surfactant grafting but also in defect level and possibly surface chemistry. They should clarify how these confounding factors are controlled.
(5) Authors should provide error bars, replicate numbers, and statistical treatment consistently for performance metrics (FE, production rate, stability) and scaling quantification (ICP/EDS/profilometry).

Recommendation: The work contains potentially publishable observations and a practically relevant direction: Ca2+-tolerant H2O2 electrosynthesis in realistic aqueous systems. However, the mechanistic understanding has not reached standard expected for Nature Communications. I recommend a major revision, with emphasis on major and minor issues mentioned above.

Reviewer #4

(Remarks to the Author)
I co-reviewed this manuscript with one of the reviewers who provided the listed reports. This is part of the Nature Communications initiative to facilitate training in peer review and to provide appropriate recognition for Early Career Researchers who co-review manuscripts.

Version 1:

Reviewer comments:

Reviewer #1

(Remarks to the Author)
In the revised manuscript, the author conducted an extensive series of experiments and measurements, including RRDE tests and ATR-SEIRAS, to strengthen the empirical foundation of the study. Additionally, the authors have carefully adjusted the DFT calculations, including modifying the model to align better with realistic conditions and incorporating the effects of applied potential and interfacial electric fields. The revised manuscript has seen significant improvements in terms of logical structure, data support, and the depth of argumentation. In my opinion, the current manuscript meets the high standard of Nature Communications. I’m pleased to recommend that the editorial team accept this manuscript.

Reviewer #2

(Remarks to the Author)
The authors have significantly revised the manuscript and have addressed the major concerns raised in my previous review. I appreciate the substantial efforts made to strengthen the mechanistic understanding, improve experimental validation, and clarify the scope of the proposed strategy. The revised manuscript is considerably improved compared with the original version.
In particular, the authors have provided additional evidence regarding the interfacial microenvironment, the role of dissolved Ca^{2+} versus precipitated calcium scale, and the influence of scaling on H_2O_2 selectivity. These new results, together with the revised discussion, substantially strengthen the proposed mechanism and alleviate my previous concerns that the conclusions relied primarily on indirect electrochemical evidence. Regarding the theoretical calculations, the authors have comprehensively revised the theoretical calculations by reconstructing the interfacial model and incorporating electrochemical field constraints into the simulations. These improvements address my previous concerns regarding the oversimplified surface model and the absence of potential-dependent interfacial effects. The authors have also appropriately revised several statements that were previously too broad, especially regarding the universality of the approach in natural water environments. The updated discussion now better acknowledges the complexity of real water matrices and the potential influence of other ions and environmental factors. Furthermore, the authors have clarified the role of the engineering strategies introduced in the latter part of the manuscript, improving the overall logical consistency of the study.
Overall, I believe that the revised manuscript presents a more convincing and comprehensive study. The concept that dissolved calcium ions and calcium scale can exert distinct and even opposite effects on electrochemical H_2O_2 production is interesting and provides valuable insights into the design of electrocatalytic systems operating under realistic aqueous conditions. The major concerns raised previously have been adequately addressed, and the manuscript has reached a level suitable for publication.

Reviewer #3

(Remarks to the Author)
The authors have addressed my questions. The paper can be published.

Reviewer #4

(Remarks to the Author)
I co-reviewed this manuscript with one of the reviewers who provided the listed reports. This is part of the Nature Communications initiative to facilitate training in peer review and to provide appropriate recognition for Early Career Researchers who co-review manuscripts.

Open Access This Peer Review File is licensed under a Creative Commons Attribution 4.0 International License, which permits use, sharing, adaptation, distribution and reproduction in any medium or format, as long as you give appropriate credit to the original author(s) and the source, provide a link to the Creative Commons license, and indicate if changes were made.

In cases where reviewers are anonymous, credit should be given to 'Anonymous Referee' and the source.
The images or other third party material in this Peer Review File are included in the article's Creative Commons license, unless indicated otherwise in a credit line to the material. If material is not included in the article's Creative Commons license and your intended use is not permitted by statutory regulation or exceeds the permitted use, you will need to obtain permission directly from the copyright holder.
To view a copy of this license, visit https://creativecommons.org/licenses/by/4.0/

Modifications to comments from Reviewer #1

General comments

The article employs experimental and theoretical analyses to reveal that dissolved Ca^{2+} rather than precipitated calcium scale, is the primary factor suppressing the two-electron oxygen reduction reaction ($2e^-$ ORR) for H_2O_2 electrosynthesis in natural water. Furthermore, the surfactant-modified cathode (C18) and the 3D polyurethane sponge (PUS)-fenced cathode architecture developed in this study demonstrate exceptional activity and Ca^{2+}-tolerance by effectively modulating the interfacial microenvironment. This provides new insights and methods for the practical synthesis of hydrogen peroxide in natural water matrices. I have a few questions shown below:

Specific comments

1.The C18 system operates with high selectivity in Ca^{2+}-containing electrolytes. While the authors proposed that Ca^{2+} shifts the pathway toward the $4e^-$ ORR, characterization using techniques such as the rotating ring-disk electrode (RRDE) is required to calculate the electron transfer number and confirm this mechanism dynamically.

2. To confirm the stability of the non-covalently assembled STAB molecules after long-term stability measurements, characterization using techniques such as post-reaction high-resolution XPS or FTIR is required, especially considering the highly alkaline local microenvironment generated during operation.

3. It is suggested to provide the calculated adsorption energies of key $2e^-$ ORR intermediates (e.g., *OOH) on the C18 surface with and without Ca^{2+}, which would fully validate the proposed thermodynamic shift from the $2e^-$ to the $4e^-$ pathway.

4. In light of the contact angle and ECSA measurements, the authors should provide a reasonable explanation for the performance differences observed between the significantly reduced active area (ECSA drops from 3.01 to 0.94 mF/cm^2) and the enhanced H_2O_2 yield of C18. A discussion on this trade-off is needed.

5. A photograph of the 3D PUS-fenced electrolyzer during actual operation should be provided, as beyond experimental data, such visual documentation holds significant referential value.

1.The C18 system operates with high selectivity in Ca^{2+}-containing electrolytes. While the authors proposed that Ca^{2+} shifts the pathway toward the $4e^-$ ORR, characterization using techniques such as the rotating ring-disk electrode (RRDE) is required to calculate the electron transfer number and confirm this mechanism dynamically.

Response:

Thank you for this constructive suggestion. We fully agree that our original manuscript lacked direct in-situ evidence for the Ca^{2+}-induced shift in ORR pathway from $2e^-$ to $4e^-$, and that the rotating ring-disk electrode (RRDE) measurements are the appropriate technique to quantify this effect dynamically. In response, we have conducted comprehensive RRDE experiments to address this gap.

RRDE measurements were performed in Na^+ and Ca^{2+} systems, respectively. The specific experimental setup and testing parameters are provided in the Methods section below. The analysis of the RRDE results is presented as follows:

- **(1) Key RRDE results: Ca^{2+} shifts the ORR pathway toward the $4e^-$ route**

  The RRDE results are presented in the following **Figure**. In the Ca^{2+} system, the electron transfer number (n) is consistently higher than in the Na^+ system across the entire potential range tested, and the H_2O_2 selectivity is correspondingly lower. This provides direct, in-situ and dynamic evidence that dissolved Ca^{2+} shifts the ORR pathway from the $2e^-$ toward the $4e^-$ direction under continuous O_2 reduction conditions.

- **(2) Deconvolution of ORR current density into two-step sequential reactions**

  To further elucidate the specific kinetic step at which Ca^{2+} exerts its effect, we deconvoluted the disk current density into two sequential reaction pathways following the established methodology in the literature[23]. In this framework, the total ORR current density (j_D) is deconvoluted into:

  Oxygen-to-peroxide (O2P): The $2e^-$ reduction of O_2 to H_2O_2. The kinetic current density for this step (j_{O2P}) represents the intrinsic activity for the $2e^-$ ORR pathway.

  Peroxide-to-hydroxide (P2H): The further $2e^-$ reduction of the in-situ generated H_2O_2 to H_2O. The kinetic current density for this step (j_{P2H}) represents the parasitic H_2O_2 consumption pathway.

  (a) j_{O2P} (O_2 to H_2O_2): No significant difference between Na^+ and Ca^{2+} systems.

  The kinetic current for the $2e^-$ reduction of O_2 to H_2O_2 is nearly identical in the Na^+ and Ca^{2+} electrolytes across the entire potential range. This demonstrates that dissolved Ca^{2+} does not directly inhibit the $2e^-$ ORR pathway at the O_2 activation step. The intrinsic activity of the C0 catalyst for H_2O_2 generation is preserved even in the presence of Ca^{2+}.

  (b) j_{P2H} (H_2O_2 to H_2O): Dramatically enhanced by Ca^{2+}.

  In contrast, the kinetic current for the further reduction of H_2O_2 to H_2O is substantially higher in the Ca^{2+} system than in the Na^+ system. This directly demonstrates that Ca^{2+} accelerates the parasitic consumption of in-situ generated H_2O_2, converting it to H_2O and thereby reducing the net H_2O_2 selectivity.

- **(3) Consistency with the central thesis of this work**

  The RRDE deconvolution results are in complete agreement with the core mechanistic conclusion of our study: dissolved Ca^{2+} suppresses net H_2O_2 electrosynthesis primarily by accelerating the further reduction of H_2O_2 to H_2O, rather than by inhibiting the $2e^-$ O_2-to-H_2O_2 step itself.

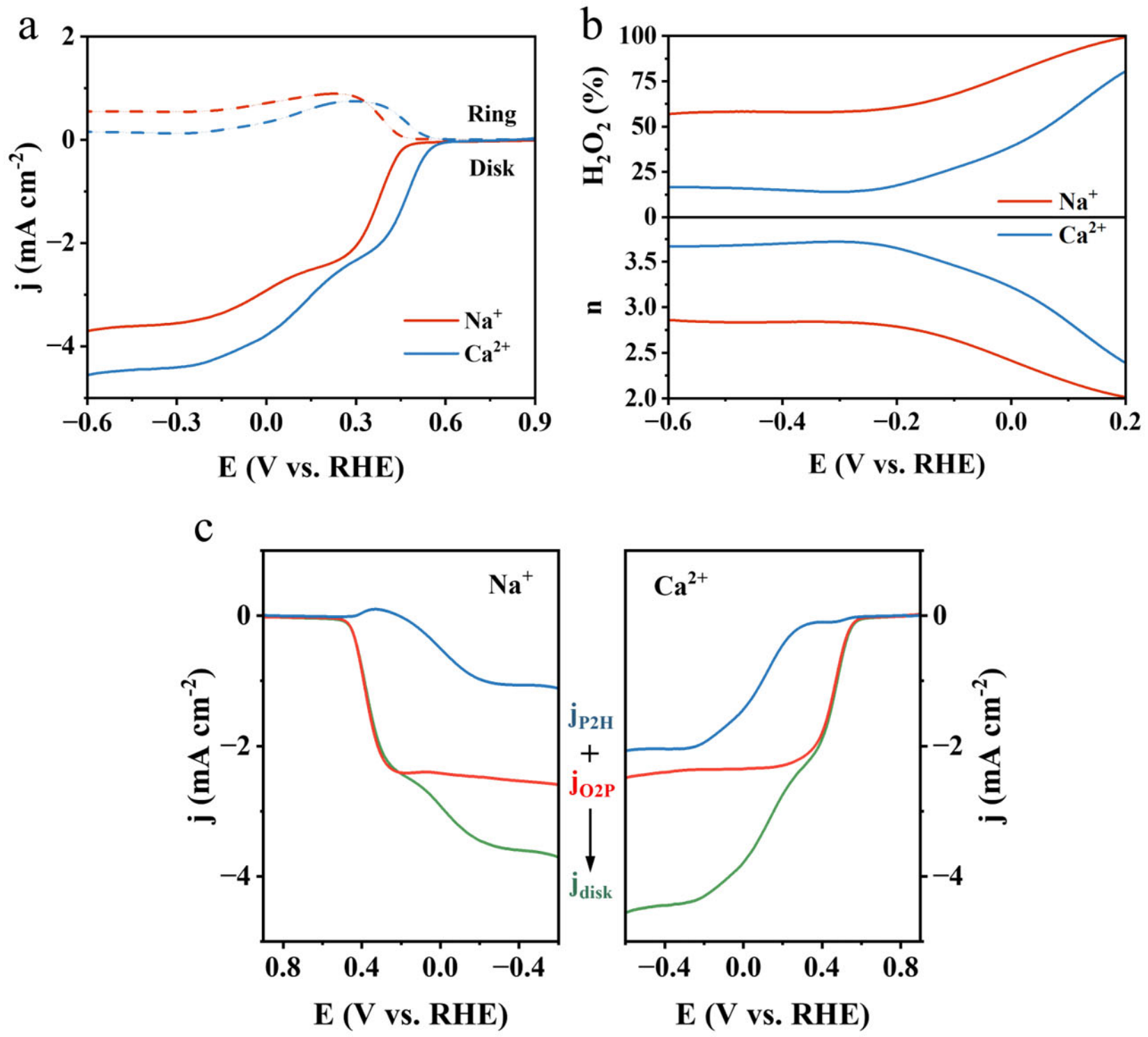

Figure RRDE measurements for C0. (a) LSV curves of C0 tested at 1600 rpm in O_2-saturated 0.1M NaCl or 0.1M $CaCl_2$. (b) The H_2O_2 selectivity and electron transfer number of C0 in different systems. (c) Current deconvolution analysis of C0 in Na^+ and Ca^{2+} systems.

The Revised Content in Manuscript

Based on your comments, we have conducted comprehensive RRDE experiments and deconvolution analysis to directly prove the Ca^{2+}-induced shift in ORR pathway from $2e^-$ to $4e^-$. The following specific revisions have been implemented in the manuscript and supporting information:

- **New Figures and Discussion in Section "Evidence for the suppression of $2e^-$ ORR by dissolved Ca^{2+}." [Line 135-149, Page 5 (Revised)]**

To provide direct in-situ evidence for the Ca^{2+}-induced ORR pathway shift, rotating ring-disk electrode (RRDE) measurements were performed in O_2-saturated electrolyte **(Fig. 1b)**. In the presence of Ca^{2+}, the electron transfer number (n) increased and the H_2O_2 selectivity correspondingly decreased across the entire potential range compared to the Na^+ system **(Fig. 1c)**, confirming that dissolved Ca^{2+} dynamically shifts the ORR pathway toward the $4e^-$ direction. To further identify the specific kinetic step affected, the total ORR current (j_D) was deconvoluted into two sequential reactions: oxygen-to-peroxide (O2P) and peroxide-to-hydroxide (P2H) **(Fig. 1d)**[23]. The kinetic current for O2P (j_{O2P}) showed negligible difference between the Na^+ and Ca^{2+} systems, indicating that dissolved Ca^{2+} does not directly inhibit the $2e^-$ reduction of O_2 to H_2O_2. In contrast, the kinetic current for P2H (j_{P2H}) was substantially enhanced in the Ca^{2+}-containing electrolyte, demonstrating that Ca^{2+} accelerates the parasitic electroreduction of in-situ generated H_2O_2 to H_2O. These results provide direct in-situ kinetic evidence that

dissolved Ca^{2+} suppresses net H_2O_2 electrosynthesis primarily by promoting the further reduction of H_2O_2 rather than by inhibiting the O_2-to-H_2O_2 step itself.

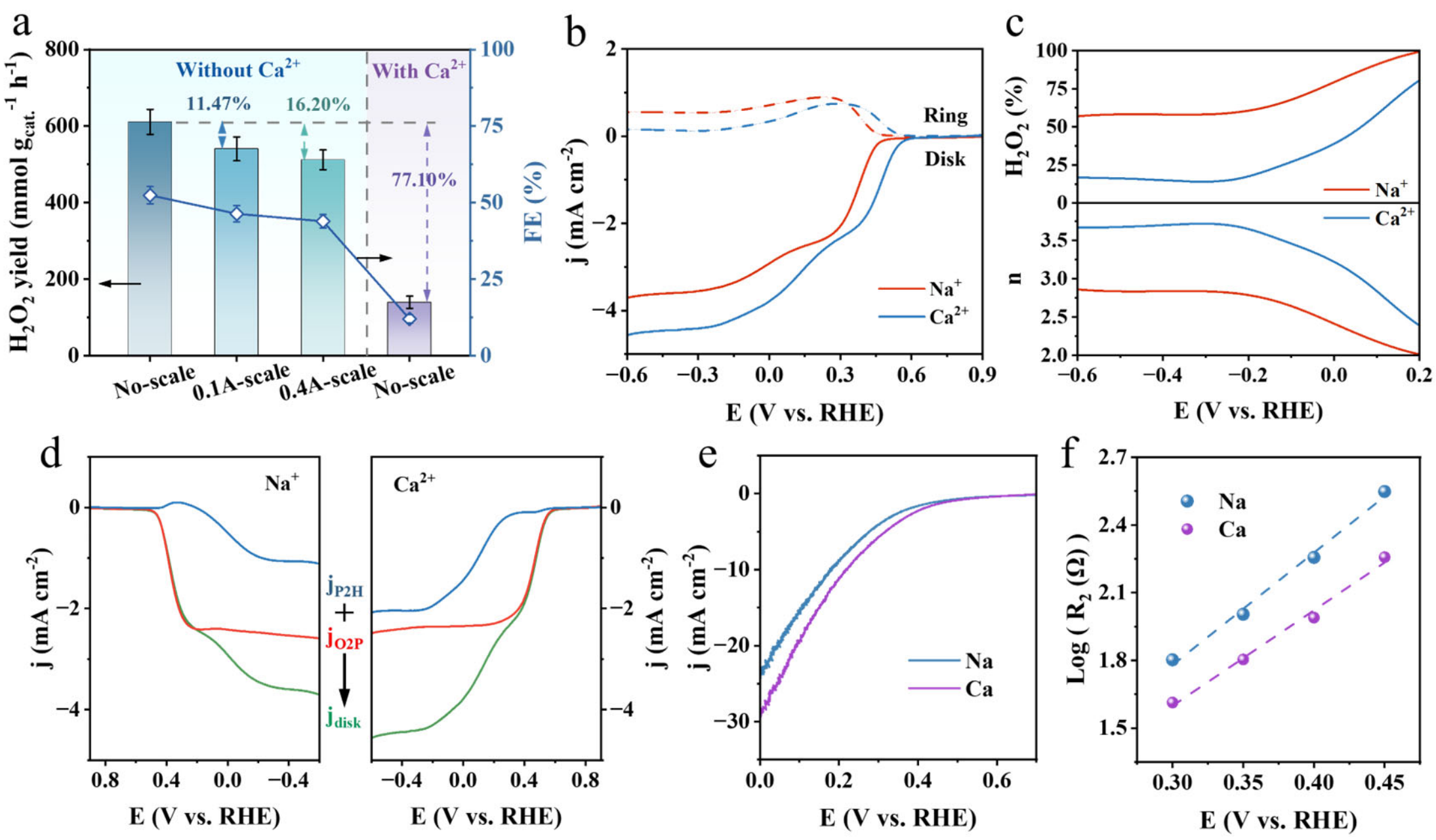

Fig. 1. Effects of dissolved Ca^{2+} on $2e^-$ ORR for oxygen-doped carbon black (named C0). (a) H_2O_2 electrosynthesis performance of C0 cathodes with varying degrees of scaling in 0.05M Na_2SO_4 and no-scale cathode in 0.05M Na_2SO_4 and 200 mg/L Ca^{2+}. (b) LSV curves of C0 from RRDE tested at 1600 rpm in O_2-saturated 0.1M NaCl or 0.1M $CaCl_2$. (c) The electron transfer number and H_2O_2 selectivity of C0 in different systems. (d) Deconvolution analysis of disk currents in Na^+ and Ca^{2+} systems. (e) LSV curves and (f) EIS-derived Tafel plots obtained from the hydrogen adsorption resistance R_2 of C0 in N_2-saturated 0.1M NaCl or $CaCl_2$ electrolytes containing 10mM H_2O_2.

- **New Figures in Supplementary Information:**
  Fig. 1a in the original manuscript has been transferred to the Supplementary Information and is now renumbered as **Supplementary Fig. 1**.

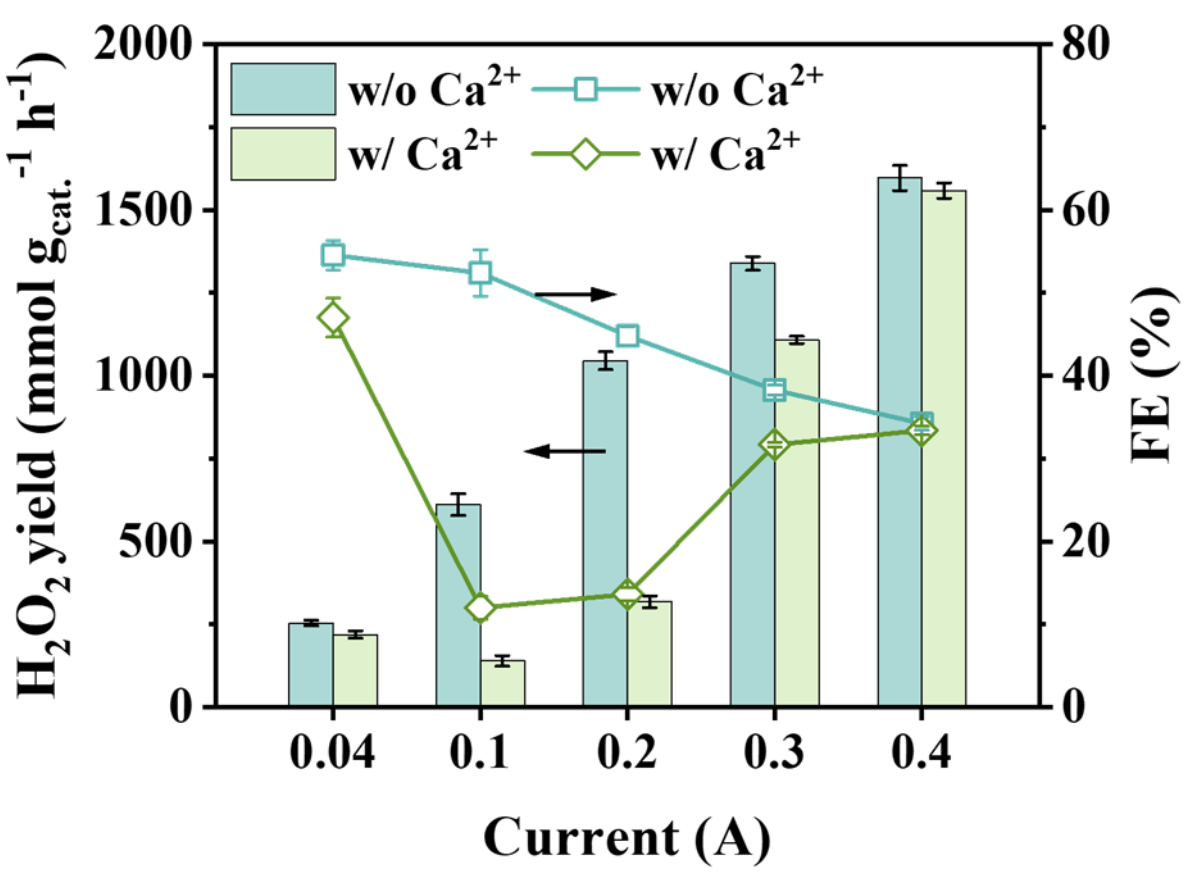

Supplementary Fig. 1. Effects of current densities on H_2O_2 electrosynthesis performance for C0 in the absence (w/o) and presence (w/) of 200 mg/L Ca^{2+}. Reaction conditions: $[Na_2SO_4]$ = 0.05 M, a rotation speed of 600 rpm, an initial pH of 7 and electrolysis of 3 h.

Supplementary Fig. 3 in the original Supplementary Information has been replaced and is now renumbered as **Supplementary Fig. 5**.

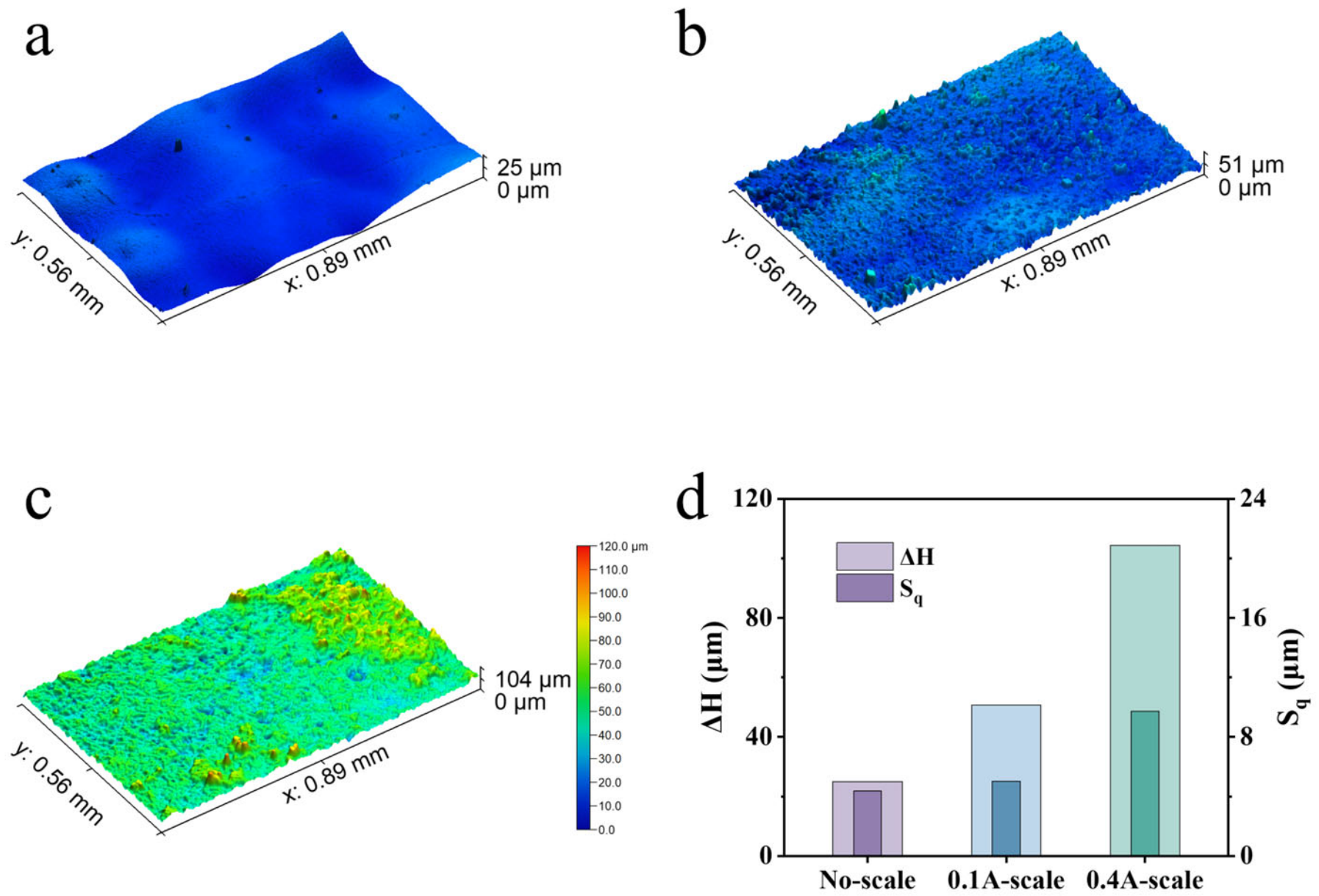

Supplementary Fig. 5. 3D surface profilometry of C0 (a) before experiments and after experiments at (b) 0.1A and (c) 0.4A in the presence of 200 mg/L Ca^{2+}. Variations in the height range (ΔH) and RMS roughness (S_q) of C0 cathodes with varying degrees of scaling. Reaction conditions: [Na_2SO_4] = 0.05 M, [Ca^{2+}]$_0$ = 200 mg/L, a rotation speed of 600 rpm, an initial pH of 7 and electrolysis of 3 h.

- **Updated Methods Section [Line773-800, Page 24 (Revised)]**

RRDE measurement and current density deconvolution for ORR

The selectivity of 2e$^-$ ORR was evaluated using a rotating ring-disk electrode (RRDE) setup (AFMSRCE, Pine, Physicochemical Co. Ltd.), consisting of a glassy carbon disk electrode (diameter: 5.61mm, area: 0.2475 cm^2) and a platinum ring electrode (inner diameter: 6.25mm, outer diameter: 7.92mm, area: 0.1866 cm^2).10 μL of the catalyst ink was drop-cast onto the disk electrode and allowed to dry naturally, serving as the working electrode. An Ag/AgCl electrode and a platinum wire were used as the reference and counter electrodes, respectively. Prior to electrochemical measurements, the catalyst was electrochemically activated by performing cyclic voltammetry (CV) scans at 50 mV/s until a stable voltammogram was obtained. Linear sweep voltammetry (LSV) was conducted in O_2-saturated 0.1 M NaCl or 0.1 M $CaCl_2$ solutions at a scan rate of 10 mV/s, with the RRDE rotating at 1600 rpm. The platinum ring electrode was held at 1.2 V vs. RHE to detect the generated H_2O_2. The H_2O_2 selectivity and the electron transfer number (n) were calculated using the following equations:

$$H_2O_2\ selectivity\ (\%) = \frac{I_R/N}{I_D + I_R/N} \times 200$$

$$n = \frac{I_D}{I_D + I_R/N} \times 4$$

Where I_D and I_R represent the disk current and ring current, respectively, and N is the collection efficiency of the ring electrode (N = 0.37).

Referring to a previously reported method, the ring-disk current densities can be deconvoluted into two sequential reaction current densities: those corresponding to the reduction of oxygen to peroxide (O2P), and those corresponding to the subsequent reduction of peroxide to hydroxide (P2H)[23]. The method describes a stepwise oxygen reduction reaction (ORR) pathway, which is consistent with the current reaction occurring on the carbon electrode. The current densities for the O2P and P2H steps were then calculated using the following formulas:

$$j_{O2P} = j_D \times (\frac{\sigma N + 1}{2\sigma N})$$

$$j_{P2H} = j_D \times (\frac{\sigma N - 1}{2\sigma N})$$

Where j_D is the disk current density, σ is the disk-to-ring current density ratio.

- **New reference**

23. Yang, Q. et al. Selective O_2-to-H_2O_2 Electrosynthesis by a High-Performance, Single-Pass Electrofiltration System Using Ibuprofen-Laden CNT Membranes. *Environmental Science & Technology* **58**, 19058-19069 (2024).

2. To confirm the stability of the non-covalently assembled STAB molecules after long-term stability measurements, characterization using techniques such as post-reaction high-resolution XPS or FTIR is required, especially considering the highly alkaline local microenvironment generated during operation.

Response:

Thank you for this important suggestion regarding the stability of the non-covalently assembled STAB molecules under operating conditions. We agree that the highly alkaline local microenvironment generated during H_2O_2 electrosynthesis could potentially impair the integrity of the surfactant layer, and that post-reaction characterization is necessary to verify its durability.

- **Post-reaction XPS characterization confirms the stability of assembled STAB.**
  We have conducted high-resolution XPS analysis on the C18 cathode after long-term electrolysis (10 cycles, 30 h cumulative operation in Ca^{2+}-containing electrolyte). The post-reaction N1s spectrum is presented in the revised **Supplementary Information (Supplementary Fig. 76)**. The characteristic peak at approximately 403.0 eV, corresponding to the quaternary ammonium nitrogen ($-N^+(CH_3)_3$) of STAB, remains clearly detectable after extended operation. The N content retained approximately 70.3% of its initial content (from 2.73 at% to 1.92 at%), confirming that the majority of the STAB molecules remain assembled on the cathode surface despite prolonged exposure to the alkaline interfacial microenvironment and the applied electric field.
  We attribute this satisfactory stability to the strong electrostatic attraction between the positively charged quaternary ammonium headgroups and the negatively charged oxygen functional groups on the carbon surface, which provides a robust anchoring mechanism that withstands the operational conditions.

The Revised Content in Manuscript

To confirm the stability of the non-covalently assembled STAB molecules after long-term stability measurements, the following XPS analysis have been made to the manuscript and Supporting Information:

- **New discussion in Section "Enhanced Ca^{2+}-tolerance by a fenced cathode structure." [Line 575-584, Page 18 (Revised)]**
  Notably, the operational stability of the electrostatically assembled STAB layer was verified by post-reaction XPS analysis. After 10 cycles (30 h cumulative operation) of H_2O_2 electrosynthesis in Ca^{2+}-containing electrolyte, the characteristic N1s peak at ~403.0 eV corresponding to the quaternary ammonium nitrogen ($-N^+(CH_3)_3$) of STAB remained clearly detectable on the C18 cathode (**Supplementary Fig. 76**), with the N content retaining ~70.3% of its initial value (from 2.73 at% to 1.92 at%). This demonstrates that majority of STAB molecules remain anchored on the cathode surface despite prolonged exposure to the alkaline interfacial microenvironment and the applied electric field.

- **New Supplementary Fig. 76 in the Supplementary Information:**

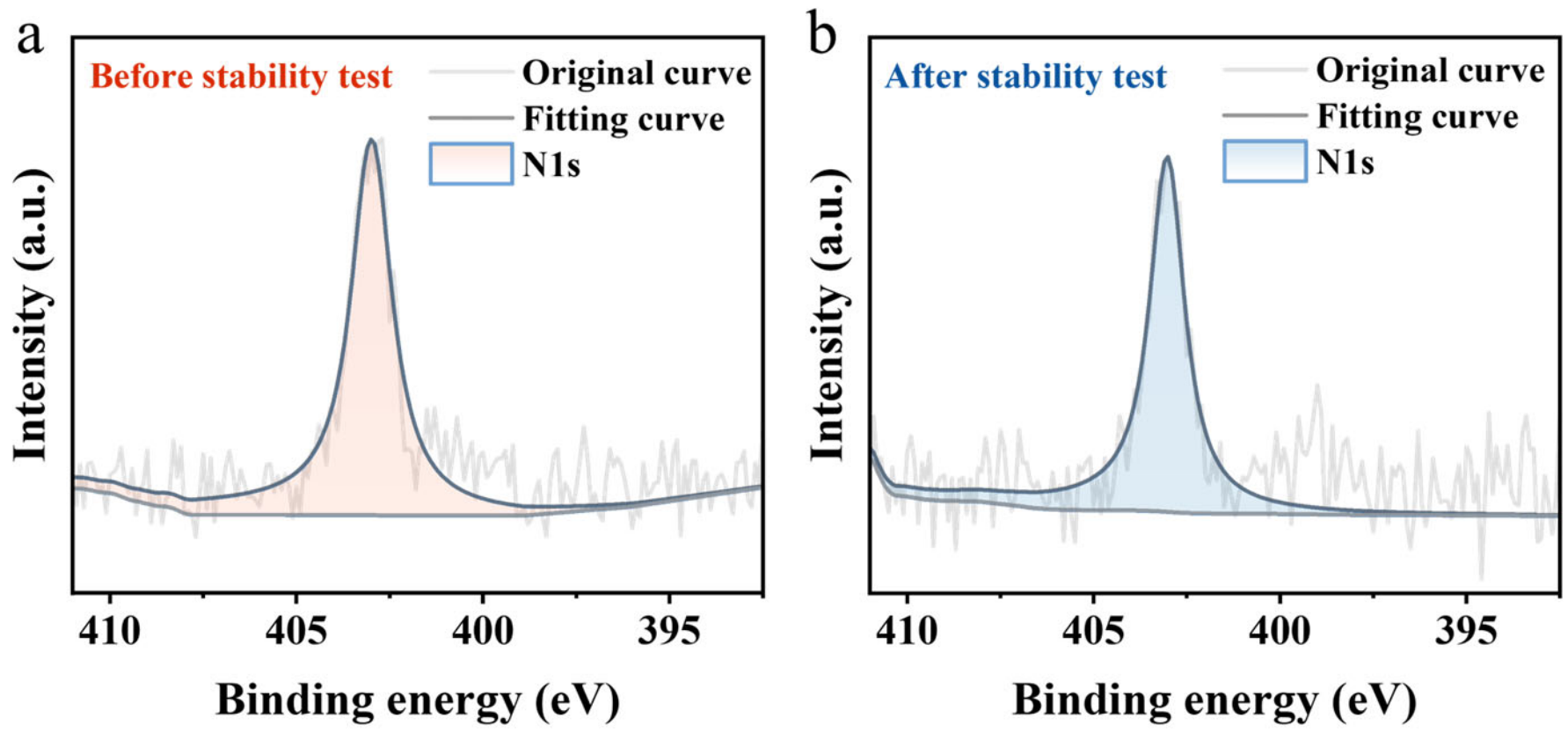

Supplementary Fig. 76 XPS high-resolution N1s spectra of C18 (a) before and (b) after stability measurements.

3. It is suggested to provide the calculated adsorption energies of key $2e^-$ ORR intermediates (e.g., *OOH) on the C18 surface with and without Ca^{2+}, which would fully validate the proposed thermodynamic shift from the $2e^-$ to the $4e^-$ pathway.

Response:

Thank you for this constructive suggestion regarding the computational validation of the proposed thermodynamic shift from the $2e^-$ to the $4e^-$ pathway. We appreciate this suggestion and would like to clarify our research framework and the complementary evidence we have provided.

- **(1) Research framework and rationale for using C0 as the model catalyst**

  In this work, C0 (oxygen-doped carbon black) was employed as the model catalyst to systematically investigate the inhibitory mechanism of dissolved Ca^{2+} on the $2e^-$ ORR. Based on the mechanistic insights gained from C0, we subsequently developed the STAB modification strategy (C18) and the 3D PUS-fenced architecture to enhance the Ca^{2+}-tolerance. Therefore, the fundamental mechanistic investigation–including the inhibitory effect of Ca^{2+} on the $2e^-$ ORR pathway–was conducted primarily on the C0 catalyst. Accordingly, we have supplemented the DFT calculations of the *OOH adsorption energy on the C0 surface with and without Ca^{2+}, using the reconstructed model incorporating both a C-O-C ether group and an adjacent carbon vacancy defect within the graphene monolayer.

- **(2) DFT-calculated *OOH adsorption energies on C0 surface**

  The *OOH is the key intermediate in ORR pathways and the calculated *OOH adsorption energy on the reconstructed C0 model is presented in new **Supplementary Fig. 14**. The results show that the *OOH binding strength is moderately enhanced in the Ca^{2+} system relative to the Na^+ system, suggesting that dissolved Ca^{2+} does not inhibit the $2e^-$ ORR pathway by suppressing the formation of *OOH.

- **(3) In-situ ATR-SEIRAS validation**

  Beyond DFT calculations, we have further performed in-situ attenuated total reflectance surface-enhanced infrared absorption spectroscopy (ATR-SEIRAS) measurements to directly probe the interfacial reaction intermediates and water structure under operando ORR conditions. The key findings are as follows:

- **(a) Ca^{2+} does not inhibit the O_2-to-H_2O_2 (O2P) step**

  The in-situ ATR-SEIRAS spectra reveal that the characteristic peak associated with the *OOH (~1100 cm^{-1}) intermediate–the key reaction intermediate of the $2e^-$ ORR pathway–is clearly observed in both the Na^+ and Ca^{2+} systems. Notably, the *OOH intensity in the Ca^{2+} system is even stronger than that in the Na^+ system, which is consistent with the DFT-calculated *OOH adsorption energies. This provides direct spectroscopic evidence that dissolved Ca^{2+} does not inhibit the O2P step by suppressing the formation of *OOH. This finding is fully consistent with our RRDE deconvolution results, which show that the kinetic current for the O2P step (j_{O2P}) is nearly identical in Na^+ and Ca^{2+} electrolytes.

- **(b) Ca^{2+} enhances interfacial water activation and proton availability**

  In contrast, the in-situ ATR-SEIRAS spectra show that the introduction of Ca^{2+} significantly enhances the intensities corresponding to $*H_2O$ (~1670 cm^{-1}) and O-H stretching vibrations (~3000-3700 cm^{-1}). To further elucidate the effect of Ca^{2+} on the

interfacial water structure, the O-H stretching band was deconvoluted into four-coordinated H-bonded water (4-HB-H_2O), two-coordinated H-bonded water (2-HB-H_2O), and free water. The fitting results reveal that Ca^{2+} increases the proportion of 2-HB-H_2O and free water, while decreasing the proportion of 4-HB-H_2O. This redistribution of interfacial water species can facilitate water dissociation and enhance proton availability, thereby promoting the parasitic H_2O_2-to-H_2O (P2H) reduction step.

- **(4) Summary of revisions**

  These spectroscopic findings converge with our DFT calculations (showing a lower *OOH adsorption energy and water dissociation energy barriers in the Ca^{2+} system) and RRDE measurements with deconvolution analysis (showing Ca^{2+}-enhanced j_{P2H} but unchanged j_{O2P} and the detailed supplementary experiments and analyses are provided in the response to Comment 1). These evidences consistently support the conclusion that dissolved Ca^{2+} suppresses net H_2O_2 electrosynthesis primarily by accelerating water dissociation and the further reduction of H_2O_2 to H_2O, rather than by inhibiting the O_2-to-H_2O_2 step.

The Revised Content in Manuscript

Based on your comments, we have provided the adsorption energies of *OOH based on DFT calculations and further conducted in-situ ATR-SEIRAS measurements to directly probe the interfacial reaction intermediates and water structure under operando conditions. The following specific revisions have been implemented in the manuscript and supporting information:

- **New Figures and Discussion in Section "Evidence for the suppression of 2e$^-$ ORR by dissolved Ca^{2+}." [Line 175-194, Page 6 (Revised)]**

  The reaction intermediates and interfacial water structures were directly probed using in situ electrochemical attenuated total reflection surface-enhanced infrared absorption spectroscopy (ATR-SEIRAS). Notably, the characteristic *OOH band (~1100 cm^{-1}) in the Ca^{2+} system was even stronger than that in the Na^+ system **(Fig. 1g and 1h)**, suggesting that dissolved Ca^{2+} does not impede the O2P step by inhibiting *OOH formation[24]. The reduced *OOH adsorption energy collectively indicates a stronger stabilizing effect of Ca^{2+} on *OOH intermediate (**Supplementary Fig. 14**). In contrast, the Ca^{2+} system demonstrated enhanced signals associated with *H_2O (~1670 cm^{-1}) and O-H stretching vibrations (~3000-3700 cm^{-1}) **(Fig. 1g and 1h)**, indicating that dissolved Ca^{2+} can enhance interfacial H_2O adsorption and activation. The O-H stretching modes can be further deconvoluted into tetrahedrally coordinated (4-HB-H_2O), doubly coordinated (2-HB-H_2O), and free water[25]. Among these species, 2-HB-H_2O is more favorable for water dissociation toward proton generation[26,27]. Compared with Na^+, Ca^{2+} markedly promoted the conversion of 4-HB-H_2O into 2-HB-H_2O and free water **(Fig. 1i and Supplementary Fig. 15)**. Consequently, Ca^{2+} is more favorable for interfacial water adsorption and dissociation, thereby enhancing proton availability and accelerating the further reduction of H_2O_2 to H_2O. These in situ spectroscopic results provide direct evidence that dissolved Ca^{2+} primarily affects the 2e$^-$ ORR selectivity by regulating interfacial water activation and proton availability, rather than by suppressing *OOH formation in the O_2-to-H_2O_2 pathway.

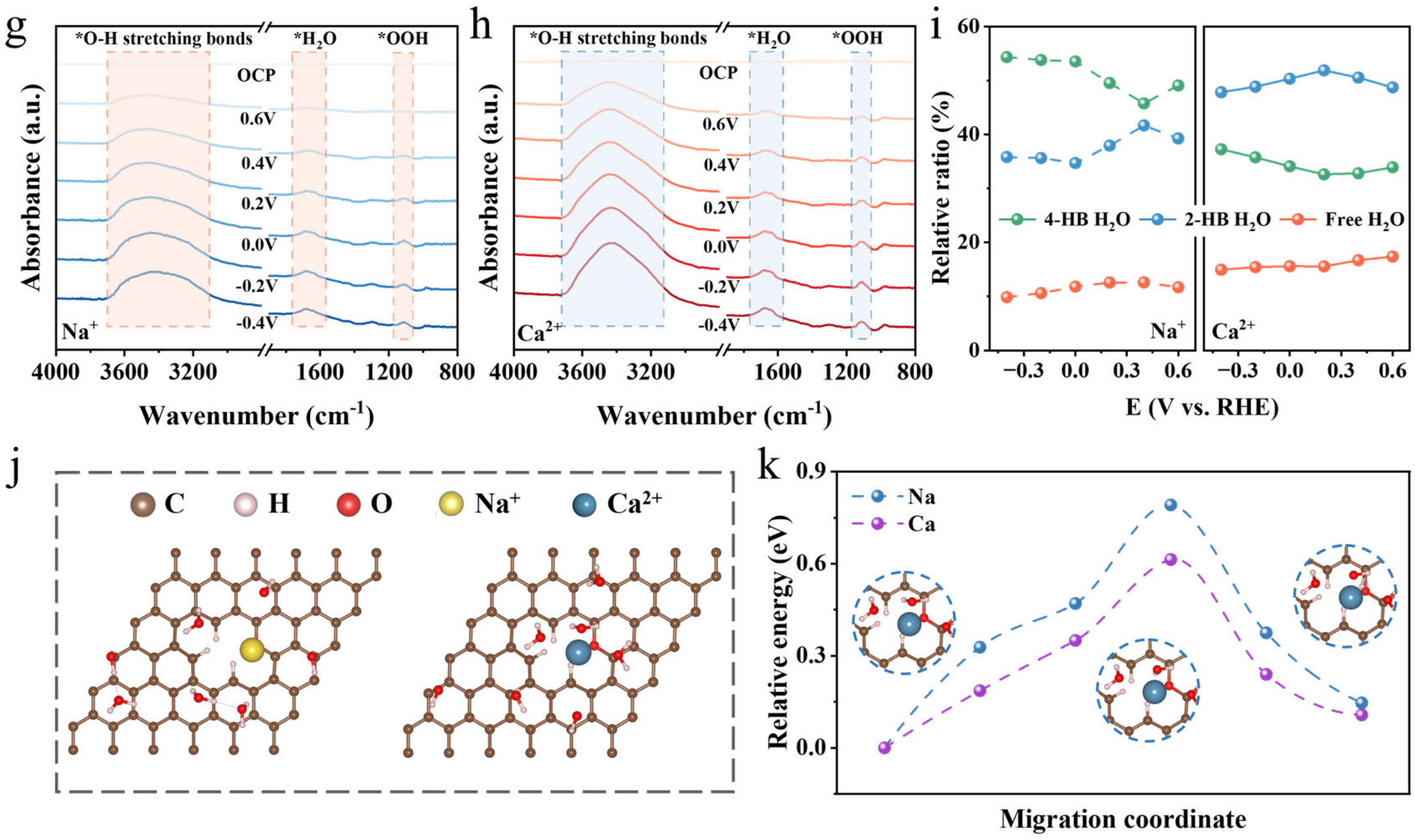

Fig. 1. Effects of dissolved Ca^{2+} on $2e^-$ ORR for oxygen-doped carbon black (named C0). In-situ ATR-SEIRAS spectra for C0 in O_2-saturated (g) 0.1M NaCl and (h) 0.1M $CaCl_2$. (i) The relative ratio of 4-HB-H_2O, 2-HB-H_2O and free H_2O for C0 in Na^+ and Ca^{2+} systems. (j) Theoretical models of the oxygen-doped graphene surface with Na^+ and Ca^{2+}. (k) Transition states and energy profiles of water dissociation process.

- **New Figures in Supplementary Information:**

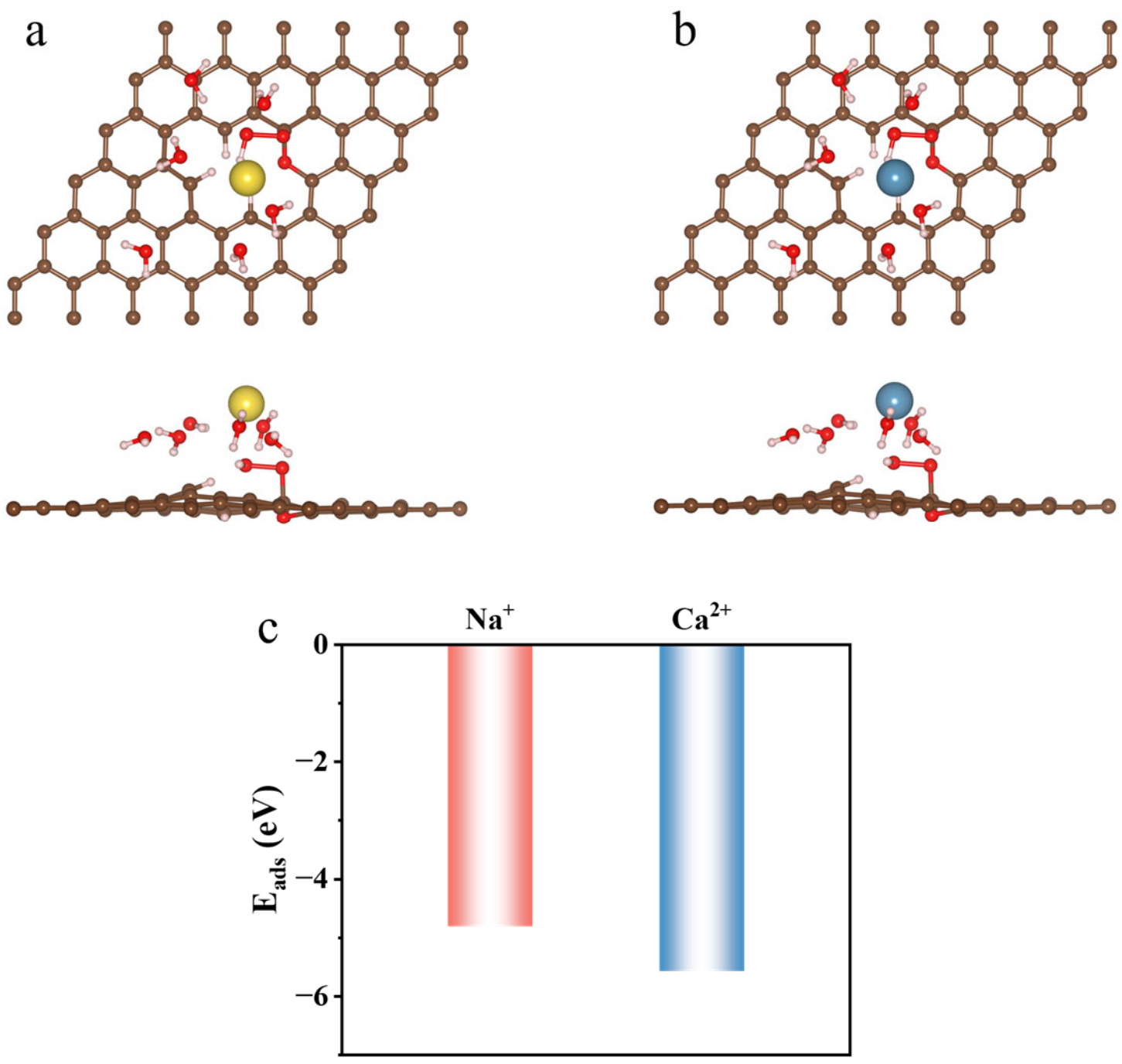

Supplementary Fig. 14. Atomic configurations of *OOH on the surface with (a) Na^+ and (b) Ca^{2+}. (c) The adsorption energies (E_{ads}) for *OOH on the graphene surfaces in Na^+ and Ca^{2+} systems. The brown, red, pink, yellow and blue spheres represent C, O, H, Na and Ca atoms, respectively.

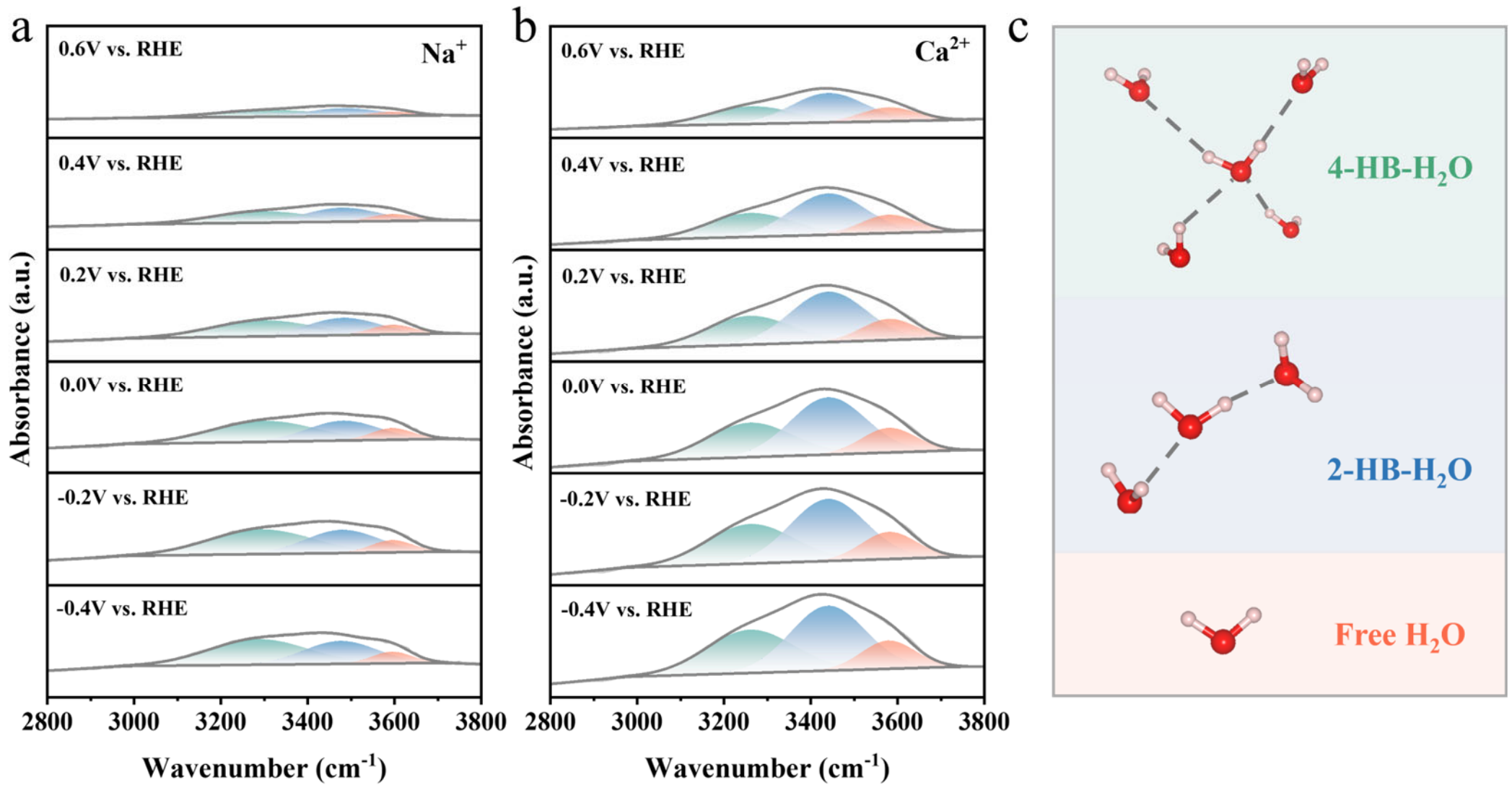

Supplementary Fig. 15. Deconvolution of O-H stretching band in ATR-SEIRAS spectra into 4-HB-H_2O, 2-HB-H_2O and free H_2O for C0 in (a) Na^+ and (b) Ca^{2+} systems. (c) Schematic illustration of the three interfacial water species.

- **Replaced Figures in Supporting Information:**
  Fig. 1h, 1i and 1j in the original manuscript have been moved to the **Supplementary Figs. 16, 17, and 19** in the original Supporting Information, and are now renumbered as **Supplementary Figs. 21, 22 and 24,** respectively.

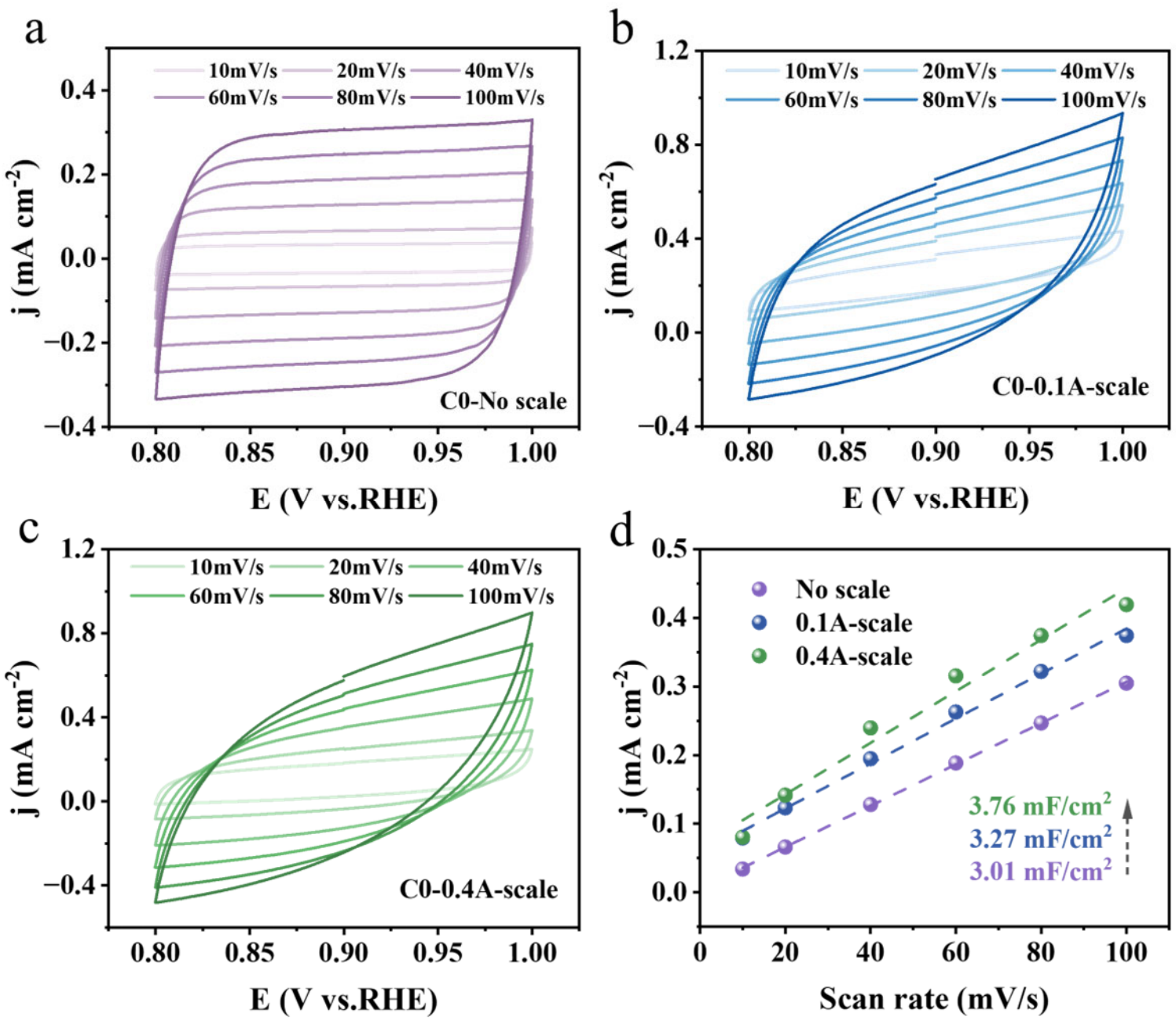

Supplementary Fig. 21. CV curves of the (a) No-scale, (b) 0.1A-scale and (c) 0.4A-scale C0 cathodes in N_2-saturated 0.1M Na_2SO_4 electrolyte for ECSA measurements. (d) Calculated ECSA of C0 cathodes with varying degrees of scaling.

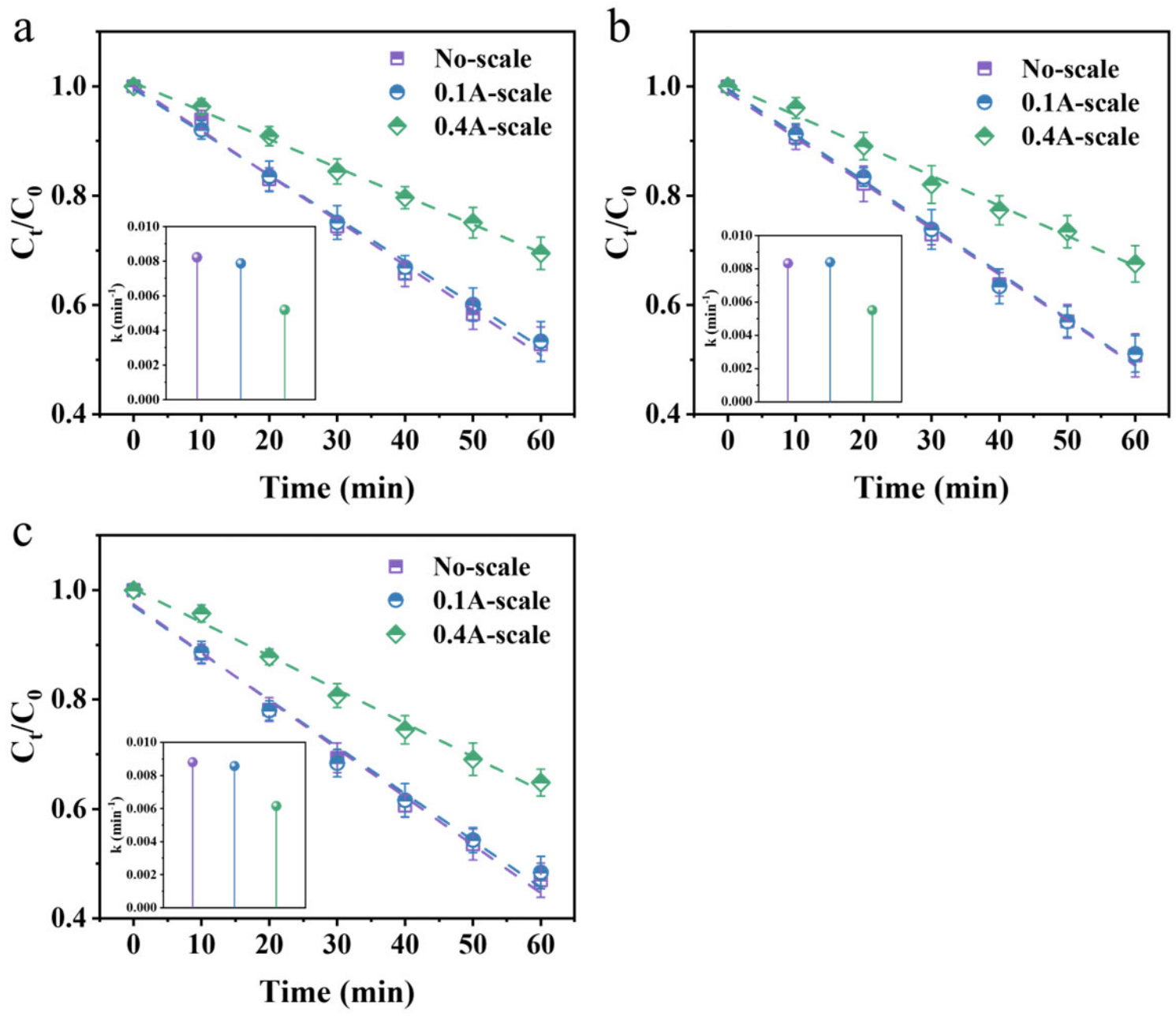

Supplementary Fig. 22. H_2O_2 decomposition curves and H_2O_2 decomposition rate constant k of C0 cathodes with varying degrees of scaling at (a) 0.1A, (b) 0.2A and (c) 0.4A with an initial H_2O_2 concentration of 1200 mg/L. Reaction conditions: [Na_2SO_4] = 0.05 M, a rotation speed of 600 rpm, an initial pH of 7, and electrolysis of 1h.

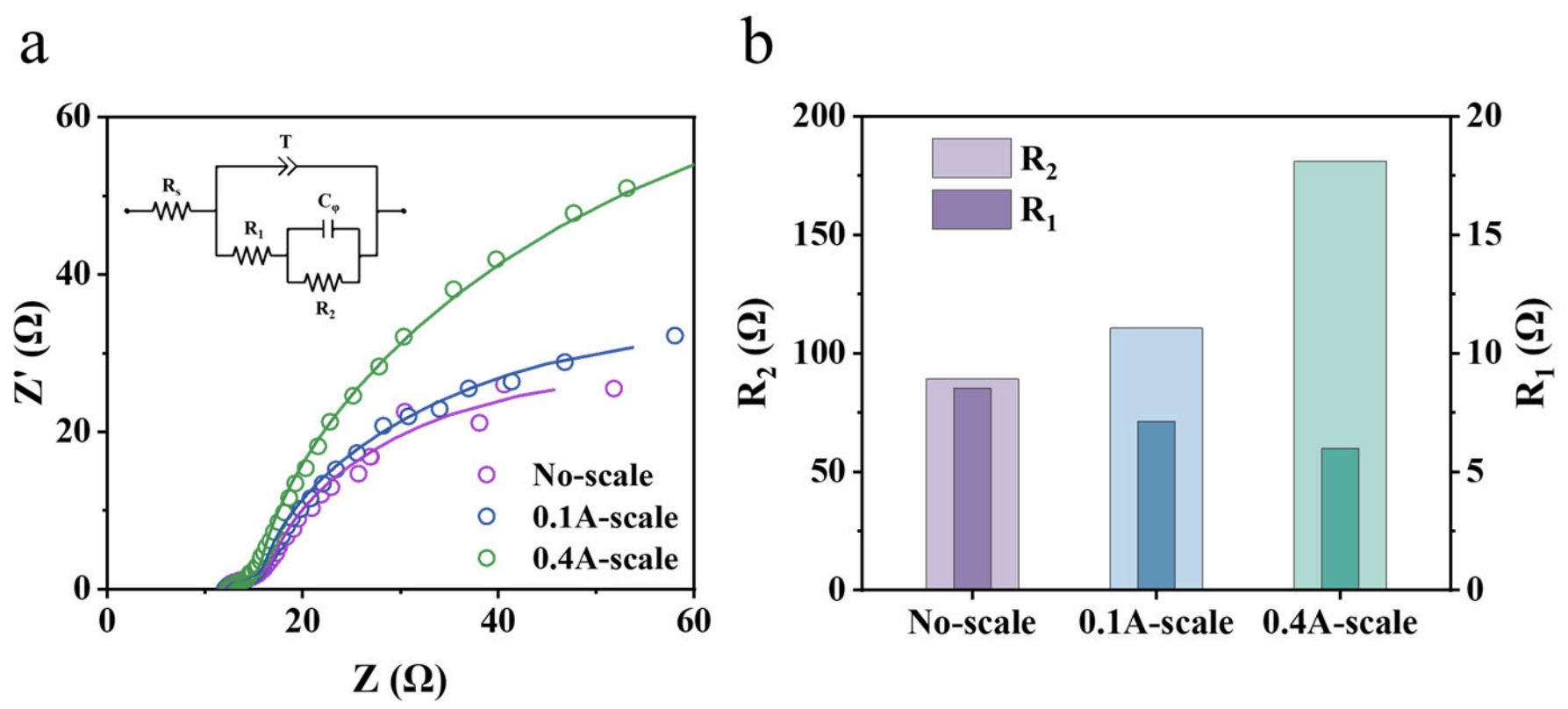

Supplementary Fig. 24. (a) EIS Nyquist plots and (b) impedance components of H_2O_2 reduction for C0 cathodes with varying degrees of scaling in N_2-saturated 0.1 M Na_2SO_4 solution containing 10 mM H_2O_2. R_1 and R_2 represent the charge transfer resistance and the hydrogen adsorption resistance, respectively.

- **Updated Methods Section**

  In-situ ATR-SEIRAS measurements [Line 801-815, Page 24 (Revised)]

  In-situ electrochemical ATR-SEIRAS measurements were performed using a Fourier transform infrared spectrometer (Nicolet iS50, Thermo Fisher Scientific) equipped with a liquid-nitrogen-cooled mercury cadmium telluride (MCT) detector. A Si ATR prism chemically coated with a thin Au film was used simultaneously as the optical waveguide and the conductive working-electrode substrate, onto which the catalyst ink

was uniformly deposited. A standard three-electrode configuration was employed for all tests, comprising the catalyst-modified Au/Si ATR electrode as the working electrode, an Ag/AgCl electrode as the reference electrode, and a Pt wire as the counter electrode. All electrochemical measurements were conducted in a dual-compartment H-cell reactor. The cathodic compartment was filled with O_2-saturated 0.1 M NaCl or 0.1 M $CaCl_2$ as the working electrolyte, while the anodic compartment contained 0.1 M Na_2SO_4. The ATR-SEIRAS spectra were recorded over a potential range from 0.6 to -0.4 V vs. RHE. The spectrum collected at the open-circuit potential was used as the background.

Density functional theory (DFT) calculations [Line 862-874, Page 26 (Revised)]

A water bi-layer was included to describe explicitly the polarization involved in water dissociation. The implicit solvation effects were implemented using the VASPsol package.

The electric field strength at the interface between the graphene surface and the electrolyte was evaluated using a parallel-plate capacitor model. The electric field strength (E) was calculated according to the following equation: $E = (Q_1+Q_2) / (2\varepsilon S)$, where Q_1 and Q_2 represent the net charges of the graphene slab and the electrolyte, respectively, as determined by Bader charge analysis; S represents the surface area of the graphene slab; and ε represents the absolute permittivity of water.

The adsorption energies of *OOH or Ca^{2+} on the surface of C0 or C18 were calculated by the formula: $E_{ad} = E_{total} - E_{base} - E_{adsorbate}$, where E_{total} is the total energy of the absorbed system, E_{base} is the basement energy and $E_{adsorbate}$ is the energy of adsorbate.

- **New reference**

24. Nie, J. et al. Accelerating water dissociation to achieve ampere-level hydrogen peroxide electrosynthesis in brine and seawater. *Nature Communications* **16**, 5895 (2025).
25. Wang, Y. et al. Differentiating interfacial water structures via alkali metal cation promotor for H_2O_2 electrosynthesis in acid. *Nature Communications* **17**, 4973 (2026).
26. Yu, Y. et al. Regulating active hydrogen supply and intermediate binding for pH-universal H_2O_2 electrosynthesis at ampere-level current density. *Nature Communications* **16**, 10784 (2025).
27. Guo, L. et al. Formate - Anion - Induced Water Network Reshaping Enables Concurrent Hydrogen and Magnesium Hydroxide Production From Seawater. *Angewandte Chemie International Edition* **65**, e6937994 (2026).

4. In light of the contact angle and ECSA measurements, the authors should provide a reasonable explanation for the performance differences observed between the significantly reduced active area (ECSA drops from 3.01 to 0.94 mF/cm^2) and the enhanced H_2O_2 yield of C18. A discussion on this trade-off is needed.

Response:

Thank you for this insightful comment, which allows us to clarify the apparent trade-off between the reduced ECSA and the enhanced H_2O_2 electrosynthesis performance of the C18 cathode in the absence of Ca^{2+}.

As the reviewer noted, the ECSA of C18 (0.94 mF/cm^2) is indeed lower than that of C0 (3.01 mF/cm^2), which is an expected consequence of the hydrophobic alkyl chains introduced by STAB self-assembly. However, we wish to emphasize that the H_2O_2 electrosynthesis performance is governed by multiple factors, and the ECSA represents only the total electrochemically accessible area rather than the specific activity toward the desired 2e$^-$ ORR pathway. The enhanced H_2O_2 yield of C18, despite the reduced ECSA, can be rationally explained by the following mechanisms:

- **(1) Enhanced oxygen mass transport counterbalances active site loss.**
  For electrochemical reactions involving gaseous reactants, maintaining an efficient triple-phase interface (solid-liquid-gas) is critically important. The significantly enhanced hydrophobicity of C18 (contact angle increasing from 59.34° to 132.67°) can suppress water flooding of the gas diffusion electrode (**Supplementary Figs. 15** and **44**), establishing a robust triple-phase interface. This facilitates oxygen transport to the active sites, partially compensating for the negative impact of reduced active area, and ultimately enhances the H_2O_2 electrosynthesis performance.
- **(2) Suppressed parasitic H_2O_2 reduction compensates for the lower active area.**
  The significantly enhanced hydrophobicity of C18 facilitates the rapid desorption and diffusion of in-situ generated H_2O_2 from the cathode surface into the bulk solution, thereby effectively suppressing the parasitic reduction of H_2O_2 to H_2O at the cathode. As shown in the **Fig. 1i** and **Fig. 3l**, the H_2O_2 decomposition kinetic constant of C18 at 0.1A is only 0.00637 min^{-1}, representing a 22.5% reduction compared to that of C0 (0.00822 min^{-1}). This result further proves the remarkable advantage of the C18 cathode in inhibiting H_2O_2 reduction, thereby enhancing the overall selectivity of the 2e$^-$ ORR.

The Revised Content in Manuscript

To clarify the apparent trade-off between the reduced ECSA and the enhanced H_2O_2 electrosynthesis, the following specific additions have been made to the manuscript:

- **New discussion in Section "2e$^-$ ORR performance and scale formation of surfactant-modified cathodes in Ca^{2+}-containing electrolytes." [Line 336-341, Page 11 (Revised)]**
  In the absence of Ca^{2+}, C18 exhibited higher H_2O_2 electrosynthesis performance (**Supplementary Fig. 30**) despite its significantly reduced ECSA (0.94 vs. 3.01 mF/cm^2 for C0). This can be attributed to the enhanced hydrophobicity of C18, which facilitates oxygen mass transport by mitigating water flooding[42], and simultaneously promotes the desorption and diffusion of in-situ generated H_2O_2 into the bulk solution, thereby suppressing its parasitic reduction to H_2O[43], as will be discussed in detail below.

5. A photograph of the 3D PUS-fenced electrolyzer during actual operation should be provided, as beyond experimental data, such visual documentation holds significant referential value.

Response:

Thank you for this valuable suggestion to provide visual documentation of the 3D PUS-fenced reactor during actual operation. We fully agree that such photographic evidence can offer intuitive and instructive reference for readers.

As requested, we have added photographs of the 3D PUS-fenced reactor in operation to the revised Supplementary Information **(Supplementary Fig. 78)**. The image clearly shows the configuration of the PUS-fenced cathode assembly and the overall experimental setup during H_2O_2 electrosynthesis. Additionally, we have incorporated a detailed description of the operational setup for the 3D PUS-fenced reactor in the Methods section for clarity.

The Revised Content in Manuscript

- **New Supplementary Fig. 78 in the Supplementary Information:**

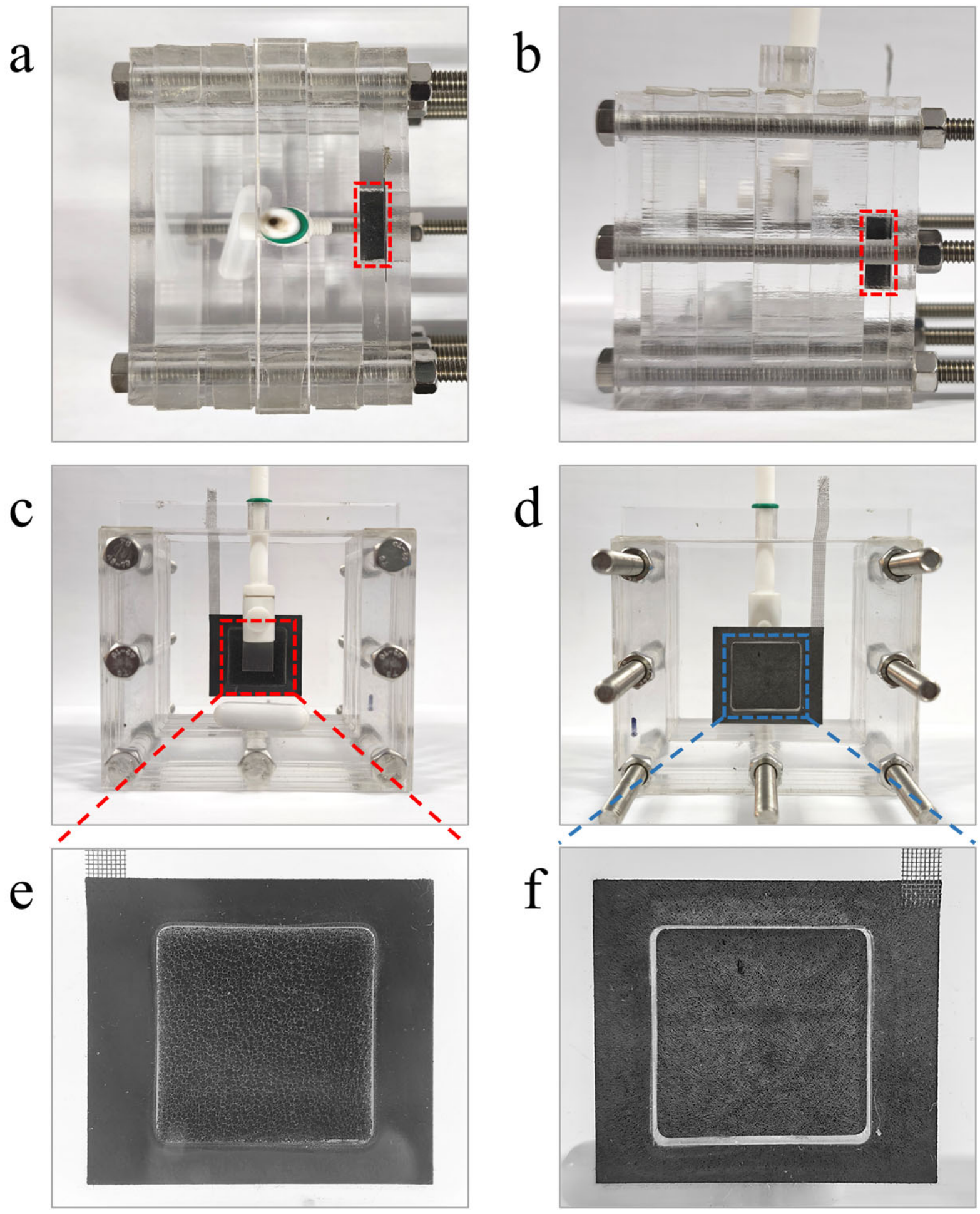

Supplementary Fig. 78 Photograph of the 3D PUS-fenced reactor for H_2O_2 electrosynthesis. (a) Top view, (b) side view, (c) front view, and (d) back view of the3D PUS-fenced reactor. (e) Enlarged view of the PUS structure and (f) the air-diffusion cathode. The structure enclosed by the red dashed box corresponds to the PUS.

- **Updated Methods Section of "H_2O_2 electrosynthesis in GDEs" [Line 691-696, Page 22 (Revised)]**

  The detailed description of the operational setup for the 3D PUS-fenced reactor now clearly reads:

  "The fundamental modification from 2D planar to 3D fenced cathode architecture involved only the incorporation of a PUS (20ppi, 30ppi, 40ppi, 60ppi; 20×20×5 mm) positioned in direct contact with the cathode, preserving identical experimental conditions otherwise. The 3D PUS fenced cathode configuration was illustrated in **Fig. 5a** and **Supplementary Fig. 78**."

Modifications to comments from Reviewer #2

General comments

This manuscript investigates the roles of calcium ions and scaling in the electrosynthesis of hydrogen peroxide within natural water systems. The authors propose the perspective that "dissolved Ca^{2+} is harmful, whereas precipitated calcium scale is actually beneficial." This topic demonstrates a degree of novelty and possesses the potential to challenge conventional wisdom. However, after thoroughly reviewing the main text and supplementary information, I believe the core issue with the current manuscript is that the strength of the existing evidence is insufficient to support such strong conclusions. Specifically, key issues regarding the interfacial microenvironment, the pathways of further H_2O_2 reduction, the representativeness of long-term operation, and the credibility of the theoretical calculations remain unconvincingly resolved.

Specific comments

1. The authors' core conclusion is that dissolved Ca^{2+} promotes interfacial water dissociation and shifts the reaction pathway toward the $4e^-$ ORR, whereas precipitated calcium scale acts as a local alkaline or proton barrier that inhibits the further reduction of H_2O_2, thereby enhancing $2e^-$ ORR selectivity. However, this proposed mechanism currently relies primarily on electrochemical impedance spectroscopy (EIS) fitting parameters, H_2O_2 decomposition experiments, contact angle measurements, and theoretical calculations. It lacks direct evidence to conclusively substantiate this core claim, such as the characterization of the local interfacial pH microenvironment, changes in proton accessibility, or in-situ/operando monitoring of reaction intermediates.

2. In lines 531-538 of the main text, the authors claim: "The hybrid strategy delivered H_2O_2 electrosynthesis performance in four real water matrices… and maintained 96% of its initial efficacy after 30 h of cyclic testing, highlighting its robust stability. In conclusion, this work… establishing a new paradigm for designing efficient and stable electrocatalytic systems in natural water." However, almost all experimental evidence supporting the "beneficial scale" mechanism is based on extremely early-stage scaling conditions (only 3 hours of electrolysis at a relatively low current density of 0.1 A). Furthermore, the claimed 30-hour stability is merely an intermittent cyclic test (10 cycles × 3 hours each), not continuous operation. More importantly, the authors themselves observed scale-induced electrode water flooding (Figs. S15 and S44). In actual long-term continuous operation, this flooding and the continuous thickening of the scale layer will inevitably lead to the complete collapse of the three-phase boundary. Therefore, the current experimental results are completely insufficient to support the conclusion that "calcium scale remains beneficial and stable during long-term operation." Unless a single, continuous long-term test of at least 100 hours is provided, the claim of "efficient and stable in natural water" must be considered a severe overestimation.

3. The authors explicitly point out in the text (Lines 173-179) and SI (Figs. S14, S15, S42, S44) that the formation of calcium scale transitions the electrode surface from hydrophobic to hydrophilic, triggering severe water flooding and inhibiting oxygen

transport. Additionally, the ECSA and surface roughness change significantly with the degree of scaling. In Gas Diffusion Electrodes (GDEs), the kinetics and selectivity of the $2e^-$ ORR are extremely sensitive to the gas-liquid distribution at the three-phase boundary and the local oxygen concentration. Since scaling has already triggered such severe mass transport limitations and interfacial physical changes, the attenuation or fluctuation in electrode performance is entirely dominated by these physical factors. However, when discussing the core mechanism, the authors repeatedly attribute this phenomenon to dissolved Ca^{2+} promoting interfacial water dissociation and shifting the reaction pathway toward the $4e^-$ ORR. The current experimental design fails to decouple the 'physical effects caused by deteriorated mass transport/wettability' from the 'chemical catalytic effects of the scale barrier.' Without ruling out the severe disruption of the three-phase boundary as a dominant confounding factor, this mechanistic hypothesis remains unconvincing."

4. The manuscript attempts to demonstrate that dissolved Ca^{2+} promotes the further consumption of H_2O_2, while calcium scale inhibits this parasitic reaction, by relying on H_2O_2 decomposition curves, reduction polarization, and impedance analysis. However, these experiments represent oversimplified conditions that are not equivalent to the real ORR environment where H_2O_2 is generated dynamically under continuous oxygen supply and local alkalinization.

5. The authors employ theoretical calculations to demonstrate the core hypothesis that "dissolved Ca^{2+} accelerates interfacial water dissociation." However, the constructed theoretical model is unreasonable. First, the authors selected standard single-layer graphene as the model, completely ignoring the crucial influence of the abundant oxygen-containing functional groups and defect sites present on the surface of the actual catalyst used (oxygen-doped carbon black, C0). Second, as a core step in the electrochemical reaction, the calculation of the water dissociation energy barrier ignores the critical effects of the applied potential and the interfacial electric field. Consequently, the obtained energy barrier data lack credibility.

6. In the latter half of the manuscript, the authors introduce a C18 surfactant modification and a 3D-PUS sponge fenced strategy to demonstrate the engineering application value of their mechanism. However, the introduction of these two strategies adds more variables to the electrochemical reaction, leading to a somewhat confused core logical chain. The C18 modification (with the introduction of long alkyl chains) not only alters the Zeta potential but significantly changes the electrode's wettability. The authors must provide control experiments to decouple the effects of improved physical mass transport from electrostatic repulsion, rather than attributing the performance enhancement solely to the repulsion of Ca^{2+}. Furthermore, regarding the 3D-PUS strategy, the physical barrier of the sponge inherently hinders OH^- diffusion and elevates local pH, an effect that typically improves $2e^-$ ORR selectivity in other systems anyway (even in the absence of calcium). Overall, the engineering optimization in the latter half fails to further solidify the fundamental chemical mechanism proposed in the first half; instead, by mixing in additional variables, it blurs the main storyline of the manuscript.

7. Although the title, abstract, and conclusions heavily frame this work around

applications in "natural water," the primary experimental conditions presented in the main text and supplementary materials indicate that the vast majority of the experiments were actually conducted in simplified model systems (e.g., 0.05 M Na_2SO_4 with added Ca^{2+}, or NaCl/$CaCl_2$). Real natural water matrices are highly complex, involving the simultaneous and interactive presence of not only Ca^{2+} but also Mg^{2+}, bicarbonate, natural organic matter, sulfate, chloride, and other constituents. By attributing nearly all performance variations solely to calcium ions without clearly delineating the boundaries of applicability, the manuscript's claim of "universal applicability in natural water" is overstated.

1. The authors' core conclusion is that dissolved Ca^{2+} promotes interfacial water dissociation and shifts the reaction pathway toward the 4e$^-$ ORR, whereas precipitated calcium scale acts as a local alkaline or proton barrier that inhibits the further reduction of H_2O_2, thereby enhancing 2e$^-$ ORR selectivity. However, this proposed mechanism currently relies primarily on electrochemical impedance spectroscopy (EIS) fitting parameters, H_2O_2 decomposition experiments, contact angle measurements, and theoretical calculations. It lacks direct evidence to conclusively substantiate this core claim, such as the characterization of the local interfacial pH microenvironment, changes in proton accessibility, or in-situ/operando monitoring of reaction intermediates.

Response:

Thank you for this constructive and insightful comment. We fully acknowledge that our original mechanistic claims relied primarily on indirect evidence from EIS fitting, H_2O_2 decomposition experiments, and theoretical calculations. In response, we have now conducted in-situ attenuated total reflectance surface-enhanced infrared absorption spectroscopy (ATR-SEIRAS) measurements to directly probe the interfacial reaction intermediates and water structure under operando conditions. These new results provide direct evidence that supports our core mechanistic claims.

- **(1) Ca^{2+} does not inhibit the O_2-to-H_2O_2 (O2P) step**
  The in-situ ATR-SEIRAS spectra reveal that the characteristic peak associated with the *OOH (~1100 cm^{-1}) intermediate–the key reaction intermediate of the 2e$^-$ ORR pathway–is clearly observed in both the Na^+ and Ca^{2+} systems. Notably, the *OOH intensity in the Ca^{2+} system is even slightly stronger than that in the Na^+ system. This provides direct spectroscopic evidence that dissolved Ca^{2+} does not inhibit the O2P step by suppressing the formation of *OOH. This finding is fully consistent with our RRDE deconvolution results, which show that the kinetic current for the O2P step (j_{O2P}) is nearly identical in Na^+ and Ca^{2+} electrolytes.
- **(2) Ca^{2+} enhances interfacial water activation and proton availability**
  In contrast, the in-situ ATR-SEIRAS spectra show that the introduction of Ca^{2+} significantly enhances the intensities corresponding to *H_2O (~1670 cm^{-1}) and O-H stretching vibrations (~3000-3700 cm^{-1}). To further elucidate the effect of Ca^{2+} on the interfacial water structure, the O-H stretching band was deconvoluted into four-coordinated H-bonded water (4-HB-H_2O), two-coordinated H-bonded water (2-HB-H_2O), and free water. The fitting results reveal that Ca^{2+} increases the proportion of 2-HB-H_2O and free water, while decreasing the proportion of 4-HB-H_2O. This redistribution of interfacial water species can facilitate water dissociation and enhance proton availability, thereby promoting the parasitic H_2O_2-to-H_2O (P2H) reduction step.
- **(3) Spectroscopic evidence corroborates the central mechanistic claim**
  These spectroscopic findings converge with our DFT calculations (showing a lower water dissociation energy barriers in the Ca^{2+} system and the detailed supplementary calculations and analyses are provided in the response to Comment 5) and RRDE measurements with deconvolution analysis (showing Ca^{2+}-enhanced j_{P2H} but unchanged j_{O2P} and the detailed supplementary experiments and analyses are provided in the response to Comment 4). These evidences consistently support the conclusion

that dissolved Ca^{2+} suppresses net H_2O_2 electrosynthesis primarily by accelerating water dissociation and the further reduction of H_2O_2 to H_2O, rather than by inhibiting the O_2-to-H_2O_2 step.

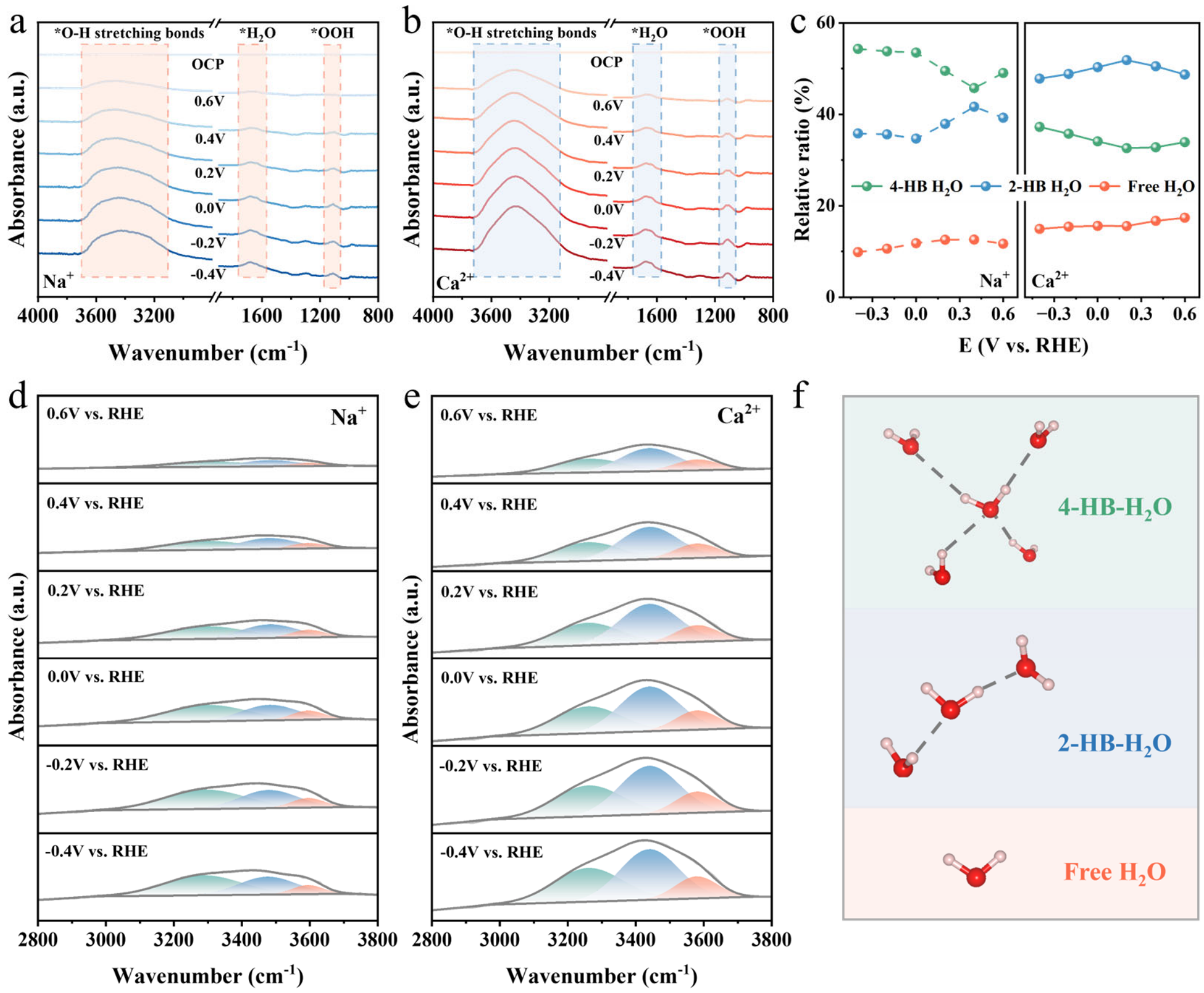

Figure In-situ ATR-SEIRAS measurements for C0. In-situ ATR-SEIRAS spectra for C0 in O_2-saturated (a) 0.1M NaCl and (b) 0.1M $CaCl_2$. (c) The relative ratio of 4-HB-H_2O, 2-HB-H_2O and free H_2O for C0 in different systems. Deconvolution of O-H stretching region into 4-HB-H_2O, 2-HB-H_2O and free H_2O for C0 in (d) Na^+ and (e) Ca^{2+} systems. (f) Schematic illustration of the three interfacial water species.

The Revised Content in Manuscript

Based on your comments, we have conducted in-situ ATR-SEIRAS measurements to directly probe the interfacial reaction intermediates and water structure under operando conditions. The following specific revisions have been implemented in the manuscript and supporting information:

- **New Figures and Discussion in Section "Evidence for the suppression of $2e^-$ ORR by dissolved Ca^{2+}." [Line 175-194, Page 6 (Revised)]**

The reaction intermediates and interfacial water structures were directly probed using in situ electrochemical attenuated total reflection surface-enhanced infrared absorption spectroscopy (ATR-SEIRAS). Notably, the characteristic *OOH band (~1100 cm^{-1}) in the Ca^{2+} system was even stronger than that in the Na^+ system **(Fig. 1g and 1h)**, suggesting that dissolved Ca^{2+} does not impede the O2P step by inhibiting *OOH formation[24]. The reduced *OOH adsorption energy collectively indicates a stronger

stabilizing effect of Ca^{2+} on *OOH intermediate (**Supplementary Fig. 14**). In contrast, the Ca^{2+} system demonstrated enhanced signals associated with *H_2O (~1670 cm^{-1}) and O-H stretching vibrations (~3000-3700 cm^{-1}) **(Fig. 1g and 1h)**, indicating that dissolved Ca^{2+} can enhance interfacial H_2O adsorption and activation. The O-H stretching modes can be further deconvoluted into tetrahedrally coordinated (4-HB-H_2O), doubly coordinated (2-HB-H_2O), and free water[25]. Among these species, 2-HB-H_2O is more favorable for water dissociation toward proton generation[26,27]. Compared with Na^+, Ca^{2+} markedly promoted the conversion of 4-HB-H_2O into 2-HB-H_2O and free water **(Fig. 1i** and **Supplementary Fig. 15)**. Consequently, Ca^{2+} is more favorable for interfacial water adsorption and dissociation, thereby enhancing proton availability and accelerating the further reduction of H_2O_2 to H_2O. These in situ spectroscopic results provide direct evidence that dissolved Ca^{2+} primarily affects the 2e$^-$ ORR selectivity by regulating interfacial water activation and proton availability, rather than by suppressing *OOH formation in the O_2-to-H_2O_2 pathway.

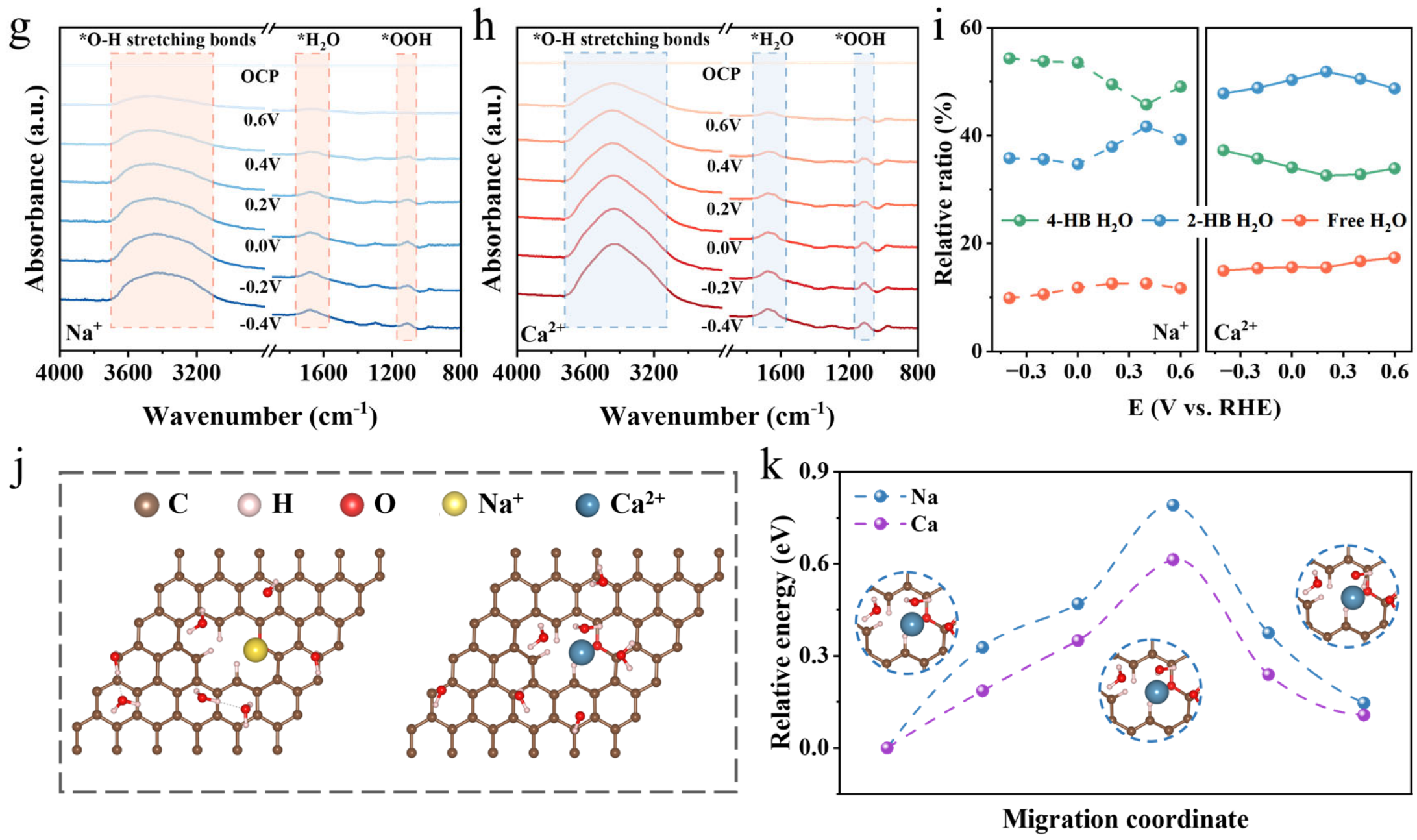

Fig. 1. Effects of dissolved Ca^{2+} on 2e$^-$ ORR for oxygen-doped carbon black (named C0). In-situ ATR-SEIRAS spectra for C0 in O_2-saturated (g) 0.1M NaCl and (h) 0.1M $CaCl_2$. (i) The relative ratio of 4-HB-H_2O, 2-HB-H_2O and free H_2O for C0 in Na^+ and Ca^{2+} systems. (j) Theoretical models of the oxygen-doped graphene surface with Na^+ and Ca^{2+}. (k) Transition states and energy profiles of water dissociation process.

- **New Supplementary Fig. 15 in Supplementary Information:**

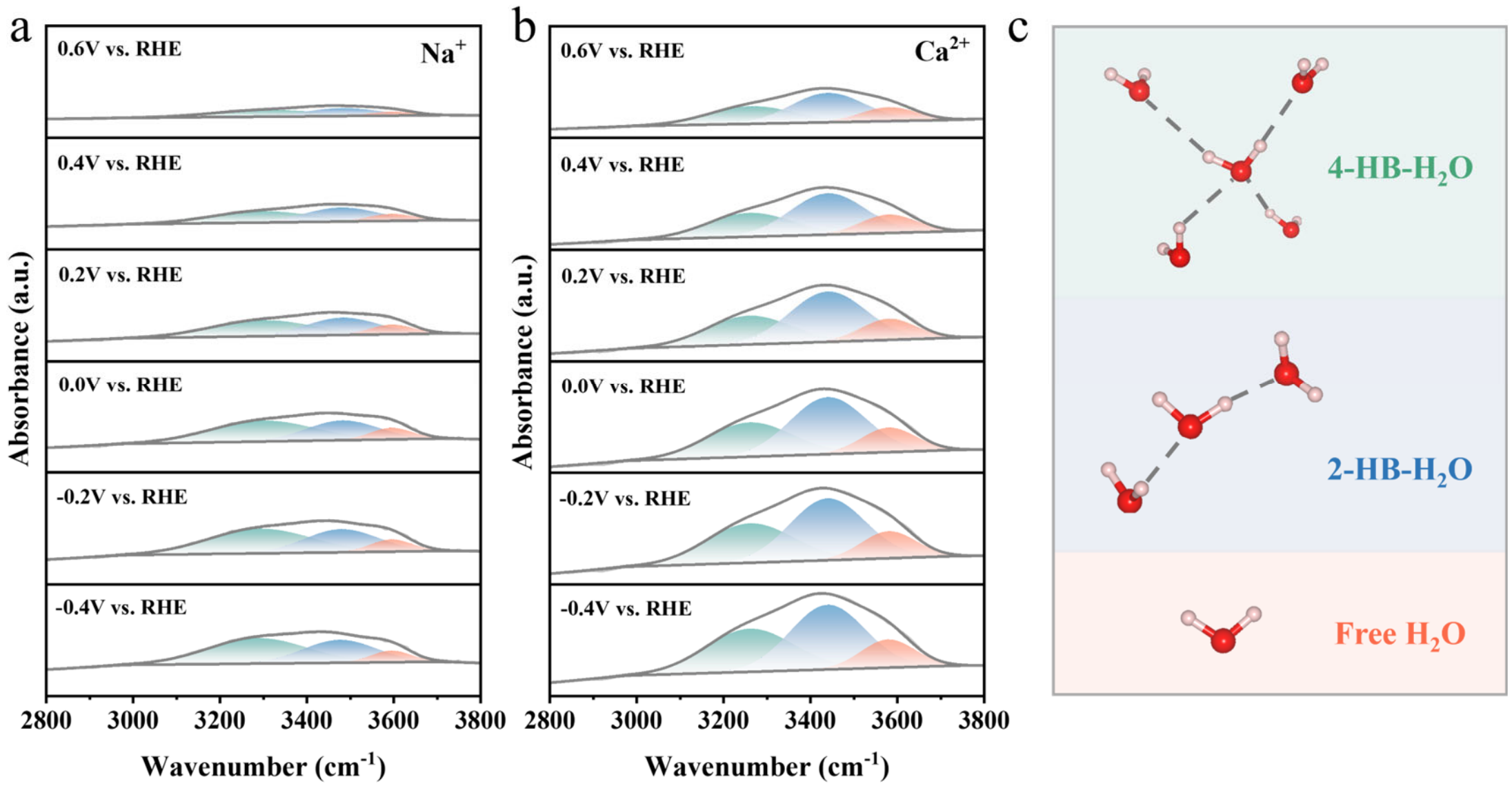

Supplementary Fig. 15. Deconvolution of O-H stretching band in ATR-SEIRAS spectra into 4-HB-H_2O, 2-HB-H_2O and free H_2O for C0 in (a) Na^+ and (b) Ca^{2+} systems. (c) Schematic illustration of the three interfacial water species.

- **Replaced Figures in Supplementary Information:**
  Fig. 1h, 1i and **1j** in the original manuscript have been moved to the **Supplementary Figs. 16, 17,** and **19** in the original Supplementary Information, and are now renumbered as **Supplementary Figs. 21, 22** and **24,** respectively.

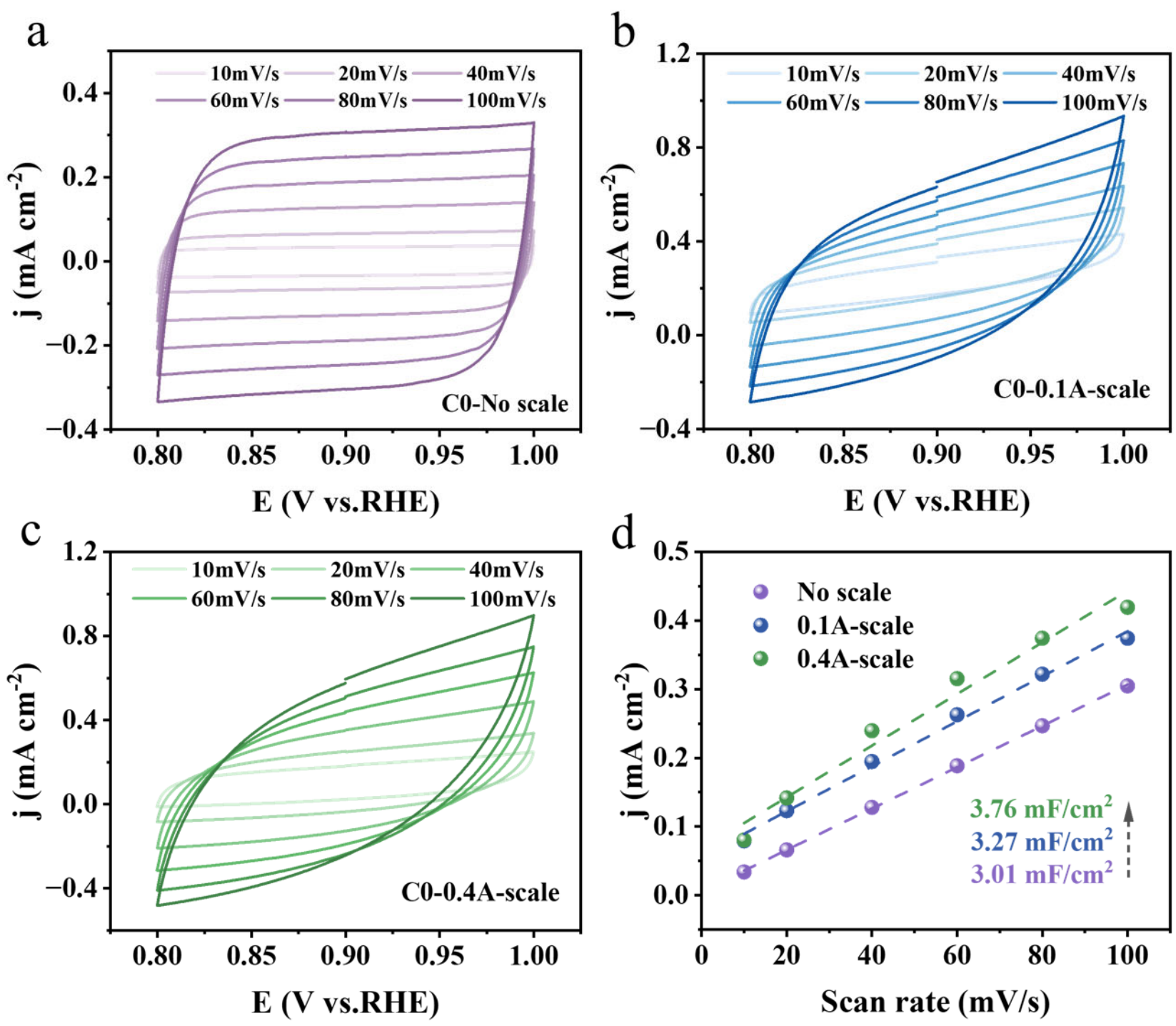

Supplementary Fig. 21. CV curves of the (a) No-scale, (b) 0.1A-scale and (c) 0.4A-scale C0 cathodes in N_2-saturated 0.1M Na_2SO_4 electrolyte for ECSA measurements. (d) Calculated ECSA of C0 cathodes with varying degrees of scaling.

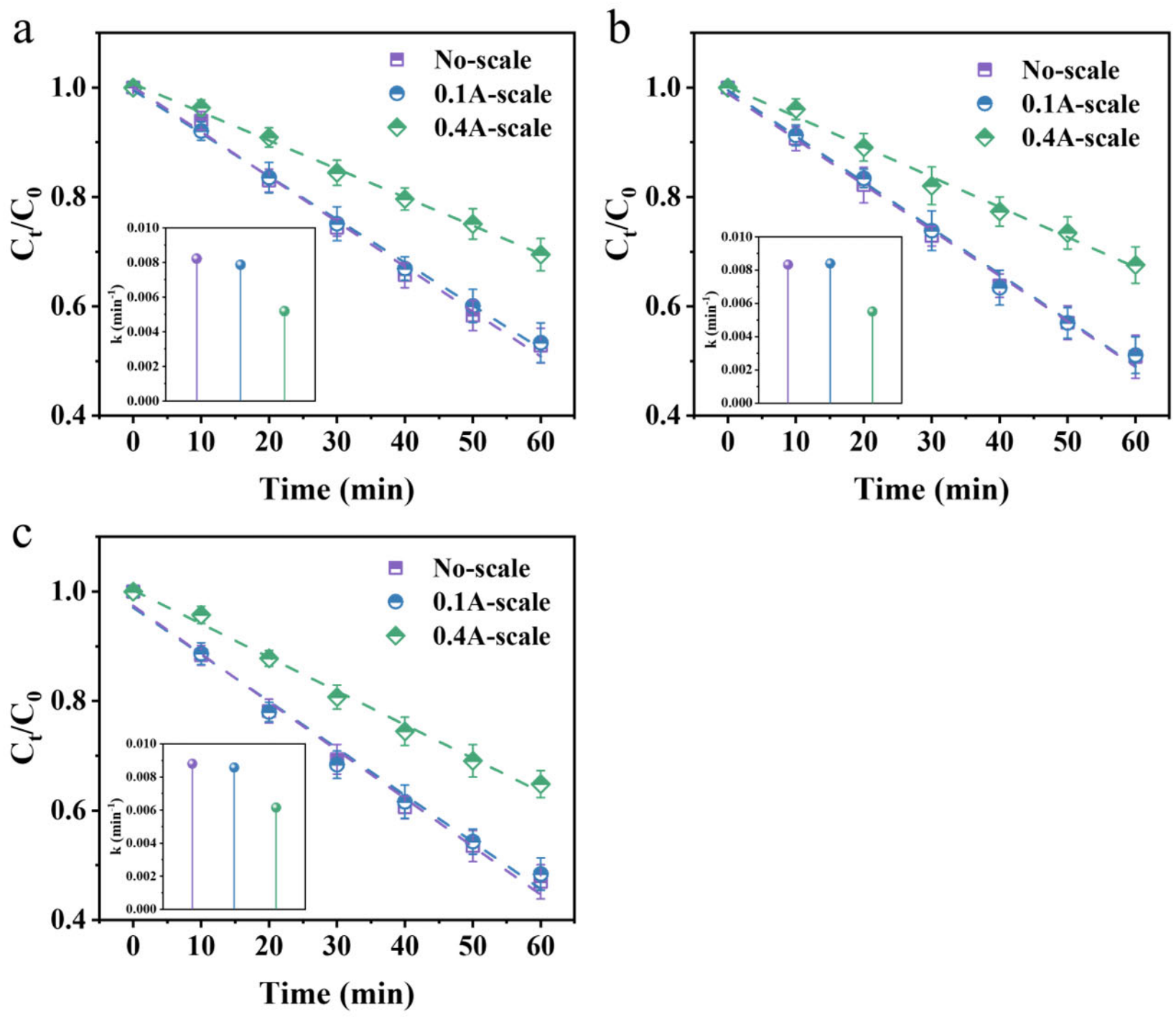

Supplementary Fig. 22. H_2O_2 decomposition curves and H_2O_2 decomposition rate constant k of C0 cathodes with varying degrees of scaling at (a) 0.1A, (b) 0.2A and (c) 0.4A with an initial H_2O_2 concentration of 1200 mg/L. Reaction conditions: [Na_2SO_4] = 0.05 M, a rotation speed of 600 rpm, an initial pH of 7, and electrolysis of 1h.

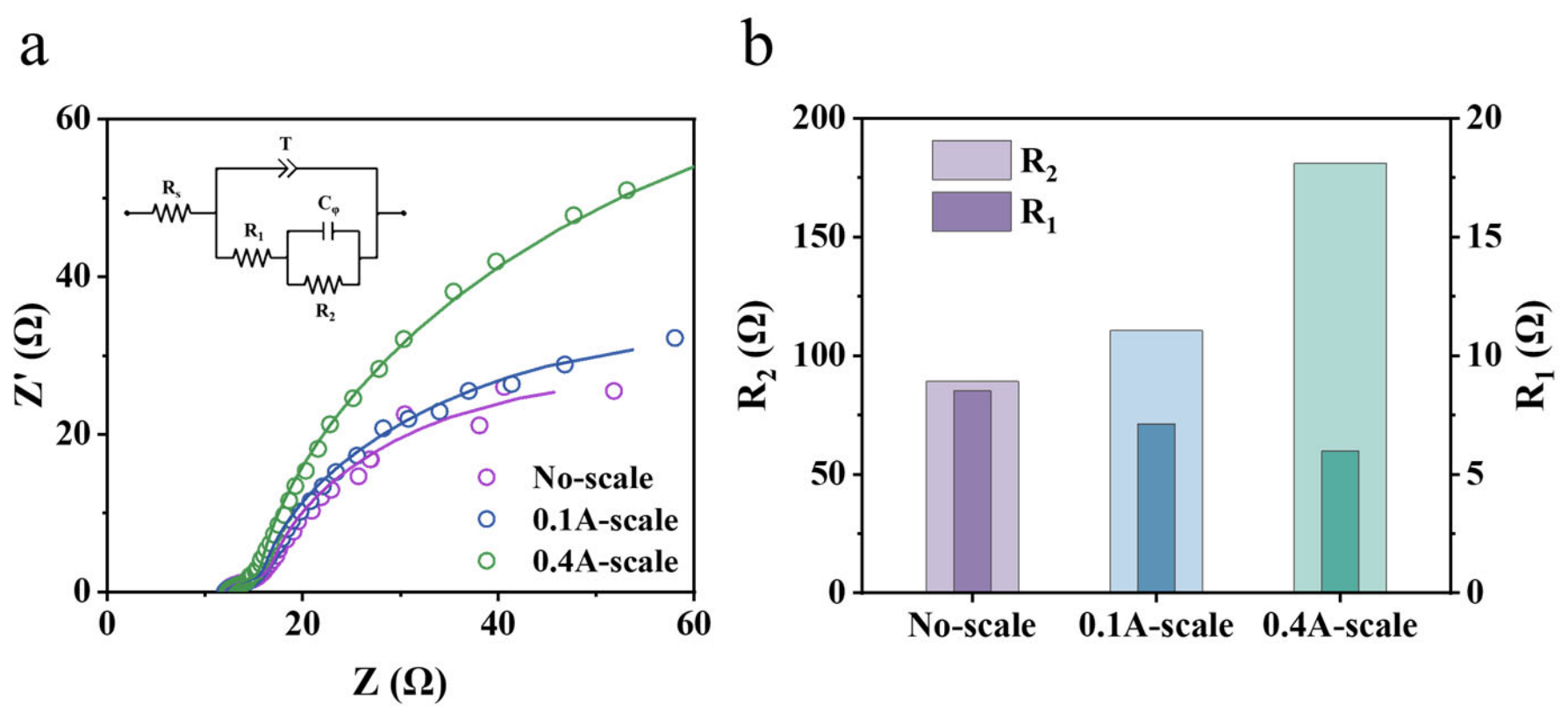

Supplementary Fig. 24. (a) EIS Nyquist plots and (b) impedance components of H_2O_2 reduction for C0 cathodes with varying degrees of scaling in N_2-saturated 0.1 M Na_2SO_4 solution containing 10 mM H_2O_2. R_1 and R_2 represent the charge transfer resistance and the hydrogen adsorption resistance, respectively.

- **Updated Methods Section**

In-situ ATR-SEIRAS measurements [Line 801-815, Page 24 (Revised)]

In-situ electrochemical ATR-SEIRAS measurements were performed using a Fourier transform infrared spectrometer (Nicolet iS50, Thermo Fisher Scientific) equipped with a liquid-nitrogen-cooled mercury cadmium telluride (MCT) detector. A Si ATR prism

chemically coated with a thin Au film was used simultaneously as the optical waveguide and the conductive working-electrode substrate, onto which the catalyst ink was uniformly deposited. A standard three-electrode configuration was employed for all tests, comprising the catalyst-modified Au/Si ATR electrode as the working electrode, an Ag/AgCl electrode as the reference electrode, and a Pt wire as the counter electrode. All electrochemical measurements were conducted in a dual-compartment H-cell reactor. The cathodic compartment was filled with O_2-saturated 0.1 M NaCl or 0.1 M $CaCl_2$ as the working electrolyte, while the anodic compartment contained 0.1 M Na_2SO_4. The ATR-SEIRAS spectra were recorded over a potential range from 0.6 to -0.4 V vs. RHE. The spectrum collected at the open-circuit potential was used as the background.

- **New reference**

24. Nie, J. et al. Accelerating water dissociation to achieve ampere-level hydrogen peroxide electrosynthesis in brine and seawater. *Nature Communications* **16**, 5895 (2025).
25. Wang, Y. et al. Differentiating interfacial water structures via alkali metal cation promotor for H_2O_2 electrosynthesis in acid. *Nature Communications* **17**, 4973 (2026).
26. Yu, Y. et al. Regulating active hydrogen supply and intermediate binding for pH-universal H_2O_2 electrosynthesis at ampere-level current density. *Nature Communications* **16**, 10784 (2025).
27. Guo, L. et al. Formate‐Anion‐Induced Water Network Reshaping Enables Concurrent Hydrogen and Magnesium Hydroxide Production From Seawater. *Angewandte Chemie International Edition* **65**, e6937994 (2026).

2. In lines 531-538 of the main text, the authors claim: “The hybrid strategy delivered H_2O_2 electrosynthesis performance in four real water matrices… and maintained 96% of its initial efficacy after 30 h of cyclic testing, highlighting its robust stability. In conclusion, this work… establishing a new paradigm for designing efficient and stable electrocatalytic systems in natural water.” However, almost all experimental evidence supporting the “beneficial scale” mechanism is based on extremely early-stage scaling conditions (only 3 hours of electrolysis at a relatively low current density of 0.1 A). Furthermore, the claimed 30-hour stability is merely an intermittent cyclic test (10 cycles × 3 hours each), not continuous operation. More importantly, the authors themselves observed scale-induced electrode water flooding (Figs. S15 and S44). In actual long-term continuous operation, this flooding and the continuous thickening of the scale layer will inevitably lead to the complete collapse of the three-phase boundary. Therefore, the current experimental results are completely insufficient to support the conclusion that “calcium scale remains beneficial and stable during long-term operation.” Unless a single, continuous long-term test of at least 100 hours is provided, the claim of “efficient and stable in natural water” must be considered a severe overestimation.

Response:

Thank you for this constructive comment regarding the long-term stability claims and the duration of our scaling experiments. We address each concern below and have substantially revised the manuscript to appropriately temper our claims.

- **(1) On the "protective scale" mechanism and the 3-hour experimental duration**
We sincerely apologize if our original statements gave you the mistaken impression that calcium scale is overall beneficial, which was certainly not our intended message. We acknowledge that our experiments (3 h electrolysis at 0.04-0.4 A) only capture the early-stage scaling behavior. Our goal was not to claim that scale formation is overall beneficial, but rather to reveal its dual role in the $2e^-$ ORR: it negatively impacts performance by exacerbating water flooding and inhibiting oxygen transport (**Supplementary Figs. 20 and 51**), while simultaneously playing a partially protective role by suppressing cathodic H_2O_2 reduction. We fully agree with the reviewer that under prolonged continuous operation, progressive scale thickening will inevitably lead to the collapse of the triple-phase boundary, at which point the negative effects of scaling will outweigh the protective benefits. Therefore, calcium scale is indeed overall detrimental in the actual long-term continuous operation. However, the central point we wished to convey is that dissolved Ca^{2+}, not precipitated scale, is the dominant factor responsible for suppressing H_2O_2 electrosynthesis performance. Even at early stages, the detrimental effect of dissolved Ca^{2+} on $2e^-$ ORR selectivity is already severe enough to cause system failure (FE dropping from ~52.4% to ~12.0% at 0.1A, Fig. 1a), well before catastrophic scale accumulation occurs. This suggests that managing dissolved Ca^{2+} should take precedence over managing scale deposition in practical system design. We have revised the Discussion section to clearly articulate this nuanced position, and have explicitly acknowledged the limitations of our current experimental timescale.

- **(2) On the 30-hour stability claim**
  We agree that intermittent cyclic testing does not equate to continuous long-term operation and the term "30 h of cyclic testing" could be misleading if not properly contextualized. Therefore, we have revised this throughout the manuscript to explicitly state "intermittent cyclic stability test (10 cycles × 3 h each, 30 h cumulative operation)" and have replaced "robust stability" with "promising reusability under repeated batch operation" to accurately reflect the nature of the experiment. These intermittent cycling experiments are designed to demonstrate the stability of tolerance toward dissolved Ca^{2+}, rather than to address the long-term mitigation of scale-induced flooding.
  We have revised the statement from "maintained 96% of its initial efficacy after 30 h of cyclic testing, highlighting its robust stability" to "retained 96% of its initial performance over 10 intermittent operation cycles (30 h cumulative), demonstrating promising reusability toward dissolved Ca^{2+}."
- **(3) On the claim of "efficient and stable in natural water"**
  We would like to acknowledge an inherent limitation of the current reactor configuration. The natural air-diffusion electrode employed in this study is intrinsically susceptible to long-term water flooding, which represents a well-recognized challenge in the field. Even in the absence of Ca^{2+}, this configuration is susceptible to noticeable water flooding during extended operation. Therefore, limited by the current reactor design, we are unable to conduct a continuous long-term test of 100 h at present. Additionally, it is important to clarify that our intermittent cycling tests are designed to demonstrate the stability of dissolved Ca^{2+} resistance, rather than to achieve effective mitigation of flooding. We acknowledge this limitation and will address it in future work through optimization of the electrode architecture and hydrophobic durability.
  Therefore, we agree that the original claim was a significant overstatement unsupported by the current experimental evidence. The concluding statement has been tempered from "establishing a new paradigm for designing efficient and stable electrocatalytic systems" to " establishing a new paradigm for designing Ca^{2+}-tolerant cathodes".

The Revised Content in Manuscript

Based on your comments, we acknowledge our limitation and have substantially revised the manuscript to more clearly elucidate the central focus of our study and appropriately temper our claims.

- **New discussion in Section "Dual effect of precipitated calcium scale on $2e^-$ ORR." [Line 249-255, Page 7 (Revised)]**
  It is worth noting that the accumulation of calcium scale over longer time can further exacerbate electrode flooding, potentially leading to negative effects that far outweigh any protective role. Nevertheless, we emphasize that even at early stages, the detrimental effect of dissolved Ca^{2+} on $2e^-$ ORR selectivity is already severe enough to cause system failure. This suggests that managing dissolved Ca^{2+} should take precedence over controlling calcium scale deposition in the design of electrochemical systems.

- **Revised statement in Section "Discussion" [Line 625-632, Page 20 (Revised)]**
  The hybrid strategy delivered H_2O_2 electrosynthesis performance in four real water matrices comparable to that in deionized water, and retained 96% of its initial performance over 10 intermittent operation cycles (30 h cumulative), demonstrating promising reusability toward dissolved Ca^{2+}. In conclusion, this work pioneers two strategies including surfactant-induced interfacial field control and 3D fenced-cathode confinement that enables simultaneous Ca^{2+} repulsion, proton-availability suppression, and alkaline microenvironment localization, establishing a new paradigm for designing Ca^{2+}-tolerant cathodes, with potential promise for extension to natural water matrices.

3. The authors explicitly point out in the text (Lines 173-179) and SI (Figs. S14, S15, S42, S44) that the formation of calcium scale transitions the electrode surface from hydrophobic to hydrophilic, triggering severe water flooding and inhibiting oxygen transport. Additionally, the ECSA and surface roughness change significantly with the degree of scaling. In Gas Diffusion Electrodes (GDEs), the kinetics and selectivity of the 2e$^-$ ORR are extremely sensitive to the gas-liquid distribution at the three-phase boundary and the local oxygen concentration. Since scaling has already triggered such severe mass transport limitations and interfacial physical changes, the attenuation or fluctuation in electrode performance is entirely dominated by these physical factors. However, when discussing the core mechanism, the authors repeatedly attribute this phenomenon to dissolved Ca^{2+} promoting interfacial water dissociation and shifting the reaction pathway toward the 4e$^-$ ORR. The current experimental design fails to decouple the 'physical effects caused by deteriorated mass transport/wettability' from the 'chemical catalytic effects of the scale barrier.' Without ruling out the severe disruption of the three-phase boundary as a dominant confounding factor, this mechanistic hypothesis remains unconvincing."

Response:

Thank you for this insightful suggestion regarding the decoupling of physical and chemical effects in our mechanistic interpretation. We greatly appreciate the fundamental concern whether the observed performance attenuation is dominated by physical mass transport limitations (three-phase boundary disruption results from water flooding) rather than the chemical catalytic effect of dissolved Ca^{2+} (4e$^-$ ORR pathway shift results from enhanced water dissociation), as it has prompted us to strengthen our argument substantially.

We fully acknowledge that in a GDE system, scaling-induced wettability changes and water flooding can severely impair oxygen transport, thereby reducing apparent H_2O_2 electroproduction independently of any chemical catalytic effect. To address this, we designed a decoupling experiment that were not sufficiently emphasized in the original manuscript. Specifically:

- **Scaled cathodes tested in Ca^{2+}-free electrolyte:**

  As shown in **Fig. 1a** in the manuscript, we prepared scaled C0 cathodes at different currents (0.1A-scaled and 0.4A-scaled), then transferred them to Ca^{2+}-free electrolyte for performance evaluation. These scaled cathodes retain the same physical effects of scaling (increased wettability, ECSA and roughness changes), yet operate in the complete absence of dissolved Ca^{2+}. The key result is that the 0.1A-scaled cathode achieved an FE of 46.40% in Ca^{2+}-free electrolyte, whereas the fresh C0 cathode achieved only 12.00% FE in the presence of 200 mg/L Ca^{2+}. If physical effects were the dominant factor, the scaled cathode should have performed worse, not better. The scaled C18 cathodes tested in Ca^{2+}-free electrolyte also exhibited a similar trend, further supporting the above decoupling analysis. This directly demonstrates that dissolved Ca^{2+} exerts a chemical catalytic effect that dominates the performance loss, exceeding the physical factors.

To better delineate the respective contributions of physical and chemical effects to the H_2O_2 electrosynthesis performance, we have provided an additional schematic diagram

in the Supplementary Information, roughly partitioning the contributions of these two factors.

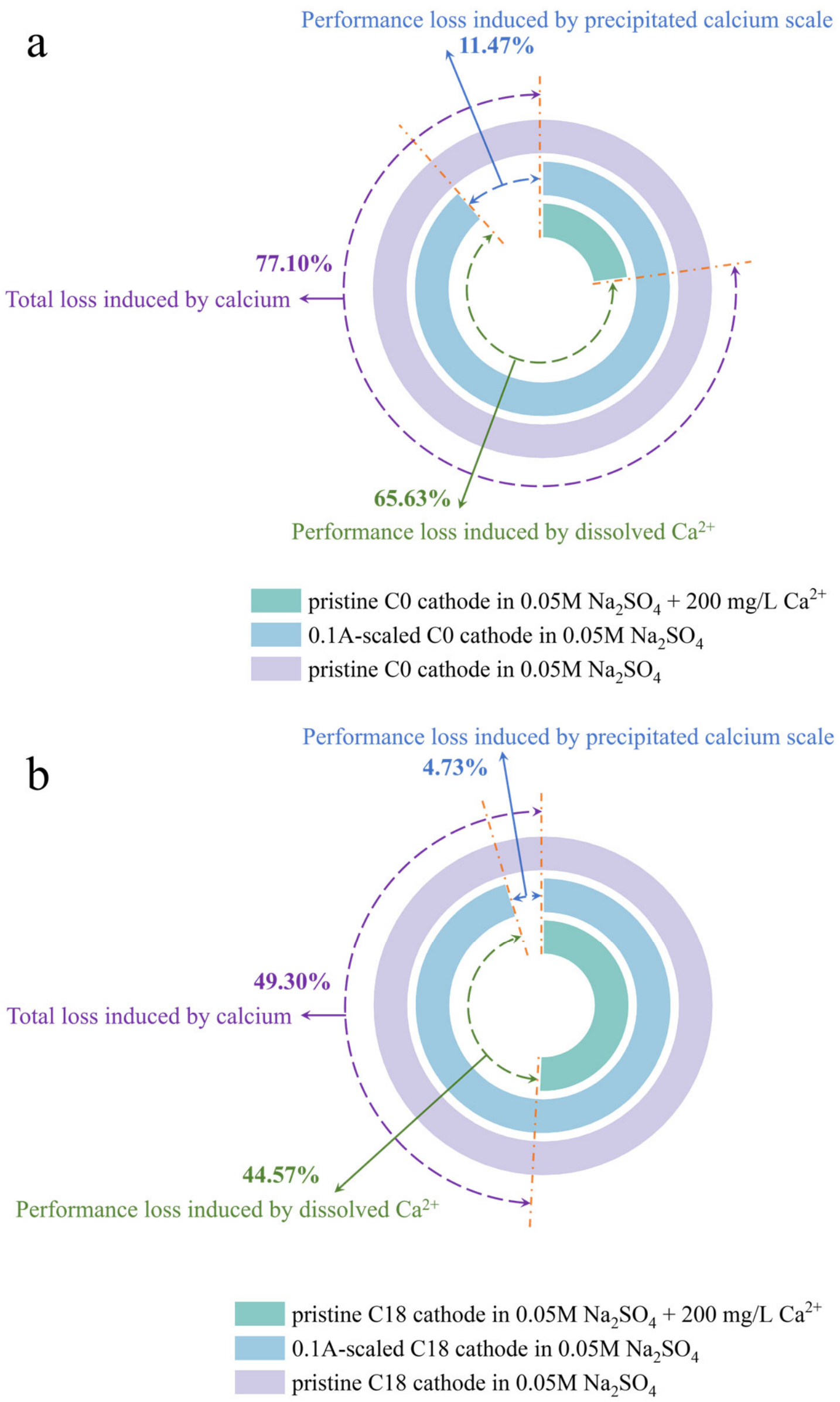

Figure Decoupling analysis of the inhibitory effects of dissolved Ca^{2+} and precipitated calcium scale on H_2O_2 electrosynthesis performance for (a) C0 and (b) C18.

- **For the C0 cathode at 0.1A:** The overall performance decay attributed to calcium species was 77.10%. Notably, dissolved Ca^{2+} dominated this loss with an 85.12% contribution, whereas deposited calcium scale played a minor role, accounting for only 14.88% of the performance decrease.
- **For the C18 cathode at 0.1A:** The presence of calcium species resulted in a total performance degradation of 49.30%, of which dissolved Ca^{2+} was responsible for a 90.41% decline and deposited calcium scale for an 9.59% decline.

We wish to clarify that our mechanistic argument is not that physical effects are absent, but that dissolved Ca^{2+} exerts the dominant chemical effect, especially in the early-stage

scaling regime where catastrophic three-phase boundary collapse has not yet occurred. We have acknowledged that in the GDE configuration, physical and chemical effects are coupled, and that the decoupling experiments demonstrate the primacy of the chemical effect under our tested conditions, with physical oxygen transport limitations playing a secondary but non-negligible role.

The Revised Content in Manuscript

Based on your comments, we have performed decoupling analysis and clearly demonstrated that dissolved Ca^{2+} plays the dominant role in suppressing the $2e^-$ ORR, while precipitated calcium scale exerts a non-negligible secondary effect. The following specific revisions have been implemented in the manuscript and supporting information:

- **Updated discussion in Section "Evidence for the suppression of $2e^-$ ORR by dissolved Ca^{2+}." [Line 114-134, Page 4 (Revised)]**

  It is important to distinguish the physical effects of scaling from the chemical catalytic effect of dissolved Ca^{2+} on the observed performance degradation. To decouple these contributions, the H_2O_2 electrosynthesis performance of scaled cathodes was directly evaluated in Ca^{2+}-free systems, thereby preserving the physical consequences of scaling while eliminating dissolved Ca^{2+}. The 0.1A-scaled C0 cathode achieved 46.40% FE in Ca^{2+}-free electrolyte, whereas the fresh C0 cathode reached only 12.00% FE in the presence of 200 mg/L Ca^{2+} (**Fig. 1a and Supplementary Fig. 6**). To further quantify these contributions, we roughly partitioned the overall performance loss into dissolved Ca^{2+}-induced and scale-induced components (**Supplementary Fig. 7**). For the C0 cathode at 0.1 A, the total performance decay attributed to calcium species was 77.10%, of which dissolved Ca^{2+} contributed 85.12% and deposited scale accounted for only 14.88%. Linear sweep voltammetry (LSV) curves of cathodes with different scaling degrees further supported these observations (**Supplementary Fig. 8**). Despite contaminated with calcium scale, the scaled cathodes retained most of their ORR activity. Control experiments with NaCl addition were performed to isolate the effect of Cl^- co-introduced during Ca^{2+} addition, confirming that chloride species contributed negligibly to the observed variations in cathodic H_2O_2 production (**Supplementary Fig. 9**). These collective results directly demonstrate that dissolved Ca^{2+} exerts the dominant cationic effect on suppressing the $2e^-$ ORR performance, while precipitated calcium scale plays a secondary but non-negligible role under the tested conditions.

- **New discussion in Section "$2e^-$ ORR performance and scale formation of surfactant-modified cathodes in Ca^{2+}-containing electrolytes." [Line 384-388, Page 12 (Revised)]**

  It is noteworthy that the mechanistic role of dissolved Ca^{2+} versus precipitated calcium scale in modulating the $2e^-$ ORR selectivity also applies to the C18 cathode. This observation further supports the main contribution of dissolved Ca^{2+} and the secondary contribution of deposited calcium scale to the suppression of H_2O_2 electroproduction (**Fig. 3g** and **Supplementary Figs. 43-45**).

- **New Figures in Supplementary Information:**

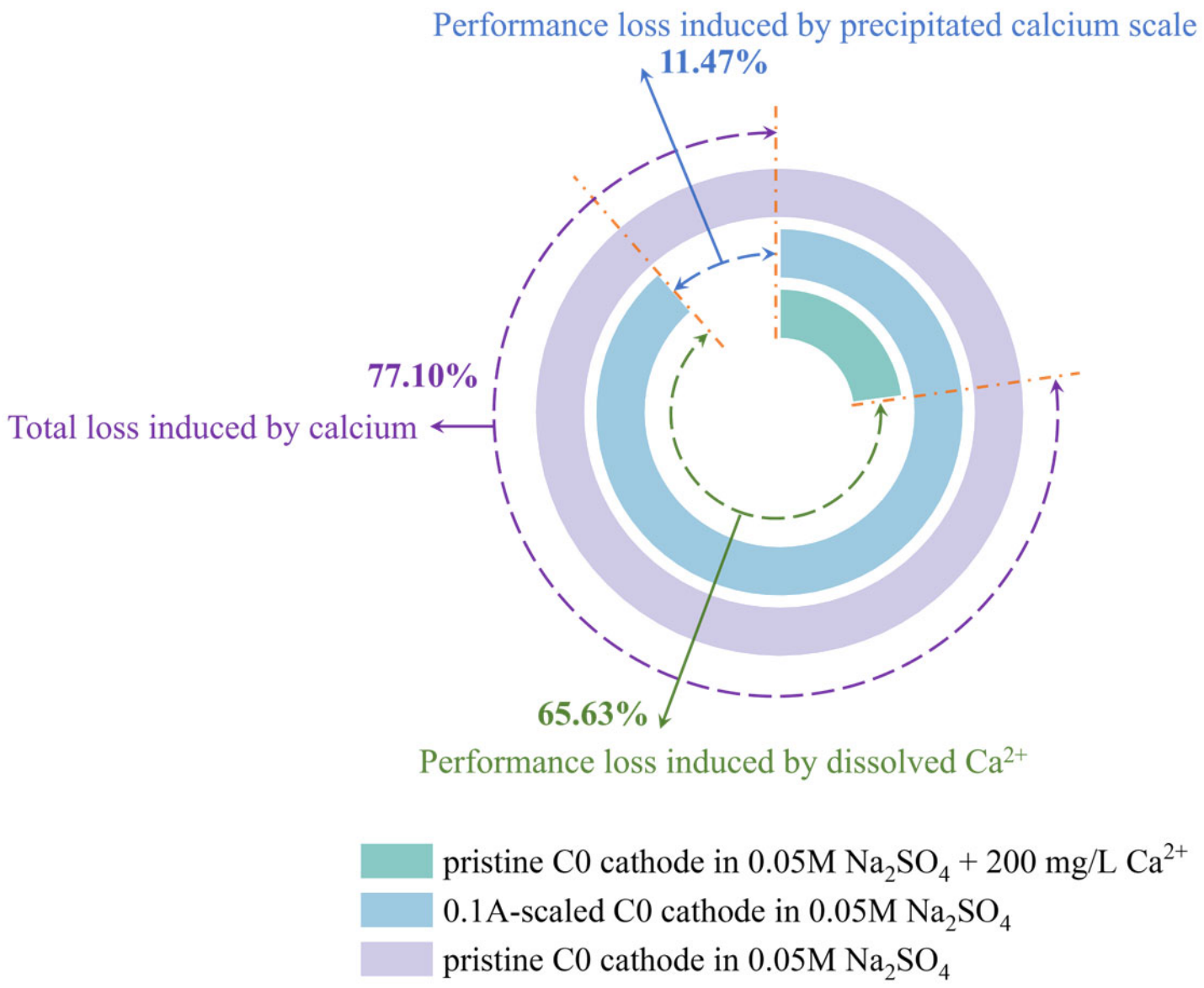

Supplementary Fig. 7. Decoupling analysis of the inhibitory effects of dissolved Ca^{2+} and precipitated calcium scale on H_2O_2 electrosynthesis performance for C0.

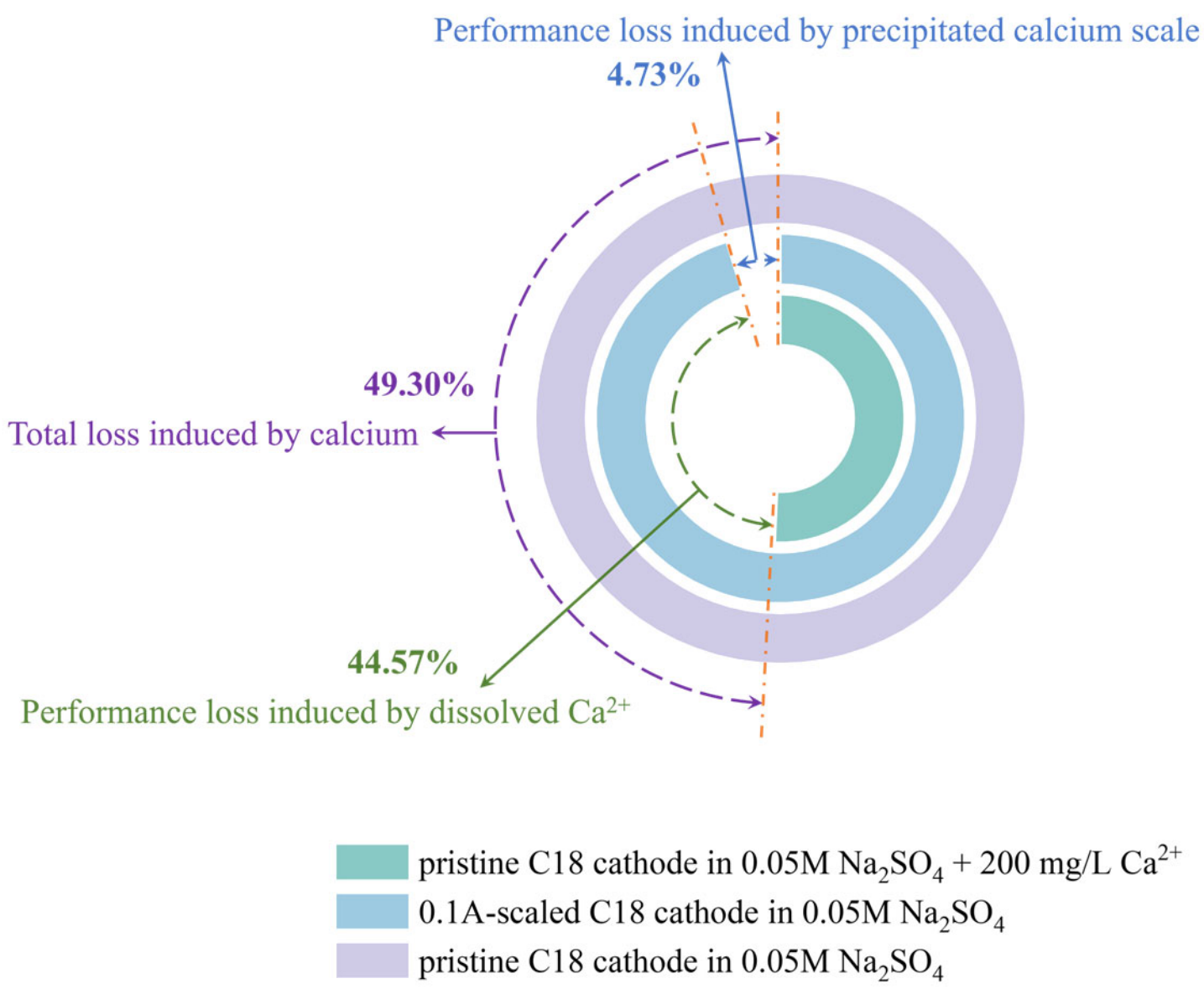

Supplementary Fig. 44. Decoupling analysis of the inhibitory effects of dissolved Ca^{2+} and precipitated calcium scale on H_2O_2 electrosynthesis performance for C18.

4. The manuscript attempts to demonstrate that dissolved Ca^{2+} promotes the further consumption of H_2O_2, while calcium scale inhibits this parasitic reaction, by relying on H_2O_2 decomposition curves, reduction polarization, and impedance analysis. However, these experiments represent oversimplified conditions that are not equivalent to the real ORR environment where H_2O_2 is generated dynamically under continuous oxygen supply and local alkalinization.

Response:

Thank you for this insightful comment. We agree that our H_2O_2 decomposition experiments, reduction polarization curves, and EIS analyses were conducted under ex-situ conditions that do not fully replicate the dynamic ORR environment where H_2O_2 is generated continuously under oxygen supply and local alkalinization. We fully acknowledge this concern and have conducted additional in-situ RRDE measurements to address this gap.

The specific experimental setup and testing parameters are provided in the Methods section below. The analysis of the RRDE results is presented as follows:

- **(1) RRDE measurements in O_2-saturated electrolyte replicate the real ORR environment**

  To address the concern, we have performed rotating ring-disk electrode (RRDE) measurements in O_2-saturated electrolyte, which provides a real ORR environment with continuous O_2 supply, directly replicating the conditions under which H_2O_2 is generated and potentially further reduced. The RRDE results confirmed that dissolved Ca^{2+} shifts the ORR pathway from the $2e^-$ toward the $4e^-$ direction, as evidenced by the increased electron transfer number (n) and decreased H_2O_2 selectivity across the entire potential range compared to the Na^+ system.

- **(2) Deconvolution of ORR current directly identifies the specific step affected by Ca^{2+}**

  To further elucidate the kinetic origin of the Ca^{2+}-induced selectivity loss, we deconvoluted the total ORR current into two sequential steps following the established methodology in the literature[23]. In this framework, the total ORR current density (j_D) is deconvoluted into:

  Oxygen-to-peroxide (O2P): The $2e^-$ reduction of O_2 to H_2O_2. The kinetic current density for this step (j_{O2P}) represents the intrinsic activity for the $2e^-$ ORR pathway.

  Peroxide-to-hydroxide (P2H): The further $2e^-$ reduction of the in-situ generated H_2O_2 to H_2O. The kinetic current density for this step (j_{P2H}) represents the parasitic H_2O_2 consumption pathway.

  (a) j_{O2P} (O_2 to H_2O_2): No significant difference between Na^+ and Ca^{2+} systems.

  The deconvolution results reveal that the kinetic current density for O2P (j_{O2P}) is nearly identical in the Na^+ and Ca^{2+} systems, indicating that dissolved Ca^{2+} does not directly inhibit the $2e^-$ reduction of O_2 to H_2O_2. The intrinsic activity of the C0 catalyst for H_2O_2 generation is preserved even in the presence of Ca^{2+}.

  (b) j_{P2H} (H_2O_2 to H_2O): Dramatically enhanced by Ca^{2+}.

  In contrast, the kinetic current density for the further reduction of H_2O_2 to H_2O (j_{P2H}) is substantially enhanced in the Ca^{2+} system. This provides direct in-situ evidence that

dissolved Ca^{2+} accelerates the further electroreduction of dynamically generated H_2O_2 to H_2O, which is the primary cause of the reduced net $2e^-$ ORR selectivity.

- **(3) Consistency with the central thesis of this work**

  The RRDE deconvolution results are in complete agreement with the core mechanistic conclusion of our study: dissolved Ca^{2+} suppresses net H_2O_2 electrosynthesis primarily by accelerating the further reduction of H_2O_2 to H_2O (P2H), rather than by inhibiting the $2e^-$ O_2-to-H_2O_2 step (O2P) itself. The ex-situ experiments, including H_2O_2 decomposition curves, reduction polarization and impedance analysis, while conducted under simplified conditions, are now validated by the in-situ RRDE results and serve to provide kinetic and mechanistic details that complement the pathway-level insights from RRDE.

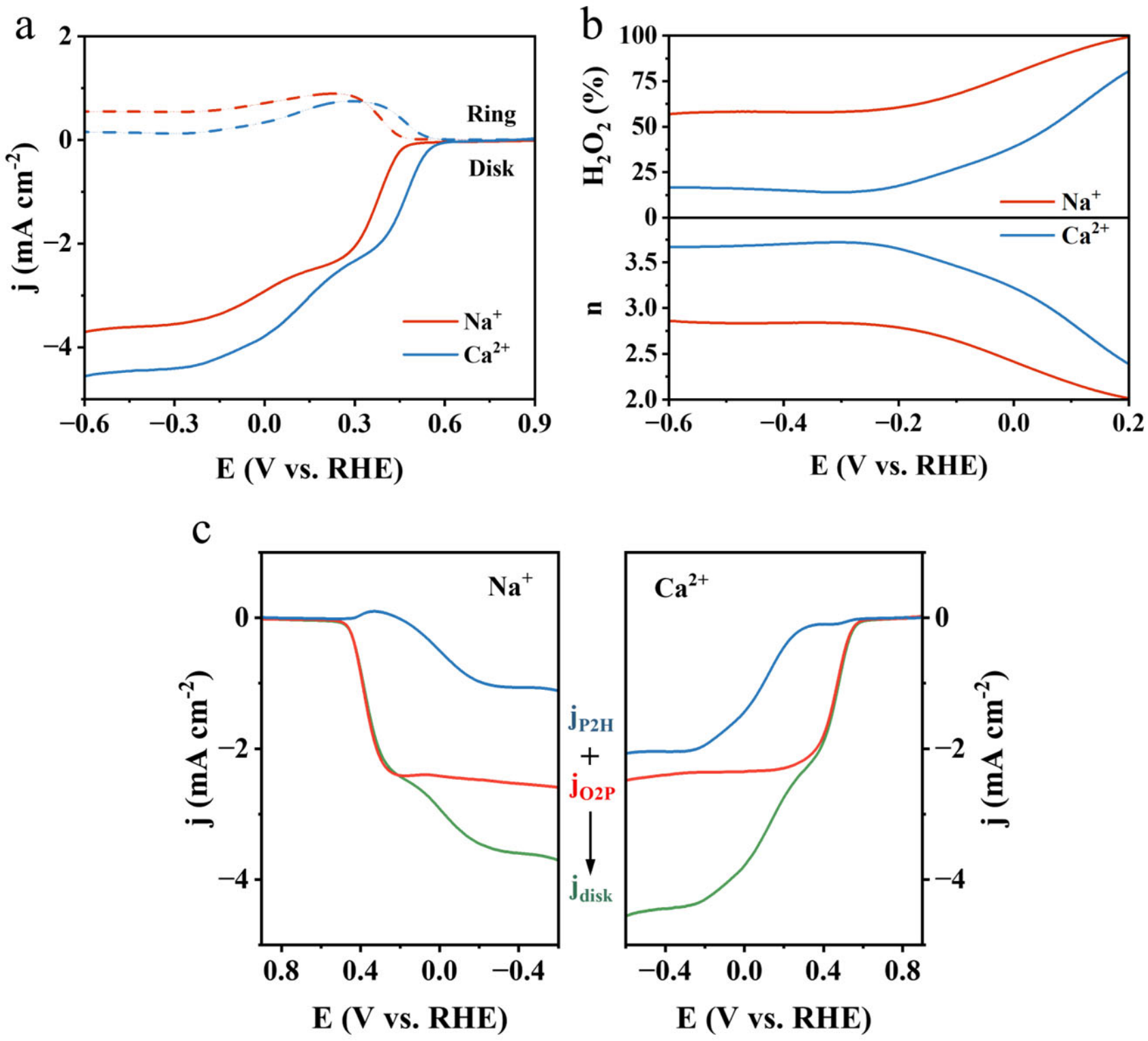

Figure RRDE measurements for C0. (a) LSV curves of C0 tested at 1600 rpm in O_2-saturated 0.1M NaCl or 0.1M $CaCl_2$. (b) The H_2O_2 selectivity and electron transfer number of C0 in different systems. (c) Current deconvolution analysis of C0 in Na^+ and Ca^{2+} systems.

The Revised Content in Manuscript

Based on your comments, we have revised the manuscript to present this integrated evidence, with the in-situ RRDE deconvolution as the cornerstone linking the dynamic ORR environment to the ex-situ kinetic analyses and theoretical calculations. The following specific revisions have been implemented in the manuscript and supporting information:

- **New Figures and Discussion in Section "Evidence for the suppression of $2e^-$ ORR by dissolved Ca^{2+}." [Line 135-149, Page 5 (Revised)]**

  To provide direct in-situ evidence for the Ca^{2+}-induced ORR pathway shift, rotating ring-disk electrode (RRDE) measurements were performed in O_2-saturated electrolyte

(Fig. 1b). In the presence of Ca^{2+}, the electron transfer number (n) increased and the H_2O_2 selectivity correspondingly decreased across the entire potential range compared to the Na^+ system **(Fig. 1c)**, confirming that dissolved Ca^{2+} dynamically shifts the ORR pathway toward the $4e^-$ direction. To further identify the specific kinetic step affected, the total ORR current (j_D) was deconvoluted into two sequential reactions: oxygen-to-peroxide (O2P) and peroxide-to-hydroxide (P2H) **(Fig. 1d)**[23]. The kinetic current for O2P (j_{O2P}) showed negligible difference between the Na^+ and Ca^{2+} systems, indicating that dissolved Ca^{2+} does not directly inhibit the $2e^-$ reduction of O_2 to H_2O_2. In contrast, the kinetic current for P2H (j_{P2H}) was substantially enhanced in the Ca^{2+}-containing electrolyte, demonstrating that Ca^{2+} accelerates the parasitic electroreduction of in-situ generated H_2O_2 to H_2O. These results provide direct in-situ kinetic evidence that dissolved Ca^{2+} suppresses net H_2O_2 electrosynthesis primarily by promoting the further reduction of H_2O_2 rather than by inhibiting the O_2-to-H_2O_2 step itself.

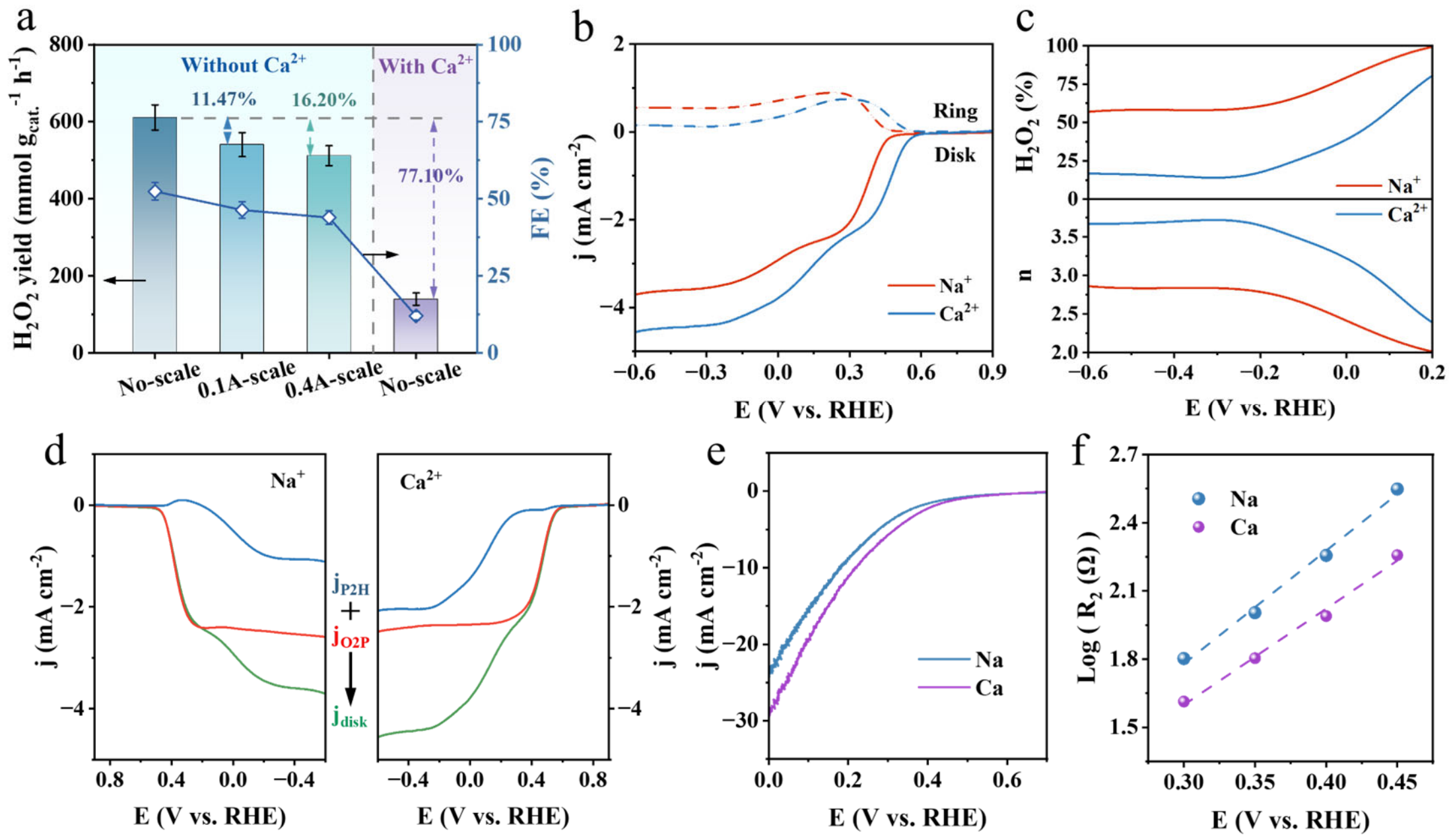

Fig. 1. Effects of dissolved Ca^{2+} on $2e^-$ ORR for oxygen-doped carbon black (named C0). (a) H_2O_2 electrosynthesis performance of C0 cathodes with varying degrees of scaling in 0.05M Na_2SO_4 and no-scale cathode in 0.05M Na_2SO_4 and 200 mg/L Ca^{2+}. (b) LSV curves of C0 from RRDE tested at 1600 rpm in O_2-saturated 0.1M NaCl or 0.1M $CaCl_2$. (c) The electron transfer number and H_2O_2 selectivity of C0 in different systems. (d) Deconvolution analysis of disk currents in Na^+ and Ca^{2+} systems. (e) LSV curves and (f) EIS-derived Tafel plots obtained from the hydrogen adsorption resistance R_2 of C0 in N_2-saturated 0.1M NaCl or $CaCl_2$ electrolytes containing 10mM H_2O_2.

- **New Figures in Supplementary Information:**
  Fig. 1a in the original manuscript has been transferred to the Supplementary Information and is now renumbered as **Supplementary Fig. 1**.

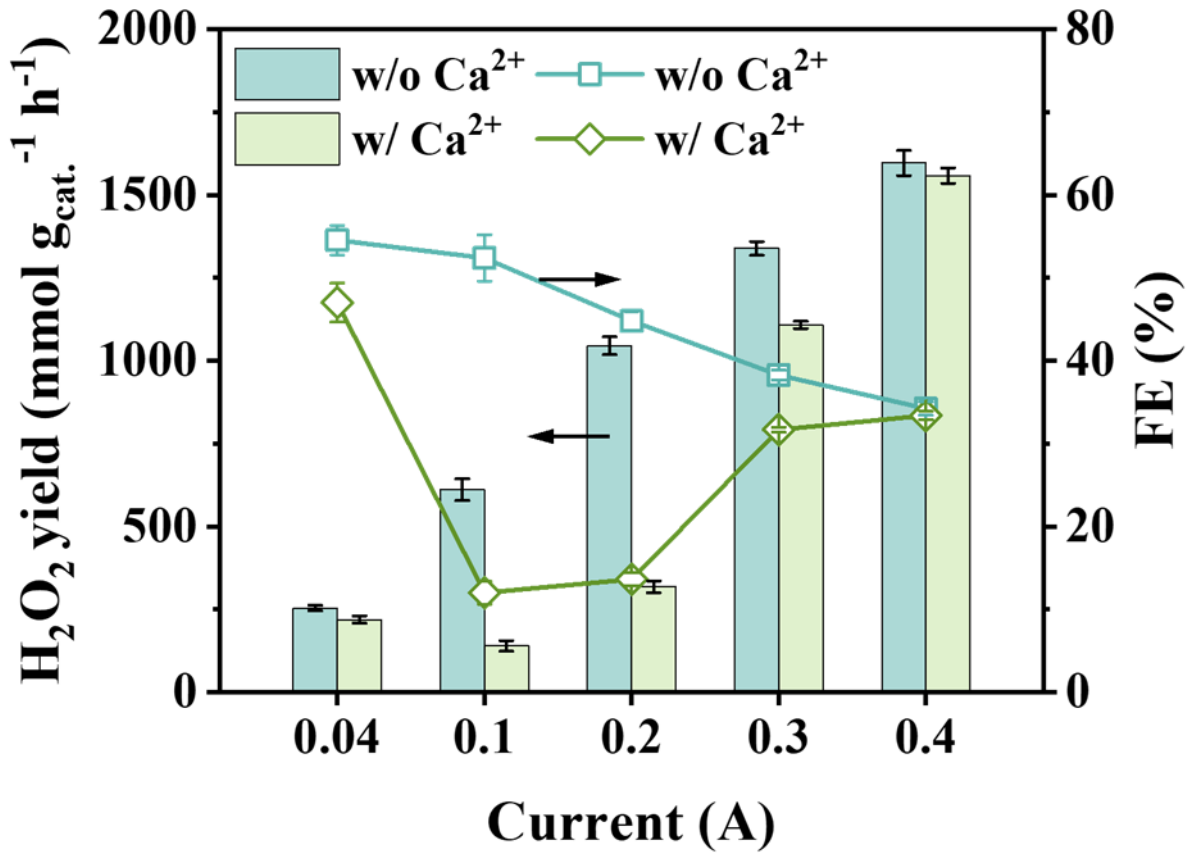

Supplementary Fig. 1. Effects of current densities on H_2O_2 electrosynthesis performance for C0 in the absence (w/o) and presence (w/) of 200 mg/L Ca^{2+}. Reaction conditions: [Na_2SO_4] = 0.05 M, a rotation speed of 600 rpm, an initial pH of 7 and electrolysis of 3 h.

Supplementary Fig. 3 in the original Supplementary Information has been replaced and is now renumbered as **Supplementary Fig. 5**.

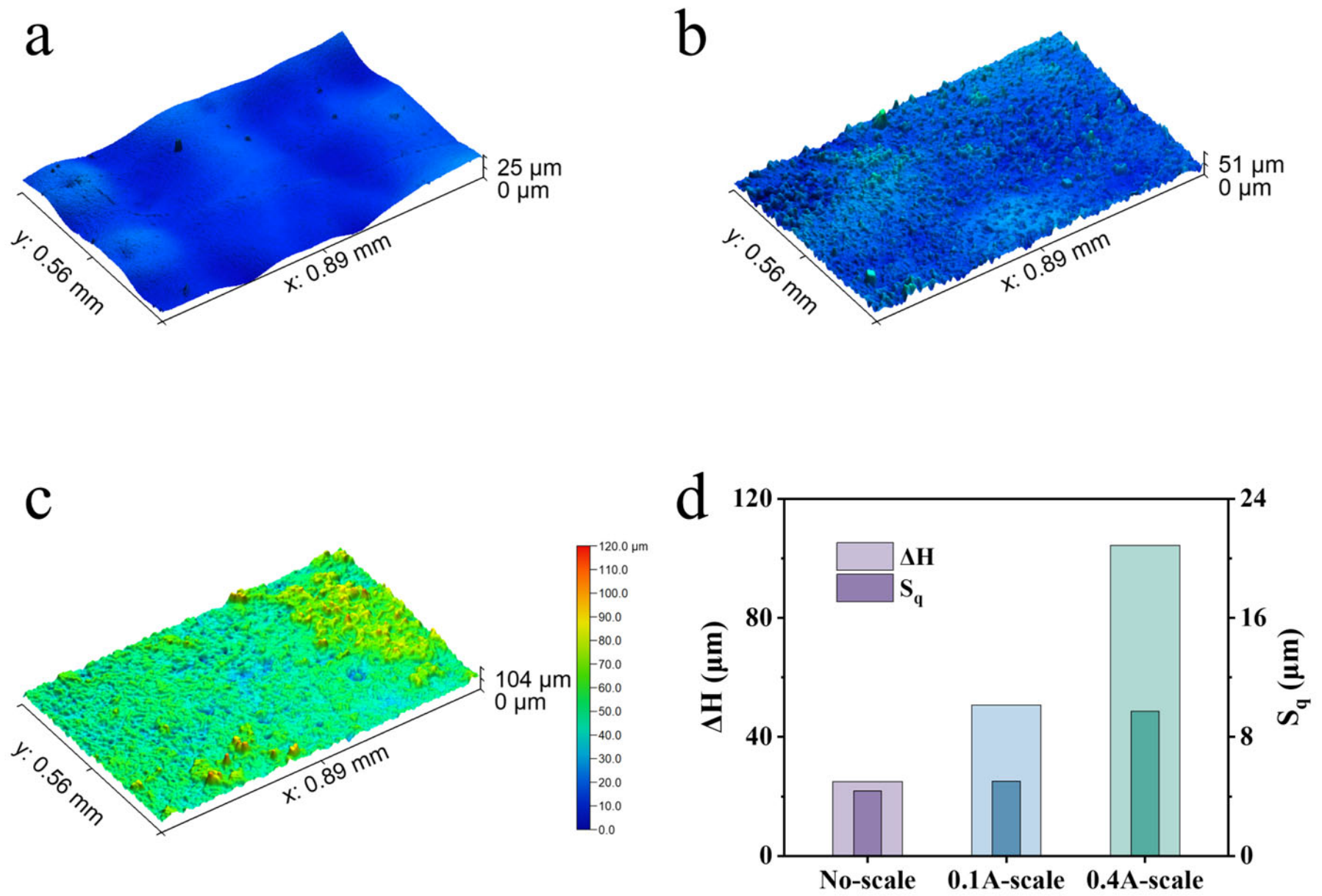

Supplementary Fig. 5. 3D surface profilometry of C0 (a) before experiments and after experiments at (b) 0.1A and (c) 0.4A in the presence of 200 mg/L Ca^{2+}. Variations in the height range (ΔH) and RMS roughness (S_q) of C0 cathodes with varying degrees of scaling. Reaction conditions: [Na_2SO_4] = 0.05 M, $[Ca^{2+}]_0$ = 200 mg/L, a rotation speed of 600 rpm, an initial pH of 7 and electrolysis of 3 h.

- **Updated Methods Section**

RRDE measurement and current density deconvolution for ORR [Line 773-800, Page 24 (Revised)]

The selectivity of $2e^-$ ORR was evaluated using a rotating ring-disk electrode (RRDE) setup (AFMSRCE, Pine, Physicochemical Co. Ltd.), consisting of a glassy carbon disk

electrode (diameter: 5.61mm, area: 0.2475 cm^2) and a platinum ring electrode (inner diameter: 6.25mm, outer diameter: 7.92mm, area: 0.1866 cm^2).10 µL of the catalyst ink was drop-cast onto the disk electrode and allowed to dry naturally, serving as the working electrode. An Ag/AgCl electrode and a platinum wire were used as the reference and counter electrodes, respectively. Prior to electrochemical measurements, the catalyst was electrochemically activated by performing cyclic voltammetry (CV) scans at 50 mV/s until a stable voltammogram was obtained. Linear sweep voltammetry (LSV) was conducted in O_2-saturated 0.1 M NaCl or 0.1 M $CaCl_2$ solutions at a scan rate of 10 mV/s, with the RRDE rotating at 1600 rpm. The platinum ring electrode was held at 1.2 V vs. RHE to detect the generated H_2O_2. The H_2O_2 selectivity and the electron transfer number (n) were calculated using the following equations:

$$H_2O_2\ selectivity\ (\%) = \frac{I_R/N}{I_D + I_R/N} \times 200$$

$$n = \frac{I_D}{I_D + I_R/N} \times 4$$

where I_D and I_R represent the disk current and ring current, respectively, and N is the collection efficiency of the ring electrode (N = 0.37).

Referring to a previously reported method, the ring-disk current densities can be deconvoluted into two sequential reaction current densities: those corresponding to the reduction of oxygen to peroxide (O2P), and those corresponding to the subsequent reduction of peroxide to hydroxide (P2H)[23]. The method describes a stepwise oxygen reduction reaction (ORR) pathway, which is consistent with the current reaction occurring on the carbon electrode. The current densities for the O2P and P2H steps were then calculated using the following formulas:

$$j_{O2P} = j_D \times (\frac{\sigma N + 1}{2\sigma N})$$

$$j_{P2H} = j_D \times (\frac{\sigma N - 1}{2\sigma N})$$

where j_D is the disk current density, σ is the disk-to-ring current density ratio.

- **New reference**

23. Yang, Q. et al. Selective O_2-to-H_2O_2 Electrosynthesis by a High-Performance, Single-Pass Electrofiltration System Using Ibuprofen-Laden CNT Membranes. *Environmental Science & Technology* **58**, 19058-19069 (2024).

5. The authors employ theoretical calculations to demonstrate the core hypothesis that "dissolved Ca^{2+} accelerates interfacial water dissociation." However, the constructed theoretical model is unreasonable. First, the authors selected standard single-layer graphene as the model, completely ignoring the crucial influence of the abundant oxygen-containing functional groups and defect sites present on the surface of the actual catalyst used (oxygen-doped carbon black, C0). Second, as a core step in the electrochemical reaction, the calculation of the water dissociation energy barrier ignores the critical effects of the applied potential and the interfacial electric field. Consequently, the obtained energy barrier data lack credibility.

Response:

Thank you for this constructive comment regarding the DFT calculations. We acknowledge that a more realistic computational model, incorporating applied potential and interfacial electric field, would significantly strengthen the credibility of our calculated data for water dissociation energy barriers. We fully accept these criticisms and have comprehensively reconstructed our DFT calculations to address both concerns.

- **(1) Reconstruction of the DFT model based on experimental characterization and literature guidance**

We fully agree that the initial model is inadequate for C0. Our experimental characterization (XPS, FTIR and Raman) demonstrates that C0 possesses both oxygen functional groups and intrinsic carbon defects. Furthermore, recent literature on carbon-based $2e^-$ ORR catalysts has identified configurations combining ether groups (C-O-C) with adjacent carbon vacancies as particularly favorable active sites for H_2O_2 electrosynthesis, owing to their optimal *OOH binding energy that favors H_2O_2 desorption over O-O bond cleavage[28].

In response to your comment, we have reconstructed our DFT model to incorporate both a C-O-C ether group and an adjacent carbon vacancy defect within the graphene monolayer. The model directly reflects the experimental surface chemistry of C0 and represents the type of active site configuration associated with high $2e^-$ ORR selectivity in the literature. The revised model and corresponding calculations are presented in the updated manuscript and Supplementary Information (**Figs. 1j, 1k** and **Supplementary Figs. 16-18**).

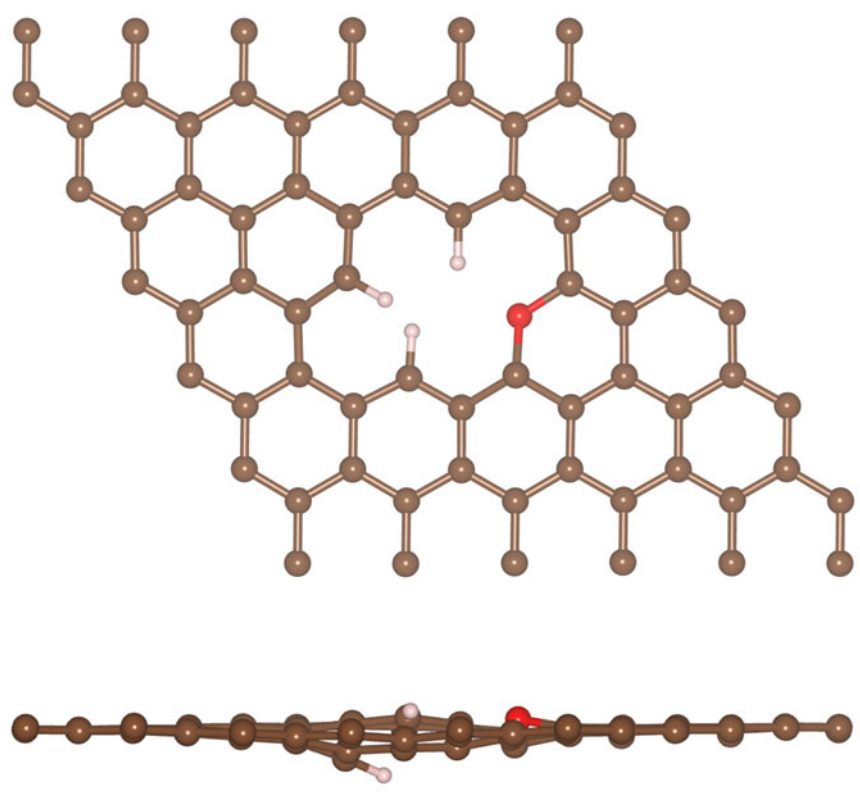

Figure Atomic configurations of the reconstructed model incorporating both a C-O-C ether group and an adjacent carbon vacancy defect within the graphene monolayer. The brown, red, and pink spheres represent C, O, and H atoms, respectively.

- **(2) Incorporation of applied potential and interfacial electric field effects**
  To address the second concern, we have also incorporated the effects of applied potential and interfacial electric field into our calculations (details presented in the revised Methods). These results, combined with the reconstructed structural model, provide a substantially more rigorous theoretical foundation for our mechanistic hypothesis.
- **(3) Key results from the reconstructed model**
  Despite the significant change in the active site configuration (from pristine graphene to C-O-C + adjacent vacancy defect), the core mechanistic conclusions remain qualitatively consistent:
  (a) Ca^{2+} continues to lower the activation barrier for interfacial water dissociation on the reconstructed C0 model.
  (b) The STAB-modified surface (C18) continues to exhibit weaker Ca^{2+} binding compared to C0.

The Revised Content in Manuscript

Based on your comments, we have reconstructed the computational model and incorporated the effects of applied potential and interfacial electric field on the water dissociation energy barrier. The following specific revisions have been implemented in the manuscript and supporting information:

- **Replaced Figures and Analysis in Section "Evidence for the suppression of 2e$^-$ ORR by dissolved Ca^{2+}." [Line195-211, Page 6 (Revised)]**
  The original **Fig. 1g** has been replaced with the new energy profiles of water dissociation process (**new Fig. 1k**) based on the reconstructed models. The corresponding analysis in the text has also been revised as follows:
  DFT calculations were further employed to elucidate the critical role of Ca^{2+} in promoting water dissociation. A monolayer graphene structure incorporating an ether group and an adjacent carbon vacancy defect was constructed as a representative model for DFT calculations (**Fig. 1j, Supplementary Figs. 16** and **17**), considering the experimental surface chemistry of C0 and the active site configuration associated with high 2e$^-$ ORR selectivity in the literature[28]. The calculation results demonstrate that Ca^{2+} significantly lowers the activation energy barrier for water dissociation ($H_2O \rightarrow H^+ + OH^-$), markedly enhancing the dissociation process (**Fig. 1k**). To further elucidate the origin of Ca^{2+}-accelerated interfacial water dissociation, the role of interfacial electric field was examined. Charge transfer between the slab and electrolyte was quantified using Bader charge analysis, and the interfacial electric field strength was estimated based on a parallel-plate capacitor model. Under applied electric field, the H-OH bond of interfacial H_2O is polarized through *H-OH$^{\delta-}$-M$^+$ interactions, as established in previous fundamental studies[16]. The Ca^{2+} system exhibits a substantially enhanced electric field strength, accompanied by elongation of the H-OH bond, indicating that Ca^{2+} facilitates the water dissociation step by promoting interfacial H_2O polarization (**Supplementary Fig. 18**).

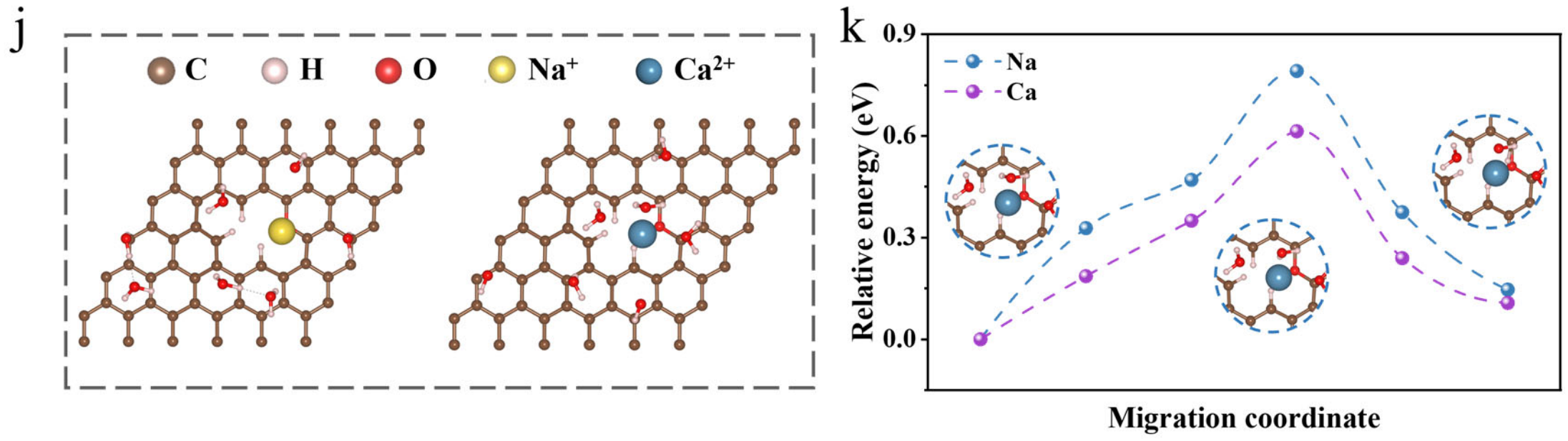

Fig. 1. Effects of dissolved Ca^{2+} on $2e^{-}$ ORR for oxygen-doped carbon black (named C0). (j) Theoretical models of the oxygen-doped graphene surface with Na^{+} and Ca^{2+}. (k) Transition states and energy profiles of water dissociation process.

- **Replaced Figures and Analysis in Section "Mechanistic study for enhanced Ca^{2+}-tolerance of surfactant-modified cathodes." [Line 467-472, Page 15 (Revised)]**

The original **Fig. 4e and 4f** have been replaced with the new adsorption energy profiles (new **Fig. 4e and 4f**) based on the reconstructed models. The corresponding analysis in the text has also been revised as follows:

To elucidate the mechanism underlying the high Ca^{2+} tolerance of the C18 cathode, DFT calculations were employed to investigate the thermodynamic properties of the C0 and C18. The pristine graphene model and the one modified with STAB were used to represent C0 and C18, respectively (**Fig. 4e, 4f and Supplementary Fig. 54**). The adsorption energies (E_{ad}) of Ca^{2+} on C0 and C18 were -1.34 eV and -0.85 eV, respectively, demonstrating a weaker interaction between Ca^{2+} and C18.

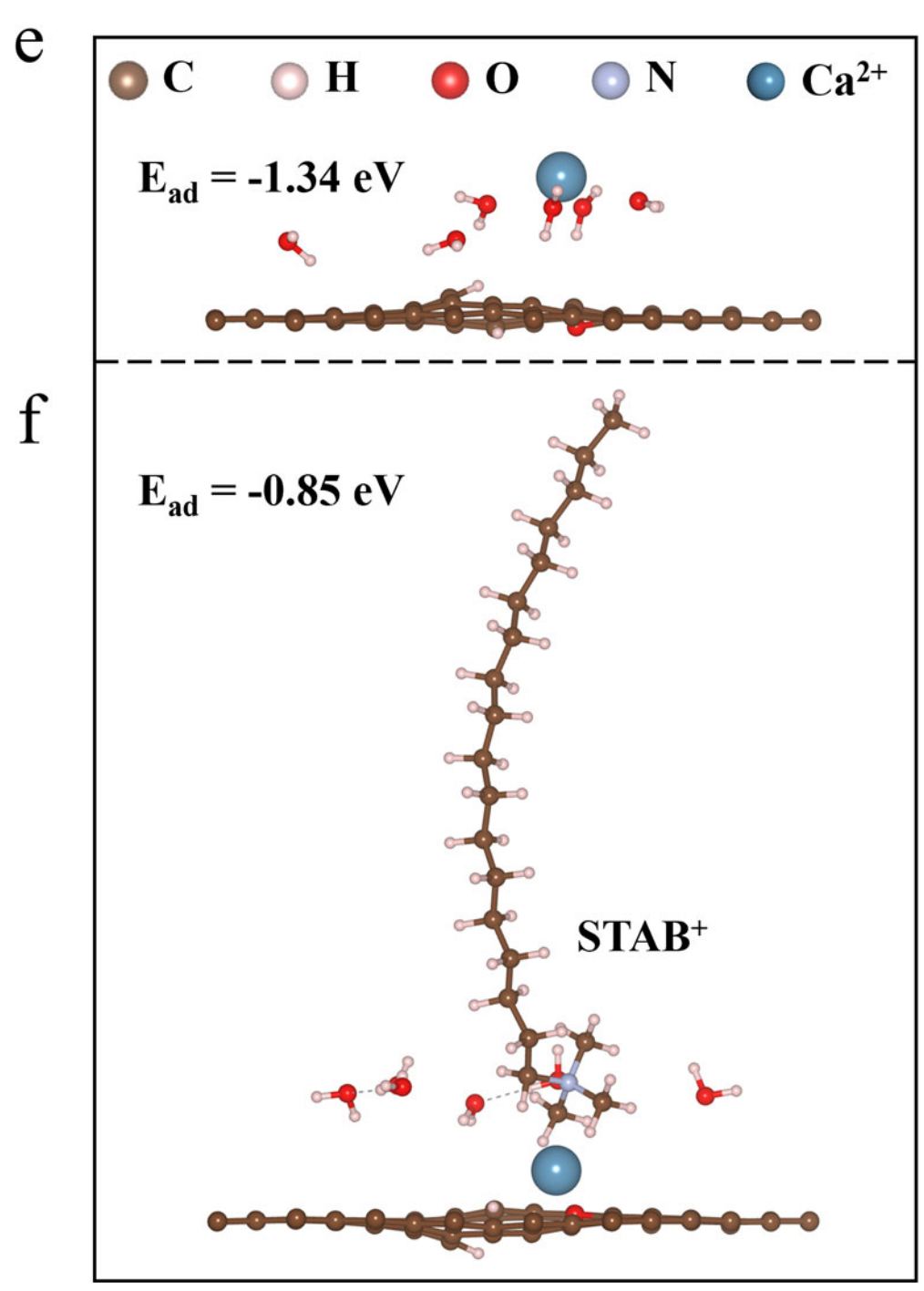

Fig. 4. The adsorption energies (E_{ad}) for Ca^{2+} on the surfaces of (e) C0 and (f) C18 cathodes obtained from DFT calculations.

- **Replaced Figures in Supplementary Information:**
  Supplementary Figs. 11 and **12** in the Supplementary Information have been replaced and are now renumbered as **Supplementary Figs. 16 and 17**.

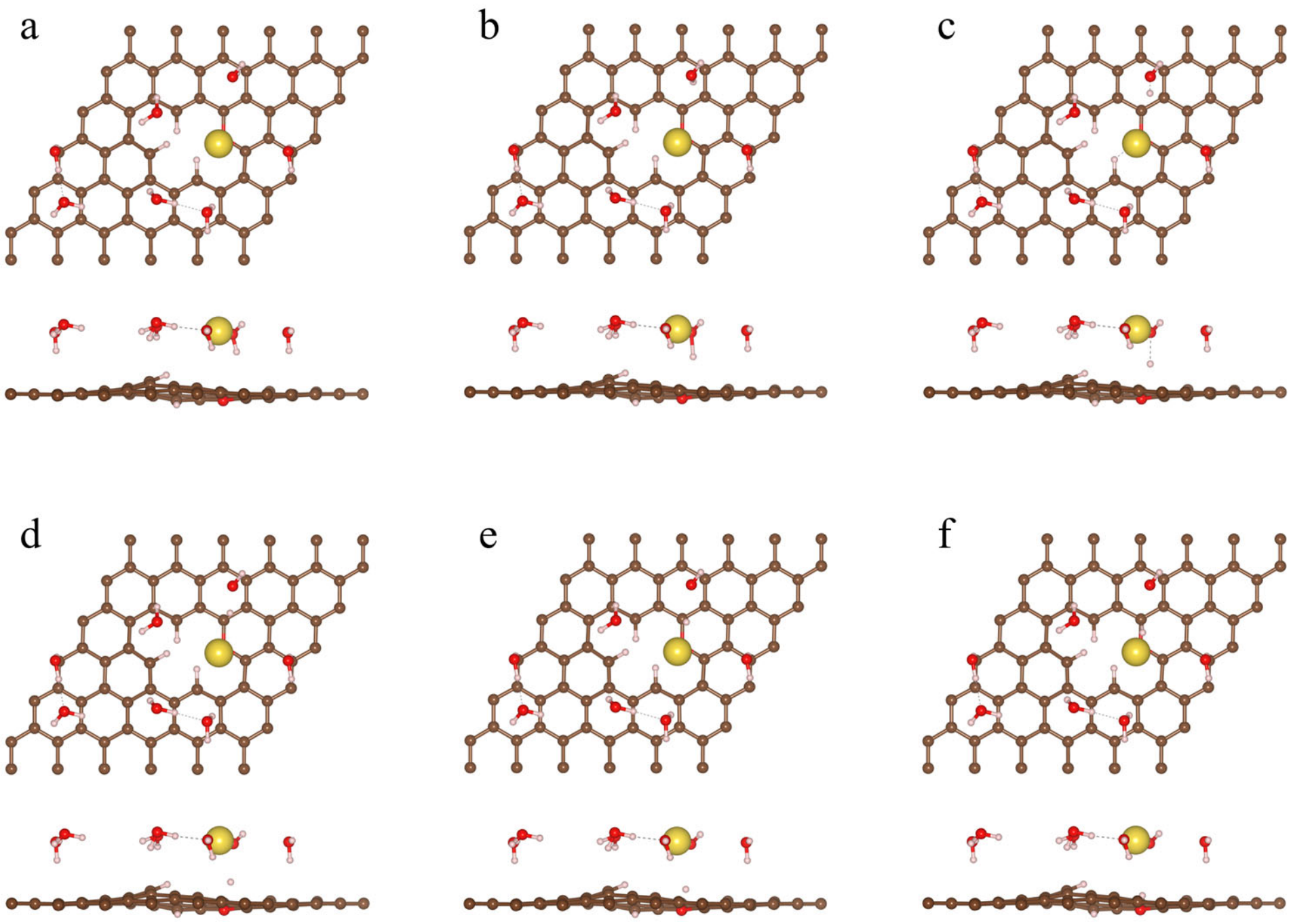

Supplementary Fig. 16. Atomic configurations of water dissociation step on the graphene surface with Na^{+}: (a) initial state, (b-e) transition state and (f) final state. The brown, red, pink, and yellow spheres represent C, O, H and Na atoms, respectively.

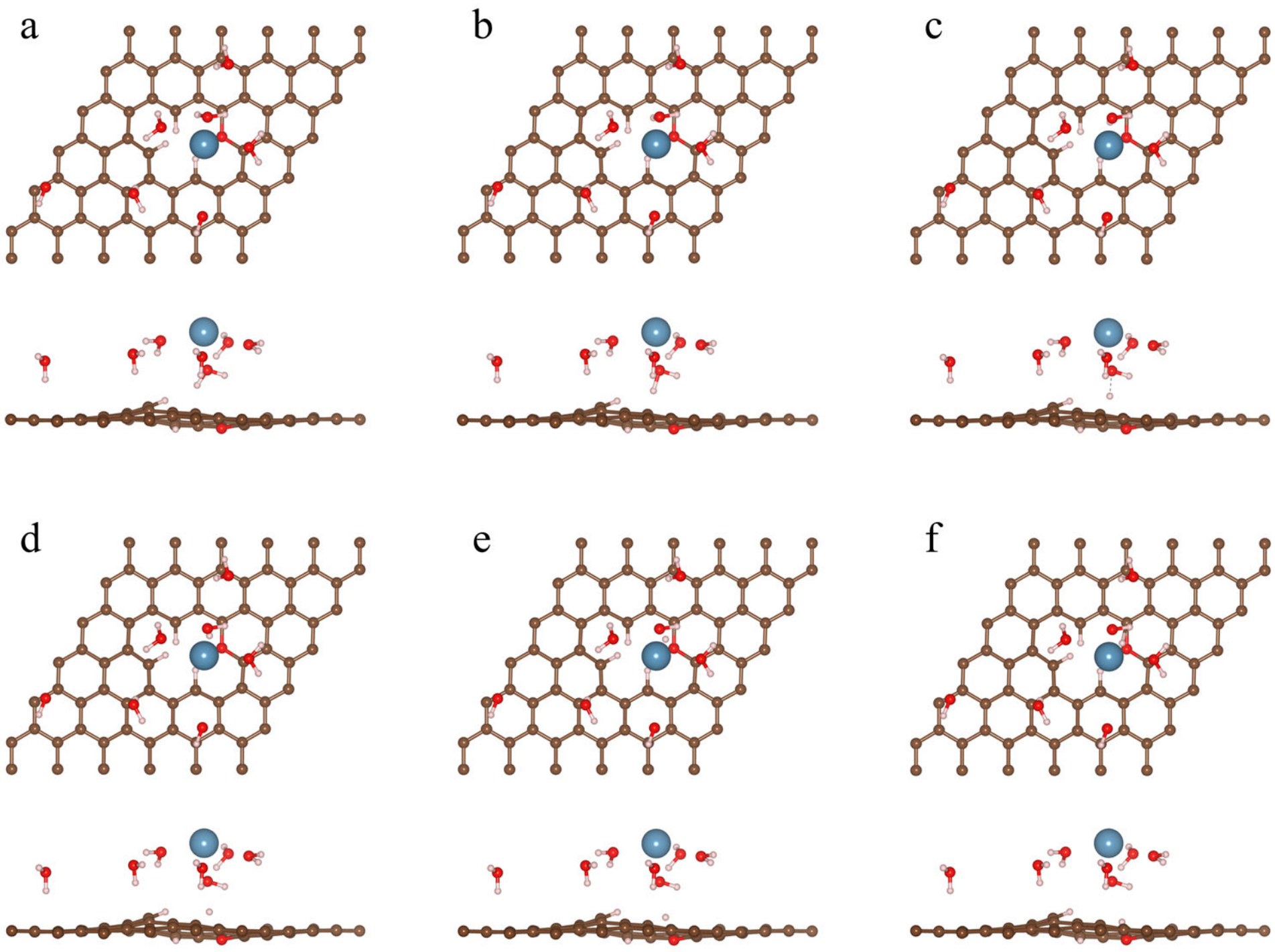

Supplementary Fig. 17. Atomic configurations of water dissociation step on the graphene surface with Ca^{+}: (a) initial state, (b-e) transition state and (f) final state. The brown, red, pink, and blue spheres represent C, O, H and Ca atoms, respectively.

- **New Figures in Supplementary Information:**

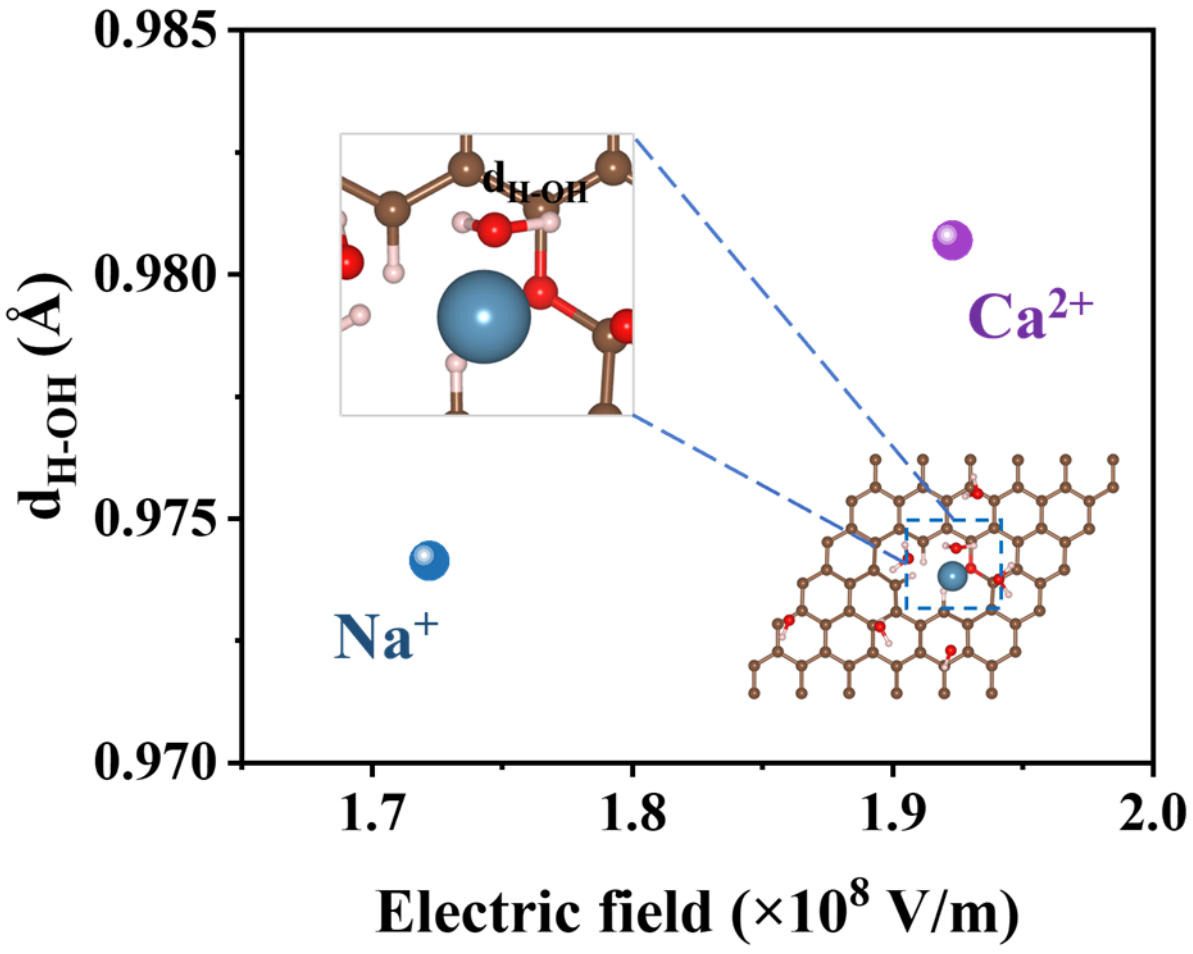

Supplementary Fig. 18. The measured bond length of H-OH bond in Na^{+} and Ca^{2+} systems.

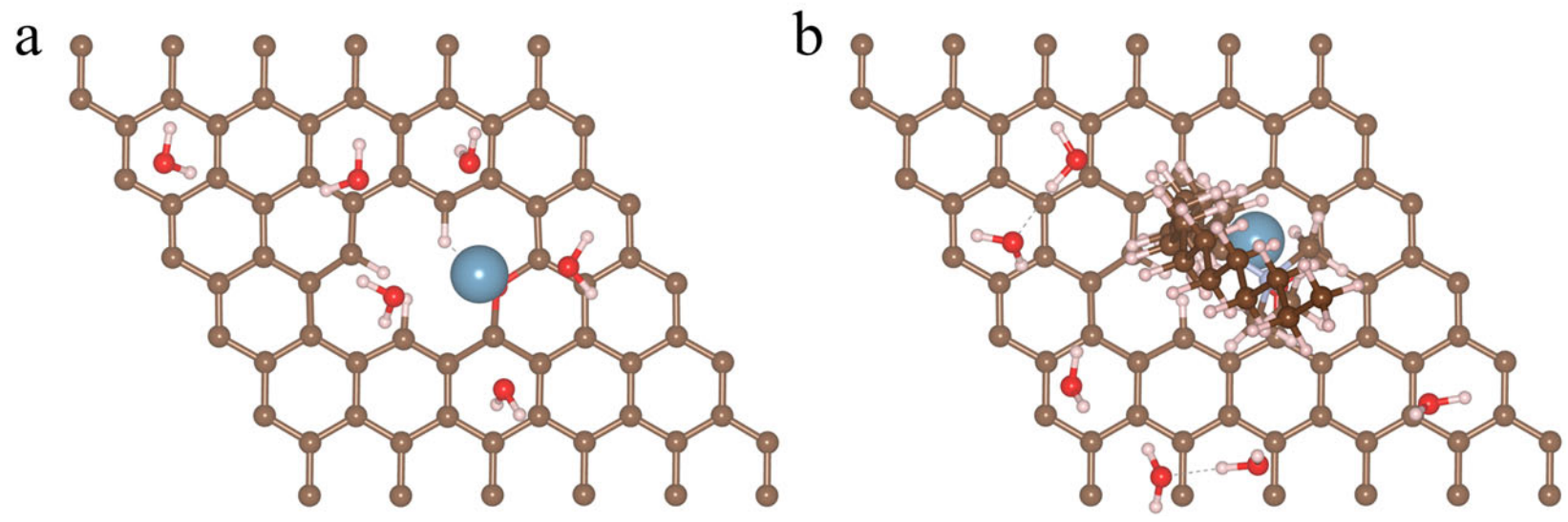

Supplementary Fig. 54. The adsorption planar models for Ca^{2+} on the surfaces of (a) C0 and (b) C18 cathodes. The brown, red, pink, and blue spheres represent C, O, H and Ca atoms, respectively.

- **Updated Methods Section**

Density functional theory (DFT) calculations [Line 862-874, Page 26 (Revised)]

A water bi-layer was included to describe explicitly the polarization involved in water dissociation. The implicit solvation effects were implemented using the VASPsol package.

The electric field strength at the interface between the graphene surface and the electrolyte was evaluated using a parallel-plate capacitor model[16]. The electric field strength (E) was calculated according to the following equation: $E = (Q_1+Q_2) / (2\varepsilon S)$, where Q_1 and Q_2 represent the net charges of the graphene slab and the electrolyte, respectively, as determined by Bader charge analysis; S represents the surface area of the graphene slab; and ε represents the absolute permittivity of water.

The adsorption energies of *OOH or Ca^{2+} on the surface of C0 or C18 were calculated by the formula: $E_{ad} = E_{total} - E_{base} - E_{adsorbate}$, where E_{total} is the total energy of the absorbed system, E_{base} is the basement energy and $E_{adsorbate}$ is the energy of adsorbate.

- **New reference**

28. Cao, P. *et al.* Cation-tuned acidic electrified interface for hydrogen peroxide electrosynthesis with industrial-level current densities in natural seawater. *Nature Communications* **17**, 5443 (2026).

6. In the latter half of the manuscript, the authors introduce a C18 surfactant modification and a 3D-PUS sponge fenced strategy to demonstrate the engineering application value of their mechanism. However, the introduction of these two strategies adds more variables to the electrochemical reaction, leading to a somewhat confused core logical chain. The C18 modification (with the introduction of long alkyl chains) not only alters the Zeta potential but significantly changes the electrode's wettability. The authors must provide control experiments to decouple the effects of improved physical mass transport from electrostatic repulsion, rather than attributing the performance enhancement solely to the repulsion of Ca^{2+}. Furthermore, regarding the 3D-PUS strategy, the physical barrier of the sponge inherently hinders OH^- diffusion and elevates local pH, an effect that typically improves $2e^-$ ORR selectivity in other systems anyway (even in the absence of calcium). Overall, the engineering optimization in the latter half fails to further solidify the fundamental chemical mechanism proposed in the first half; instead, by mixing in additional variables, it blurs the main storyline of the manuscript.

Response:

Thank you for this exceptionally insightful and constructive critique. We agree that the introduction of C18 surfactant modification and the 3D-PUS architecture add more variables without adequately decoupling their individual contributions. We fully accept this criticism and have conducted systematic control experiments to disentangle these effects. We are grateful for the opportunity to substantially improve the mechanistic narrative of our work.

- **(1) Decoupling electrostatic repulsion effect from hydrophobicity-enhanced mass transport of C18**

  We agree that the C18 modification simultaneously alters surface charge (zeta potential from -25.26 to +15.48 mV) and wettability (contact angle from 59.34° to 132.67°), and that the performance enhancement cannot be solely attributed to electrostatic Ca^{2+} repulsion without ruling out the contribution of improved physical mass transport. To decouple these two effects, we designed control experiments using an additional material as follows:

  (a) N-C18 (non-ionic long-chain surfactant-modified C0) preparation and characterization: Modified with 1-Octadecanethiol (ODT). The physical characterization of N-C18 is presented in new **Supplementary Fig. 58**. This material is hydrophobic (contact angle 134.02°, comparable to C18) but carries no positive charge (zeta potential -23.05 mV, comparable to C0), thereby isolating the hydrophobic effect.

  (b) Performance comparison among C0, N-C18, and C18 in the presence of 200 mg/L Ca^{2+} at 0.1 A: The FE of N-C18 was 15.60%, representing a 29.95% enhancement relative to C0 (12.00%). However, the FE of C18 reached 32.22%, corresponding to a 168.66% increase over C0 (12.00%). These results demonstrate that hydrophobicity-enhanced mass transport (N-C18 vs. C0) contributes approximately 17.8%, providing a significant but secondary benefit. Electrostatic Ca^{2+} repulsion contributes approximately 82.2% of the total C18 enhancement, confirming it as the dominant mechanism.

We have therefore refined our mechanistic attribution: the C18 performance enhancement is driven primarily by electrostatic Ca^{2+} repulsion (~82.2%), with hydrophobicity-driven mass transport and H_2O_2 desorption providing a supplementary contribution (~17.8%). Both contributions are now explicitly quantified and discussed.

- **(2) Decoupling the general alkaline effect of 3D-PUS strategy from its Ca^{2+}-specific management effect**

Thank you for the insightful suggestion to decouple the general alkaline enhancement effect on $2e^-$ ORR of the 3D-PUS architecture from its Ca^{2+}-specific management effect. As recommended, we have conducted additional control experiments to comprehensively evaluate the H_2O_2 electrosynthesis performance of bare and PUS-fenced cathodes in Ca^{2+}-free and Ca^{2+}-containing electrolyte. The new experimental results are presented in the revised Supplementary Information **(Supplementary Fig. 62)**. The key findings are as follows:

(a) The 3D-PUS strategy does not enhance $2e^-$ ORR selectivity in the absence of Ca^{2+}.

In Ca^{2+}-free electrolyte (0.05 M Na_2SO_4), the FE of the C0-PUS cathode (51.26%) was comparable to that of the bare C0 cathode (52.41%) at 0.1A. The C18-PUS configuration demonstrated similar behavior. These results demonstrate that the pore confinement-induced alkaline microenvironment alone does not improve the $2e^-$ ORR selectivity under our experimental conditions in the absence of Ca^{2+}. Despite the enhanced local alkalinity favors the $2e^-$ pathway, the introduction of PUS concomitantly increased the mass transfer resistance for H_2O_2, which may account for the lack of improvement in its apparent yield.

(b) The 3D-PUS strategy significantly enhances $2e^-$ ORR selectivity in the presence of Ca^{2+}.

In contrast, the PUS strategy delivers a dramatic enhancement only in Ca^{2+}-containing electrolyte.

For C0 at 0.1A: The C0-PUS cathode achieved a 293.3% enhancement in H_2O_2 yield over the bare C0 (550.26 versus 139.96 mmol $g_{cat.}^{-1}$ h^{-1}).

For C18 at 0.1A: The C18-PUS cathode exhibited a significantly higher H_2O_2 yield of 611.88 mmol $g_{cat.}^{-1}$ h^{-1}, which is 1.63 times that of bare C18 (376.04 mmol $g_{cat.}^{-1}$ h^{-1}).

The striking divergence between the Ca^{2+}-free and Ca^{2+}-containing results directly demonstrates that the PUS-induced enhancement is predominantly Ca^{2+}-specific: the pore confinement restricts OH^- diffusion, creating a localized alkaline microenvironment that reduces the interfacial concentration of dissolved Ca^{2+} and mitigates its detrimental catalytic effect on H_2O_2 reduction. We have revised the Discussion to clearly distinguish the Ca^{2+}-specific enhancement from any general alkaline effect.

- **(3) Revised logical chain of the manuscript**

With these decoupling experiments, the core storyline of our manuscript now exhibits coherently from fundamental mechanism to engineering strategy:

(a) Fundamental mechanism (first half): Dissolved Ca^{2+}, not precipitated scale, is the primary inhibitor of $2e^-$ ORR, predominantly by accelerating the further reduction of in-situ generated H_2O_2 to H_2O (the P2H step).

(b) Strategy 1–Interfacial field modulation (C18): The positively charged surface electrostatically repels Ca^{2+} from the interface (dominant mechanism, ~82.2% contribution), while the hydrophobic chains improve O_2 transport and H_2O_2 desorption (secondary mechanism, ~17.8% contribution). Both effects synergistically suppress the Ca^{2+}-induced P2H side reaction.

(c) Strategy 2–3D alkaline confinement (PUS-fenced): The pore confinement effect functions specifically through Ca^{2+} management—restricting OH^- diffusion to reduce interfacial dissolved Ca^{2+}—rather than through a general alkaline enhancement of $2e^-$ ORR selectivity.

The revised logical chain now directly connects each engineering strategy to the core chemical mechanism established in the first half of the manuscript. Specifically, dissolved Ca^{2+} was demonstrated to be the primary inhibitor of the $2e^-$ ORR. Based on this mechanistic understanding, two strategies were developed to optimize interfacial Ca^{2+} management, thereby enhancing H_2O_2 electrosynthesis performance.

The Revised Content in Manuscript

Based on your comments, we have conducted control experiments to disentangle the effects of C18 surfactant modification and the 3D-PUS architecture on Ca^{2+}-tolerance. The following specific revisions have been implemented in the manuscript and supporting information:

- **Replaced Discussion in Section "Mechanistic study for enhanced Ca^{2+}-tolerance of surfactant-modified cathodes." [Line 492-504, Page 15 (Revised)]**
  Furthermore, to decouple the electrostatic and hydrophobic contributions of the C18 modification, a non-ionic long-chain surfactant-modified cathode (named N-C18) was prepared using 1-octadecanethiol (ODT). XRD, Raman and XPS spectra confirmed the successful modification of N-C18 (**Supplementary Fig. 58**). N-C18 exhibited a contact angle of 134.02° (comparable to C18 at 132.67°) while retaining a zeta potential of -23.05 mV (comparable to C0 at -25.26 mV) (**Supplementary Fig. 58**), thereby isolating the hydrophobic effect from electrostatic repulsion. Compared with C0, N-C18 exhibited a 29.95% improvement in performance in Ca^{2+}-containing electrolyte, whereas C18 achieved a 168.66% enhancement (**Supplementary Fig. 59**). These results indicate that the hydrophobicity-driven mass transport plays a secondary role in enhancing Ca^{2+}-tolerance (~17.8% contribution), whereas the electrostatic effect constitutes the dominant mechanism (~82.2% contribution), which mitigates the adverse effects of dissolved Ca^{2+} on H_2O_2 electroproduction.
- **New Discussion in Section "Enhanced Ca^{2+}-tolerance by a fenced cathode structure." [Line 532-536, Page 17 (Revised)]**
  Notably, the FEs of C0-PUS and C18-PUS cathodes were comparable to that of the bare counterpart in Ca^{2+}-free electrolyte (**Supplementary Fig. 62**). The striking divergence between the Ca^{2+}-free and Ca^{2+}-containing results directly demonstrates that the PUS-induced enhancement is predominantly Ca^{2+}-specific rather than any general alkaline effect.
- **New Figures in Supplementary Information:**

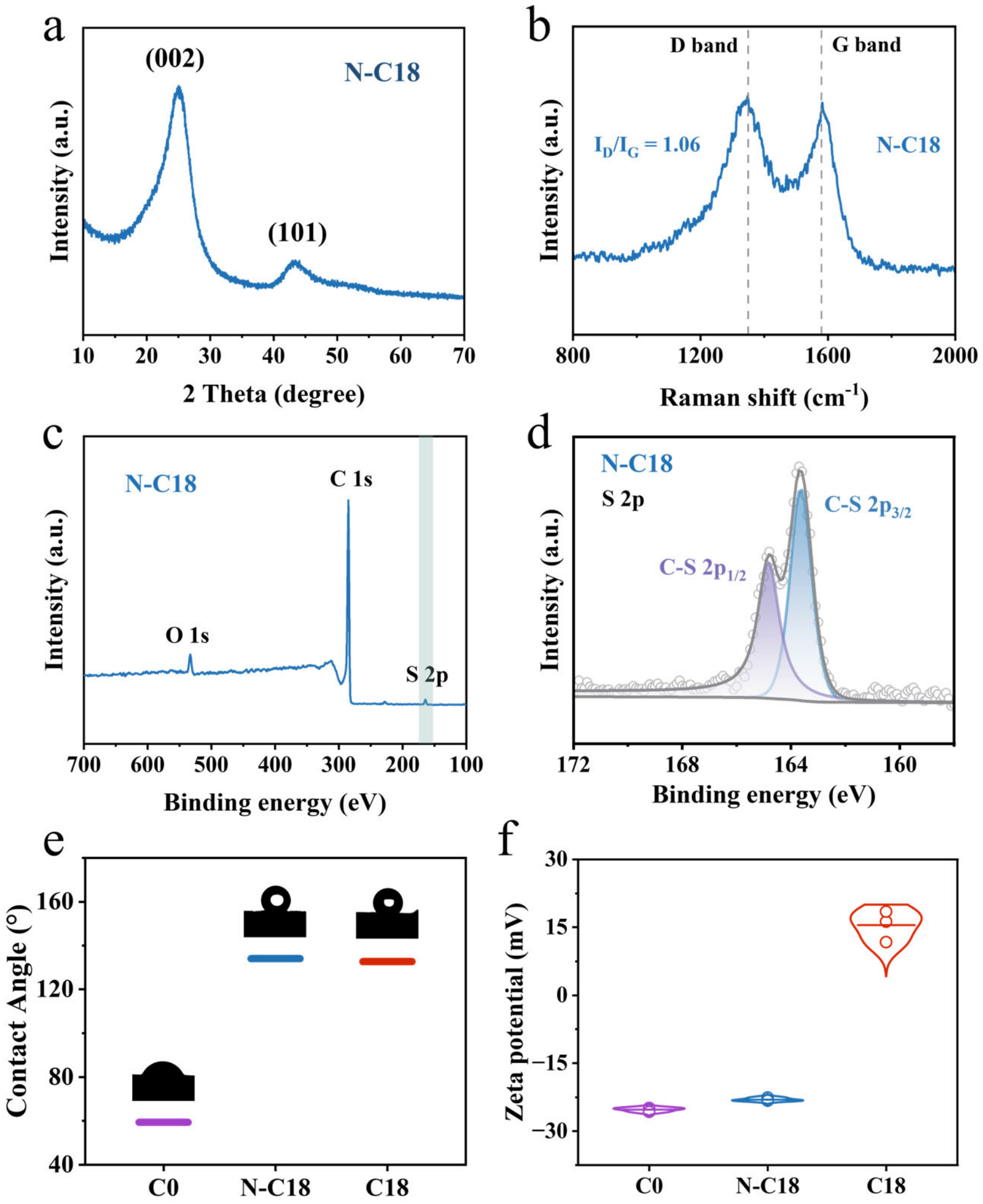

Supplementary Fig. 58 (a) XRD pattern and (b) Raman spectra of the N-C18 catalyst. (c) XPS spectra and (d) high-resolution S 2p spectra of N-C18. (e) Contact angle and (f) Zeta potential measurements of C0, N-C18, and C18.

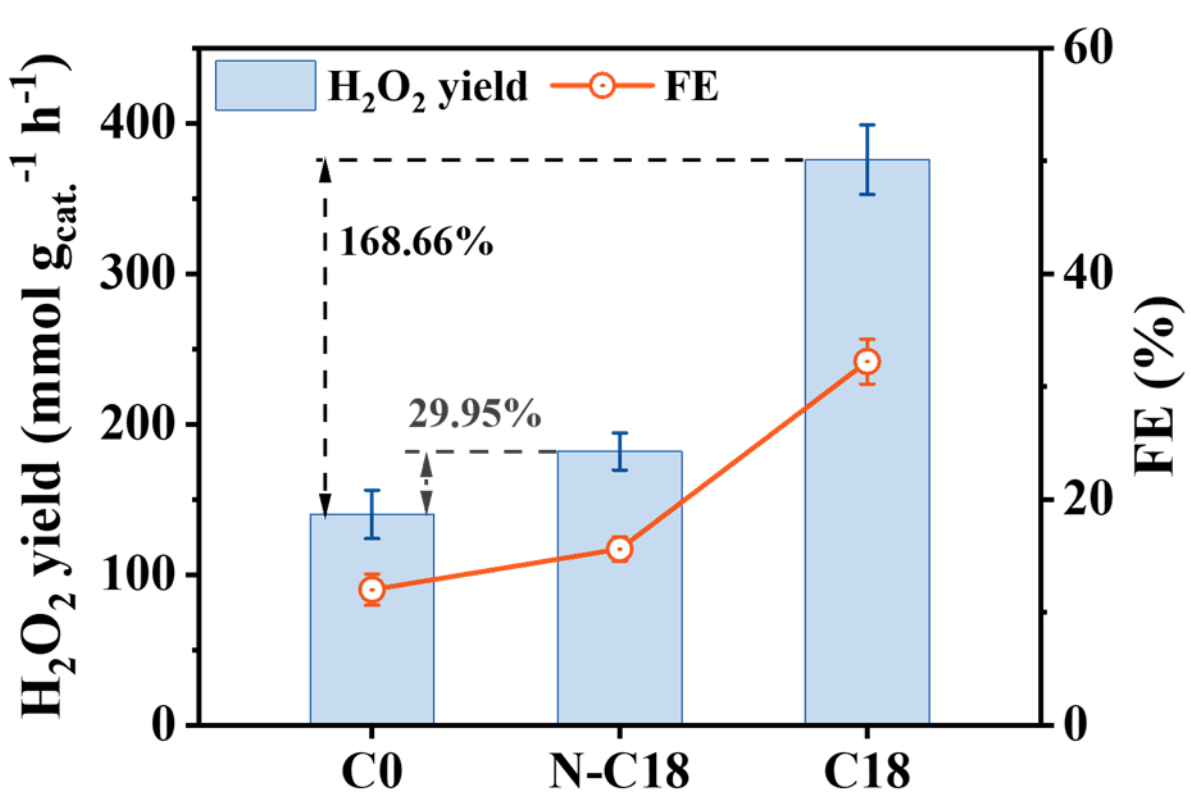

Supplementary Fig. 59 H_2O_2 electrosynthesis performance of the C0, N-C18, and C18 cathodes in the presence of Ca^{2+}. Reaction conditions: $[Na_2SO_4]$ = 0.05 M, $[Ca^{2+}]_0$ = 200 mg/L, a current of 0.1 A, a rotation speed of 600 rpm, an initial pH of 7 and electrolysis of 3 h.

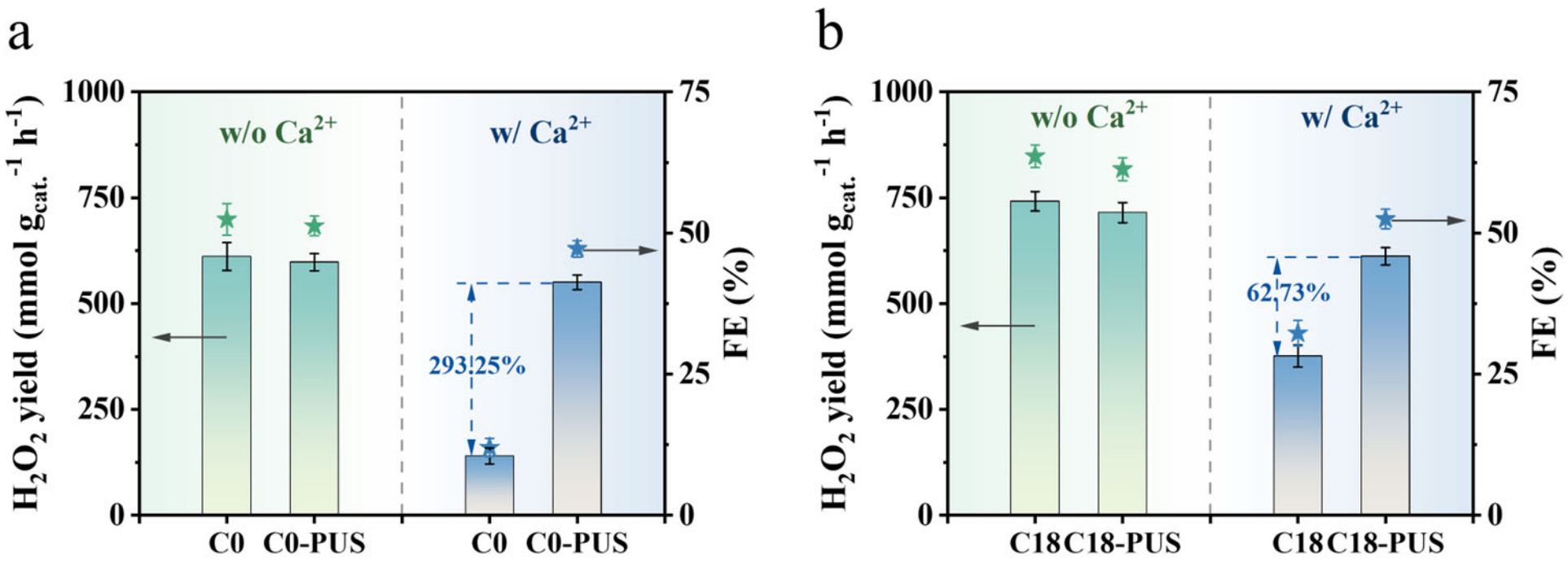

Supplementary Fig. 62 (a) H_2O_2 electrosynthesis performance of the C0 and C0-PUS cathodes in the absence (w/o) or presence (w/) of 200 mg/L Ca^{2+}. (a) H_2O_2 electrosynthesis performance of the C18 and C18-PUS cathodes in the absence (w/o) or presence (w/) of 200 mg/L Ca^{2+}. Reaction conditions: [Na_2SO_4] = 0.05 M, a current of 0.1 A, a rotation speed of 600 rpm, an initial pH of 7 and electrolysis of 3 h.

- **Updated Methods Section**

Chemicals and materials [Line 637-641, Page 20 (Revised)]

Potassium titanium oxalate ($C_4H_2K_2O_{10}Ti$, purity > 99%), 1-Octadecanethiol (ODT), Octyltrimethylammonium bromide (OTAB), decyltrimethylammonium bromide (DTAB), tetradecyltrimethylammonium bromide (TTAB), and stearyltrimethylammonium bromide (STAB) were purchased from Shanghai Macklin Biotechnology Co., Ltd.

Synthesis of surfactants modified oxygen-doped carbon black [Line 662-667, Page 21 (Revised)]

Synthesis of ODT-OCB (named N-C18, non-ionic long-chain surfactant-modified C0). To prepare the non-ionic long-chain surfactant-modified OCB catalyst, 60 mg of OCB and 240 μmol of ODT were added into 20 mL of anhydrous ethanol, followed by sonication for 1 h and stirring for 24 h to obtain a homogeneous suspension. The final samples were collected through vacuum filtration followed by washing with plenty anhydrous ethanol, and were subsequently vacuum-dried for 24 h at 60°C.

7. Although the title, abstract, and conclusions heavily frame this work around applications in “natural water,” the primary experimental conditions presented in the main text and supplementary materials indicate that the vast majority of the experiments were actually conducted in simplified model systems (e.g., 0.05 M Na_2SO_4 with added Ca^{2+}, or $NaCl/CaCl_2$). Real natural water matrices are highly complex, involving the simultaneous and interactive presence of not only Ca^{2+} but also Mg^{2+}, bicarbonate, natural organic matter, sulfate, chloride, and other constituents. By attributing nearly all performance variations solely to calcium ions without clearly delineating the boundaries of applicability, the manuscript’s claim of “universal applicability in natural water” is overstated.

Response:

Thank you for this important critique regarding the gap between our claims of "natural water applicability" and the predominant use of simplified model systems in our experiments. We acknowledge that the real natural water matrices are far more complex than the Na_2SO_4 + Ca^{2+} model systems we primarily employed, and that our original statement overstated the universality of our conclusions. We fully accept this criticism and have substantially revised the manuscript to appropriately delineate the boundaries of applicability.

- **(1) We acknowledge the overstatement and have revised our claims accordingly.**
  Attributing performance variations in natural water almost exclusively to Ca^{2+}, without adequately discussing the potential roles of other co-existing constituents, constitutes an overstatement. We have made the following specific revisions throughout the manuscript:
  Abstract: We have removed "natural water" from generalized claims. The abstract now explicitly states that the strategies provide a paradigm for designing Ca^{2+}-tolerant cathodes, rather than claiming universal natural water applicability.
  Introduction and Results: We have added explicit notes acknowledging that real natural water contains multiple interacting constituents (Mg^{2+}, HCO_3^-, natural organic matter, etc.) that may influence performance in ways not captured by our simplified model systems. We now clearly state that our mechanistic conclusions regarding Ca^{2+} are derived from simplified Ca^{2+}-containing electrolytes, and that the real water tests serve as initial proof-of-concept rather than exhaustive validation.
  Discussion: The original claim of "establishing a new paradigm for designing efficient and stable electrocatalytic systems in natural water" has been revised to "establishing a new paradigm for designing Ca^{2+}-tolerant cathodes, with potential promise for extension to natural water matrices."
- **(2) We clarify the reason and limitations of our experimental approach.**
  Our choice to focus on simplified model systems (0.05 M Na_2SO_4 + Ca^{2+}) was deliberate and methodologically necessary for mechanistic investigation. The complex interplay of multiple ions and organic species in real water would make it impossible to isolate the specific role of Ca^{2+} and to validate our central hypothesis that dissolved Ca^{2+}, rather than precipitated scale, is the dominant inhibitor of $2e^-$ ORR. We have now explicitly demonstrated this reason in the revised Methods section, while also acknowledging its limitations:

Simplified systems enable rigorous mechanistic decoupling but cannot capture all synergistic/antagonistic effects in real water.

The four real water matrices tested (tap water, river water, groundwater, municipal wastewater) serve as a preliminary demonstration that the C18-PUS strategy can maintain reasonable performance under realistic conditions, but do not constitute exhaustive validation across all water constituents.

- **(3) We reiterate the innovation of our core mechanistic finding.**
  Despite the simplified nature of our experimental matrix, we respectfully submit that the core mechanistic insight—dissolved Ca^{2+}, rather than precipitated scale, is the primary inhibitor of $2e^-$ ORR—remains a conceptually important contribution. This finding fundamentally reframes the rational design of Ca^{2+}-tolerant cathodes for Ca^{2+}-containing water, shifting focus from the single scale prevention to dissolved Ca^{2+} management.

The Revised Content in Manuscript

Based on your comments, we have substantially revised the manuscript to appropriately delineate the boundaries of applicability. The following specific revisions have been implemented in the manuscript:

(1) Abstract: These two strategies of interfacial field manipulation and 3D alkaline confinement provide a new paradigm for designing Ca^{2+}-tolerant cathode for efficient H_2O_2 electrosynthesis. **[Line 35-37, Page 2 (Revised)]**

(2) Introduction: This work provides novel perspectives into the distinct roles of dissolved Ca^{2+} versus calcium scale in $2e^-$ ORR and establishes a new paradigm for designing Ca^{2+}-tolerant cathodes for H_2O_2 electrosynthesis. Combining experimental and theoretical approaches, we aim to: …… (3) further propose a 3D polyurethane sponge (PUS) fenced cathode configuration to facilitate a highly alkaline microenvironment for efficient H_2O_2 electrosynthesis. **[Line 81-91, Page 3 (Revised)]**

(3) Discussion: In conclusion, this work pioneers two strategies including surfactant-induced interfacial field control and 3D fenced-cathode confinement that enables simultaneous Ca^{2+} repulsion, proton-transfer suppression, and alkaline microenvironment localization, establishing a new paradigm for designing Ca^{2+}-tolerant cathodes, with potential promise for extension to natural water matrices. **[Line 628-632, Page 20 (Revised)]**

- **Updated Methods Section**
  We have now explicitly demonstrated the reason for selected electrolytes in the revised Methods section. The specific revisions have been implemented as follows:

  H_2O_2 electrosynthesis in GDEs [Line 678-683, Page 21 (Revised)]

  H_2O_2 electrosynthesis experiments were conducted in a custom-made undivided electrochemical reactor, as illustrated in **Supplementary Fig. 77**. To avoid confounding effects from interactions among the complex components present in real water matrices, a 100 mL Na_2SO_4 (0.05 M) solution without or with Ca^{2+} served as the supporting electrolyte, ensuring that any observed impacts could be attributed solely to Ca^{2+}. The solution was continuously stirred at 600 rpm to ensure sufficient mass transfer.

Modifications to comments from Reviewer #3

General comments

Summary: This manuscript targets Ca^{2+}-related performance loss in electrochemical H_2O_2 production in realistic aqueous systems. Authors argue that dissolved Ca^{2+} cation rather than precipitated $CaCO_3$ scale is the dominant source of selectivity loss and propose two mitigation strategies: (i) employment of quaternary ammonium surfactants to modify the oxygen-doped carbon cathode (C0) with tunable surface wettability and electrostatic potential (C8, C10, C14 and C18) and (ii) incorporation of a 3D polyurethane sponge (PUS) on the cathode surface to induce a localized alkaline environment. Authors managed to improve H_2O_2 selectivity and Ca^{2+} tolerance using modified electrodes and accomplish their mechanistic interpretation using scaling characterization, electrochemical probes, and MD/DFT calculations. I am a computational chemist, so my comments will focus more on mechanistic and computational components of the manuscript.

Specific comments

Major Issues:

1. Central paradox of more scaling yet better performance is not convincingly resolved. A striking observation is that C18 can deposit more $CaCO_3$ scaling than C0 while delivering superior Ca^{2+} tolerance and higher H_2O_2 selectivity. Authors frame this as a challenge to the prevailing opinion about the detrimental impact of scaling. However, the mechanistic explanation remains incomplete. For example, why does additional scaling on C18 not systematically correlate with an improved protection, and how do the surface location and morphology of scales affect mass transport and active site accessibility? At minimum, the manuscript should clarify where scales form (surface vs pores vs phase boundary) and demonstrate their effects in mass transport.
2. Multiple mechanisms are invoked without being disentangled or quantified. Authors attribute the improved selectivity to a combination of multiple factors, including (i) exclusion of Ca^{2+} from a more positive surface potential, (ii) disruption of hydrogen bond networks and limited proton transfers via hydrophobicity, (iii) formation of a highly alkaline microenvironment from the PUS confinement, and (iv) suppression of side reactions like H_2O_2 reduction and decomposition from protective roles of scales. All these effects can compete and overlap with each other, and authors should perform an ablation study to elucidate which factor is more deterministic. To “make up” some examples, dissolved Ca^{2+} may suppress the synthesis of H_2O_2 by accelerating O−O dissociation, and C18 may play an opposite role by slowing proton transfers. Without even a simplest rate and transport analysis, the conclusions read as a list of narratives rather than testable mechanisms.
3. Electrostatic interpretation based on PZC/zeta potential is oversimplified. Authors link the exclusion of Ca^{2+} to a more positive surface potential of C18. However, PZC and zeta potential measured under non-operando conditions cannot be directly mapped onto the interfacial charge and potential. Authors should discuss the relationship between PZC, electrode potential, specific adsorption, and double-layer structure. Moreover, authors suggest enhanced interfacial cation density and claim electrostatic

repulsion of Ca^{2+}, which appears contradictory unless clarified.
4. Claims about highly alkaline interfacial microenvironment rely on questionable proxies. Authors argue PUS-fenced electrode to restrict OH^- diffusion and therefore create a localized alkaline microenvironment that suppresses the Ca^{2+} activity, but they bulk pH values taken ~1 cm from the electrode, which is not a reliable proxy. Authors should either (i) provide more appropriate interfacial pH diagnostics (e.g., microelectrode profiling at the surface, spectroscopic probes, or modeling constrained by measured currents), or (ii) substantially soften this claim and reframe it as a meso-scale transport effect.
5. DFT and MD modeling do not fully support the mechanistic claims. Reported MD simulations (20 ns NVT, GAFF/RESP, explicit electrolyte) provide structural information but do not establish proton transfer kinetics. Therefore, the claim about slower proton transfer kinetics from reduced hydrogen bond or water density is not directly grounded. Charge and potential of the electrode are not physically implemented and the claim about the repulsion of Ca^{2+} is not straightforward either. For DFT calculations, while the computational workflow is standard (VASP PBE/D3, NEB), the treatment for Ca^{2+} adsorption and energetics is incomplete without being fully solvated by water molecules.

Minor Issues:
1. Authors should clarify the rationale for selecting the specific active sites and models used in DFT, and whether alternative defects and functional group sites were tested.
2. Authors interpret results from electrochemical impedance spectroscopy (EIS) (e.g., “hydrogen adsorption resistance”) as evidence of slower proton transfers. They should provide the equivalent circuit, fitting quality, parameter identifiability, and a clearer connection between these parameters, and the surface availability of protons.
3. Authors should discuss the diffusion and H-type cell experiments used to support Ca^{2+} exclusion more carefully with respect to their transferability to polarized cathode surfaces.
4. Authors mention that C18 differs from C0 not only in surfactant grafting but also in defect level and possibly surface chemistry. They should clarify how these confounding factors are controlled.
5. Authors should provide error bars, replicate numbers, and statistical treatment consistently for performance metrics (FE, production rate, stability) and scaling quantification (ICP/EDS/profilometry).

Recommendation: The work contains potentially publishable observations and a practically relevant direction: Ca^{2+}-tolerant H_2O_2 electrosynthesis in realistic aqueous systems. However, the mechanistic understanding has not reached standard expected for Nature Communications. I recommend a major revision, with emphasis on major and minor issues mentioned above.

Major Issues:
1. Central paradox of more scaling yet better performance is not convincingly resolved. A striking observation is that C18 can deposit more $CaCO_3$ scaling than C0 while delivering superior Ca^{2+} tolerance and higher H_2O_2 selectivity. Authors frame this as a challenge to the prevailing opinion about the detrimental impact of scaling. However, the mechanistic explanation remains incomplete. For example, why does additional scaling on C18 not systematically correlate with an improved protection, and how do the surface location and morphology of scales affect mass transport and active site accessibility? At minimum, the manuscript should clarify where scales form (surface vs pores vs phase boundary) and demonstrate their effects in mass transport.

Response:

Thank you for this insightful critique regarding the central paradox of "more scaling yet better performance". We appreciate the opportunity to clarify our position and provide a more complete mechanistic explanation.

- **(1) Clarifying our position**

  We wish to first clarify that we do not intend to claim that calcium scale is overall beneficial, nor do we assert that "more scale is better." Our experiments do reveal an apparent paradox: the C18 cathode deposits more calcium scale yet delivers higher H_2O_2 selectivity than C0. Nevertheless, we do not intend to dismiss the conventional understanding of scale-induced performance loss. On the contrary, we have carefully examined these negative effects including aggravated water flooding and inhibited oxygen transport (**Supplementary Figs. 19, 20, 49 and 51**), while also discovering an unexpected protective aspect of the scale. More importantly, our intention is not to argue that "more scale is better", but rather to draw attention to the overlooked role of dissolved Ca^{2+} from this counterintuitive phenomenon. The central contribution of this work is the clarification that dissolved Ca^{2+}, rather than precipitated calcium scale, serves as the dominant inhibitor of the $2e^-$ ORR. Guided by this mechanistic understanding, we further developed two interfacial Ca^{2+} management strategies to mitigate Ca^{2+}-induced inhibition and thereby improve H_2O_2 electrosynthesis performance.

- **(2) Why additional scaling on C18 does not systematically correlate with improved protection**

  In the H_2O_2 decomposition experiments, the increase in calcium scale indeed exerted a more pronounced protective effect by suppressing further decomposition of H_2O_2 at the cathode. However, the net H_2O_2 electrosynthesis performance in Ca^{2+}-containing electrolyte is governed by the interplay of three experimentally verified factors:

  The inhibitory effect of dissolved Ca^{2+} (dominant): Dissolved Ca^{2+} enhances interfacial water dissociation and accelerates the cathodic reduction of H_2O_2 to H_2O, thereby shifting the ORR pathway toward $4e^-$ direction and reducing net H_2O_2 selectivity.

  The dual role of precipitated calcium scale (secondary): Scale exerts a negative effect by enhancing water flooding and inhibiting oxygen transport (**Supplementary Figs. 19 and 20**), but simultaneously exerts a partially protective effect by suppressing the cathodic reduction of H_2O_2 (**Supplementary Figs. 22-24**).

The competition between the above effects determines the net performance. For example, as the total Ca^{2+} concentration in the electrolyte increases, both the amount of precipitated scale and the concentration of dissolved Ca^{2+} increase simultaneously. Because dissolved Ca^{2+} is the dominant factor, the rising dissolved Ca^{2+} concentration drives further performance degradation that overwhelms any incremental protective benefit from the additional scale. This is evidenced by the fact that the FE does not increase monotonically with increasing Ca^{2+} concentration despite greater scaling (**Figs. 3b**). At higher Ca^{2+} concentrations, the protective effect of the increasing scale and the inhibitory effect of dissolved Ca^{2+} approach a dynamic balance, and the apparent H_2O_2 FE stabilizes.

Therefore, the more extensive scaling on C18 is not a main cause of its improved performance, but rather a consequence of its effective Ca^{2+} management strategy: the positively charged surface of C18 electrostatically repels Ca^{2+} from the interface, thereby mitigating its catalytic effect on the parasitic H_2O_2 reduction pathway.

- **(3) How do the surface location and morphology of scales affect mass transport and active site accessibility**

  To directly address your concern, we have performed cross-sectional SEM imaging combined with EDS elemental mapping on post-electrolysis C0 and C18 cathodes. The results are presented in new **Supplementary Figs. 4 and 42**. The cross-sectional SEM-EDS analysis reveals that on both C0 and C18 cathodes, calcium scale is predominantly deposited on the electrode surface, with no fundamental difference in the spatial location of scale formation between the two electrodes. Furthermore, the XRD, FTIR and SEM characterization of post-reaction C0 and C18 cathodes in the manuscript (**Supplementary Figs. 3** and **38-40**) indicates that the scale morphology on both cathodes is dominated by amorphous $CaCO_3$, calcite and vaterite, with no significant difference.

  To investigate the effect of surface scaling on active site accessibility, we also conducted electrochemical active surface area (ECSA) measurements in our original manuscript. The results show that all scaled cathodes exhibit higher ECSA than their fresh counterparts (**Supplementary Figs. 21** and **50**), which is attributed to the scaling-induced enhancement of cathode hydrophilicity, thereby expanding the effective contact area between the cathode and the electrolyte. These results reveal a paradoxical scaling effect that calcium deposits physically block some original active surface sites and simultaneously create new electrochemically accessible surfaces by facilitating the electrolyte penetration. Furthermore, our results also reveal that calcium scale formation significantly improved the hydrophilicity of the cathodes, with the surface of the 0.4A-scaled cathodes becoming completely wetted (**Supplementary Figs. 19** and **49**). This enhanced hydrophilicity can aggravate water flooding (**Supplementary Figs. 20** and **51**), thereby inhibiting oxygen transport and ultimately leading to decreased H_2O_2 electroproduction.

The Revised Content in Manuscript

- **New discussion in Section "2e⁻ ORR performance and scale formation of surfactant-modified cathodes in Ca^{2+}-containing electrolytes." [Line 374-383, Page 11 (Revised)]**

Furthermore, the cross-sectional SEM-EDS analysis revealed that calcium scale was predominantly deposited on the surface of both C0 and C18 cathodes, with no fundamental difference in the spatial location of scale formation between the two electrodes (**Supplementary Figs. 4** and **42**). These characterization results provide evidence of the high similarity in calcium scale morphology and location between C0 and C18 cathodes. Combined with the anomalous difference in scaling quantity, this further suggests that the superior Ca^{2+} tolerance of C18 does not stem from a reduction in scaling amount or alterations in scale morphology and location. These findings prompt further investigations into the underlying mechanism of the high Ca^{2+} tolerance exhibited by C18.

- **New Figures in Supplementary Information:**

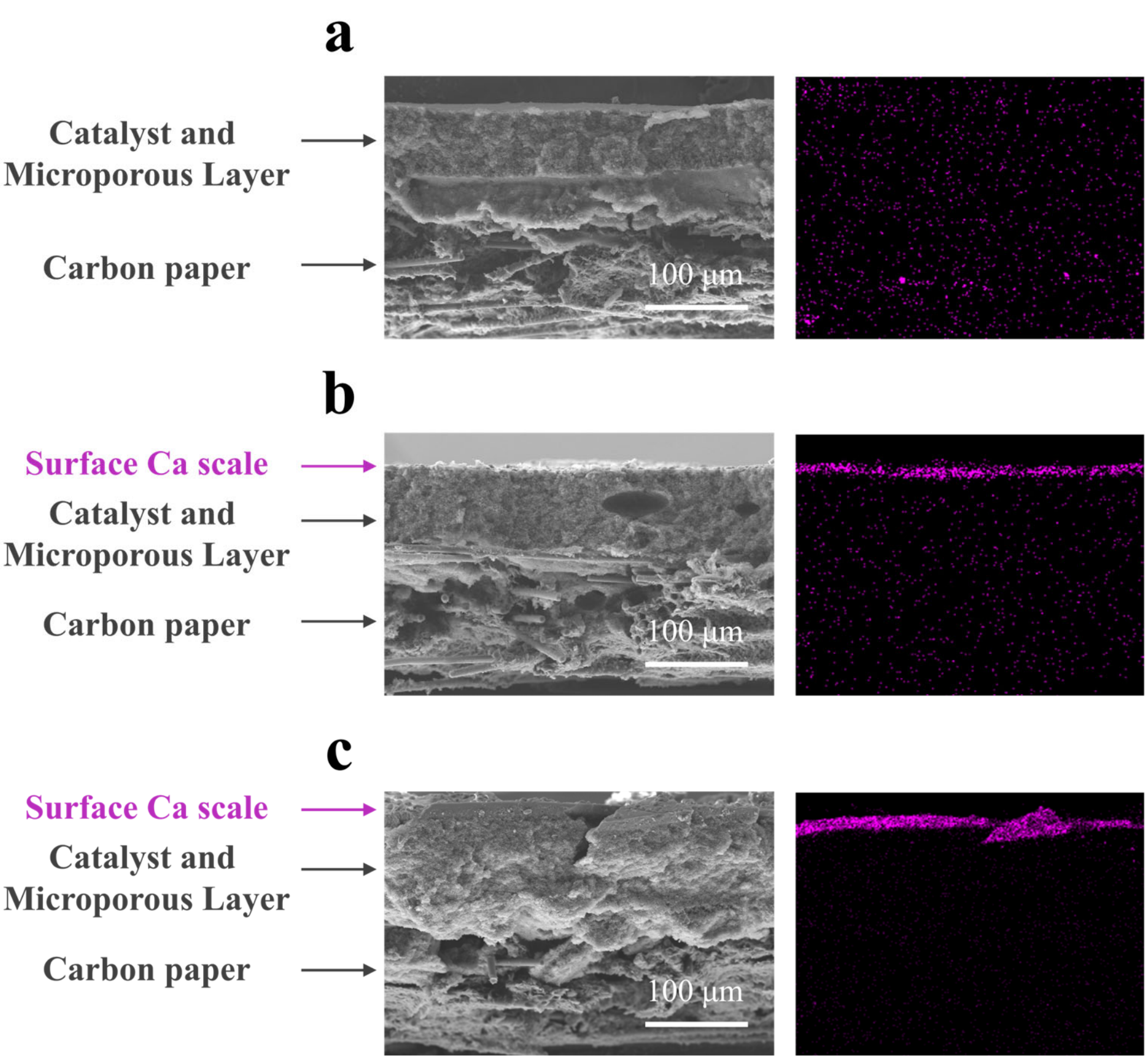

Supplementary Fig. 4. SEM images of cross-sectional view and EDS maps of Ca element for the C0 cathode (a) before experiment, (b) after experiment at 0.1A and (c) after experiment at 0.4A. Reaction conditions: $[Na_2SO_4]$ = 0.05 M, $[Ca^{2+}]_0$ = 200 mg/L, a rotation speed of 600 rpm, an initial pH of 7 and electrolysis of 3 h.

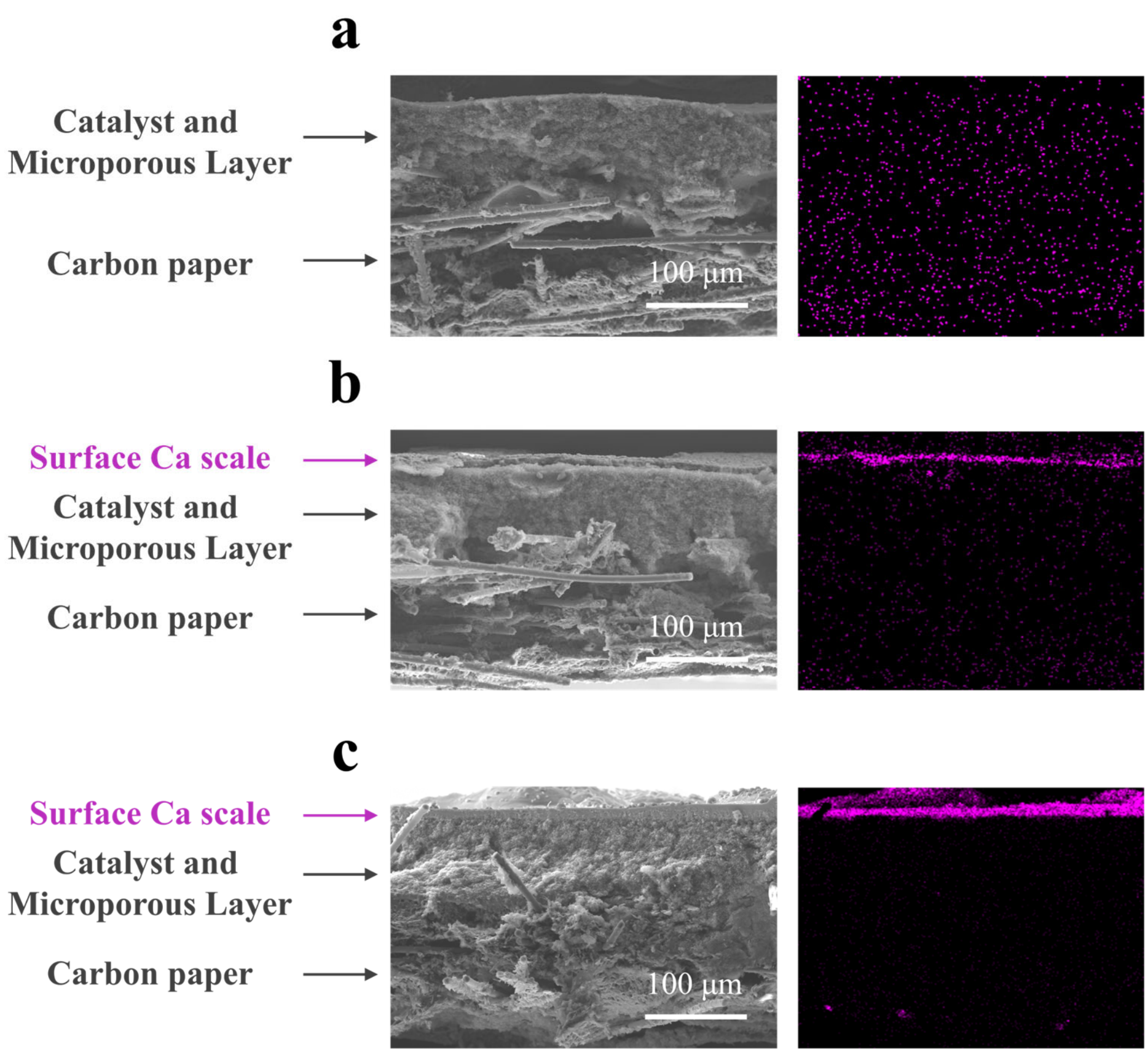

Supplementary Fig. 42. SEM images of cross-sectional view and EDS maps of Ca element for the C18 cathode (a) before experiment, (b) after experiment at 0.1A and (c) after experiment at 0.4A. Reaction conditions: $[Na_2SO_4]$ = 0.05 M, $[Ca^{2+}]_0$ = 200 mg/L, a rotation speed of 600 rpm, an initial pH of 7 and electrolysis of 3 h.

2. Multiple mechanisms are invoked without being disentangled or quantified. Authors attribute the improved selectivity to a combination of multiple factors, including (i) exclusion of Ca^{2+} from a more positive surface potential, (ii) disruption of hydrogen bond networks and limited proton transfers via hydrophobicity, (iii) formation of a highly alkaline microenvironment from the PUS confinement, and (iv) suppression of side reactions like H_2O_2 reduction and decomposition from protective roles of scales. All these effects can compete and overlap with each other, and authors should perform an ablation study to elucidate which factor is more deterministic. To "make up" some examples, dissolved Ca^{2+} may suppress the synthesis of H_2O_2 by accelerating O−O dissociation, and C18 may play an opposite role by slowing proton transfers. Without even a simplest rate and transport analysis, the conclusions read as a list of narratives rather than testable mechanisms.

Response:

Thank you for this constructive suggestion regarding our mechanism interpretation. We fully accept this suggestion and have conducted a systematic ablation study to address it. We are grateful for the opportunity to substantially strengthen the mechanistic interpretation of our work.

- **(1) Ablation study for C18: systematic decoupling of electrostatic effect and hydrophobic effect**

  To decouple these two effects, we designed control experiments using an additional material:

  (a) N-C18 (non-ionic long-chain surfactant-modified C0) preparation and characterization: Modified with 1-Octadecanethiol (ODT). The physical characterization of N-C18 is presented in new **Supplementary Fig. 58**. This material is hydrophobic (contact angle 134.02°, comparable to C18) but carries no positive charge (zeta potential -23.05 mV, comparable to C0), thereby isolating the hydrophobic effect.

  (b) Performance comparison among C0, N-C18, and C18 in the presence of 200 mg/L Ca^{2+} at 0.1 A: The FE of N-C18 was 15.60%, representing a 29.95% enhancement relative to C0 (12.00%). However, the FE of C18 reached 32.22%, corresponding to a 168.66% increase over C0 (12.00%). These results demonstrate that hydrophobic inhibition of proton availability (N-C18 vs. C0) contributes approximately 17.8%, providing a significant but secondary benefit. Electrostatic Ca^{2+} repulsion contributes approximately 82.2% of the total C18 enhancement, confirming it as the dominant mechanism.

  We have therefore refined our mechanistic attribution: the C18 performance enhancement is driven primarily by electrostatic Ca^{2+} repulsion (~82.2%), with hydrophobic inhibition of proton availability providing a supplementary contribution (~17.8%). Both contributions are now explicitly quantified and discussed.

- **(2) Isolating 3D-PUS alkaline confinement from Ca^{2+}-specific management effect**

  We have conducted additional control experiments to comprehensively evaluate the H_2O_2 electrosynthesis performance of bare C0 and C0-PUS cathodes in Ca^{2+}-free and Ca^{2+}-containing electrolyte. The new experimental results are presented in the revised Supplementary Information (**Supplementary Fig. 62**). The key findings are as follows:

(a) The 3D-PUS strategy does not enhance 2e$^-$ ORR selectivity in the absence of Ca^{2+}.

In Ca^{2+}-free electrolyte (0.05 M Na_2SO_4) at 0.1A, the H_2O_2 electrosynthesis performance of the C0-PUS cathode was comparable to that of the bare C0 cathode (597.68 versus 611.08 mmol $g_{cat.}^{-1}$ h^{-1}). The C18-PUS configuration demonstrated similar behavior. These results demonstrate that the pore confinement-induced alkaline microenvironment alone does not improve the 2e$^-$ ORR selectivity under our experimental conditions in the absence of Ca^{2+}. Despite the enhanced local alkalinity favors the 2e$^-$ pathway, the introduction of PUS concomitantly increased the mass transfer resistance for H_2O_2, which may account for the lack of improvement in its apparent yield.

(b) The 3D-PUS strategy significantly enhances 2e$^-$ ORR selectivity in the presence of Ca^{2+}.

In contrast, the PUS strategy delivers a dramatic enhancement only in Ca^{2+}-containing electrolyte.

For C0 at 0.1A: The C0-PUS cathode achieved a 293.3% enhancement in H_2O_2 yield over the bare C0 (550.26 versus 139.96 mmol $g_{cat.}^{-1}$ h^{-1}).

For C18 at 0.1A: The C18-PUS cathode exhibited a significantly higher H_2O_2 yield of 611.88 mmol $g_{cat.}^{-1}$ h^{-1}, which is 1.63 times that of bare C18 (376.04 mmol $g_{cat.}^{-1}$ h^{-1}).

The striking divergence between the Ca^{2+}-free and Ca^{2+}-containing results directly demonstrates that the PUS-induced enhancement is predominantly Ca^{2+}-specific: the pore confinement restricts OH^- diffusion, creating a localized alkaline microenvironment that reduces the interfacial concentration of dissolved Ca^{2+} and mitigates its detrimental catalytic effect on H_2O_2 reduction. We have revised the Discussion to clearly distinguish the Ca^{2+}-specific enhancement from any general alkaline effect of the PUS-fenced cathode architecture.

- **(3) The protective role of calcium scale in inhibiting H_2O_2 decomposition**

  We do not ascribe the enhanced H_2O_2 electrosynthesis performance to the suppression of H_2O_2 decomposition by calcium scale, because scale formation plays a dual role, exerting both detrimental and protective effects. Notably, in the absence of Ca^{2+}, the net effect of scale formation remains unfavorable. Therefore, the apparent paradox of scale-enhanced performance in Ca^{2+}-containing systems fundamentally arises from changes in dissolved Ca^{2+} concentration. Since the decrease in interfacial dissolved Ca^{2+} concentration and the formation of surface scale occur concurrently, greater scale deposition is inevitably accompanied by a lower local concentration of dissolved Ca^{2+} at the electrode-electrolyte interface. Accordingly, the improved H_2O_2 electrosynthesis performance should be intrinsically attributed to the reduced concentration of dissolved Ca^{2+} at the interface, rather than to a direct beneficial effect of the scale itself.

- **(4) Summary of revisions**

  In summary, this work focuses on the mechanistic role of dissolved Ca^{2+} in the 2e$^-$ ORR. Dissolved Ca^{2+} was identified as the primary inhibitor of the 2e$^-$ ORR, rather than precipitated calcium scale. Guided by this mechanistic insight, two strategies were developed to regulate interfacial Ca^{2+} distribution, thereby improving 2e$^-$ ORR selectivity. Moreover, the supplementary ablation experiments confirmed that reducing

the concentration of dissolved Ca^{2+} at the cathode-electrolyte interface is the decisive factor responsible for the enhanced H_2O_2 electroproduction.

The Revised Content in Manuscript

Based on your comments, we have conducted a systematic ablation study to disentangle the multiple effects on Ca^{2+}-tolerance. The following specific revisions have been implemented in the manuscript and supporting information:

- **Replaced Discussion in Section "Mechanistic study for enhanced Ca^{2+}-tolerance of surfactant-modified cathodes." [Line 492-504, Page 15 (Revised)]**

  Furthermore, to decouple the electrostatic and hydrophobic contributions of the C18 modification, a non-ionic long-chain surfactant-modified cathode (named N-C18) was prepared using 1-octadecanethiol (ODT). XRD, Raman and XPS spectra confirmed the successful modification of N-C18 (**Supplementary Fig. 58**). N-C18 exhibited a contact angle of 134.02° (comparable to C18 at 132.67°) while retaining a zeta potential of -23.05 mV (comparable to C0 at -25.26 mV) (**Supplementary Fig. 58**), thereby isolating the hydrophobic effect from electrostatic repulsion. Compared with C0, N-C18 exhibited a 29.95% improvement in performance in Ca^{2+}-containing electrolyte, whereas C18 achieved a 168.66% enhancement (**Supplementary Fig. 59**). These results indicate that the hydrophobicity-driven mass transport plays a secondary role in enhancing Ca^{2+}-tolerance (~17.8% contribution), whereas the electrostatic effect constitutes the dominant mechanism (~82.2% contribution), which mitigates the adverse effects of dissolved Ca^{2+} on H_2O_2 electroproduction.
- **New Discussion in Section "Enhanced Ca^{2+}-tolerance by a fenced cathode structure." [Line 532-536, Page 17 (Revised)]**

  Notably, the FEs of C0-PUS and C18-PUS cathodes were comparable to that of the bare counterpart in Ca^{2+}-free electrolyte (**Supplementary Fig. 62**). The striking divergence between the Ca^{2+}-free and Ca^{2+}-containing results directly demonstrates that the PUS-induced enhancement is predominantly Ca^{2+}-specific rather than any general alkaline effect.
- **New Figures in Supporting Information:**

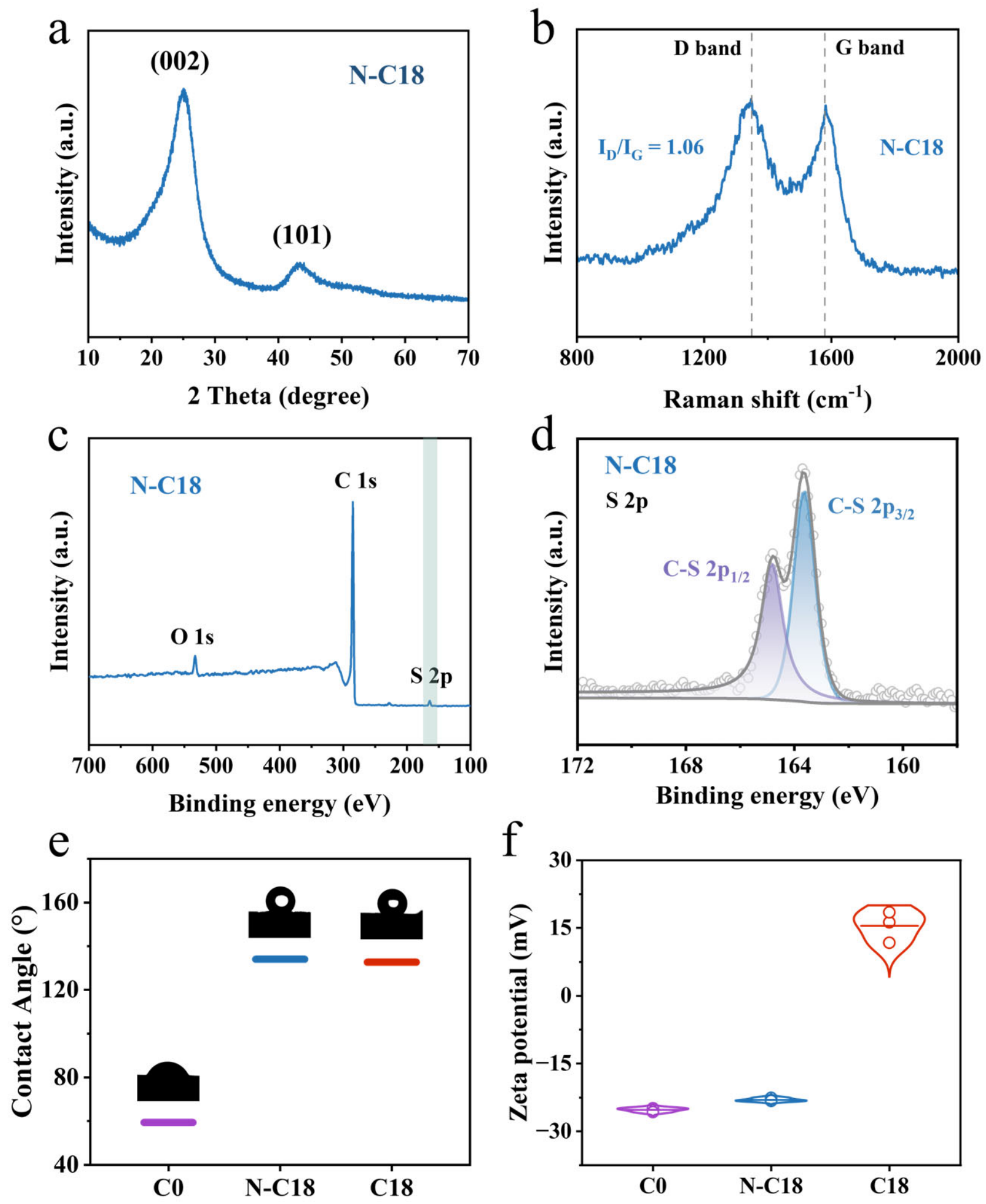

Supplementary Fig. 58 (a) XRD pattern and (b) Raman spectra of the N-C18 catalyst. (c) XPS spectra and (d) high-resolution S 2p spectra of N-C18. (e) Contact angle and (f) Zeta potential measurements of C0, N-C18, and C18.

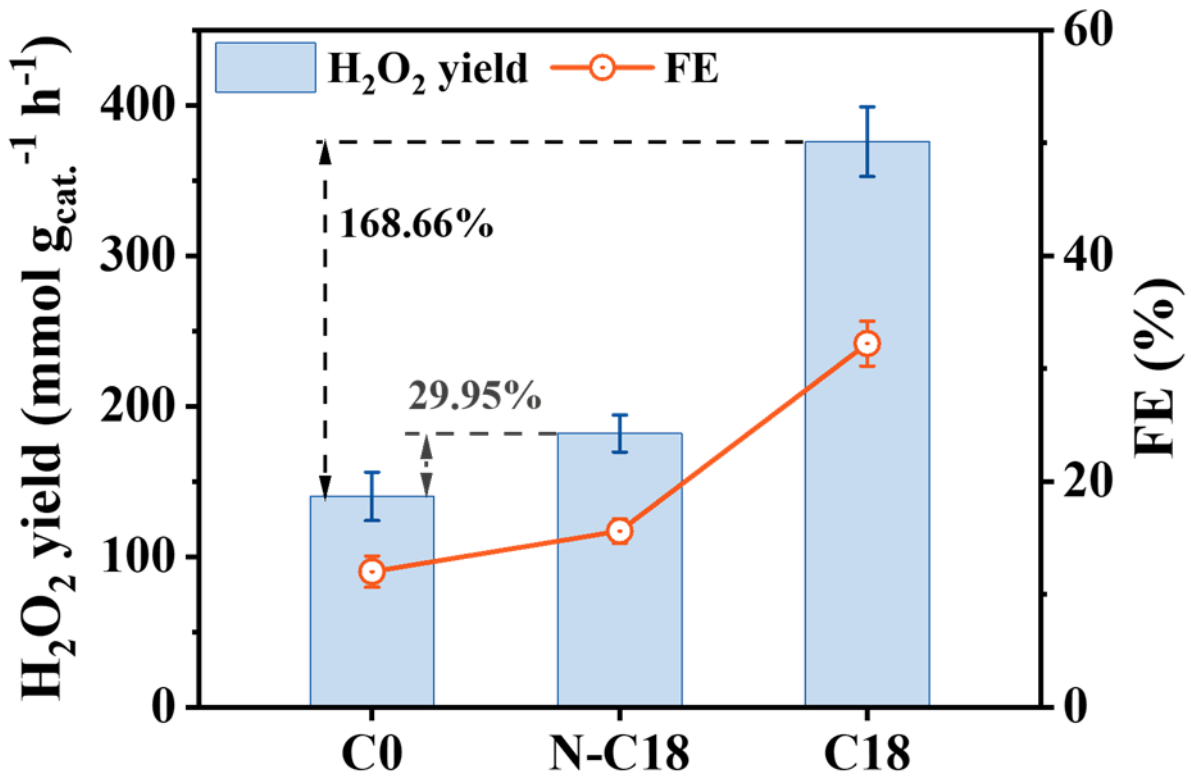

Supplementary Fig. 59 H_2O_2 electrosynthesis performance of the C0, N-C18, and C18 cathodes in the presence of Ca^{2+}. Reaction conditions: $[Na_2SO_4]$ = 0.05 M, $[Ca^{2+}]_0$ = 200 mg/L, a current of 0.1 A, a rotation speed of 600 rpm, an initial pH of 7 and electrolysis of 3 h.

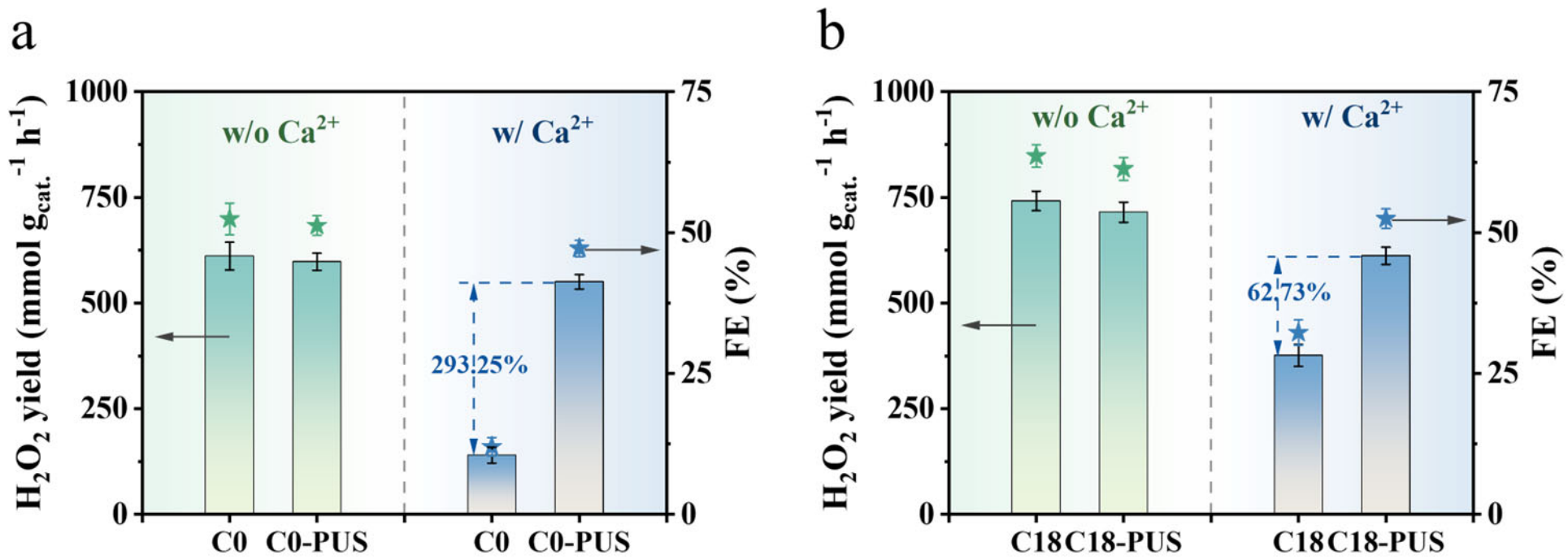

Supplementary Fig. 62 (a) H_2O_2 electrosynthesis performance of the C0 and C0-PUS cathodes in the absence (w/o) or presence (w/) of 200 mg/L Ca^{2+}. (a) H_2O_2 electrosynthesis performance of the C18 and C18-PUS cathodes in the absence (w/o) or presence (w/) of 200 mg/L Ca^{2+}. Reaction conditions: $[Na_2SO_4]$ = 0.05 M, a current of 0.1 A, a rotation speed of 600 rpm, an initial pH of 7 and electrolysis of 3 h.

- **Updated Methods Section**

Chemicals and materials [Line 637-641, Page 20 (Revised)]

Potassium titanium oxalate ($C_4H_2K_2O_{10}Ti$, purity > 99%), 1-Octadecanethiol (ODT), Octyltrimethylammonium bromide (OTAB), decyltrimethylammonium bromide (DTAB), tetradecyltrimethylammonium bromide (TTAB), and stearyltrimethylammonium bromide (STAB) were purchased from Shanghai Macklin Biotechnology Co., Ltd.

Synthesis of surfactants modified oxygen-doped carbon black [Line 662-667, Page 21 (Revised)]

Synthesis of ODT-OCB (named N-C18, non-ionic long-chain surfactant-modified C0). To prepare the non-ionic long-chain surfactant-modified OCB catalyst, 60 mg of OCB and 240 μmol of ODT were added into 20 mL of anhydrous ethanol, followed by sonication for 1 h and stirring for 24 h to obtain a homogeneous suspension. The final samples were collected through vacuum filtration followed by washing with plenty anhydrous ethanol, and were subsequently vacuum-dried for 24 h at 60°C.

3. Electrostatic interpretation based on PZC/zeta potential is oversimplified. Authors link the exclusion of Ca^{2+} to a more positive surface potential of C18. However, PZC and zeta potential measured under non-operando conditions cannot be directly mapped onto the interfacial charge and potential. Authors should discuss the relationship between PZC, electrode potential, specific adsorption, and double-layer structure. Moreover, authors suggest enhanced interfacial cation density and claim electrostatic repulsion of Ca^{2+}, which appears contradictory unless clarified.

Response:

Thank you for this insightful critique regarding our electrostatic interpretation based on zeta potential or PZC measurements. We fully accept these criticisms and address each point below.

- **(1) Correction of the PZC value**

  We first wish to correct an error in our original manuscript. The PZC of C0 was originally reported without conversion to the RHE scale. After proper conversion, the PZC of C0 is 0.80 V vs. RHE. The PZC of C18 (0.35 V vs. RHE) is correct. We have updated these values throughout the revised manuscript and apologize for this oversight.

- **(2) Discussion of the relationship between PZC, electrode potential, specific adsorption, and double-layer structure**

  We fully agree that PZC and zeta potential measured under equilibrium, non-polarized conditions cannot be directly mapped onto the interfacial charge and potential under cathodic polarization. In response to your suggestion, we have added a detailed discussion by explicitly considering the relationship among the potential of zero charge (PZC), applied electrode potential, specific ion adsorption, and electrical double-layer (EDL) structure in the revised manuscript.

  In this work, the PZC is used as a reference potential to evaluate the tendency of surface charge accumulation, rather than as a direct measure of the operando surface potential. The PZC values of C0 and C18 were determined to be 0.80 and 0.35 V vs. RHE, respectively. Under cathodic polarization, the applied electrode potential is typically more negative than the PZC of both electrodes, leading to the formation of negative excess surface charge. In principle, the excess surface charge is governed by the deviation of the applied potential from the PZC (i.e., $E-E_{PZC}$), together with the differential double-layer capacitance. Therefore, at the same cathodic potential, C0 with a more positive PZC would exhibit a larger negative deviation from its PZC and thus a stronger electrostatic tendency to attract Ca^{2+} toward the cathode interface. In contrast, the more negative PZC of C18 reduces the negative deviation from PZC under identical cathodic polarization, thereby weakening the purely electrostatic driving force for Ca^{2+} accumulation.

  More importantly, Ca^{2+} distribution near the cathode is not determined solely by the net surface charge. The STAB modification on C18 substantially alters the EDL structure, especially the Stern layer adjacent to the carbon surface. The long hydrophobic alkyl chains and interfacial organic layer can reduce the accessibility of Ca^{2+} to the inner Helmholtz plane and suppress specific Ca^{2+} adsorption and interfacial enrichment by steric effects. As a result, C18 regulates Ca^{2+} behavior through a combined mechanism involving electrostatic repulsion and steric exclusion effects within the EDL.

- **(3) Clarification of the apparent contradiction**
  We sincerely apologize for the confusing and seemingly contradictory statement regarding the enhanced interfacial cation density and electrostatic repulsion of Ca^{2+} in our original manuscript. In response, we clarify our intended meaning as follows:
  The original statement was imprecisely worded. What we intended to convey is that the C18 surface, due to the presence of quaternary ammonium cations ($-N^{+}(CH_3)_3$) from STAB modification, possesses a higher density of positive surface charge compared to C0, which enabling the electrostatic repulsion of dissolved Ca^{2+} from the interface. In other words, the "enhanced cation density" refers to the surface-bound quaternary ammonium groups ($-N^{+}(CH_3)_3$) on C18, not to an increased concentration of Ca^{2+} at the interface. We have thoroughly revised the relevant text to eliminate this ambiguity.

The Revised Content in Manuscript

Based on your comments, we have comprehensively discussed the relationship between PZC, electrode potential, specific adsorption, and double-layer structure, and have thoroughly clarified the apparent contradiction to eliminate any ambiguity. The following specific revisions have been implemented in the revised manuscript:

- **Replaced Figure and Analysis in Section "Mechanistic study for enhanced Ca^{2+}-tolerance of surfactant-modified cathodes." [Line 448-460, Page 14 (Revised)]**
  To investigate the differences in interfacial properties between C0 and C18, the potential of zero charge (PZC) was determined by the differential capacitance method.[43] Compared with C0 (0.80 V vs. RHE), C18 exhibited a pronounced negative shift in PZC (0.35 V vs. RHE), attributable to the modification with positively charged quaternary ammonium groups ($-N^{+}(CH_3)_3$) on its surface (**Fig. 4c**)[50]. Under cathodic polarization (typically $E < E_{PZC}$ for both cathodes), the excess negative surface charge is governed by the deviation from PZC ($E-E_{PZC}$)[51]. Therefore, the more negative PZC of C18 can reduce the negative deviation from PZC under identical cathodic polarization, thereby exhibiting less negative charge and weakening the purely electrostatic driving force for Ca^{2+} accumulation. Beyond net surface charge, the STAB modification can substantially alter the electrical double-layer (EDL) structure. The hydrophobic alkyl chains and interfacial organic layer can reduce Ca^{2+} accessibility to the inner Helmholtz plane and suppress specific Ca^{2+} adsorption through steric effects.

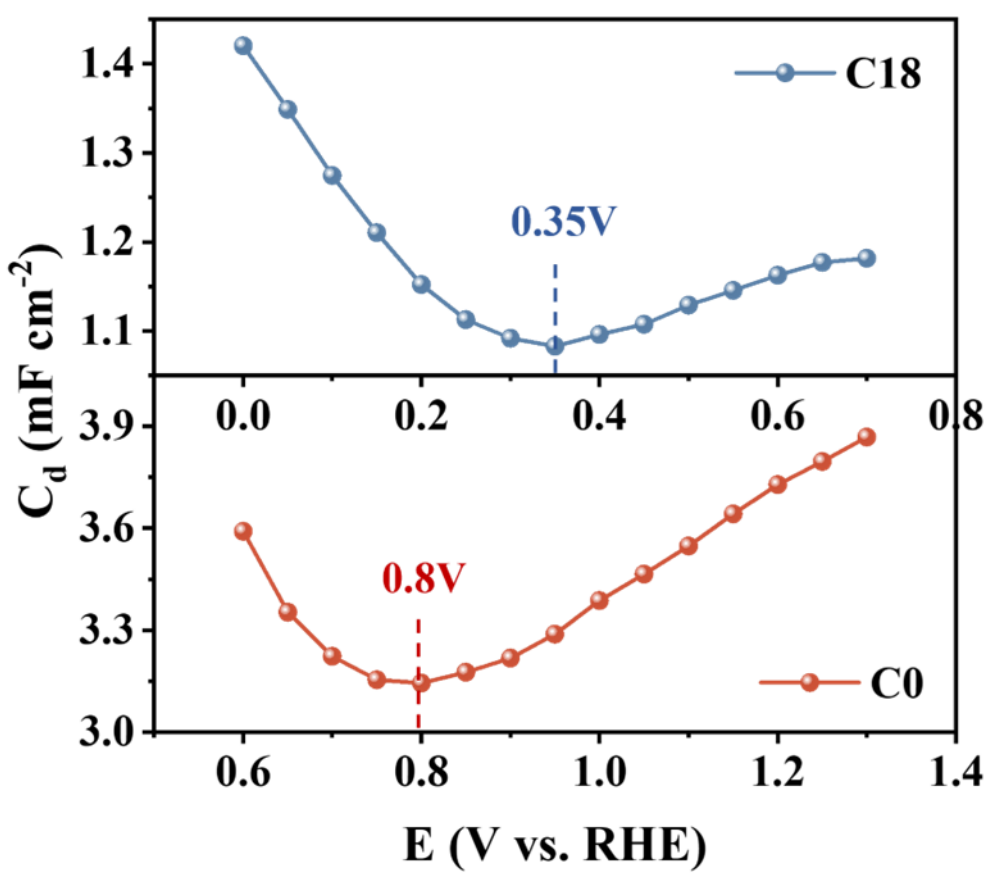

Fig. 4. (c) Comparison of PZC of C0 and C18.

- **New reference**

50. Wu, T. *et al.* Surface-treated carbon electrodes with modified potential of zero charge for capacitive deionization. *Water Research* **93**, 30-37 (2016).
51. Duan, S. *et al.* Potential of zero charge regulating highly selective removal of nitrate anions through capacitive deionization. *Chemical Engineering Journal* **442**, 136287 (2022).

4. Claims about highly alkaline interfacial microenvironment rely on questionable proxies. Authors argue PUS-fenced electrode to restrict OH^- diffusion and therefore create a localized alkaline microenvironment that suppresses the Ca^{2+} activity, but they bulk pH values taken ~1 cm from the electrode, which is not a reliable proxy. Authors should either (i) provide more appropriate interfacial pH diagnostics (e.g., microelectrode profiling at the surface, spectroscopic probes, or modeling constrained by measured currents), or (ii) substantially soften this claim and reframe it as a meso-scale transport effect.

Response:

Thank you for this important question regarding our pH measurement methodology. We acknowledge that bulk pH values measured at ~1 cm from the cathode surface cannot be considered a reliable proxy for the interfacial pH, and that our claim of a "highly alkaline interfacial microenvironment" requires more direct evidence. We fully accept this suggestion and have taken the following actions to address it.

Response:

Due to the lack of operando interfacial pH diagnostics, such as surface-positioned pH microelectrode profiling or spectroscopic pH probes, we have substantially softened the original claim to accurately reflect the strength of our evidence. In the revised manuscript, we no longer state that the PUS-fenced electrode definitively creates a “highly alkaline interfacial microenvironment” on the cathode surface. Instead, we reframe the effect as a meso-scale transport phenomenon. Specifically, the PUS-fenced structure is described as a confined barrier that partially restricting electrolyte exchange and outward diffusion of electrochemically generated OH^-, thereby favoring OH^- retention and an elevated alkalinity within the confined near-cathode region. This elevated alkalinity drives $CaCO_3$ precipitation, thereby reducing the concentration of dissolved Ca^{2+} in the near-cathode region and subsequently promoting the $2e^-$ ORR selectivity.

The Revised Content in Manuscript

Based on your comments, we have revised the manuscript to substantially soften the original claim and accurately reflect the strength of our evidence. The following specific revisions have been implemented in the manuscript:

- **Replaced discussion in Section “Enhanced Ca^{2+}-tolerance by a fenced cathode structure.”**

The pH of the bulk solution at 1 cm from the cathode was measured to illustrate the regulatory effect of PUS on alkalinity near the cathode region. The results indicated that a smaller pore size leads to a more pronounced alkaline environment. The bulk pH followed the order: No-PUS > 20 PPI > 30 PPI > 40 PPI > 60 PPI (**Fig. 5h**). Given the theoretically consistent OH^- generation across cathodes, this trend directly confirms that the pore confinement effect of PUS restricts OH^- diffusion, thereby enhancing the alkaline environment. Visualization experiments employing thymolphthalein as the pH indicator further corroborated this finding (**Fig. S74**)[53]. Collectively, the 3D foam-fenced cathode system utilizes the pore confinement effect of the PUS to substantially elevate the OH^- concentration, creating a highly alkaline environment within the

confined near-cathode region. The elevated alkalinity promotes the 2e$^-$ ORR pathway by reducing proton availability while simultaneously inducing calcium precipitation, thereby decreasing interfacial dissolved Ca^{2+} concentration and mitigating its adverse effects on the 2e$^-$ ORR selectivity. **[Line 555-568, Page 17 (Revised)]**

Taken together, the synergistic combination of electrostatic potential modulation and PUS confinement substantially elevates the OH^- concentration in the near-cathode region. This highly alkaline environment mitigates the adverse cationic effects of Ca^{2+} and effectively suppresses the transition from the 2e$^-$ ORR to the 4e$^-$ ORR pathway, thereby enhancing H_2O_2 electrosynthesis performance in Ca^{2+}-containing systems. **[Line 584-589, Page 18 (Revised)]**

- **Replaced statement in Section "Discussion"**

  Moreover, the pore confinement effect of the 3D-PUS restricts OH^- diffusion and creates a localized alkaline environment within the confined near-cathode region that further inhibits Ca^{2+} activity, thereby enhancing the 2e$^-$ ORR selectivity in Ca^{2+}-containing environments. **[Line 621-624, Page 20 (Revised)]**

5. DFT and MD modeling do not fully support the mechanistic claims. Reported MD simulations (20 ns NVT, GAFF/RESP, explicit electrolyte) provide structural information but do not establish proton transfer kinetics. Therefore, the claim about slower proton transfer kinetics from reduced hydrogen bond or water density is not directly grounded. Charge and potential of the electrode are not physically implemented and the claim about the repulsion of Ca^{2+} is not straightforward either. For DFT calculations, while the computational workflow is standard (VASP PBE/D3, NEB), the treatment for Ca^{2+} adsorption and energetics is incomplete without being fully solvated by water molecules.

Response:

Thank you for this insightful comment of our computational methodology. We fully agree the three specific concerns and address each point below.

- **(1) Clarification of MD simulation scope: structural but not kinetic information**

  We fully agree that our MD simulations (20 ns NVT ensemble, GAFF/RESP, explicit electrolyte) provide equilibrium structural information—specifically, radial distribution functions, density profiles, and hydrogen bond statistics—rather than direct proton transfer kinetics. We acknowledge that in our original manuscript, the connection between the structural MD results and the claim of "impedes proton transfer kinetics" was insufficiently qualified. In response, we have revised the manuscript to clearly delineate what the MD simulations do and do not establish:

  What MD establishes: The hydrophobic alkyl chains of STAB reduce the number of interfacial water molecules and disrupt the hydrogen-bond network near the C18 surface compared to C0 (**Fig. 4h** and **Supplementary Fig. 57**), resulting in a decrease in proton source and thereby suppressing the proton availability. This is a structural observation from MD simulations.

  Where the kinetic evidence comes from: The claim of slower proton transfer on C18 is directly supported by our experimental EIS data, which show a larger EIS-derived Tafel slope for C18 compared to C0 (**Figs. 1f** and **3i**), and by the H_2O_2 decomposition kinetics showing slower H_2O_2 reduction on C18 than that on C0 (**Fig. 3l** and **Supplementary Fig. 22**). The MD simulations provide the structural rationale for why proton availability is reduced, but the kinetic conclusion depends on the experimental measurements.

  We have added explicit qualifying language throughout the revised manuscript to ensure this distinction is clear: the MD simulations reveal the structural origin of reduced proton availability, while the EIS and decomposition experiments provide the kinetic confirmation.

- **(2) Clarification of electrode potential implementation in MD simulations**

  We apologize for the omission of critical methodological details in our original manuscript. In fact, an external electric field of 0.1 V nm^{-1} was physically applied perpendicular to the electrode surface (along the z-direction) in our original MD simulations. The applied electric field exerts a force on all charged species in the system: In our system, Ca^{2+} experience a force directing them toward the negatively polarized electrode surface. The resulting Ca^{2+} density profiles (**Fig. 4i**) reflect the balance between electromigration toward the surface and electrostatic repulsion from the

positively charged -$N^+(CH_3)_3$ groups on C18. Therefore, the key observation—that Ca^{2+} is both less concentrated and farther from the C18 surface (peak at ~11.91 Å, 8.27 mg cm^{-3}) compared to the C0 surface (peak at ~7.95 Å, 9.58 mg cm^{-3})—is obtained under the applied electric field that explicitly simulates the polarized condition, which directly supports the Ca^{2+} exclusion mechanism. We have added these key details to the revised Methods section.

- **(3) DFT for water dissociation and Ca^{2+} adsorption calculations with explicit water molecules and the solvation model**
  We acknowledge that our original DFT calculations for Ca^{2+} adsorption energies were computed for unsolvated Ca^{2+} in vacuum, which neglects the critical effects of the solvent. We fully accept this limitation and have performed new calculations with explicit water molecules and the solvation model. To ensure consistency and accuracy of the DFT calculations, the models for the water dissociation pathway calculations were also reconstructed using the same explicit water molecules and solvation model. It is worth noting that, in accordance with your first minor issue, the slab model has also been reconstructed, as will be elaborated in the response to the first minor issue.
  Specifically, we reconstructed solvated models by placing Na^+ or Ca^{2+} with explicit water molecules forming a water bilayer between the ion and the electrode surface. The solvation model in the DFT calculations was implemented using the VASPsol package. The energy barrier of the water dissociation and the adsorption energy was then recomputed. The core mechanistic conclusions from the reconstructed solvation model remain qualitatively consistent:
  (a) Ca^{2+} continues to lower the activation barrier for interfacial water dissociation on the reconstructed solvation model, demonstrating that dissolved Ca^{2+} can markedly promote the water dissociation process.
  (b) Ca^{2+} adsorption from the reconstructed solvation model remains thermodynamically less favorable on C18 (E_{ad} = -0.85 eV) than on C0 (E_{ad} = -1.34 eV), confirming that the electrostatic repulsion mechanism persists under solvated conditions.

The Revised Content in Manuscript

- **(1) Clearer statement of the conclusions and calculation parameters for MD simulations**
  Based on your comments, we have more clearly elucidated the conclusions derived from the MD simulations. The following specific revisions have been implemented in the manuscript:
- **Replaced Analysis in Section "Mechanistic study for enhanced Ca^{2+}-tolerance of surfactant-modified cathodes." [Line 477-480, Page 15 (Revised)]**
  Compared to C0, the C18 exhibited a decrease number of interfacial water molecules and hydrogen bonds (**Fig. 4h and Supplementary Fig. 57**), indicating that the hydrophobic chains of STAB can disrupt the interfacial water structure and suppress proton availability at the interface.
- **Updated Methods Section**
  Molecular dynamics (MD) simulation [Line 885-886, Page 26 (Revised)]

An external electric field of 0.1 V nm^{-1} was physically applied perpendicular to the electrode surface (along the z-direction).

- **(2) Reconstructed computational models with explicit water molecules and the solvation model**

Based on your comments, we have reconstructed the computational models with explicit water molecules and the solvation model. The following specific revisions have been implemented in the manuscript and supporting information:

- **Replaced Figures and Analysis in Section "Evidence for the suppression of $2e^-$ ORR by dissolved Ca^{2+}." [Line 195-211, Page 6 (Revised)]**

The original **Fig. 1g** has been replaced with the new energy profiles of water dissociation process (**new Fig. 1k**) based on the reconstructed models. The corresponding analysis in the text has also been revised as follows:

DFT calculations were further employed to elucidate the critical role of Ca^{2+} in promoting water dissociation. A monolayer graphene structure incorporating an ether group and an adjacent carbon vacancy defect was constructed as a representative model for DFT calculations (**Fig. 1j, Supplementary Figs. 16** and **17**), considering the experimental surface chemistry of C0 and the active site configuration associated with high $2e^-$ ORR selectivity in the literature[28]. The calculation results demonstrate that Ca^{2+} significantly lowers the activation energy barrier for water dissociation ($H_2O \rightarrow H^+ + OH^-$), markedly enhancing the dissociation process (**Fig. 1k**). To further elucidate the origin of Ca^{2+}-accelerated interfacial water dissociation, the role of interfacial electric field was examined. Charge transfer between the slab and electrolyte was quantified using Bader charge analysis, and the interfacial electric field strength was estimated based on a parallel-plate capacitor model. Under applied electric field, the H-OH bond of interfacial H_2O is polarized through $*H\text{-}OH^{\delta-}\text{-}M^+$ interactions, as established in previous fundamental studies[16]. The Ca^{2+} system exhibits a substantially enhanced electric field strength, accompanied by elongation of the H-OH bond, indicating that Ca^{2+} facilitates the water dissociation step by promoting interfacial H_2O polarization (**Supplementary Fig. 18**).

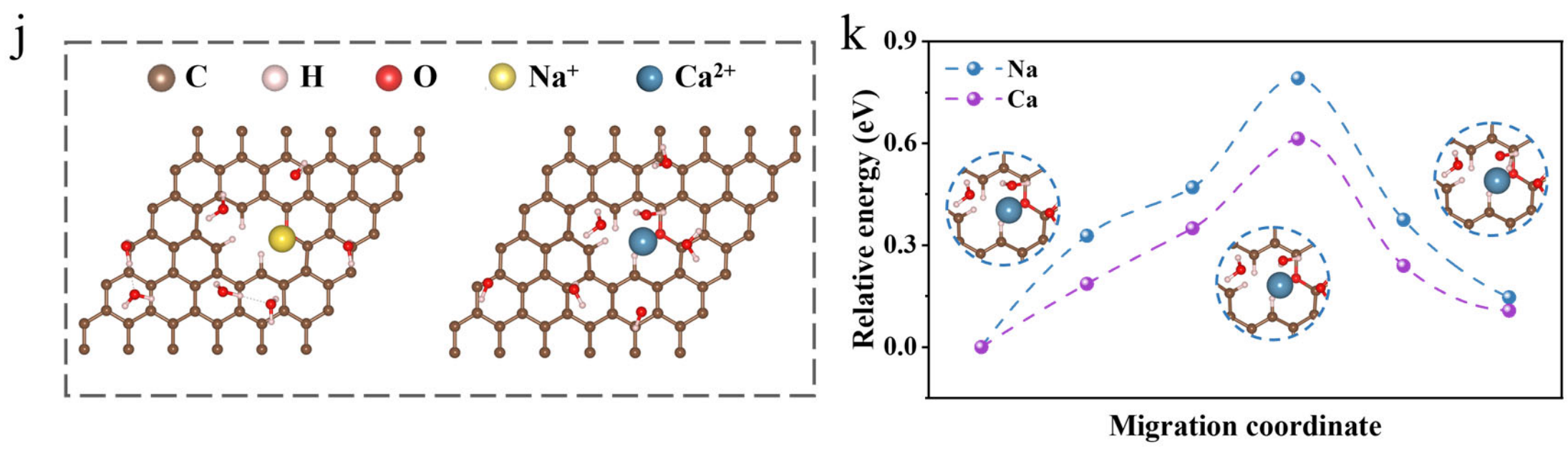

Fig. 1. Effects of dissolved Ca^{2+} on $2e^-$ ORR for oxygen-doped carbon black (named C0). (j) Theoretical models of the oxygen-doped graphene surface with Na^+ and Ca^{2+}. (k) Transition states and energy profiles of water dissociation process.

- **Replaced Figures and Analysis in Section "Mechanistic study for enhanced Ca^{2+}-tolerance of surfactant-modified cathodes." [Line 467-472, Page 15 (Revised)]**

The original **Fig. 4e and 4f** have been replaced with the new adsorption energy profiles (new **Fig. 4e and 4f**) based on the reconstructed models. The corresponding analysis in the text has also been revised as follows:

To elucidate the mechanism underlying the high Ca^{2+} tolerance of the C18 cathode, DFT calculations were employed to investigate the thermodynamic properties of the C0 and C18. The pristine graphene model and the one modified with STAB were used to represent C0 and C18, respectively (**Fig. 4e, 4f** and **Supplementary Fig. 54**). The adsorption energies (E_{ad}) of Ca^{2+} on C0 and C18 were -1.34 eV and -0.85 eV, respectively, demonstrating a weaker interaction between Ca^{2+} and C18.

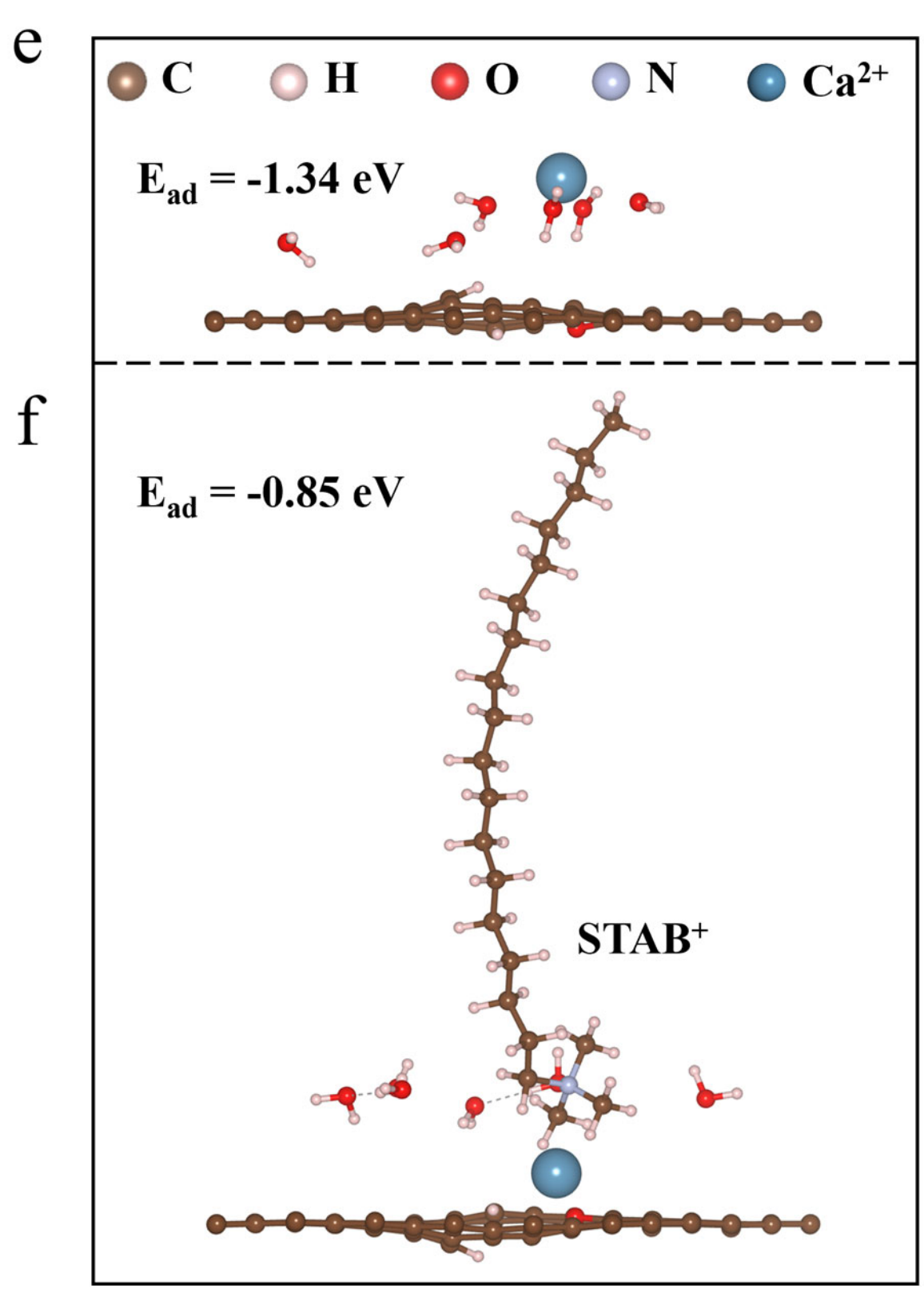

Fig. 4. The adsorption energies (E_{ad}) for Ca^{2+} on the surfaces of (e) C0 and (f) C18 cathodes obtained from DFT calculations.

- **Replaced Figures in Supplementary Information:**
  Supplementary Figs. 11 and 12 in the Supplementary Information have been replaced and are now renumbered as **Supplementary Figs. 16** and **17**.

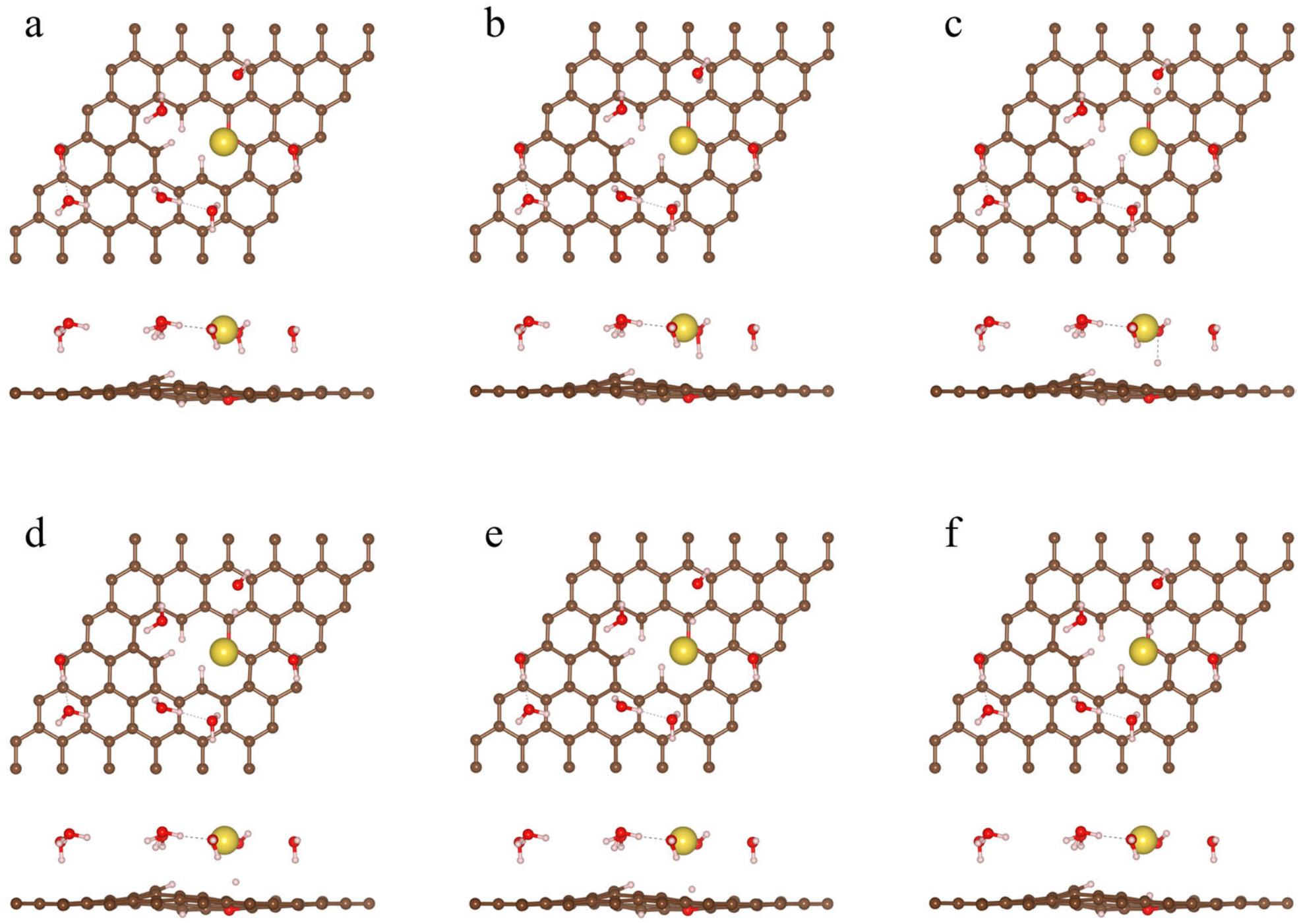

Supplementary Fig. 16. Atomic configurations of water dissociation step on the graphene surface with Na^+: (a) initial state, (b-e) transition state and (f) final state. The brown, red, pink, and yellow spheres represent C, O, H and Na atoms, respectively.

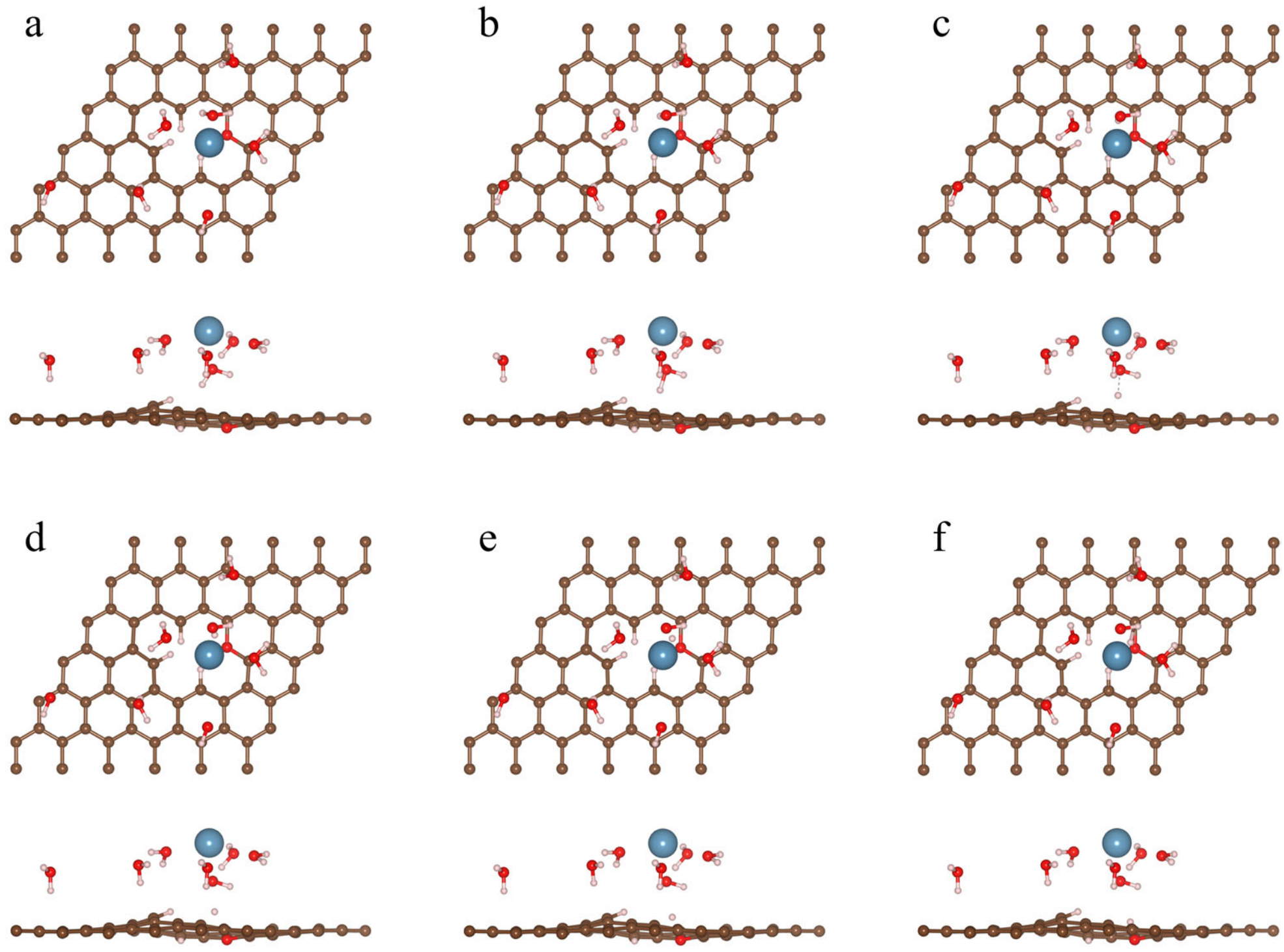

Supplementary Fig. 17. Atomic configurations of water dissociation step on the graphene surface with Ca^+: (a) initial state, (b-e) transition state and (f) final state. The brown, red, pink, and blue spheres represent C, O, H and Ca atoms, respectively.

- **New Figures in Supplementary Information:**

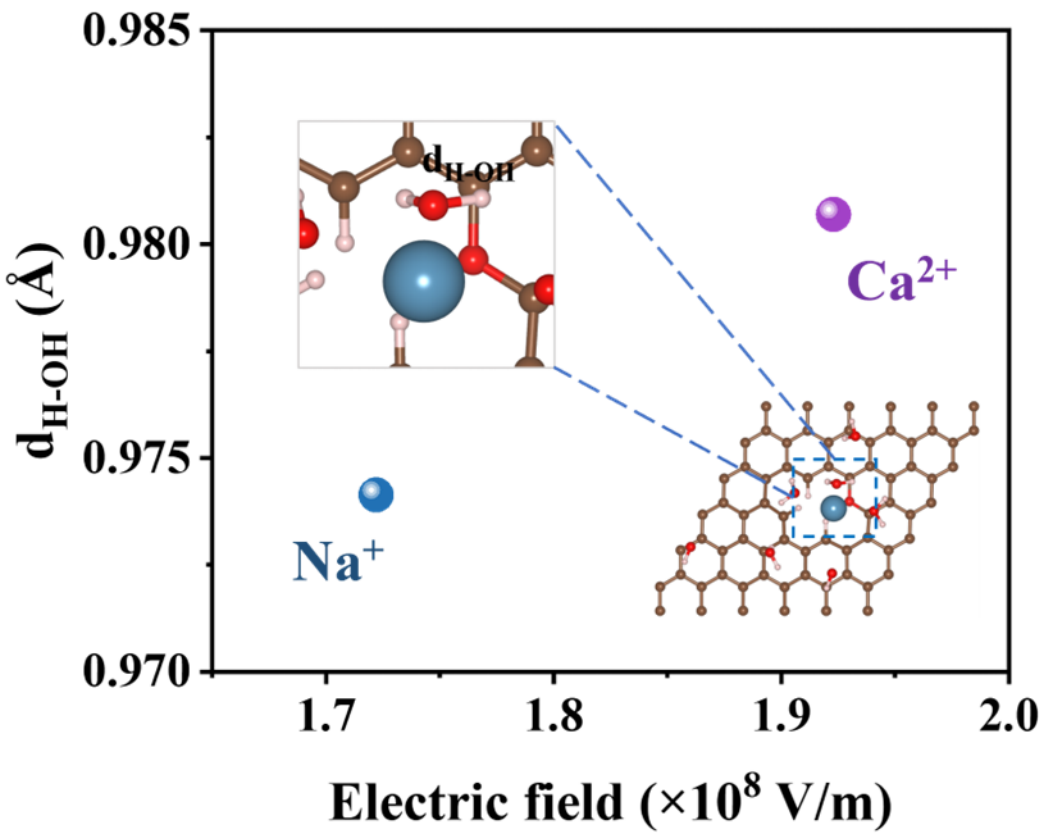

Supplementary Fig. 18. The measured bond length of H-OH bond in Na^+ and Ca^{2+} systems.

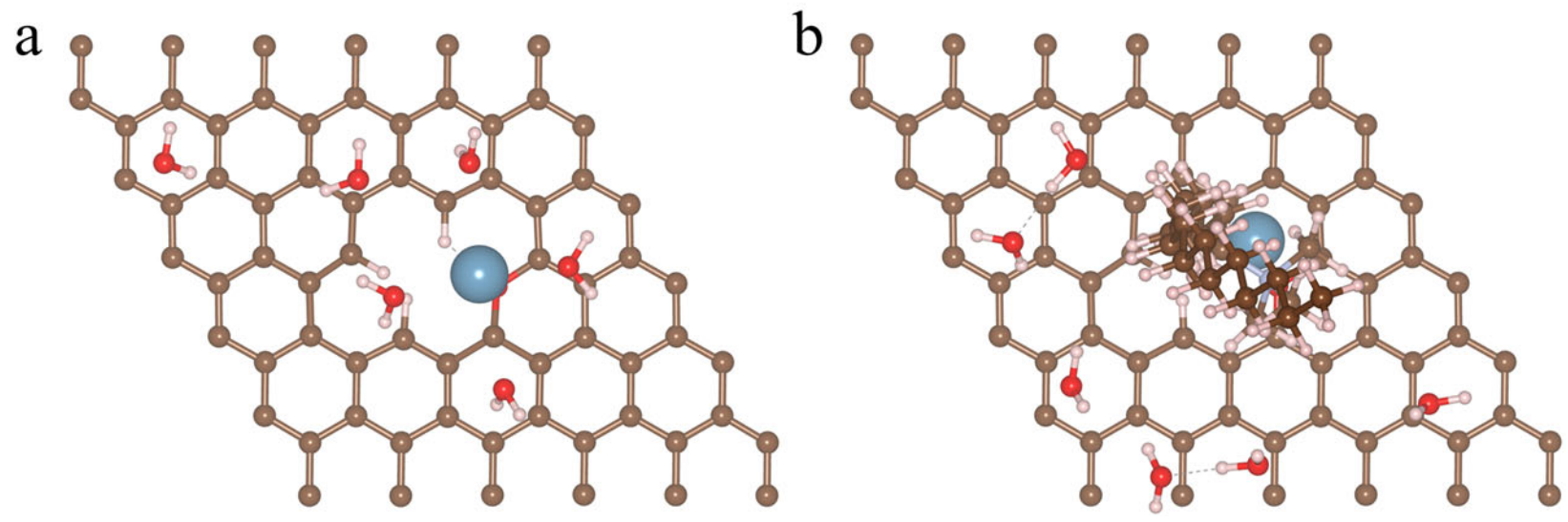

Supplementary Fig. 54. The adsorption planar models for Ca^{2+} on the surfaces of (a) C0 and (b) C18 cathodes. The brown, red, pink, and blue spheres represent C, O, H and Ca atoms, respectively.

- **Updated Methods Section**

Density functional theory (DFT) calculations [Line 862-874, Page 26 (Revised)]

A water bi-layer was included to describe explicitly the polarization involved in water dissociation. The implicit solvation effects were implemented using the VASPsol package.

The electric field strength at the interface between the graphene surface and the electrolyte was evaluated using a parallel-plate capacitor model[16]. The electric field strength (E) was calculated according to the following equation: $E = (Q_1+Q_2) / (2\varepsilon S)$, where Q_1 and Q_2 represent the net charges of the graphene slab and the electrolyte, respectively, as determined by Bader charge analysis; S represents the surface area of the graphene slab; and ε represents the absolute permittivity of water.

The adsorption energies of *OOH or Ca^{2+} on the surface of C0 or C18 were calculated by the formula: $E_{ad} = E_{total} - E_{base} - E_{adsorbate}$, where E_{total} is the total energy of the absorbed system, E_{base} is the basement energy and $E_{adsorbate}$ is the energy of adsorbate.

- **New reference**

28. Cao, P. *et al.* Cation-tuned acidic electrified interface for hydrogen peroxide electrosynthesis with industrial-level current densities in natural seawater. *Nature Communications* **17**, 5443 (2026).

Minor Issues:

1. Authors should clarify the rationale for selecting the specific active sites and models used in DFT, and whether alternative defects and functional group sites were tested.

Response:

Thank you for this constructive comment regarding the rationale of our DFT models selection. We appreciate the opportunity to clarify the basis for our model construction and to address whether alternative active site configurations were considered. In response, we have carefully re-examined our experimental characterization data, reviewed the relevant literature, and reconstructed our DFT models to better reflect the actual surface chemistry of C0 and C18. We have also performed additional calculations to verify the robustness of our mechanistic conclusions.

- **(1) Reconsidering the DFT models based on comprehensive experimental characterization and literature references**

  Our experimental characterization (Raman, FTIR and XPS) reveals that C0 possesses both intrinsic carbon defects and oxygen functional groups. Therefore, a realistic model of the C0 surface should account for both oxygen functional groups and carbon defect sites. Furthermore, a survey of recent literature on carbon-based $2e^-$ ORR catalysts indicates that configurations combining C-O-C groups with adjacent carbon defects often exhibit the highest $2e^-$ ORR selectivity, which provides an optimal *OOH binding energy that favors H_2O_2 desorption over O-O bond cleavage[28].

- **(2) DFT models reconstruction and key computational results**

  In response to the comment, we have reconstructed our DFT models. The new model incorporates both a C-O-C functional group (ether) and an adjacent carbon vacancy defect within the graphene monolayer, reflecting the experimental features and representing a configuration associated with high $2e^-$ ORR selectivity in the literature. The revised model and corresponding calculations are presented in the updated manuscript and Supplementary Information.

  Based on this more rational model, we have recalculated the key mechanistic parameters. The results are as follows:

  (a) Water dissociation promoted by Ca^{2+} is confirmed on the reconstructed model.

  Despite the change in active sites configuration (from pristine ether to ether + adjacent vacancy defect), Ca^{2+} consistently lowers the activation energy barrier for water dissociation ($H_2O \rightarrow H^+ + OH^-$). This trend is qualitatively consistent with our original calculation and confirms that the promoting effect of Ca^{2+} on interfacial water dissociation is robust and not an artifact of a specific active site model.

  (b) STAB-modified surface (C18) exhibits weaker Ca^{2+} binding on the reconstructed model.

  We further constructed the corresponding STAB-modified surface model (C18) based on the new C0 substrate, with the quaternary ammonium surfactant electrostatically assembled on the surface. The positive shift in adsorption energy of Ca^{2+} confirms that the STAB modification effectively reduces the affinity of the cathode surface for Ca^{2+}, which remains valid across different active site configurations.

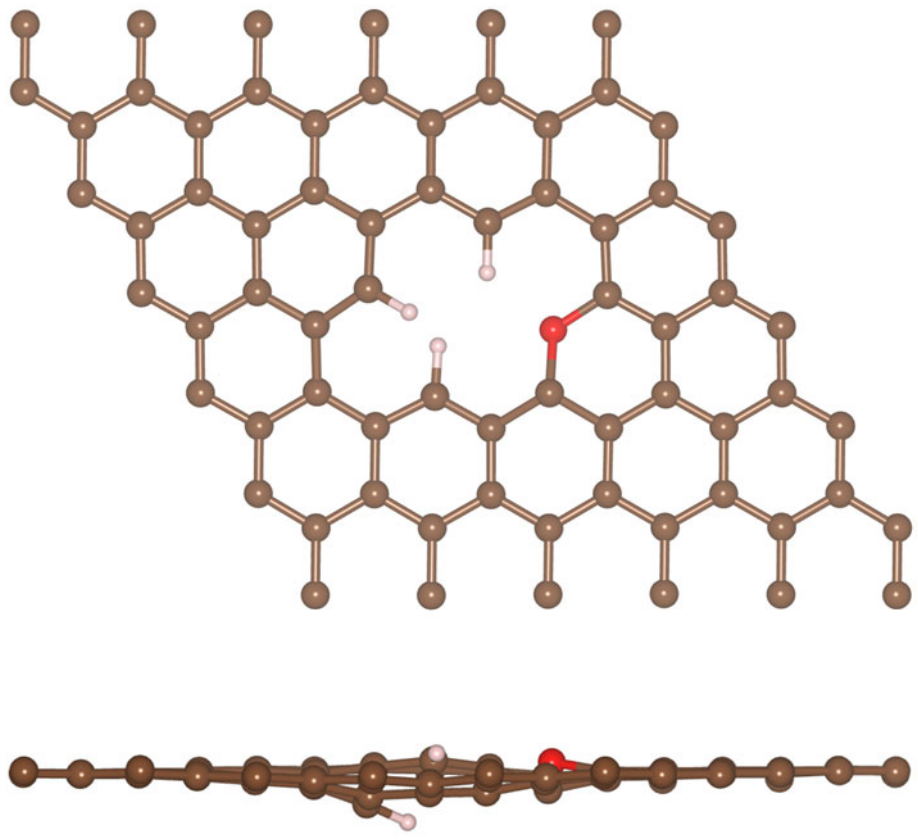

Figure Atomic configurations of the reconstructed model incorporating both a C-O-C ether group and an adjacent carbon vacancy defect within the graphene monolayer. The brown, red, and pink spheres represent C, O, and H atoms, respectively.

The Revised Content in Manuscript

In response to your comment, we have reconstructed our DFT model to incorporate both a C-O-C ether group and an adjacent carbon vacancy defect within the graphene monolayer, which captures the dominant surface chemistry of C0 and represents the type of active site configuration associated with high $2e^-$ ORR selectivity in the literature. The specific revisions in the manuscript and supporting information are consistent with the response to the fifth major issue.

2. Authors interpret results from electrochemical impedance spectroscopy (EIS) (e.g., "hydrogen adsorption resistance") as evidence of slower proton transfers. They should provide the equivalent circuit, fitting quality, parameter identifiability, and a clearer connection between these parameters, and the surface availability of protons.

Response:

Thank you for this detailed comment regarding our EIS data analysis and interpretation. We fully agree that these details are essential for establishing the credibility of our EIS-based mechanistic claims. Below, we address each concern comprehensively.

- **(1) Equivalent circuit model and physical interpretation**

The EIS data in this study were fitted using a modified equivalent circuit:

R_s + [T ∥ (R_1 + (R_2 ∥ $C_φ$))], where:

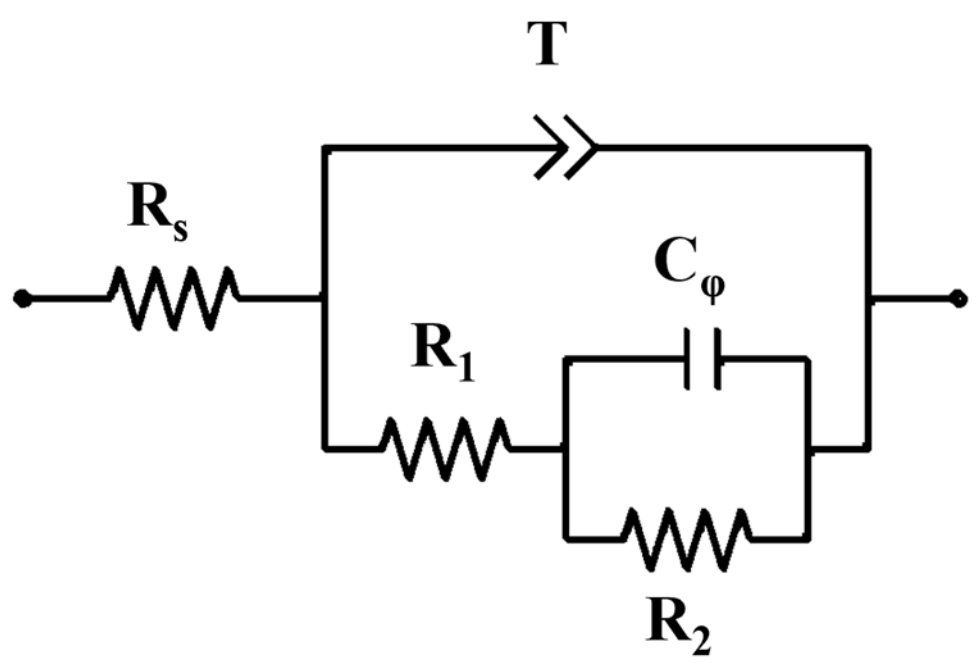

Figure. The equivalent circuit.

R_s: Ohmic series resistance, representing the combined solution resistance and contact resistance between the electrode and current collector.

R_1 ∥ T (high-frequency semicircle): This parallel combination represents the electron transfer process at the cathode-electrolyte interface. R_1 is the high-frequency charge transfer resistance associated with the Faradaic reduction reaction of H_2O_2 to H_2O. T accounts for the non-ideal double-layer response. A lower R_1 value indicates faster electron transfer kinetics and a higher intrinsic rate of H_2O_2 reduction.

R_2 ∥ $C_φ$ (low-frequency semicircle): This parallel combination represents the low-frequency relaxation of proton-coupled adsorbed intermediates. R_2 reflects the resistance associated with proton/hydrogen adsorption, which is the prerequisite step for proton-coupled H_2O_2 reduction pathway. $C_φ$ describes the corresponding pseudo-capacitance. Given that the protons must be generated in-situ via water dissociation ($H_2O \rightarrow H^+ + OH^-$) under the locally alkaline environment, R_2 is a direct indicator of proton availability and accessibility at the electrode-electrolyte interface. A larger R_2 represents greater kinetic resistance to proton/hydrogen adsorption and lower effective proton availability.

- **(2) Fitting quality, parameter statistics and identifiability**

All the original impedance data were fitted using ZView software and the same equivalent circuit (as shown above) was used for all samples. The fitted curves with the experimental Nyquist plots are provided in **Fig. 3k, Supplementary Fig. 12, 24** and **46** for all representative spectra. The key fitted parameters and the corresponding fitting residuals are now provided in the revised Supplementary Information (**Supplementary**

Tables 2-4). All parameters were well-determined with relative standard errors below 15%.

- **(3) Connection between R_2 and the surface availability of protons**
  We appreciate the request for a clearer mechanistic connection between the fitted resistance parameters and the interfacial proton availability. R_2 quantifies the kinetic resistance associated with proton-coupled H_2O_2 reduction pathway. Under the locally alkaline conditions at the operating cathode, protons are scarce and typically supplied via water dissociation ($H_2O \rightarrow H^+ + OH^-$). Therefore, a larger R_2 indicates greater resistance to proton transfer and lower effective proton availability.
  Ca^{2+} system vs. Na^+ system (C0/C18 cathode, Fig. 1e): R_2 in the Ca^{2+} system is consistently lower than in the Na^+ system. This is mechanistically consistent with our in-situ ATR-SEIRAS results (**Fig. 1g-i**) and DFT finding (**Fig. 1k**) that Ca^{2+} facilitates water dissociation and enhances proton accessibility—precisely the mechanism we propose for how dissolved Ca^{2+} suppresses $2e^-$ ORR selectivity.
  C18 vs. C0 (Figs. 1f and 3i): C18 exhibits significantly larger R_2 than C0. This is consistent with the hydrophobic alkyl chains of STAB disrupting the interfacial hydrogen-bond network, as revealed by MD simulations (**Fig. 4h**), which impedes proton transport and reduces proton availability at the electrode surface. The increased R_2 reflects a diminished ability of protons to access and adsorb onto the cathode, thereby suppressing the parasitic H_2O_2 reduction pathway.
  Scaled cathodes vs. fresh cathodes (Figs. 3k and Supplementary Fig. 24): Scaled cathodes exhibit larger R_2 than fresh cathodes. The precipitated $CaCO_3$ scale layer physically impedes proton access to the electrode surface, which we identify as the "protective" mechanism through which scale partially suppresses H_2O_2 reduction.
- **(4) Integration of EIS evidence with the broader mechanistic narrative**
  The EIS data, interpreted through the equivalent circuit model $R_s + [T \parallel (R_1 + (R_2 \parallel C_\varphi))]$, provide critical kinetic evidence that bridges our experimental observations and theoretical calculations. This consistent convergence of electrochemical kinetics (EIS), experimental evidence (H_2O_2 decomposition kinetics, RRDE deconvolution analysis, and in-situ ATR-SEIRAS results), and theoretical calculations (DFT and MD) provides a robust and coherent mechanistic picture.

  The Revised Content in Manuscript
  The equivalent circuit diagram has been inserted into all EIS-related figures in both the manuscript and the Supplementary Information, and the physical interpretation of each parameter has been specified in the corresponding figure captions. The fitting results and associated fitting errors of the key parameters are presented in the following tables:
- **New Tables in Supporting Information**

Supplementary Table 2. EIS fitting results of C0 and C18 measured in N_2-saturated 0.1M NaCl electrolyte containing 10mM H_2O_2 at different potentials.

Electrode	E (V vs. RHE)	R_s (Ω)	Error (%)	R_1 (Ω)	Error (%)	R_2 (Ω)	Error (%)
C0	0.45V	10.16	0.37	7.09	3.54	353.3	3.82
C0	0.40V	10.11	0.38	6.95	3.48	180.3	2.50
C0	0.35V	10.07	0.36	6.72	3.42	101.0	1.84
C0	0.30V	9.97	0.42	6.32	4.00	63.5	1.83
C18	0.45V	10.19	0.47	5.89	4.36	2352	12.00
C18	0.40V	10.04	0.48	5.63	4.16	702.2	5.17
C18	0.35V	9.95	0.45	5.32	3.83	233.6	3.99
C18	0.30V	9.87	0.40	5.16	3.46	103	2.69

Supplementary Table 3. EIS fitting results of C0 and C18 measured in N_2-saturated 0.1M $CaCl_2$ electrolyte containing 10mM H_2O_2 at different potentials.

Electrode	E (V vs. RHE)	R_s (Ω)	Error (%)	R_1 (Ω)	Error (%)	R_2 (Ω)	Error (%)
C0	0.45V	4.95	0.40	4.50	4.60	180.9	1.99
C0	0.40V	4.92	0.36	4.27	4.05	97.5	1.39
C0	0.35V	4.87	0.36	3.95	3.99	63.7	1.28
C0	0.30V	4.84	0.31	3.76	3.67	41.0	1.14
C18	0.45V	4.77	0.79	2.85	3.13	973.1	6.10
C18	0.40V	4.94	0.61	2.74	3.87	357	4.37
C18	0.35V	4.82	0.64	2.84	3.84	130.5	2.56
C18	0.30V	5.03	0.48	2.54	3.17	59.8	1.68

Supplementary Table 4. EIS fitting results of C0 and C18 cathodes with varying degrees of scaling measured in N_2-saturated 0.1 M Na_2SO_4 solution containing 10 mM H_2O_2.

Electrode	R_s (Ω)	Error (%)	R_1 (Ω)	Error (%)	R_2 (Ω)	Error (%)
No-scale C0	11.98	0.71	9.00	4.75	89.3	10.58
0.1A-scale C0	12.12	0.43	7.31	3.25	110.6	5.97
0.4A-scale C0	12.48	0.51	6.04	3.56	181.1	5.10
No-scale C18	12.03	0.61	8.53	4.46	157.7	11.85
0.1A-scale C18	12.20	0.42	7.13	3.19	191.4	9.10
0.4A-scale C18	12.64	0.51	5.98	3.88	332.8	8.13

3. Authors should discuss the diffusion and H-type cell experiments used to support Ca^{2+} exclusion more carefully with respect to their transferability to polarized cathode surfaces.

Response:

Thank you for raising this important point regarding our diffusion experiments. We acknowledge that the diffusion experiments used to support Ca^{2+} exclusion were driven solely by concentration gradients, which differ from the polarized cathode surfaces where an applied electric field strongly influences ion migration and interfacial distribution. We appreciate the opportunity to discuss the transferability and limitations of these experiments more carefully and have conducted additional measurements to address this concern.

- **(1) We fully acknowledge the inherent limitation of the open-circuit diffusion experiments.**

  In our Ca^{2+} diffusion experiments (**Fig. 4a** and **4b**), ion transport is driven purely by a concentration gradient across the glass fiber membrane. Under these conditions, the measured Ca^{2+} flux reflects the passive interaction between the Ca^{2+} and the C0 or C18 surfaces—specifically, electrostatic attraction/repulsion and hydrophilic/hydrophobic interaction. In fact, at a polarized cathode during ORR operation, ion transport is affected by electromigration under the applied electric field. The steady-state interfacial ion concentrations at a polarized cathode are determined by the balance of electromigration, diffusion and convection, which cannot be fully replicated in the open-circuit diffusion setup.

  We have now explicitly acknowledged this limitation in the revised manuscript and have clarified that the diffusion experiments provide qualitative evidence for the interaction between Ca^{2+} and the C0/C18 surfaces, rather than a quantitative prediction of interfacial ion concentrations under polarization.

- **(2) Measurement of electric field-induced Ca^{2+} adsorption**

  To more accurately capture the difference in Ca^{2+}-electrode interactions between C0 and C18 under polarized conditions, we employed a previously reported electrode transfer method to quantify electric-field-driven Ca^{2+} adsorption on both electrodes[16,26]. Detailed experimental procedures are provided below. The results demonstrate that under our operating conditions, the electro-adsorbed Ca^{2+} amount on C18 is significantly lower than that on C0 (3.28 mg/L vs. 14.99 mg/L), further confirming the substantially reduced affinity of C18 toward Ca^{2+} and consequently mitigating the inhibitory effect of dissolved Ca^{2+} on the $2e^-$ ORR.

The Revised Content in Manuscript

Based on your comments, we have explicitly acknowledged the limitation of diffusion experiments and employed an electrode transfer method to quantify electric-field-driven Ca^{2+} adsorption on both electrodes. The following specific revisions have been implemented in the manuscript and supporting information:

- **New discussion in Section "Mechanistic study for enhanced Ca^{2+}-tolerance of surfactant-modified cathodes." [Line 439-447, Page 14 (Revised)]**

These diffusion measurements provide qualitative evidence for the intrinsic Ca^{2+}-surface interactions under open-circuit conditions. To more directly probe Ca^{2+}-electrode interactions under polarized conditions, an electrode transfer method was employed to quantify electric-field-driven Ca^{2+} adsorption[16,26]. The electro-adsorbed Ca^{2+} amount on C18 (3.28 mg/L) was significantly lower than that on C0 (14.99 mg/L) (**Supplementary Fig. 53**), further confirming the substantially reduced affinity of C18 toward Ca^{2+}. This effective exclusion of Ca^{2+} significantly mitigates the inhibitory effect of dissolved Ca^{2+} on the 2e$^-$ ORR, representing a key factor in the superior Ca^{2+}-resistance of C18.

- **New Supplementary Fig. 53 in Supplementary Information:**

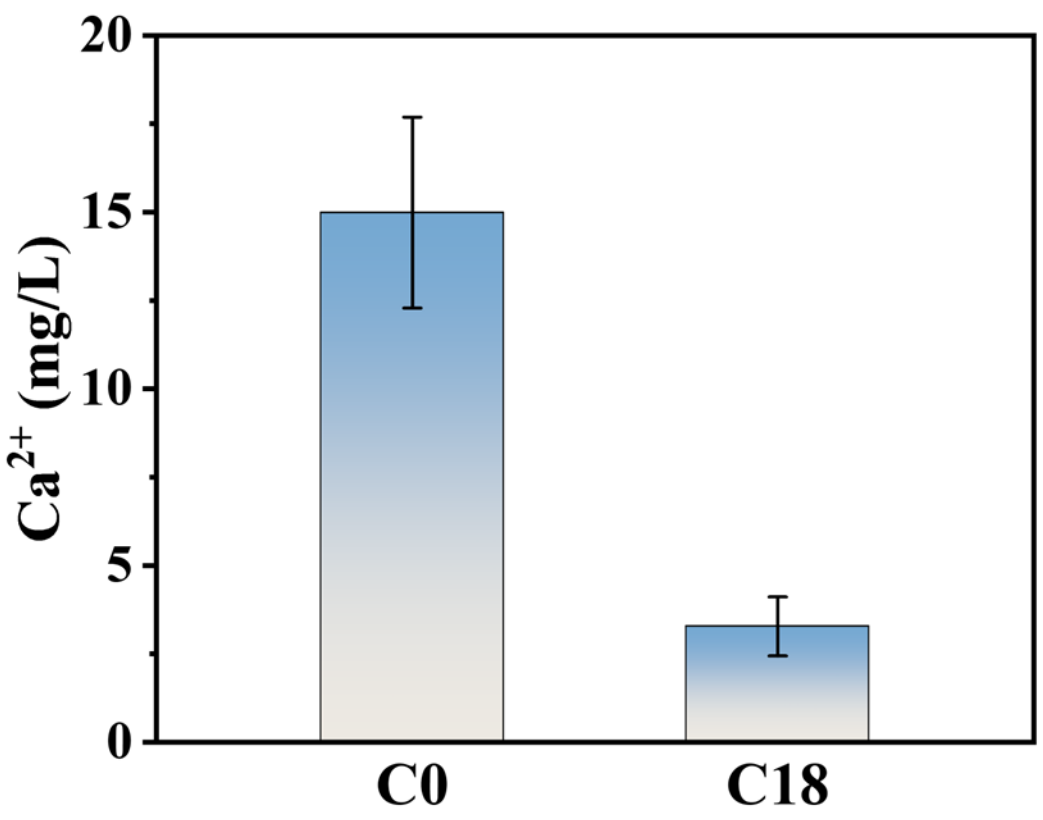

Supplementary Fig. 53 The electric-field-driven Ca^{2+} adsorption concentration detected by ICP-OES for the C0 and C18 cathode.

- **Updated Methods Section**
  Measurement of electric-field-induced Ca^{2+} adsorption [Line 816-823, Page 25 (Revised)]
  The Ca^{2+} adsorbed on the electrode surface under an applied electric field was quantified using an electrode transfer method[16,26]. Specifically, the electrode was operated at -0.3 V vs. Ag/AgCl in 0.1 M $CaCl_2$ solution for 120 s. Subsequently, the electrode was rapidly transferred from the electrolyte into 30 mL of ultrapure water while maintaining constant potential. The applied potential was then disconnected to release the adsorbed Ca^{2+} into the pure water, and the Ca^{2+} concentration was quantified by ICP-OES.

- **New reference**

26. Yu, Y. et al. Regulating active hydrogen supply and intermediate binding for pH-universal H_2O_2 electrosynthesis at ampere-level current density. *Nature Communications* **16**, 10784 (2025).

4. Authors mention that C18 differs from C0 not only in surfactant grafting but also in defect level and possibly surface chemistry. They should clarify how these confounding factors are controlled.

Response:

Thank you for this careful observation and important comment. We agree that C18 differs from C0 not only by the introduction of the STAB surfactant, but may also exhibit slight changes in defect density and surface chemistry after modification. We appreciate the opportunity to clarify this issue.

- **(1) Acknowledgment and interpretation of the differences between C0 and C18**

  The STAB surfactant grafting indeed introduces several concomitant changes beyond the intended surface charge and wettability modifications. We acknowledge that these are unavoidable consequences of the STAB modification strategy. However, we demonstrate below that these differences do not confound our mechanistic conclusions.

  A slightly higher degree of defects of C18: Specifically, C18 exhibits a slightly higher I_D/I_G ratio compared to C0 (**Fig. 2c**). Nevertheless, in our study, C18 was prepared directly from the same oxidized carbon black precursor (C0) under mild post-modification conditions, without high-temperature treatment or additional oxidative activation. Therefore, the carbon framework was largely preserved during the modification. The defects of C18 exhibited only a marginal increase (I_D/I_G ratio from 1.03 to 1.07), which we consider insufficient to account for the significantly enhanced H_2O_2 electrosynthesis performance (a 168.66% improvement in H_2O_2 yield) in the presence of Ca^{2+}.

  A lower surface oxygen content of C18: Additionally, FTIR results revealed no significant difference in the surface oxygen functional groups between C0 and C18 (**Fig. 2d**), despite the XPS results indicating a decreased surface oxygen content on C18 relative to C0 (**Supplementary Fig. 28**). The C18 modification was mainly achieved through electrostatic interaction between the $-N(CH_3)_3^+$ cationic headgroups and negatively charged oxygen-containing groups on C0. Therefore, the oxygen functional groups are expected to be largely retained at the carbon surface, although some of them may become covered or electronically perturbed after surfactant binding. Because XPS is a surface-sensitive technique, the long alkyl chain layer introduced by C18 can attenuate the O 1s signal from the underlying oxidized carbon surface. In addition, the large contribution of carbon from the STAB surfactant increases the relative carbon signal, thereby decreasing the apparent surface atomic percentage of oxygen. Thus, the lower oxygen content observed by XPS is more likely associated with surface coverage/signal attenuation and compositional dilution by the C18 layer, rather than substantial removal of oxygen functional groups. The remaining surface oxygen species, together with the intrinsic carbon defects, continue to serve as active sites for the $2e^-$ ORR. This is evidenced by the slightly enhanced FE of C18 over C0 in Ca^{2+}-free electrolyte (**Supplementary Fig. 30**), confirming that the intrinsic $2e^-$ ORR activity is largely preserved.

- **(2) N-C18 control directly isolates the surfactant effect**

  As described in our response to the Major Issues 2 regarding the ablation study to elucidate which factor is more deterministic, we prepared an N-C18 control (modified

with 1-octadecanethiol) that introduces similar long alkyl chains but without the positively charged $-N(CH_3)_3^+$ groups. It is noteworthy that the N-C18 modification inevitably introduced additional changes similarly to those of C18. Compared with C0, N-C18 also exhibited a slightly increased defect density and a reduced surface oxygen content (**Supplementary Fig. 58**), with the underlying causes being analogous to those for C18. Nevertheless, in the presence of Ca^{2+}, N-C18 achieved only a 29.95% improvement in H_2O_2 electrosynthesis performance over C0, whereas C18 delivered a substantially greater enhancement of 168.66% (**Supplementary Fig. 59**), despite introducing comparable structural perturbations. These results directly confirm that the electrostatic Ca^{2+} repulsion from the more positively charged surface, rather than any general surfactant-induced changes in defect density or other surface chemistry, is the dominant factor responsible for the superior Ca^{2+} tolerance of C18.

The Revised Content in Manuscript

Detailed information regarding the preparation method, material characterization, and performance testing of N-C18, as well as the ablation study of C18, is provided in the response to **Major Comment 2**.

5. Authors should provide error bars, replicate numbers, and statistical treatment consistently for performance metrics (FE, production rate, stability) and scaling quantification (ICP/EDS/profilometry).

Response:

Thank you for this important suggestion regarding data presentation and statistical rigor. We fully agree that error bars, replicate numbers, and statistical treatment are essential for establishing the reliability and reproducibility of our results. We have carefully revised the manuscript and Supplementary Information to consistently report these details for all performance metrics and scaling quantification. The specific revisions have been implemented in the manuscript and Supplementary Information as follows:

- **(1) Performance metrics:**
  All H_2O_2 electrosynthesis performance data (FE, H_2O_2 concentration, and production rate) are now presented as mean ± standard deviation (SD) from three independent replicate experiments (n = 3).
- **(2) Scaling quantification:**
  ICP-OES measurements of dissolved and precipitated calcium species are presented as mean ± SD from three independent cathode samples (n = 3).
  EDS elemental quantification is reported with mean ± SD from three randomly selected areas on each sample (n = 3). The overlap images of scaled cathodes are shown below.

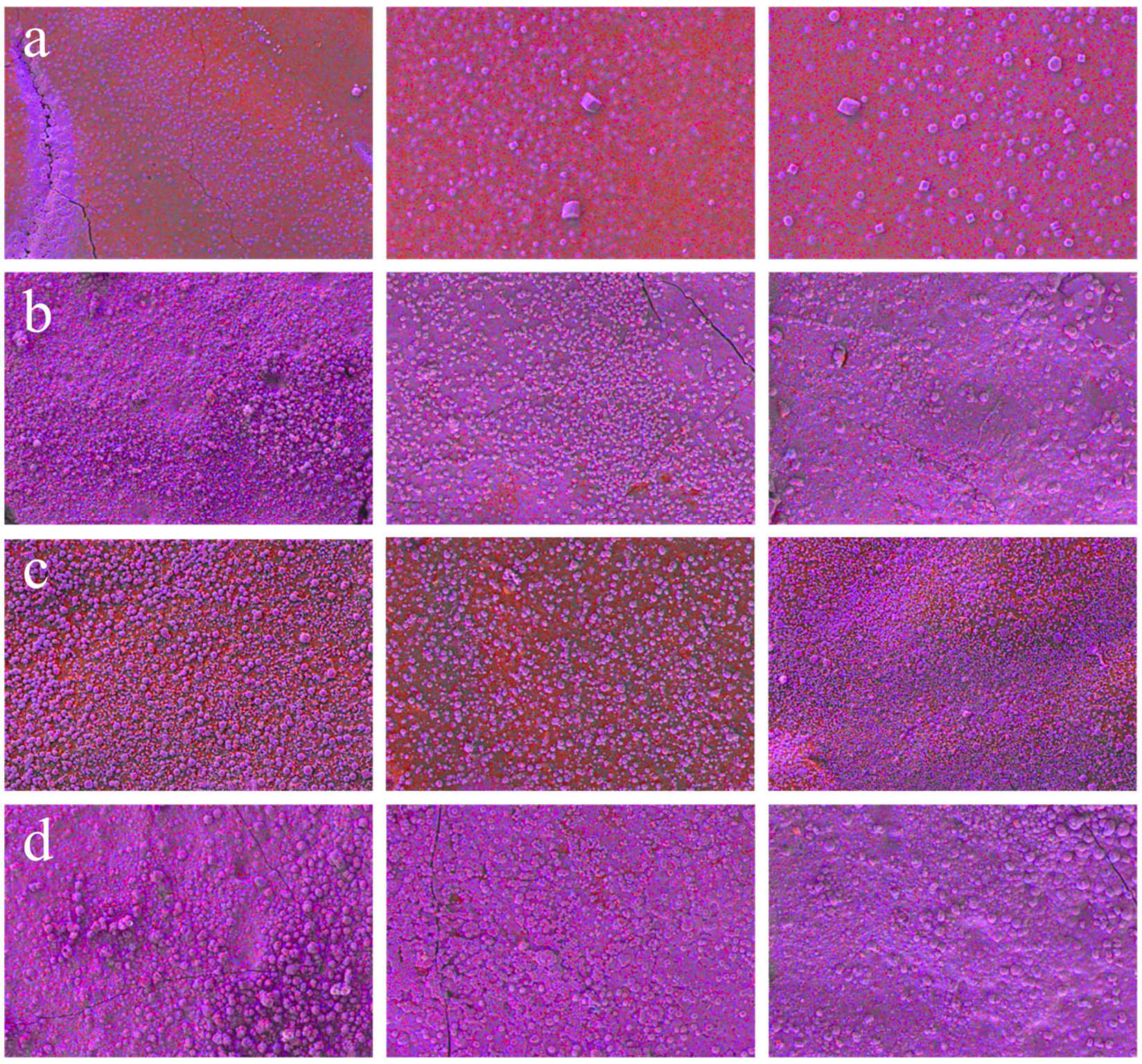

Figure EDS maps from three randomly selected areas of the C0 cathode after experiment at (a) 0.1A and (b) 0.4A in the presence of 200 mg/L Ca^{2+}. EDS maps from three randomly selected areas of the C18 cathode after experiment at (c) 0.1A and (d) 0.4A in the presence of 200 mg/L Ca^{2+}.

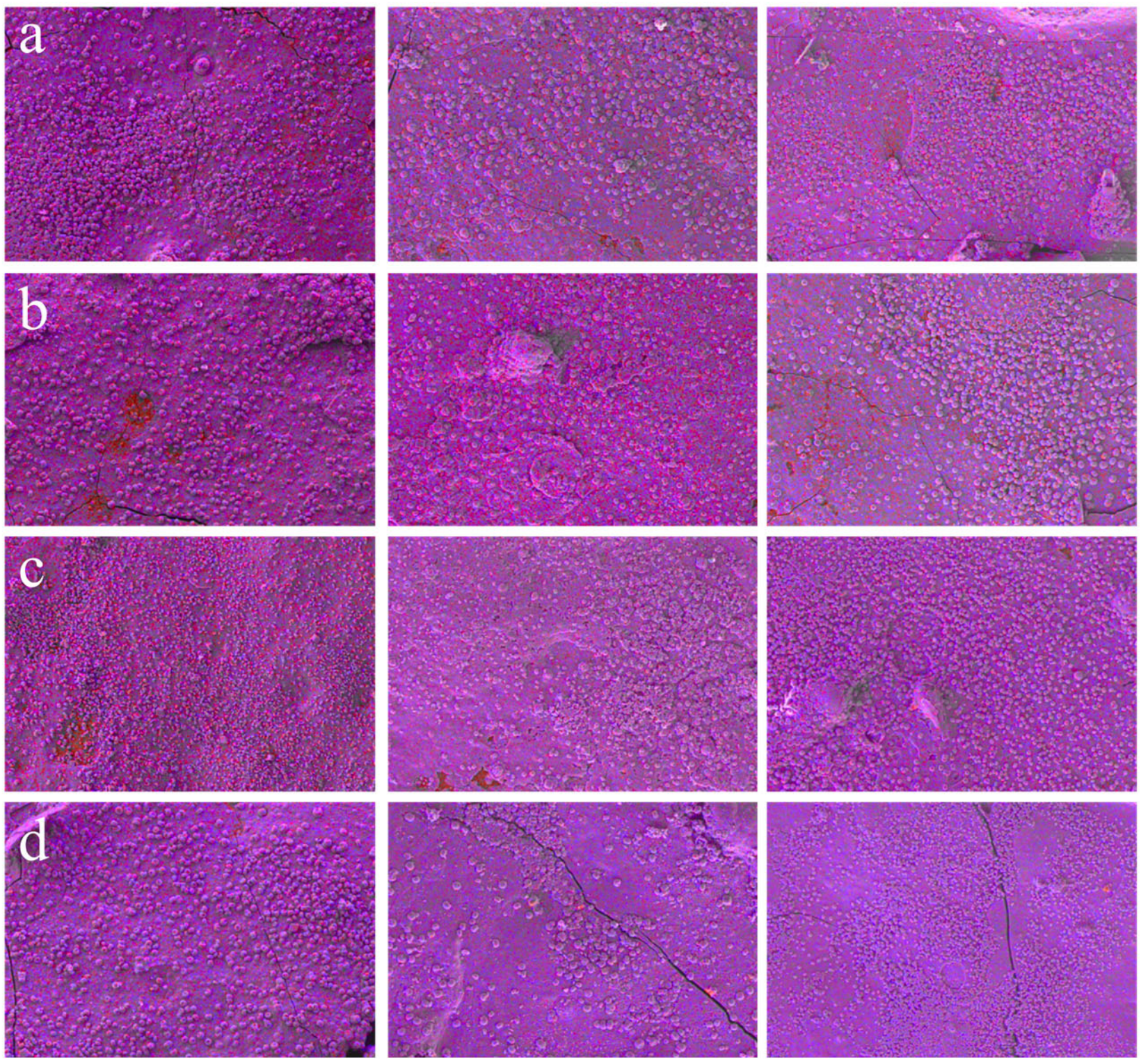

Figure EDS mappings from three randomly selected areas of the C0 cathode after experiments in (a) 20PPI-PUS-fenced and (b) 60PPI-PUS-fenced systems at 0.1A in the presence of 200 mg/L Ca^{2+}. EDS mappings from three randomly selected areas of the C18 cathode after experiments in (a) 20PPI-PUS-fenced and (b) 60PPI-PUS-fenced systems at 0.1A in the presence of 200 mg/L Ca^{2+}.

The Revised Content in Manuscript

- **New Supplementary Table 1 in Supporting Information:**

Supplementary Table 1. The surface Ca element distribution of scaled cathodes obtained from EDS tests.

Electrode	Ca content (at%)	Ca content (at%)	Ca content (at%)	Mean (at%)	SD (at%)
C0-0.1A-scale	8.09	7.68	9.33	8.37	0.86
C0-0.4A-scale	22.85	18.49	19.30	20.21	2.32
C18-0.1A-scale	10.09	9.66	12.32	10.69	1.43
C18-0.4A-scale	24.85	20.33	17.69	20.96	3.62
C0-20PUS-scale	19.92	18.12	18.87	18.97	0.90
C0-60PUS-scale	20.95	18.64	17.44	19.01	1.78
C18-20PUS-scale	22.95	18.75	18.70	20.13	2.44
C18-60PUS-scale	20.96	21.26	18.83	20.35	1.32

- **Replaced Figures in Manuscript:**

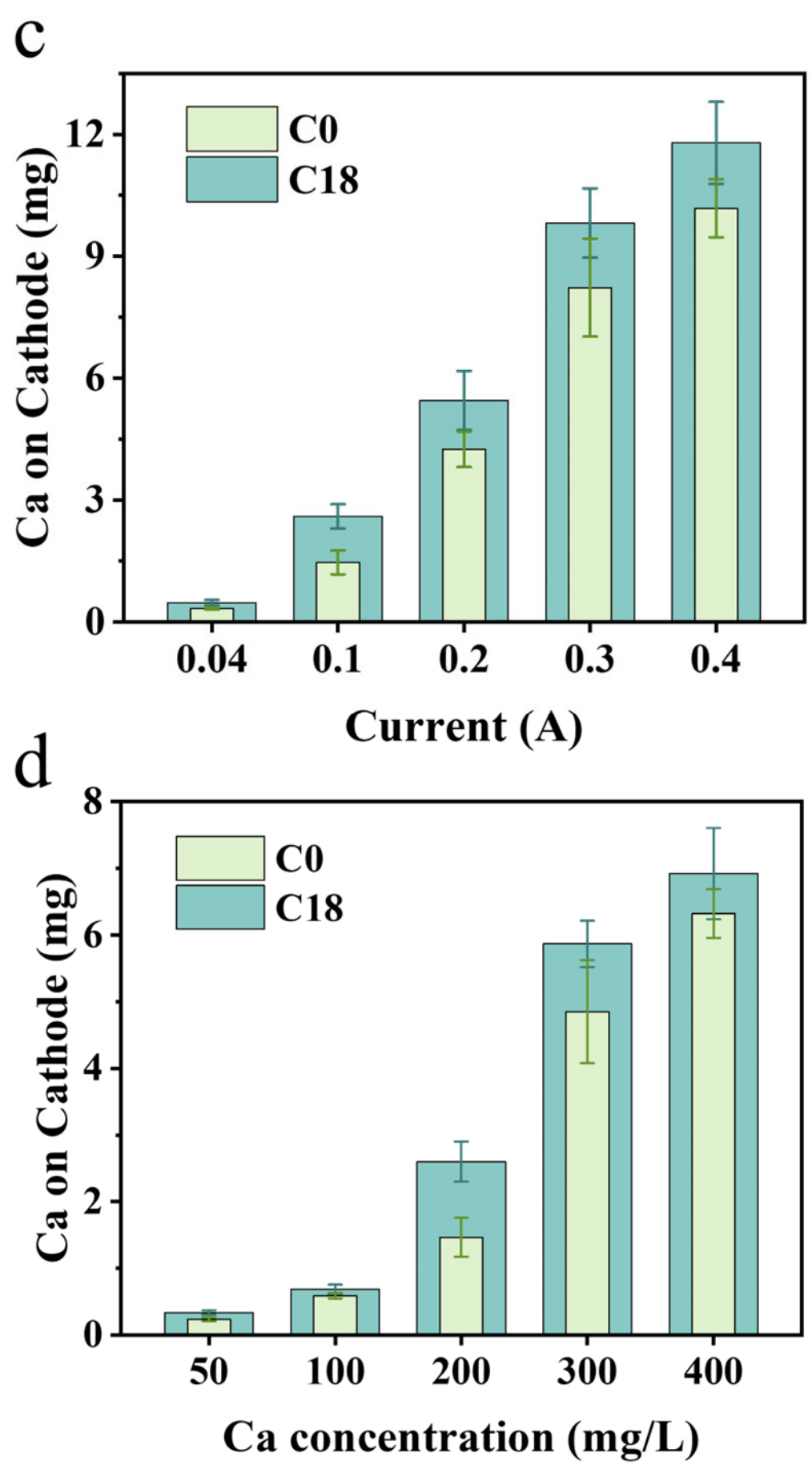

Fig. 3. Superior H_2O_2 electrosynthesis performance of C18. The mass of calcium scale on cathode under different (c) current densities and (d) initial Ca^{2+} concentrations.

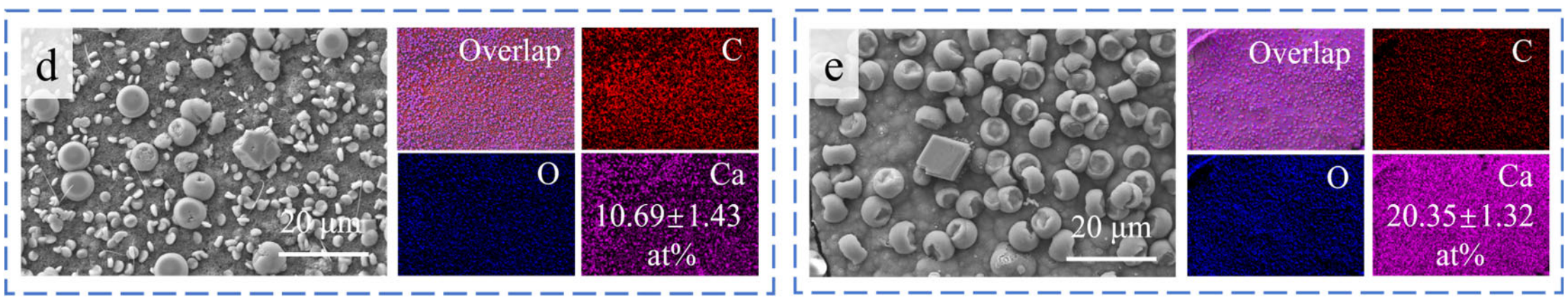

Fig. 5. H_2O_2 electrosynthesis performance enhanced by fenced cathode structure employing polyurethane sponges (PUS). SEM images and EDS mappings of the C18 cathode (d) without and (e) with PUS (60PPI) after experiment at 0.1A in the presence of 200 mg/L Ca^{2+}.

- **Replaced Figures in Supplementary Information:**
  Supplementary Fig. 1 in the original Supplementary Information has been replaced and is now renumbered as **Supplementary Fig. 2**.

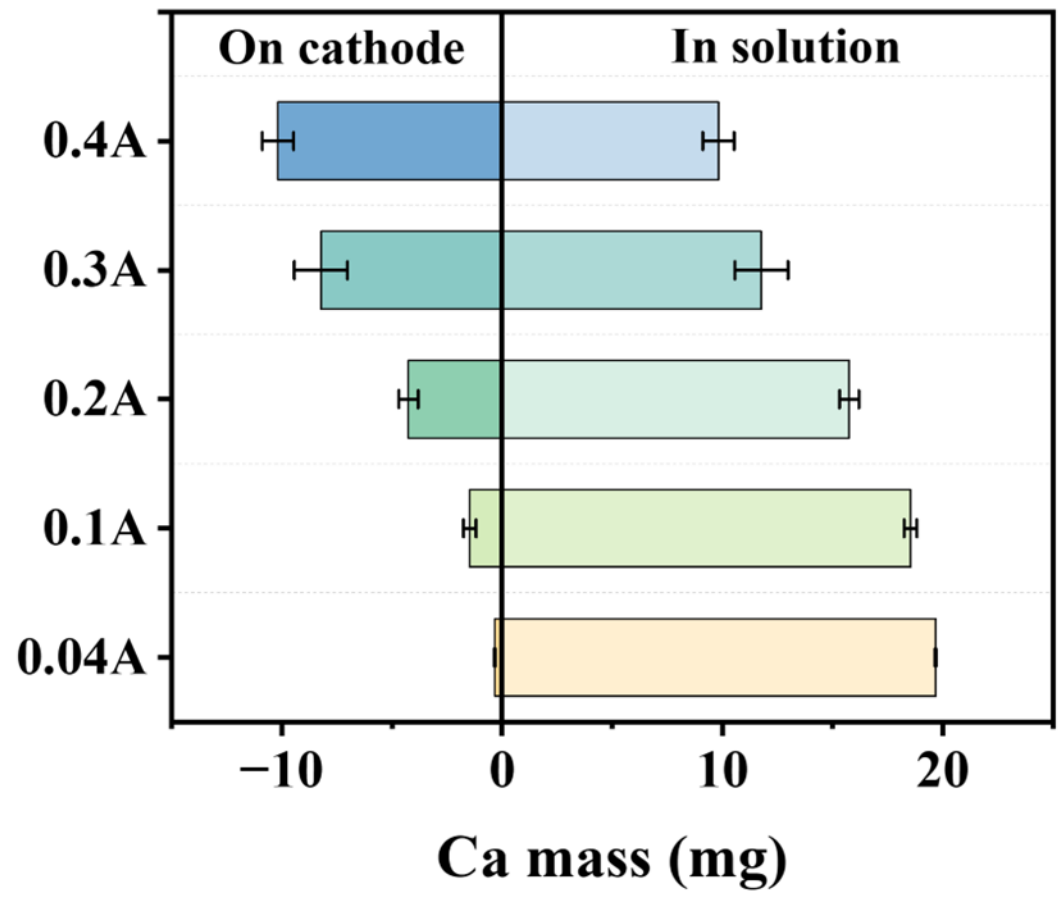

Supplementary Fig. 2. Distributions of two calcium species under different current densities for C0 cathode. Reaction conditions: [Na_2SO_4] = 0.05 M, [Ca^{2+}]$_0$ = 200 mg/L, a rotation speed of 600 rpm, an initial pH of 7 and electrolysis of 3 h.

The EDS maps in Supplementary Figs. 2, **35**, **57**, and **58** in the original Supplementary Information have been replaced and are now renumbered as **Supplementary Figs. 3**, **40**, **69**, and **70**, respectively.

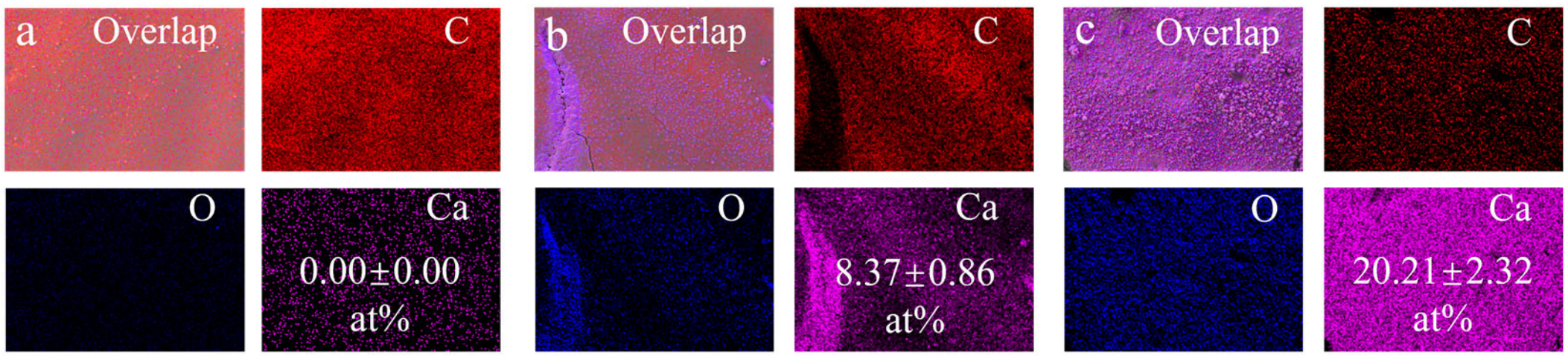

Supplementary Fig. 3. SEM images and EDS maps of the C0 cathode (a) before experiment, (b) after experiment at 0.1A and (c) after experiment at 0.4A. Reaction conditions: [Na_2SO_4] = 0.05 M, [Ca^{2+}]$_0$ = 200 mg/L, a rotation speed of 600 rpm, an initial pH of 7 and electrolysis of 3 h.

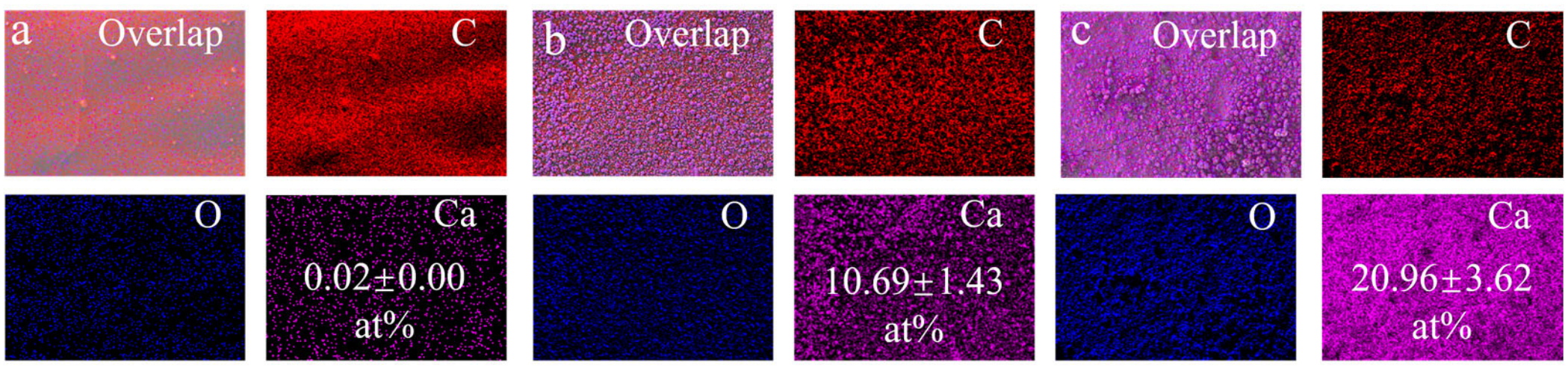

Supplementary Fig. 40. SEM images and EDS maps of the C18 cathode (a) before experiment, (b) after experiment at 0.1A and (c) after experiment at 0.4A. Reaction conditions: [Na_2SO_4] = 0.05 M, [Ca^{2+}]$_0$ = 200 mg/L, a rotation speed of 600 rpm, an initial pH of 7 and electrolysis of 3 h.

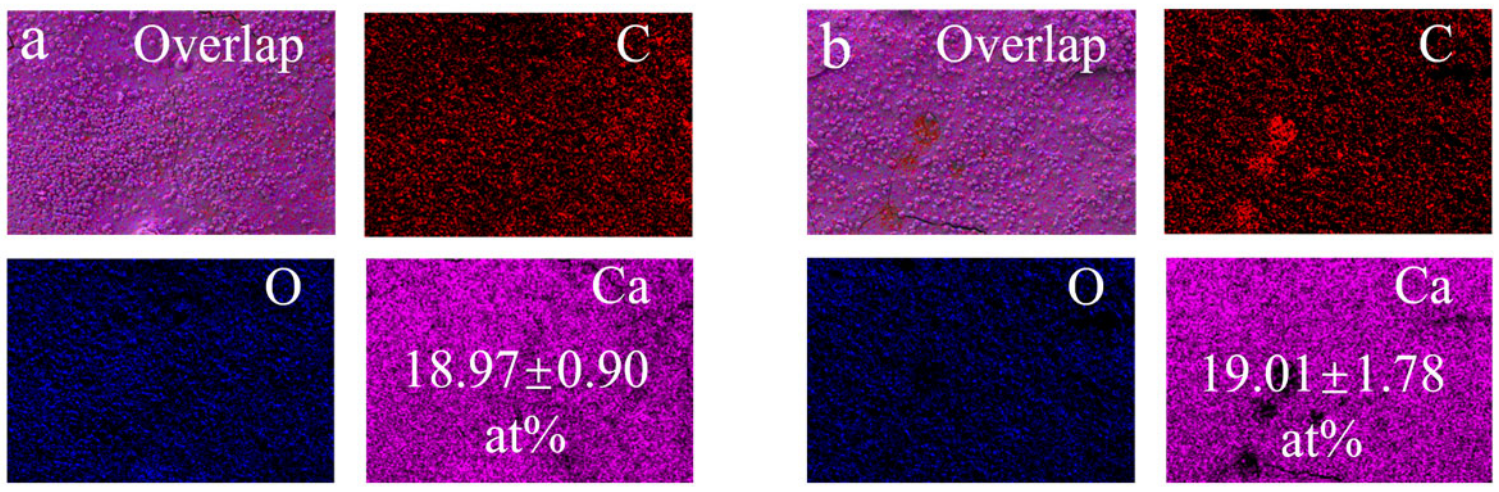

Supplementary Fig. 69. SEM images and EDS mappings of the C0 cathode after experiments in (a) 20PPI-PUS-fenced and (b) 60PPI-PUS-fenced systems at 0.1A in the presence of 200 mg/L Ca^{2+}.

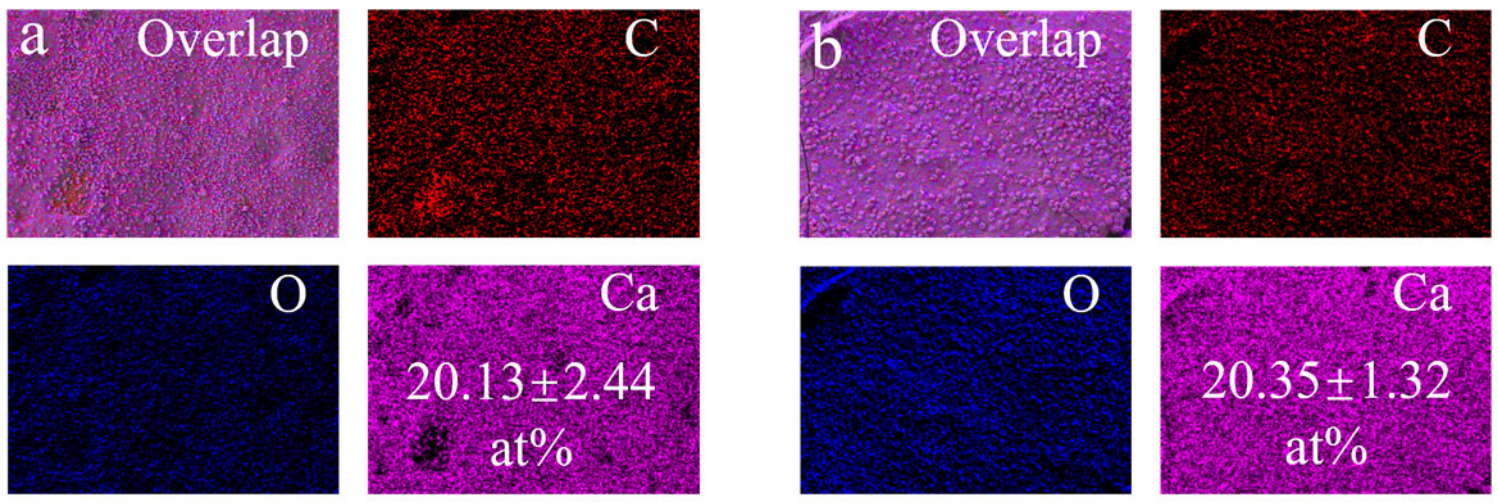

Supplementary Fig. 70. SEM images and EDS mappings of the C18 cathode after experiments in (a) 20PPI-PUS-fenced and (b) 60PPI-PUS-fenced systems at 0.1A in the presence of 200 mg/L Ca^{2+}.

Modifications to comments from Reviewer #4

General comments

I co-reviewed this manuscript with one of the reviewers who provided the listed reports. This is part of the Nature Communications initiative to facilitate training in peer review and to provide appropriate recognition for Early Career Researchers who co-review manuscripts.

Response:

Thank you for your efforts in co-reviewing this manuscript and for your valuable contribution to the peer review process. All comments raised during the review process have been carefully addressed in our revised manuscript. Detailed point-by-point responses to each comment have been provided in the accompanying Response to Reviewers document. We respectfully invite you to review the corresponding responses, and we hope that the revisions can adequately address the concerns raised.

<u>Modifications to comments from Reviewer #1</u>

General comments

In the revised manuscript, the author conducted an extensive series of experiments and measurements, including RRDE tests and ATR-SEIRAS, to strengthen the empirical foundation of the study. Additionally, the authors have carefully adjusted the DFT calculations, including modifying the model to align better with realistic conditions and incorporating the effects of applied potential and interfacial electric fields. The revised manuscript has seen significant improvements in terms of logical structure, data support, and the depth of argumentation. In my opinion, the current manuscript meets the high standard of Nature Communications. I'm pleased to recommend that the editorial team accept this manuscript.

Response:

We sincerely thank you for the positive assessment of our revised manuscript and for recognizing the additional experimental and theoretical efforts undertaken in this revision. We greatly appreciate your constructive comments throughout the review process, which have helped us improve the logical organization, experimental support, and overall depth of the manuscript. We are grateful for your recommendation for acceptance.

Modifications to comments from Reviewer #2

General comments

The authors have significantly revised the manuscript and have addressed the major concerns raised in my previous review. I appreciate the substantial efforts made to strengthen the mechanistic understanding, improve experimental validation, and clarify the scope of the proposed strategy. The revised manuscript is considerably improved compared with the original version.

In particular, the authors have provided additional evidence regarding the interfacial microenvironment, the role of dissolved Ca^{2+} versus precipitated calcium scale, and the influence of scaling on H_2O_2 selectivity. These new results, together with the revised discussion, substantially strengthen the proposed mechanism and alleviate my previous concerns that the conclusions relied primarily on indirect electrochemical evidence. Regarding the theoretical calculations, the authors have comprehensively revised the theoretical calculations by reconstructing the interfacial model and incorporating electrochemical field constraints into the simulations. These improvements address my previous concerns regarding the oversimplified surface model and the absence of potential-dependent interfacial effects. The authors have also appropriately revised several statements that were previously too broad, especially regarding the universality of the approach in natural water environments. The updated discussion now better acknowledges the complexity of real water matrices and the potential influence of other ions and environmental factors. Furthermore, the authors have clarified the role of the engineering strategies introduced in the latter part of the manuscript, improving the overall logical consistency of the study.

Overall, I believe that the revised manuscript presents a more convincing and comprehensive study. The concept that dissolved calcium ions and calcium scale can exert distinct and even opposite effects on electrochemical H_2O_2 production is interesting and provides valuable insights into the design of electrocatalytic systems operating under realistic aqueous conditions. The major concerns raised previously have been adequately addressed, and the manuscript has reached a level suitable for publication.

Response:

We sincerely thank you for the careful evaluation of our revised manuscript and for acknowledging the substantial improvements in the mechanistic analysis, experimental validation, theoretical modeling, and overall logical structure. We greatly appreciate your constructive comments throughout the review process, which have helped us clarify the distinct roles of dissolved Ca^{2+} and calcium scale and improve the scientific rigor, clarity, and overall quality of the manuscript. We are grateful for your positive assessment and recommendation for publication.

<u>Modifications to comments from Reviewer #3</u>

General comments

The authors have addressed my questions. The paper can be published.

Response:

We sincerely thank you for the careful evaluation of our manuscript and for confirming that our revisions have adequately addressed the previous questions. We greatly appreciate your constructive comments throughout the review process and the recommendation for publication.

Modifications to comments from Reviewer #4

General comments

I co-reviewed this manuscript with one of the reviewers who provided the listed reports. This is part of the Nature Communications initiative to facilitate training in peer review and to provide appropriate recognition for Early Career Researchers who co-review manuscripts.

Response:

Thank you for your efforts in co-reviewing this manuscript and for your valuable contribution to the peer review process. We greatly appreciate your positive assessment and recommendation for acceptance.
